# Pd-Catalyzed Direct Diarylation of Sodium Hypophosphite Enables the Synthesis of Diarylphosphonates

**DOI:** 10.3390/molecules30071564

**Published:** 2025-03-31

**Authors:** Jin Yang, Dangwei Qian, Gangwei Wang, Shangdong Yang

**Affiliations:** 1 State Key Laboratory of Applied Organic Chemistry, Lanzhou University, Lanzhou 730000, China; yangjin2021@lzu.edu.cn (J.Y.); qiandw16@lzu.edu.cn (D.Q.); wanggw@lzu.edu.cn (G.W.); 2 State Key Laboratory for Oxo Synthesis and Selective Oxidation, Lanzhou Institute of Chemical Physics, Chinese Academy of Sciences, Lanzhou 730000, China

**Keywords:** sodium hypophosphite, halogenated aromatic hydrocarbons, diarylphosphinates

## Abstract

A facile and efficient method for synthesizing diarylphosphinates from alcohols and aryl halides, using stable, green, and readily available sodium hypophosphite as a phosphorus source, is disclosed herein for the first time. This method offers high-efficiency and excellent functional group tolerance, providing a straightforward approach to synthesizing a broad range of diarylphosphinates from green starting materials with moderate to excellent yields.

## 1. Introduction

**Scheme 1:**
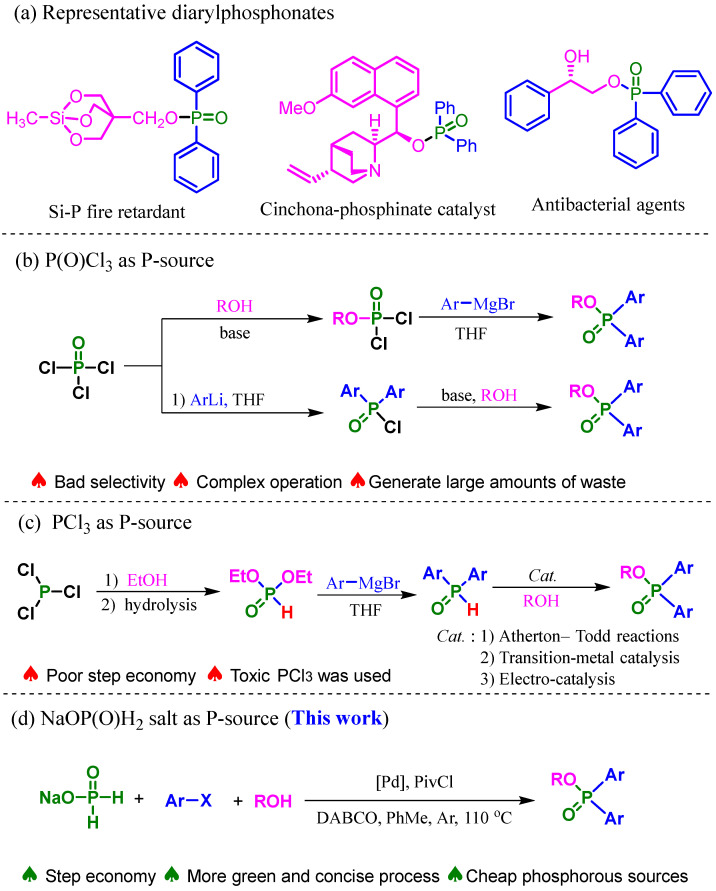
Synthesis of diarylphosphinate compounds.

Diarylphosphinates are a class of functional molecules with significant applications in pharmaceuticals [1,2], agrochemicals [3,4], flame retardants [5], ligand scaffolds [6,7], and organic synthesis [8,9]. Representative molecules are illustrated in Scheme 1a. Traditional synthetic methods primarily rely on the moisture-sensitive and toxic phosphorus oxychloride (POCl_3_) as a phosphorus source (Scheme 1b). These methods generally follow two transformation pathways: First, phosphorus oxychloride reacts with alcohol to yield dichlorophosphates, which subsequently undergo reactions with aryl magnesium bromide reagents to form diarylphosphinates [10,11]. Second, phosphorus oxychloride first reacts with aryl lithium to generate diarylphosphonyl chloride, which then undergoes nucleophilic substitution with alcohol under basic conditions to produce diarylphosphinates [12,13,14]. Furthermore, phosphorus trichloride (PCl_3_) has also been explored as the starting material, with the synthesis of diaryl phosphine oxide as a key intermediate. This intermediate can then react with alcohol through various strategies, such as the Atherton–Todd process [15,16,17,18], transition-metal-catalyzed coupling [19,20,21,22,23,24], Selectfluor mediated reactions [25], and electro-catalysis [26,27,28,29], to afford diarylphosphinates (Scheme 1c). Although the reactions described above provide useful methods for the synthesis of diarylphosphinates, they have several drawbacks. These include the use of moisture-sensitive and toxic P(O)Cl_3_ and PCl_3_ as starting materials, the use of highly reactive aryllithium and aryl magnesium bromide reagents, which lead to poor selectivity and complex operations, poor step economy, and the generation of numerous halide salts, which limit the green and environmentally friendly nature of the process. Therefore, the development of a simple and efficient protocol for the preparation of diarylphosphinates remains highly desirable.

Sodium hypophosphite (NaH_2_PO_2_) is a green, safe, stable, and cost-effective phosphorus source, making it a promising substitute for traditional phosphorus trichloride. Using NaH_2_PO_2_ in the construction of organophosphorus compounds is more economical in terms of steps and significantly reduces the overall cost—both financially and environmentally [30]. Building on previous research [31,32,33,34,35], we directly utilized sodium hypophosphite as a phosphorus source and achieved the synthesis of diarylphosphinates through a palladium-catalyzed coupling reaction in a “one-pot” manner (Scheme 1d). Compared to previous methods, our approach is more step-efficient, simpler, and more economical, enabling the construction of three new bonds in a single pot.

## 2. Results and Discussion

### 2.1. Reaction Condition Optimization

In the initial study, we selected NaH_2_PO_2_, isopropyl alcohol, and bromobenzene as standard substrates to identify optimal conditions for the diarylphosphonates’ synthesis (Table 1; see Supplementary Materials for more details). Building on previous work, we first screened activators for sodium hypophosphite in the reaction. However, the target product was not observed when no activator was used, or when benzoyl chloride or sulfonyl chloride served as an activator. Notably, when trimethylacetyl chloride (PivCl) was used as an activator, the target product **3a** was obtained with an 83% yield (Table 1, entry 1–4). After identifying the optimal activator, we focused on investigating the influence of bases on the reaction. Through systematic screening, we found that inorganic bases yielded unsatisfactory results, whereas other organic bases such as DBU, Et_3_N, and DIPEA were all capable of catalyzing the reaction. However, DABCO remained the optimal choice (Table 1, entry 5–9). Subsequently, we investigated the effect of reaction concentration on the process. The reaction proceeded smoothly across a range of concentrations; however, optimal reaction efficiency and economic viability were achieved at a concentration of 0.1 mol/L (Table 1, entry 10–11). During catalyst optimization, we found that Pd(dppe)Cl_2_ and Pd(PPh_3_)_2_Cl_2_ also promoted the reaction, but with less efficiency than Pd(dppf)Cl_2_. Inexpensive metal salts such as nickel and cobalt did not exhibit catalytic activity (Table 1, entry 12–15). Solvent screening revealed that PhMe was the optimal solvent, yielding 83% of the target product **3a**. When THF and EtOAc were used as solvents, the yields of **3a** were 51% and 63%, respectively (Table 1, entry 16–18).

### 2.2. Expansion of Aryl Halide Substrates and Alcohol Substrates

**Scheme 2:**
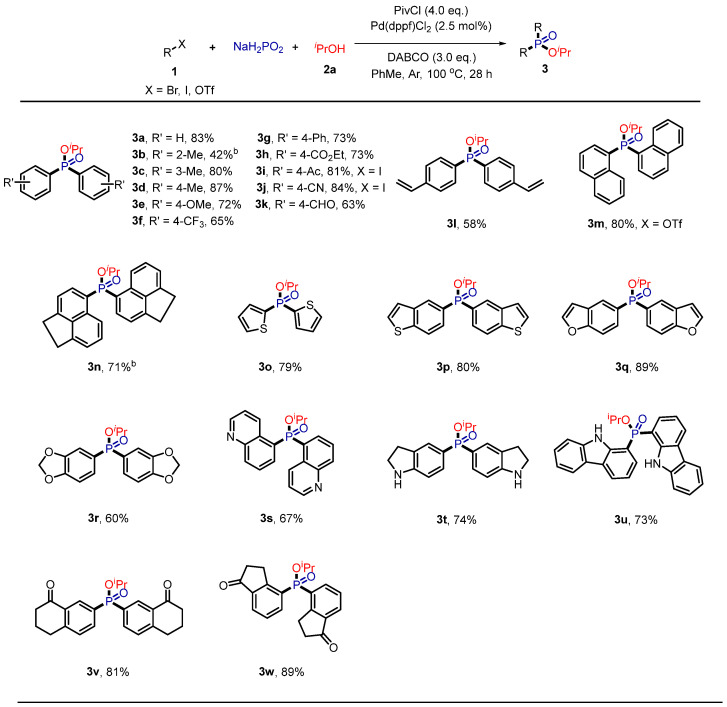
Expansion of aryl halide substrates ^a^. ^a^ Aryl halide **1** (0.2 mmol), sodium hypophosphite (0.4 mmol), isopropyl alcohol **2a** (1.6 mmol), PivCl (0.8 mmol), Pd(dppf)Cl_2_ (2.5 mol%), DABCO (0.6 mmol), PhMe (2.0 mL) under Ar at 100 °C for 28 h. ^b^ Under Ar at 120 °C for 28 h.

After establishing the protocol for the direct conversion of NaH_2_PO_2_ to arylphosphonate compounds, we next explored the scope of aryl halide coupling partners (Scheme 2). Bromobenzene was initially used as the coupling reagent, yielding isopropyl diphenylphosphonate **3a** in 83%. We then investigated the electronic and steric effects of substituents on the benzene ring. The results revealed that steric hindrance significantly affects the reaction. When o-methylbromobenzene, m-methylbromobenzene, and p-methylbromobenzene were used as substrates, only a small amount of the target product was obtained with o-methylbromobenzene. However, increasing the reaction temperature to 120 °C increased the yield of **3b** to 42%. The corresponding products (**3c**, **3d**) were obtained in 80% and 87% yields for m-methylbromobenzene and p-methylbromobenzene, respectively. When stronger electron-donating substituents, such as methoxy, were used, product **3e** was obtained with an 80% yield. Based on the aforementioned results, we observed that electron-donating substituents exhibited minimal influence on the reaction yields. Next, the effect of electron-withdrawing substituents in the para position of the benzene ring was investigated. The reaction showed good functional group compatibility, with trifluoromethyl, phenyl, ester, acetyl, and cyano groups all compatible. Diarylphosphonates (**3f**–**3j**) were obtained in good yields, indicating that electronic effects had minimal impact on the reaction outcome. Notably, when the aromatic ring carried aldehyde and vinyl groups in the para position, products **3k** and **3l** were obtained in 63% and 58% yields, respectively. Aromatic halides other than the benzene ring also proved suitable for the reaction, as demonstrated by the successful synthesis of biarylphosphates (**3m**, **3n**) from 1-naphthyl triflate and 5-bromoacenaphthene, yielding 80% and 71%, respectively. It is noteworthy that halogenated heteroaromatics can also participate in this reaction. For example, 2-iodothiophene, 5-bromobenzothiophene, 5-bromobenzofuran, and 4-bromo-1,2-methylenedioxybenzene yield the corresponding phosphonate products (**3o**–**3r**) in medium to excellent yields under the standard reaction conditions. However, 5-bromoindole does not undergo the reaction under these conditions. Interestingly, 5-bromoquinoline also reacts to yield product **3s** under standard conditions. Additionally, unprotected nitrogen heterocyclic substrates lead to products **3t** and **3u** with moderate to high yields. When naphthone and indenone are used as substrates, products **3v** and **3w** are obtained with yields of 81% and 89%, respectively. However, alkyl halides are not suitable for this reaction.

**Scheme 3:**
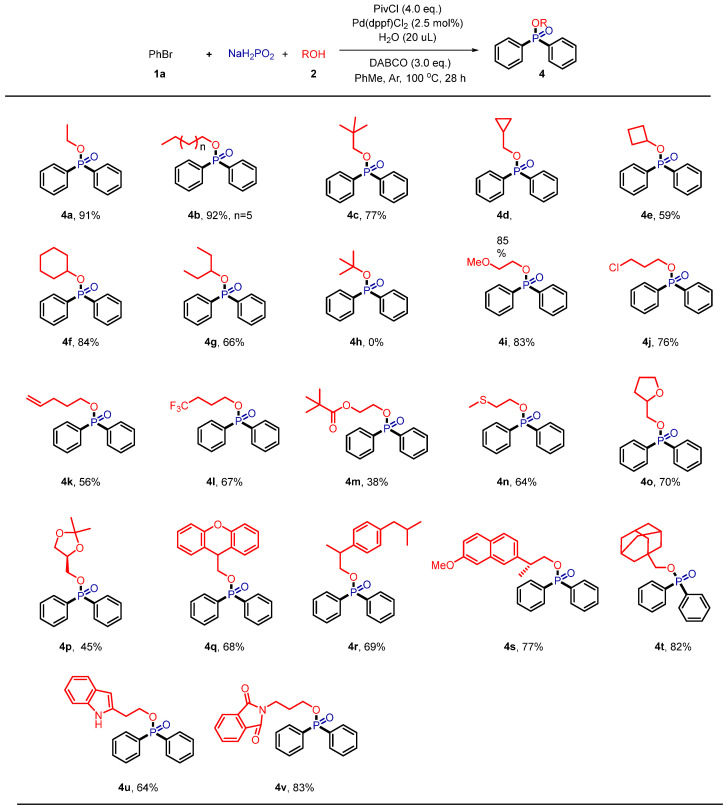
Expansion of alcohol substrates ^a^. ^a^ Bromobenzene **1a** (0.2 mmol), sodium hypophosphite (0.4 mmol), alcohol **2** (1.6 mmol), PivCl (0.8 mmol), Pd(dppf)Cl_2_ (2.5 mol%), DABCO (0.6 mmol), PhMe (2.0 mL) under Ar at 100 °C for 28 h.

After assessing the applicability of aryl halide substrates, we next investigated the scope of alcohol substrates (Scheme 3). The results showed that primary alcohols are highly compatible with this reaction. Short-chain ethanol, long-chain n-octanol, sterically hindered neopentyl alcohol, and cyclopropanol all participated effectively, yielding the corresponding diarylphosphinates (**4a**–**4d**) with excellent yields. The reaction also proceeds smoothly when secondary alcohols are used as substrates. Cyclobutanol, with high ring tension, and cyclohexanol, with low ring tension, both participate in the reaction, yielding products **4e** and **4f** in 59% and 84% yields, respectively. Increasing the steric hindrance of the secondary alcohol, 3-pentanol, leads to the target product **4g** with a medium to high yield. Tertiary alcohols with high steric hindrance are not suitable for this reaction, as no reaction occurs when tert-butanol is used as a substrate. We then investigated alcohols containing various functional groups, including methoxy, chlorine, terminal olefins, trifluoromethyl, and ester groups. We found that the functional groups in the alcohol molecules exhibit good compatibility in this reaction and are well-preserved, enabling further conversion of the products (**4i**–**4m**). Alcohols containing sulfur atoms, such as 2-methylthioethanol, were also identified as suitable substrates for this reaction (**4n**). When tetrahydrofurfuryl alcohol and chiral (R)-(-)-glyceroacetone aldehyde participate in the reaction, the corresponding target products (**4o**, **4p**) are obtained with moderate yields. It is gratifying that this reaction can introduce phosphorus atom into drug molecules, such as alcohols derived from xanthene-9-carboxylic acid, ibuprofen, and naproxen, yielding target products **4q**, **4r**, and **4s** with yields of 68%, 69%, and 77%, respectively. This product may have potential medicinal value. Surprisingly, alcohol substrates with complex structures also react smoothly, yielding product **4t**. Finally, we investigated the effect of nitrogen atoms on the reaction and found that the target product **4u**, with a yield of 64%, can still be obtained under standard conditions when chromol containing active nitrogen–hydrogen participates in the reaction. When *N*-(3-hydroxypropyl) phthalimide is used, the desired product **4v** is obtained with an 83% yield.

### 2.3. Gram-Scale Reaction of ***3a*** and Related Transformations

**Scheme 4:**
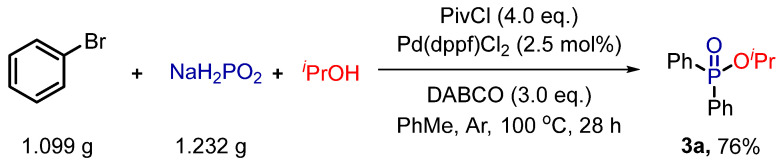
Gram-scale reaction of **3a**.

To demonstrate the practical applicability of this strategy in synthetic chemistry, a reaction on a 14.0 mmol scale was performed under standard conditions, yielding **3a** in 76% (Scheme 4).

### 2.4. Syntheses of Proposed Reaction Intermediates

**Scheme 5:**
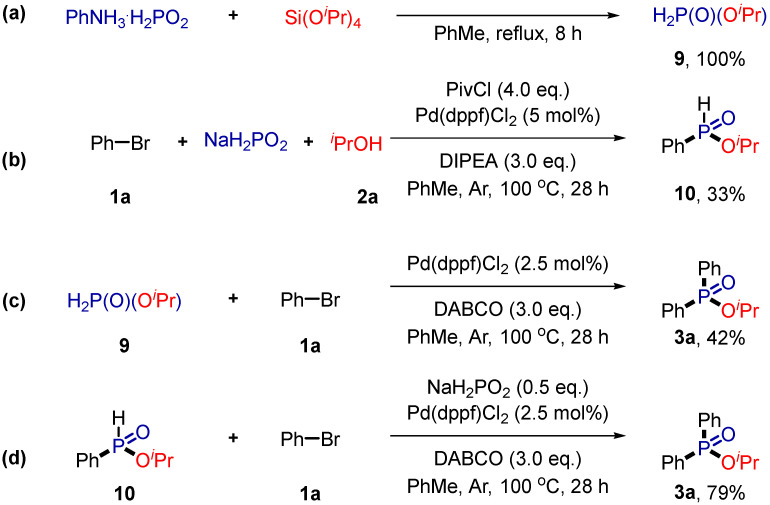
Controlled experiment.

Based on the reported literature and our understanding of the system [36,37,38], we believe that hypophosphite esters and aryl H-phosphonates are key intermediates in this reaction. To verify the validity of this hypothesis, several control experiments were conducted (Scheme 5). Initially, a toluene solution of isopropyl phosphinate was prepared by refluxing the aniline salt of hypophosphorous acid with isopropyl silicate in toluene under an argon atmosphere [39], which was subsequently used as a stock solution for further reactions. The nuclear magnetic resonance (NMR) conversion of isopropyl phosphinate **9** was determined to be 100% using triphenylphosphine as the internal standard (Scheme 5a). During the optimization of reaction conditions, it was also found that using diisopropylethylamine as a base, instead of triethylenediamine, yields isopropyl phenylphosphinate **10** (Scheme 5b). Subsequently, isopropyl phosphinate **9** and isopropyl phenylphosphinate **10** were used as substrates in coupling reactions with bromobenzene, yielding isopropyl diphenylphosphinate product **3a** in both cases (Scheme 5c,d). This result supports the hypothesis that hypophosphite esters and arylphosphonic acid esters are key intermediates in the reaction.

### 2.5. Possible Mechanisms

**Scheme 6:**
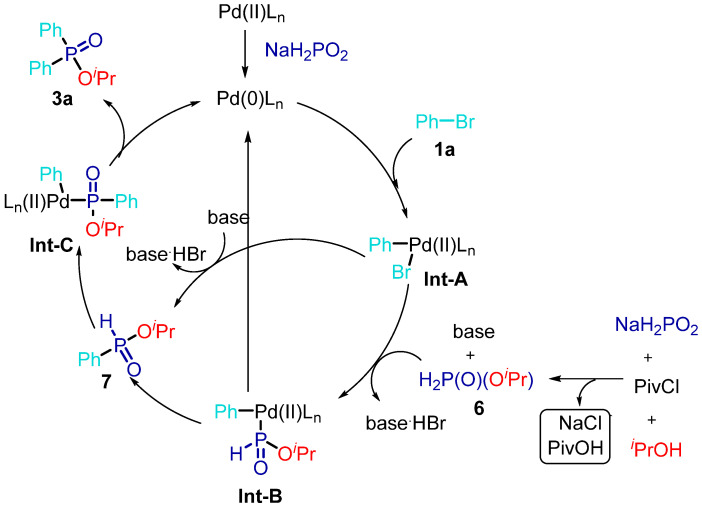
Possible mechanisms.

Based on our understanding of the coupling reaction catalyzed by transition metals, along with the results from controlled experiments, we propose a possible reaction mechanism (Scheme 6). First, sodium hypophosphite reduces the divalent palladium catalyst in situ to a zero-valent palladium catalyst, which undergoes oxidative addition with the aryl bromide substrate, forming the divalent palladium intermediate (**Int-A**). Next, isopropyl phosphinate **9**, generated in situ from sodium hypophosphite, trimethylacetyl chloride, and isopropanol, acts as a nucleophilic reagent and reacts with the divalent palladium intermediate (**Int-A**) in the presence of a base to form a new divalent palladium intermediate (**Int-B**). The divalent palladium intermediate (**Int-B**) then undergoes a reductive elimination reaction to generate aryl phosphonate **10** and release the zero-valent palladium catalyst. Next, aryl phosphonate **10** reacts with the divalent palladium intermediate (**Int-A**) in the presence of a base, forming another divalent palladium intermediate (**Int-C**). Finally, the divalent palladium intermediate (**Int-C**) undergoes reductive elimination to generate the isopropyl diphenylphosphinate **3a**, releasing the zero-valent palladium catalyst to continue the catalytic cycle.

## 3. Conclusions

In summary, we have developed a palladium-catalyzed multi-component reaction for the simultaneous construction of two carbon–phosphine bonds and one oxygen–phosphine bond, using stable, green, inexpensive, and abundant sodium hypophosphite as a phosphorus source. This method enables the synthesis of a series of diaryl phosphonates. The simple and efficient synthesis of diaryl phosphonates with high functional group tolerance demonstrates the potential of this approach for applications in biologically active molecules, catalytic ligands, and organophosphorus compounds.

## 4. Materials and Methods

General: ^1^H NMR, ^13^C NMR, ^31^P NMR, and ^19^F NMR spectra were recorded at room temperature using an Avance-400 instruments (Bruker, Billerica, MA, USA) (^1^H NMR at 400 MHz, ^13^C NMR at 101 MHz, ^31^P NMR at 162 MHz, and ^19^F NMR at 376 MHz). NMR spectra of all products were reported in ppm with reference to solvent signals [^1^H NMR: CD(H)Cl_3_ (7.26 ppm), ^13^C NMR: CD(H)Cl_3_ (77.00 ppm)]. Signal patterns are indicated as s, singlet; d, doublet; dd, doublets of doublet; t, triplet; and m, multiplet. The mass spectrometry was performed in the positive electrospray ionization (ESI+) mode. Reactions were monitored by thin-layer chromatography. Column chromatography (petroleum ether/ethyl acetate) was performed on silica gel (200–300 mesh). Analytical grade solvents and commercially available reagents were purchased from commercial sources and used directly without further purification unless otherwise stated.

Typical Procedure for Synthesis of Diarylphosphonates: To a 10 mL reaction tube, aryl halide (0.2 mmol, 1.0 eq.), sodium hypophosphite (0.4 mmol, 2.0 eq.), and Pd(dppf)Cl_2_ (0.005 mmol, 2.5 mol%) were added. The reaction tube was sealed with a rubber stopper, and the air was replaced with argon three times. Anhydrous THF (2 mL), alcohol (1.6 mmol, 8.0 eq.), and PivCl (0.8 mmol, 4.0 eq.) were then added by syringe. The mixture was stirred in a 100 °C oil bath for 28 h until the complete consumption of the starting material was confirmed by TLC analysis. After the reaction, the solution was filtered and washed with ethyl acetate. The combined filtrates were concentrated under reduced pressure, and the crude product was purified by silica-gel column chromatography (eluent: petroleum ether/ethyl acetate = 2/1–1/1), yielding the desired product.

*Isopropyl diphenylphosphinate* (**3a**) [40]. Colorless oil. Isolated yield: 83% (21.6 mg); (eluent: petroleum ether/ethyl acetate = 1/1); ^1^H NMR (400 MHz, CDCl_3_) δ 7.87–7.77 (m, 4H), 7.52–7.47 (m, 2H), 7.45–7.41 (m, 4H), 4.85–4.40 (m, 1H), 1.35 (d, *J* = 6.2 Hz, 6H). ^13^C NMR (101 MHz, CDCl_3_) δ 133.13 (s), 131.93 (d, *J* = 2.8 Hz), 131.77 (s), 131.62 (d, *J* = 10.0 Hz), 128.41 (d, *J* = 13.0 Hz), 70.20 (d, *J* = 6.0 Hz), 24.32 (d, *J* = 4.2 Hz). ^31^P NMR (162 MHz, CDCl_3_) δ 29.73 (s). HRMS (ESI): [M + H]^+^ calcd for C_15_H_18_O_2_P^+^ 261.1039; found 261.1037.

*Isopropyl di-o-tolylphosphinate* (**3b**). Colorless oil. Isolated yield: 42% (12.1 mg); (eluent: petroleum ether/ethyl acetate = 1/1); ^1^H NMR (400 MHz, CDCl_3_) δ 8.09–7.85 (m, 2H), 7.39 (t, *J* = 7.5 Hz, 2H), 7.33–7.23 (m, 2H), 7.20–7.12 (m, 2H), 4.82–4.56 (m, 1H), 2.33 (s, 6H), 1.33 (d, *J* = 6.2 Hz, 6H). ^13^C NMR (101 MHz, CDCl_3_) δ 141.42 (d, *J* = 11.3 Hz), 133.46 (d, *J* = 9.7 Hz), 132.04 (d, *J* = 2.7 Hz), 131.30 (d, *J* = 12.6 Hz), 130.16 (s), 125.41 (d, *J* = 12.6 Hz), 69.85 (d, *J* = 5.8 Hz), 24.18 (d, *J* = 4.1 Hz), 21.18 (d, *J* = 4.3 Hz). ^31^P NMR (162 MHz, CDCl_3_) δ 29.82 (s). HRMS (ESI): [M + H]^+^ calcd for C_17_H_22_O_2_P^+^ 289.1352; found 289.1353.

*Isopropyl di-m-tolylphosphinate* (**3c**) [41]. Colorless oil. Isolated yield: 80% (23.0 mg); (eluent: petroleum ether/ethyl acetate = 1/1); ^1^H NMR (400 MHz, CDCl_3_) δ 7.66 (d, *J* = 12.6 Hz, 2H), 7.61–7.56 (m, 2H), 7.41–7.24 (m, 4H), 4.69–4.61 (m, 1H), 2.37 (s, 6H), 1.35 (d, *J* = 6.2 Hz, 6H). ^13^C NMR (101 MHz, CDCl_3_) δ 138.22 (d, *J* = 13.0 Hz), 132.89 (s), 132.73 (d, *J* = 2.9 Hz), 132.13 (d, *J* = 10.1 Hz), 128.66 (d, *J* = 10.0 Hz), 128.29 (d, *J* = 13.8 Hz), 70.15 (d, *J* = 6.0 Hz), 24.33 (d, *J* = 4.2 Hz), 21.38 (s). ^31^P NMR (162 MHz, CDCl_3_) δ 30.62 (s). HRMS (ESI): [M + H]^+^ calcd for C_17_H_22_O_2_P^+^ 289.1352; found 289.1343.

*Isopropyl di-p-tolylphosphinate* (**3d**) [40]. Colorless oil. Isolated yield: 87% (25.0 mg); (eluent: petroleum ether/ethyl acetate = 1/1); ^1^H NMR (400 MHz, CDCl_3_) δ 7.69 (dd, *J* = 12.0, 8.0 Hz, 4H), 7.32–7.15 (m, 4H), 4.68–4.60 (m, 1H), 2.37 (s, 6H), 1.33 (d, *J* = 6.2 Hz, 6H). ^13^C NMR (101 MHz, CDCl_3_) δ 142.29 (d, *J* = 2.9 Hz), 131.62 (d, *J* = 10.4 Hz), 130.07 (s), 129.12 (d, *J* = 13.5 Hz), 128.68 (s), 69.90 (d, *J* = 6.0 Hz), 24.33 (d, *J* = 4.1 Hz), 21.59 (d, *J* = 1.0 Hz). ^31^P NMR (162 MHz, CDCl_3_) δ 30.77 (s). HRMS (ESI): [M + H]^+^ calcd for C_17_H_22_O_2_P^+^ 289.1352; found 289.1352.

*Isopropyl bis(4-methoxyphenyl)phosphinate* (**3e**) [42]. Colorless oil. Isolated yield: 80% (25.6 mg); (eluent: petroleum ether/ethyl acetate = 1/1); ^1^H NMR (400 MHz, CDCl_3_) δ 7.73 (dd, *J* = 11.5, 8.7 Hz, 4H), 6.94 (d, *J* = 6.7 Hz, 4H), 4.62 (td, *J* = 12.3, 6.2 Hz, 1H), 3.83 (s, 6H), 1.32 (d, *J* = 6.1 Hz, 6H). ^13^C NMR (101 MHz, CDCl_3_) δ 162.36 (d, *J* = 2.6 Hz), 133.47 (d, *J* = 11.3 Hz), 130.95 (s), 128.88–128.83 (m), 124.83 (s), 123.40 (s), 113.90 (d, *J* = 14.0 Hz), 69.68 (d, *J* = 5.6 Hz), 55.31 (s), 24.35 (d, *J* = 3.9 Hz). ^31^P NMR (162 MHz, CDCl_3_) δ 30.61 (s). HRMS (ESI): [M + H]^+^ calcd for C_17_H_22_O_4_P^+^ 321.1250; found 321.1249.

*Isopropyl bis(4-(trifluoromethyl)phenyl)phosphinate* (**3f**) [40]. Colorless oil. Isolated yield: 65% (25.7 mg); (eluent: petroleum ether/ethyl acetate = 1/1); ^1^H NMR (400 MHz, CDCl_3_) δ 7.96 (dd, *J* = 11.9, 8.0 Hz, 4H), 7.73 (dd, *J* = 8.1, 2.6 Hz, 4H), 4.81–4.66 (m, 1H), 1.39 (d, *J* = 6.2 Hz, 6H). ^13^C NMR (101 MHz, CDCl_3_) δ 135.95 (d, *J* = 135.7 Hz), 134.93–133.20 (m), 132.11 (d, *J* = 10.4 Hz), 126.01–125.08 (m), 123.47 (d, *J* = 272.6 Hz). ^31^P NMR (162 MHz, CDCl_3_) δ 25.96 (s). ^19^F NMR (376 MHz, CDCl_3_) δ -63.25 (d, *J* = 26.9 Hz). HRMS (ESI): [M + H]^+^ calcd for C_17_H_16_F_6_O_2_P^+^ 397.0787; found 397.0793.

*Isopropyl di([1,1’-biphenyl]-4-yl)phosphinate* (**3g**). Colorless oil. Isolated yield: 73% (30.1 mg); (eluent: petroleum ether/ethyl acetate = 1/1); ^1^H NMR (400 MHz, CDCl_3_) δ 7.95–7.90 (m, 4H), 7.67 (dd, *J* = 8.2, 3.1 Hz, 4H), 7.62–7.54 (m, 4H), 7.46–7.42 (m, 4H), 7.41–7.33 (m, 2H), 4.91–4.55 (m, 1H), 1.40 (d, *J* = 6.2 Hz, 6H). ^13^C NMR (101 MHz, CDCl_3_) δ 144.80 (d, *J* = 2.9 Hz), 140.04 (s), 132.18 (d, *J* = 10.4 Hz), 131.75 (s), 130.37 (s), 128.95 (s), 128.12 (s), 127.28 (d, *J* = 2.2 Hz), 127.14 (s), 70.41 (d, *J* = 6.0 Hz), 24.43 (d, *J* = 4.2 Hz). ^31^P NMR (162 MHz, CDCl_3_) δ 29.93 (s). HRMS (ESI): [M + H]^+^ calcd for C_27_H_26_O_2_P^+^ 413.1665; found 413.1660.

*Isopropyl bis(4-ethyl formatephenyl)phosphinate* (**3h**) [41]. Colorless oil. Isolated yield: 73% (29.5 mg); (eluent: petroleum ether/ethyl acetate = 1/1); ^1^H NMR (400 MHz, CDCl_3_) δ 8.11 (dd, *J* = 8.1, 3.1 Hz, 4H), 7.90 (dd, *J* = 11.9, 8.2 Hz, 4H), 4.83–4.62 (m, 1H), 3.93 (s, 6H), 1.37 (d, *J* = 6.2 Hz, 6H). ^13^C NMR (101 MHz, CDCl_3_) δ 166.16 (s), 137.32 (s), 135.98 (s), 133.43 (d, *J* = 2.9 Hz), 131.66 (d, *J* = 10.4 Hz), 129.52 (d, *J* = 13.2 Hz), 71.20 (d, *J* = 6.0 Hz), 52.46 (s), 24.27 (d, *J* = 4.2 Hz). ^31^P NMR (162 MHz, CDCl_3_) δ 27.23 (s). HRMS (ESI): [M + H]^+^ calcd for C_21_H_26_O_6_P^+^ 405.1462; found 405.1466.

*Isopropyl bis(4-acetylphenyl)phosphinate* (**3i**) [41]. Colorless oil. Isolated yield: 81% (27.9 mg); (eluent: petroleum ether/ethyl acetate = 1/1); ^1^H NMR (400 MHz, CDCl_3_) δ 8.09–7.97 (m, 4H), 7.93 (dd, *J* = 11.6, 8.0 Hz, 4H), 4.88–4.51 (m, 1H), 2.63 (s, 6H), 1.38 (d, *J* = 6.2 Hz, 6H). ^13^C NMR (101 MHz, CDCl_3_) δ 197.42 (s), 139.72 (d, *J* = 2.8 Hz), 137.37 (s), 136.03 (s), 131.93 (d, *J* = 10.3 Hz), 128.16 (d, *J* = 13.2 Hz), 71.30 (d, *J* = 6.1 Hz), 26.79 (s), 24.28 (d, *J* = 4.2 Hz). ^31^P NMR (162 MHz, CDCl_3_) δ 27.00 (s). HRMS (ESI): [M + H]^+^ calcd for C_19_H_22_O_4_P^+^ 345.1250; found 345.1246.

*Isopropyl bis(4-cyanophenyl)phosphinate* (**3j**). Colorless oil. Isolated yield: 84% (26.0 mg); (eluent: petroleum ether/ethyl acetate = 1/1); ^1^H NMR (400 MHz, CDCl_3_) δ 7.95–7.90 (m, 4H), 7.84–7.68 (m, 4H), 4.84–4.64 (m, 1H), 1.39 (d, *J* = 6.2 Hz, 6H). ^13^C NMR (101 MHz, CDCl_3_) δ 137.34 (s), 135.99 (s), 132.34 (s), 132.20 (d, *J* = 0.8 Hz), 132.10 (s), 117.66 (d, *J* = 1.3 Hz), 116.24 (d, *J* = 3.2 Hz), 72.15 (d, *J* = 6.0 Hz), 24.26 (d, *J* = 4.2 Hz). ^31^P NMR (162 MHz, CDCl_3_) δ 24.54 (s). HRMS (ESI): [M + H]^+^ calcd for C_17_H_16_N_2_O_2_P^+^ 311.0944; found 311.0944.

*Isopropyl bis(4-formylphenyl)phosphinate* (**3k**). Colorless oil. Isolated yield: 63% (19.9 mg); (eluent: petroleum ether/ethyl acetate = 1/1); ^1^H NMR (400 MHz, CDCl_3_) δ 10.08 (s, 2H), 8.16–7.79 (m, 8H), 4.76 (qd, *J* = 12.4, 6.2 Hz, 1H), 1.39 (d, *J* = 6.2 Hz, 6H). ^13^C NMR (101 MHz, CDCl_3_) δ 191.58 (s), 138.70 (d, *J* = 2.6 Hz), 137.38 (s), 132.30 (d, *J* = 10.3 Hz), 129.56 (d, *J* = 13.3 Hz), 71.62 (d, *J* = 6.1 Hz), 24.33 (d, *J* = 4.2 Hz). ^31^P NMR (162 MHz, CDCl_3_) δ 26.27 (s). HRMS (ESI): [M + H]^+^ calcd for C_17_H_18_O_4_P^+^ 317.0937; found 317.0943.

*Isopropyl bis(4-vinylphenyl)phosphinate* (**3l**). Colorless oil. Isolated yield: 58% (18.1 mg); (eluent: petroleum ether/ethyl acetate = 1/1); ^1^H NMR (400 MHz, CDCl_3_) δ 7.76 (dt, *J* = 18.5, 9.3 Hz, 4H), 7.46 (dd, *J* = 8.1, 2.9 Hz, 4H), 6.72 (dd, *J* = 17.6, 10.9 Hz, 2H), 5.83 (d, *J* = 17.6 Hz, 2H), 5.36 (d, *J* = 10.9 Hz, 2H), 4.67 (qd, *J* = 12.3, 6.1 Hz, 1H), 1.35 (d, *J* = 6.1 Hz, 6H). ^13^C NMR (101 MHz, CDCl_3_) δ 141.01 (d, *J* = 2.9 Hz), 136.01 (s), 131.93 (d, *J* = 10.4 Hz), 126.18 (d, *J* = 13.5 Hz), 116.40 (s), 70.27 (d, *J* = 5.9 Hz), 24.35 (d, *J* = 4.1 Hz). ^31^P NMR (162 MHz, CDCl_3_) δ 29.63 (s). HRMS (ESI): [M + H]^+^ calcd for C_19_H_22_O_2_P^+^ 313.1352; found 313.1350.

*Isopropyl di(naphthalen-1-yl)phosphinate* (**3m**). White solid. Isolated yield: 80% (28.8 mg); (eluent: petroleum ether/ethyl acetate = 1/1); ^1^H NMR (400 MHz, CDCl_3_) δ 8.64–8.52 (m, 2H), 8.22–8.16 (m, 2H), 8.00 (d, *J* = 8.2 Hz, 2H), 7.89–7.80 (m, 2H), 7.54–7.42 (m, 6H), 4.90–4.77 (m, 1H), 1.34 (d, *J* = 6.2 Hz, 6H). ^13^C NMR (101 MHz, CDCl_3_) δ 134.05 (d, *J* = 10.2 Hz), 133.69 (d, *J* = 10.7 Hz), 133.41 (d, *J* = 3.1 Hz), 132.98 (d, *J* = 10.5 Hz), 129.27 (s), 128.85 (d, *J* = 1.5 Hz), 127.15 (s), 126.81 (d, *J* = 4.9 Hz), 126.25 (s), 124.65 (d, *J* = 14.8 Hz), 70.95 (d, *J* = 6.0 Hz), 24.33 (d, *J* = 4.2 Hz). ^31^P NMR (162 MHz, CDCl_3_) δ 32.00 (s). HRMS (ESI): [M + H]^+^ calcd for C_23_H_22_O_2_P^+^ 361.1352; found 361.1349.

*Isopropyl bis(1,2-dihydroacenaphthylen-5-yl)phosphinate* (**3n**). White solid. Isolated yield: 71% (29.3 mg); (eluent: petroleum ether/ethyl acetate = 1/1); ^1^H NMR (400 MHz, CDCl_3_) δ 8.14 (dd, *J* = 16.1, 7.9 Hz, 4H), 7.42 (dd, *J* = 8.3, 7.1 Hz, 2H), 7.35–7.21 (m, 4H), 4.82–4.74 (m, 1H), 3.36 (s, 8H), 1.34 (d, *J* = 6.2 Hz, 6H). ^13^C NMR (101 MHz, CDCl_3_) δ 152.00 (d, *J* = 3.0 Hz), 146.44 (d, *J* = 1.8 Hz), 139.27 (d, *J* = 11.9 Hz), 135.66 (d, *J* = 11.4 Hz), 131.41 (d, *J* = 11.0 Hz), 128.93 (s), 124.69 (s), 123.33 (s), 122.27 (d, *J* = 3.5 Hz), 119.93 (s), 118.52 (d, *J* = 14.8 Hz), 70.33 (d, *J* = 5.9 Hz), 30.32 (d, *J* = 12.9 Hz), 24.44 (d, *J* = 4.2 Hz). ^31^P NMR (162 MHz, CDCl_3_) δ 29.36 (s). HRMS (ESI): [M + H]^+^ calcd for C_27_H_26_O_2_P^+^ 413.1665; found 413.1652.

*Isopropyl bis(1,2-dihydroacenaphthylen-5-yl)phosphinate* (**3o**). White solid. Isolated yield: 79% (21.5 mg); (eluent: petroleum ether/ethyl acetate = 1/1); ^1^H NMR (400 MHz, CDCl_3_) δ 7.70–7.68 (m, 2H), 7.65 (dd, *J* = 7.8, 3.6 Hz, 2H), 7.19–7.12 (m, 2H), 4.82–4.53 (m, 1H), 1.38 (d, *J* = 6.2 Hz, 6H). ^13^C NMR (101 MHz, CDCl_3_) δ 136.26 (d, *J* = 12.1 Hz), 133.93 (s), 133.53 (d, *J* = 6.4 Hz), 132.31 (s), 128.16 (d, *J* = 16.5 Hz), 71.41 (d, *J* = 5.9 Hz), 24.29 (d, *J* = 4.3 Hz). ^31^P NMR (162 MHz, CDCl_3_) δ 16.86 (s). HRMS (ESI): [M + H]^+^ calcd for C_11_H_14_O_2_PS_2_^+^ 273.0167; found 273.0164.

*Isopropyl bis(benzo[b]thiophen-5-yl)phosphinate* (**3p**). Colorless oil. Isolated yield: 80% (29.8 mg); (eluent: petroleum ether/ethyl acetate = 1/1); ^1^H NMR (400 MHz, CDCl_3_) δ 8.38 (d, *J* = 13.3 Hz, 2H), 7.93 (dd, *J* = 8.3, 2.9 Hz, 2H), 7.76–7.71 (m, 2H), 7.49 (d, *J* = 5.5 Hz, 2H), 7.39 (d, *J* = 5.5 Hz, 2H), 4.78–4.69 (m, 1H), 1.39 (d, *J* = 6.2 Hz, 6H). ^13^C NMR (101 MHz, CDCl_3_) δ 143.28 (d, *J* = 2.9 Hz), 139.24 (d, *J* = 14.9 Hz), 128.97 (s), 127.87 (d, *J* = 10.7 Hz), 127.68 (s), 126.16 (d, *J* = 11.6 Hz), 124.21 (s), 122.73 (d, *J* = 14.2 Hz), 70.39 (d, *J* = 5.9 Hz), 24.42 (d, *J* = 4.1 Hz). ^31^P NMR (162 MHz, CDCl_3_) δ 31.23 (s). HRMS (ESI): [M + H]^+^ calcd for C_19_H_18_O_2_PS_2_^+^ 373.0480; found 373.0474.

*Isopropyl di(benzofuran-5-yl)phosphinate* (**3q**). Colorless oil. Isolated yield: 89% (30.3 mg); (eluent: petroleum ether/ethyl acetate = 1/1); ^1^H NMR (400 MHz, CDCl_3_) δ 8.16 (dd, *J* = 12.6, 0.8 Hz, 2H), 7.78–7.73 (m, 2H), 7.67 (d, *J* = 2.2 Hz, 2H), 7.56 (dd, *J* = 8.5, 2.4 Hz, 2H), 6.84–6.74 (m, 2H), 4.84–4.55 (m, 1H), 1.37 (d, *J* = 6.2 Hz, 6H). ^13^C NMR (101 MHz, CDCl_3_) δ 156.76 (d, *J* = 3.1 Hz), 146.11 (s), 127.68 (d, *J* = 7.3 Hz), 127.54 (d, *J* = 12.2 Hz), 126.32 (s), 125.92 (d, *J* = 11.6 Hz), 111.70 (d, *J* = 14.7 Hz), 106.86 (d, *J* = 1.0 Hz), 70.15 (d, *J* = 5.9 Hz), 24.38 (d, *J* = 4.2 Hz). ^31^P NMR (162 MHz, CDCl_3_) δ 31.47 (s). HRMS (ESI): [M + H]^+^ calcd for C_19_H_18_O_4_P^+^ 341.0937; found 341.0932

*Isopropyl bis(benzo[d]*[*1,3*]*dioxol-5-yl)phosphinate* (**3r**). Colorless oil. Isolated yield: 60% (20.9 mg); (eluent: petroleum ether/ethyl acetate = 1/1); ^1^H NMR (400 MHz, CDCl_3_) δ 7.35 (dd, *J* = 12.9, 7.9 Hz, 1H), 7.19 (d, *J* = 11.8 Hz, 1H), 6.86 (dd, *J* = 7.9, 2.7 Hz, 1H), 6.00 (s, 1H), 4.76–4.53 (m, 1H), 1.34 (d, *J* = 6.2 Hz, 1H). ^13^C NMR (101 MHz, CDCl_3_) δ 150.76 (d, *J* = 3.1 Hz), 147.80 (d, *J* = 19.8 Hz), 127.02 (d, *J* = 11.1 Hz), 125.71 (d, *J* = 143.5 Hz), 111.01 (d, *J* = 12.6 Hz), 108.59 (d, *J* = 16.4 Hz), 101.52 (s), 70.15 (d, *J* = 5.9 Hz), 24.31 (d, *J* = 4.1 Hz). ^31^P NMR (162 MHz, CDCl_3_) δ 29.58 (s). HRMS (ESI): [M + H]^+^ calcd for C_17_H_18_O_6_P^+^ 349.0836; found 349.0828.

*Isopropyl di(quinolin-5-yl)phosphinate* (**3s**). White solid. Isolated yield: 67% (24.3 mg); (eluent: petroleum ether/ethyl acetate = 1/1); ^1^H NMR (400 MHz, CDCl_3_) δ 8.96 (dd, *J* = 12.8, 5.8 Hz, 4H), 8.31 (d, *J* = 8.5 Hz, 2H), 8.16 (dd, *J* = 15.2, 7.1 Hz, 2H), 7.77 (td, *J* = 7.9, 3.3 Hz, 2H), 7.43 (dd, *J* = 8.6, 4.2 Hz, 2H), 4.88 (qd, *J* = 12.4, 6.2 Hz, 1H), 1.37 (d, *J* = 6.2 Hz, 6H). ^13^C NMR (101 MHz, CDCl_3_) δ 150.86 (s), 148.26 (d, *J* = 10.7 Hz), 135.06 (d, *J* = 3.0 Hz), 134.71 (d, *J* = 4.7 Hz), 134.02 (d, *J* = 9.8 Hz), 129.90 (d, *J* = 211.1 Hz), 129.79 (s), 128.59–128.38 (m), 128.28 (d, *J* = 15.6 Hz), 122.03 (s), 71.61 (d, *J* = 6.0 Hz), 24.35 (d, *J* = 4.2 Hz). ^31^P NMR (162 MHz, CDCl_3_) δ 29.37 (s). HRMS (ESI): [M + H]^+^ calcd for C_21_H_20_N_2_O_2_P^+^ 363.1257; found 363.1252.

*Isopropyl di(indolin-4-yl)phosphinate* (**3t**). Colorless oil. Isolated yield: 74% (25.3 mg); (eluent: petroleum ether/ethyl acetate = 1/1); ^1^H NMR (400 MHz, CDCl_3_) δ 8.26 (dd, *J* = 8.3, 2.7 Hz, 2H), 7.73–7.52 (m, 4H), 4.62 (qd, *J* = 12.3, 6.1 Hz, 1H), 4.26 (t, *J* = 8.3 Hz, 4H), 3.15 (t, *J* = 8.2 Hz, 4H), 1.33 (d, *J* = 6.2 Hz, 6H). ^13^C NMR (101 MHz, CDCl_3_) δ 177.11 (s), 147.91 (d, *J* = 3.0 Hz), 131.64 (d, *J* = 11.0 Hz), 131.12 (d, *J* = 14.3 Hz), 127.57 (d, *J* = 11.2 Hz), 127.08 (d, *J* = 141.4 Hz),117.88 (d, *J* = 13.8 Hz), 69.86 (d, *J* = 5.9 Hz), 49.70 (s), 40.39 (s), 28.98 (s), 24.37 (d, *J* = 4.1 Hz). ^31^P NMR (162 MHz, CDCl_3_) δ 30.50 (s). HRMS (ESI): [M + H]^+^ calcd for C_19_H_24_N_2_O_2_P^+^ 343.1570; found 343.1572.

*Isopropyl di(9H-carbazol-1-yl)phosphinate* (**3u**). White solid. Isolated yield: 73% (32.0 mg); (eluent: petroleum ether/ethyl acetate = 1/1); ^1^H NMR (400 MHz, CDCl_3_) δ 10.26 (s, 2H), 8.21 (d, *J* = 7.7 Hz, 2H), 8.06 (d, *J* = 7.8 Hz, 2H), 7.72 (dd, *J* = 13.1, 7.5 Hz, 2H), 7.51 (d, *J* = 8.1 Hz, 2H), 7.45 (t, *J* = 7.3 Hz, 2H), 7.24 (dd, *J* = 9.6, 5.0 Hz, 4H), 4.74 (ddt, *J* = 12.3, 8.9, 6.2 Hz, 1H), 1.42 (d, *J* = 6.1 Hz,6H). ^13^C NMR (101 MHz, CDCl_3_) δ 141.97 (d, *J* = 6.1 Hz), 139.73 (s), 128.73 (d, *J* = 8.3 Hz), 126.65 (s), 124.63 (d, *J* = 2.6 Hz), 124.26 (d, *J* = 9.5 Hz), 122.27 (s), 120.41 (s), 119.77 (s), 118.83 (d, *J* = 12.5 Hz), 111.64 (d, *J* = 142.4 Hz), 111.24 (s), 71.40 (d, *J* = 6.1 Hz), 24.43 (d, *J* = 4.2 Hz). ^31^P NMR (162 MHz, CDCl_3_) δ 35.30 (s). HRMS (ESI): [M + H]^+^ calcd for C_27_H_24_N_2_O_2_P^+^ 439.1570; found 439.1567.

*Isopropyl bis(8-oxo-5,6,7,8-tetrahydronaphthalen-2-yl)phosphinate* (**3v**). White solid. Isolated yield: 81% (32.1 mg); (eluent: petroleum ether/ethyl acetate = 1/1); ^1^H NMR (400 MHz, CDCl_3_) δ 8.38 (dd, *J* = 12.8, 1.0 Hz, 2H), 8.08–7.85 (m, 2H), 7.36 (dd, *J* = 7.8, 2.9 Hz, 2H), 4.68 (qd, *J* = 12.3, 6.2 Hz, 1H), 3.00 (t, *J* = 5.9 Hz, 4H), 2.83–2.44 (m, 4H), 2.28–1.94 (m, 4H), 1.36 (d, *J* = 6.2 Hz, 6H). ^13^C NMR (101 MHz, CDCl_3_) δ 197.24 (s), 148.35 (d, *J* = 2.6 Hz), 135.94 (d, *J* = 10.5 Hz), 135.01 (d, *J* = 140.4Hz), 132.55 (d, *J* = 12.5 Hz), 130.66 (d, *J* = 11.4 Hz), 129.36 (d, *J* = 12.5 Hz), 70.81 (d, *J* = 6.0 Hz), 39.04 (s), 29.82 (s), 24.35 (d, *J* = 4.1 Hz), 22.79 (s). ^31^P NMR (162 MHz, CDCl_3_) δ 27.82 (s). HRMS (ESI): [M + H]^+^ calcd for C_23_H_26_O_4_P^+^ 397.1563; found 397.1561.

*Isopropyl bis(1-oxo-2,3-dihydro-1H-inden-4-yl)phosphinate* (**3w**). Colorless oil. Isolated yield: 89% (32.8 mg); (eluent: petroleum ether/ethyl acetate = 1/1); ^1^H NMR (400 MHz, CDCl_3_) δ 8.18 (dd, *J* = 12.4, 7.4 Hz, 2H), 7.95 (d, *J* = 7.6 Hz, 2H), 7.56 (dd, *J* = 7.3, 6.0 Hz, 2H), 4.85 (dq, *J* = 12.5, 6.2 Hz, 1H), 3.22 (dt, *J* = 17.9, 5.7 Hz, 2H), 3.08 (dt, *J* = 11.5, 5.6 Hz, 2H), 2.64 (t, *J* = 5.9 Hz, 4H), 1.41 (d, *J* = 6.1 Hz, 6H). ^13^C NMR (101 MHz, CDCl_3_) δ 205.78 (s), 157.64 (d, *J* = 11.6 Hz), 138.41 (d, *J* = 9.8 Hz), 137.99 (d, *J* = 11.1 Hz), 129.95 (d, *J* = 135.7 Hz), 127.95 (d, *J* = 2.7 Hz), 127.59 (d, *J* = 11.6 Hz), 71.30 (d, *J* = 5.9 Hz), 35.86 (s), 26.12 (d, *J* = 2.2 Hz), 24.37 (d, *J* = 4.1 Hz). ^31^P NMR (162 MHz, CDCl_3_) δ 24.53 (s). HRMS (ESI): [M + H]^+^ calcd for C_21_H_22_O_4_P^+^ 369.1250; found 369.1250.

*Ethyl diphenylphosphinate* (**4a**) [40]. Colorless oil. Isolated yield: 91% (22.4 mg); (eluent: petroleum ether/ethyl acetate = 1/1); ^1^H NMR (400 MHz, CDCl_3_) δ 7.88–7.77 (m, 4H), 7.55–7.48 (m, 2H), 7.48–7.41 (m, 4H), 4.11 (p, *J* = 7.1 Hz, 2H), 1.37 (t, *J* = 7.0 Hz, 3H). ^13^C NMR (101 MHz, CDCl_3_) δ 132.11 (d, *J* = 2.7 Hz), 131.68 (d, *J* = 137.4 Hz), 131.63 (d, *J* = 10.1 Hz), 128.52 (d, *J* = 13.1 Hz), 61.12 (d, *J* = 5.9 Hz), 16.52 (d, *J* = 6.7 Hz). ^31^P NMR (162 MHz, CDCl_3_) δ 31.33 (s). HRMS (ESI): [M + H]^+^ calcd for C_14_H_16_O_2_P^+^ 247.0882; found 247.0892.

*Octyl diphenylphosphinate* (**4b**) [42]. White solid. Isolated yield: 92% (30.4 mg); (eluent: petroleum ether/ethyl acetate = 1/1); ^1^H NMR (400 MHz, CDCl_3_) δ 7.89–7.77 (m, 4H), 7.55–7.47 (m, 2H), 7.44 (dt, *J* = 8.5, 4.5 Hz, 4H), 4.03 (q, *J* = 6.6 Hz, 2H), 1.79–1.66 (m, 2H), 1.38 (d, *J* = 6.6 Hz, 2H), 1.26 (d, *J* = 3.3 Hz, 8H), 0.87 (t, *J* = 6.5 Hz, 3H). **^1^**^3^C NMR (101 MHz, CDCl_3_) δ 132.08 (d, *J* = 2.7 Hz), 131.63 (d, *J* = 10.1 Hz), 131.60 (d, *J* = 138.3 Hz), 128.50 (d, *J* = 13.1 Hz), 65.06 (d, *J* = 6.1 Hz), 31.75 (s), 30.52 (d, *J* = 6.6 Hz), 29.12 (d, *J* = 5.1 Hz), 27.27 (s), 25.60 (s), 22.62 (s), 14.10 (s). ^31^P NMR (162 MHz, CDCl_3_) δ 31.22 (s). HRMS (ESI): [M + H]^+^ calcd for C_20_H_28_O_2_P^+^ 331.1821; found 331.1821.

*Neopentyl diphenylphosphinate* (**4c**) [42]. White solid. Isolated yield: 77% (22.2 mg); (eluent: petroleum ether/ethyl acetate = 1/1); ^1^H NMR (400 MHz, CDCl_3_) δ 7.82 (dd, *J* = 12.2, 7.2 Hz, 4H), 7.56–7.49 (m, 2H), 7.45 (dt, *J* = 10.2, 5.2 Hz, 4H), 3.67 (d, *J* = 4.8 Hz, 2H), 0.99 (s, 9H). ^13^C NMR (101 MHz, CDCl_3_) δ 132.07 (d, *J* = 2.8 Hz), 131.69 (d, *J* = 137.4 Hz), 131.66 (d, *J* = 10.0 Hz), 128.52 (d, *J* = 13.1 Hz), 73.84 (d, *J* = 6.5 Hz), 32.26 (d, *J* = 7.4 Hz), 26.28 (s). ^31^P NMR (162 MHz, CDCl_3_) δ 30.62 (s). HRMS (ESI): [M + H]^+^ calcd for C_17_H_22_O_2_P^+^ 289.1352; found 289.1351.

*Cyclopropylmethyl diphenylphosphinate* (**4d**). Colorless oil. Isolated yield: 85% (23.1 mg); (eluent: petroleum ether/ethyl acetate = 1/1); ^1^H NMR (400 MHz, CDCl_3_) δ 7.90–7.77 (m, 4H), 7.51 (dd, *J* = 10.5, 4.2 Hz, 2H), 7.48–7.37 (m, 4H), 3.89 (t, *J* = 7.4 Hz, 2H), 1.18 (ddd, *J* = 10.2, 7.8, 4.0 Hz, 1H), 0.54 (q, *J* = 5.2 Hz, 2H), 0.26 (q, *J* = 5.1 Hz, 2H). ^13^C NMR (101 MHz, CDCl_3_) δ 132.21 (d, *J* = 2.8 Hz), 131.72 (d, *J* = 10.2 Hz), 131.46 (d, *J* = 137.4Hz), 128.53 (d, *J* = 13.2 Hz), 71.73 (d, *J* = 6.7 Hz), 63.74 (d, *J* = 5.9 Hz), 58.95 (s). ^31^P NMR (162 MHz, CDCl_3_) δ 31.09 (s). HRMS (ESI): [M + H]^+^ calcd for C_16_H_18_O_2_P^+^ 273.1039; found 273.0996.

*Cyclobutyl diphenylphosphinate* (**4e**). Colorless oil. Isolated yield: 59% (16.0 mg); (eluent: petroleum ether/ethyl acetate = 1/1); ^1^H NMR (400 MHz, CDCl_3_) δ 7.87–7.74 (m, 4H), 7.55–7.47 (m, 2H), 7.48–7.35 (m, 4H), 4.91–4.67 (m, 1H), 2.23 (dd, *J* = 17.1, 8.6 Hz, 4H), 1.81–1.63 (m, 1H), 1.53–1.36 (m, 1H). ^13^C NMR (101 MHz, CDCl_3_) δ 132.06 (d, *J* = 2.8 Hz), 131.96 (d, *J* = 137.4 Hz), 131.63 (d, *J* = 10.2 Hz), 128.45 (d, *J* = 13.1 Hz), 69.17 (d, *J* = 7.5 Hz), 32.35 (d, *J* = 4.5 Hz), 12.86 (s). ^31^P NMR (162 MHz, CDCl_3_) δ 29.67 (s). HRMS (ESI): [M + H]^+^ calcd for C_16_H_18_O_2_P^+^ 273.1039, found 273.1034.

*Cyclohexyl diphenylphosphinate* (**4f**) [40]. Colorless oil. Isolated yield: 84% (25.2 mg); (eluent: petroleum ether/ethyl acetate = 1/1); ^1^H NMR (400 MHz, CDCl_3_) δ 7.88–7.74 (m, 4H), 7.54–7.47 (m, 2H), 7.43 (ddd, *J* = 7.1, 5.5, 2.7 Hz, 4H), 4.53–4.34 (m, 1H), 1.99–1.82 (m, 2H), 1.74 (dd, *J* = 10.1, 4.8 Hz, 2H), 1.60 (dt, *J* = 12.6, 6.3 Hz, 2H), 1.47 (dd, *J* = 9.5, 4.1 Hz, 1H), 1.37–1.25 (m, 3H). ^13^C NMR (101 MHz, CDCl_3_) δ 133.26 (s), 131.92 (d, *J* = 2.8 Hz), 131.62 (d, *J* = 10.1 Hz), 128.41 (d, *J* = 13.1 Hz), 74.97 (d, *J* = 6.2 Hz), 33.95 (d, *J* = 3.7 Hz), 25.21 (s), 23.60 (s). ^31^P NMR (162 MHz, CDCl_3_) δ 29.78 (s). HRMS (ESI): [M + H]^+^ calcd for C_18_H_22_O_2_P^+^ 301.1352; found 301.1350.

*Pentan-3-yl diphenylphosphinate* (**4g**). Colorless oil. Isolated yield: 66% (19.0 mg); (eluent: petroleum ether/ethyl acetate = 1/1); ^1^H NMR (400 MHz, CDCl_3_) δ 7.89–7.75 (m, 4H), 7.55–7.47 (m, 2H), 7.43 (td, *J* = 7.3, 3.4 Hz, 4H), 4.49–4.29 (m, 1H), 1.79–1.59 (m, 4H), 0.88 (t, *J* = 7.4 Hz, 6H). ^13^C NMR (101 MHz, CDCl_3_) δ 132.69 (d, *J* = 137.4 Hz), 131.87 (d, *J* = 2.7 Hz), 131.64 (d, *J* = 10.1 Hz), 128.36 (d, *J* = 13.1 Hz), 79.28 (d, *J* = 6.6 Hz), 27.28 (d, *J* = 3.8 Hz), 9.13 (s). ^31^P NMR (162 MHz, CDCl_3_) δ 29.45 (s). HRMS (ESI): [M + H]^+^ calcd for C_17_H_22_O_2_P^+^ 289.1352; found 289.1349.

*2-Methoxyethyl diphenylphosphinate* (**4i**). Colorless oil. Isolated yield: 83% (22.9 mg); (eluent: petroleum ether/ethyl acetate = 1/1); ^1^H NMR (400 MHz, CDCl_3_) δ 7.91–7.78 (m, 4H), 7.51 (dd, *J* = 10.5, 4.0 Hz, 2H), 7.45 (td, *J* = 7.3, 3.4 Hz, 4H), 4.18 (td, *J* = 7.5, 4.8 Hz, 2H), 3.79–3.56 (m, 2H), 3.36 (s, 3H). ^13^C NMR (101 MHz, CDCl_3_) δ 132.07 (d, *J* = 2.8 Hz), 131.83 (d, *J* = 137.4 Hz), 131.67 (d, *J* = 10.1 Hz), 128.49 (d, *J* = 13.1 Hz), 69.87 (d, *J* = 5.8 Hz), 11.48 (d, *J* = 7.2 Hz), 3.58 (s). ^31^P NMR (162 MHz, CDCl_3_) δ 32.41 (s). HRMS (ESI): [M + H]^+^ calcd for C_15_H_18_O_3_P^+^ 277.0988; found 277.0990.

*3-Chloropropyl diphenylphosphinate* (**4j**). Colorless oil. Isolated yield: 76% (22.3 mg); (eluent: petroleum ether/ethyl acetate = 1/1); ^1^H NMR (400 MHz, CDCl_3_) δ 7.88–7.75 (m, 4H), 7.57–7.50 (m, 2H), 7.46 (ddd, *J* = 7.1, 5.6, 2.8 Hz, 4H), 4.19 (dd, *J* = 12.9, 5.9 Hz, 2H), 3.71 (t, *J* = 6.3 Hz, 2H), 2.23–2.13 (m, 2H). ^13^C NMR (101 MHz, CDCl_3_) δ 132.32 (d, *J* = 2.8 Hz), 131.63 (d, *J* = 10.1 Hz), 131.20 (d, *J* = 137.4 Hz), 128.64 (d, *J* = 13.2 Hz), 61.52 (d, *J* = 5.7 Hz), 40.98 (s), 33.38 (d, *J* = 6.5 Hz). ^31^P NMR (162 MHz, CDCl_3_) δ 32.07 (s). HRMS (ESI): [M + H]^+^ calcd for C_15_H_17_ClO_2_P^+^ 295.0649; found 295.0652.

*Pent-4-en-1-yl diphenylphosphinate* (**4k**). Colorless oil. Isolated yield: 56% (16.0 mg); (eluent: petroleum ether/ethyl acetate = 1/1); ^1^H NMR (400 MHz, CDCl_3_) δ 7.81 (dd, *J* = 11.4, 7.7 Hz, 4H), 7.58–7.49 (m, 2H), 7.45 (dt, *J* = 10.2, 5.1 Hz, 4H), 5.67–5.47 (m, 1H), 5.48–5.28 (m, 1H), 4.02 (qd, *J* = 6.9, 3.1 Hz, 2H), 2.45 (dq, *J* = 31.7, 6.8 Hz, 2H), 1.65 (d, *J* = 6.2 Hz, 2H). ^13^C NMR (101 MHz, CDCl_3_) δ 132.11 (d, *J* = 2.6 Hz), 131.68 (d, *J* = 10.1 Hz), 128.51 (d, *J* = 13.1 Hz), 128.35 (s), 125.99 (s), 64.57 (d, *J* = 6.1 Hz), 33.93 (d, *J* = 6.6 Hz), 18.05 (s). ^31^P NMR (162 MHz, CDCl_3_) δ 31.24 (s). HRMS (ESI): [M + H]^+^ calcd for C_17_H_20_O_2_P^+^ 287.1195; found 287.1200.

*4,4,4-Trifluorobutyl diphenylphosphinate* (**4l**). Colorless oil. Isolated yield: 67% (22.0 mg); (eluent: petroleum ether/ethyl acetate = 1/1); ^1^H NMR (400 MHz, CDCl_3_) δ 7.88–7.73 (m, 4H), 7.55 (t, *J* = 7.4 Hz, 2H), 7.47 (td, *J* = 7.3, 3.5 Hz, 4H), 4.09 (dd, *J* = 12.7, 6.3 Hz, 2H), 2.34–2.21 (m, 2H), 1.99 (dt, *J* = 16.1, 6.1 Hz, 2H). ^13^C NMR (101 MHz, CDCl_3_) δ 132.38 (d, *J* = 2.8 Hz), 131.58 (d, *J* = 10.2 Hz), 131.12 (d, *J* = 137.4 Hz), 128.67 (d, *J* = 13.2 Hz), 63.09 (d, *J* = 5.7 Hz), 30.49 (q, *J* = 29.3 Hz), 23.44 (dd, *J* = 6.6, 3.1 Hz). ^31^P NMR (162 MHz, CDCl_3_) δ 32.18 (s). ^19^F NMR (376 MHz, CDCl_3_) δ -66.29 (s). HRMS (ESI): [M + H]^+^ calcd for C_16_H_17_F_3_O_2_P^+^ 329.0913; found 329.0908.

*2-*((*Diphenylphosphoryl)oxy)ethyl pivalate* (**4m**). Colorless oil. Isolated yield: 38% (13.1 mg); (eluent: petroleum ether/ethyl acetate = 1/1); ^1^H NMR (400 MHz, CDCl_3_) δ 7.88–7.74 (m, 4H), 7.57–7.50 (m, 2H), 7.46 (td, *J* = 7.4, 3.5 Hz, 4H), 4.38–4.30 (m, 2H), 4.26–4.19 (m, 2H), 1.21 (s, 9H). ^13^C NMR (101 MHz, CDCl_3_) δ 178.30 (s), 132.34 (d, *J* = 2.7 Hz), 131.63 (d, *J* = 10.2 Hz), 131.15 (d, *J* = 137.4Hz), 128.61 (d, *J* = 13.2 Hz), 63.17 (d, *J* = 7.7 Hz), 62.51 (d, *J* = 5.6 Hz), 38.78 (s), 27.13 (d, *J* = 6.4 Hz). ^31^P NMR (162 MHz, CDCl_3_) δ 32.39 (s). HRMS (ESI): [M + H]^+^ calcd for C_19_H_24_O_4_P^+^ 347.1407; found 347.1403.

*2-(Methylthio)ethyl diphenylphosphinate* (**4n**). Colorless oil. Isolated yield: 64% (18.7 mg); (eluent: petroleum ether/ethyl acetate = 1/1); ^1^H NMR (400 MHz, CDCl_3_) δ 7.84 (dd, *J* = 11.9, 7.6 Hz, 4H), 7.58–7.50 (m, 2H), 7.47 (dd, *J* = 7.1, 3.0 Hz, 4H), 4.17 (dd, *J* = 14.2, 7.1 Hz, 2H), 2.83 (t, *J* = 6.8 Hz, 2H), 2.10 (s, 3H). ^13^C NMR (101 MHz, CDCl_3_) δ 132.30 (d, *J* = 2.8 Hz), 131.68 (d, *J* = 10.2 Hz), 131.26 (d, *J* = 137.4 Hz), 128.61 (d, *J* = 13.2 Hz), 63.38 (d, *J* = 5.8 Hz), 34.38 (d, *J* = 6.8 Hz), 15.83 (s). ^31^P NMR (162 MHz, CDCl_3_) δ 32.04 (s). HRMS (ESI): [M + H]^+^ calcd for C_15_H_18_O_2_PS^+^ 293.0760; found 293.0757.

(*Tetrahydrofuran-2-yl)methyl diphenylphosphinate* (**4o**). Colorless oil. Isolated yield: 70% (21.1 mg); (eluent: petroleum ether/ethyl acetate = 1/1); ^1^H NMR (400 MHz, CDCl_3_) δ 7.92–7.73 (m, 4H), 7.51 (d, *J* = 6.8 Hz, 2H), 7.45 (s, 4H), 4.25–4.14 (m, 1H), 4.04 (d, *J* = 4.8 Hz, 1H), 4.00–3.91 (m, 2H), 3.90–3.76 (m, 1H), 2.03–1.95 (m, 1H), 1.93–1.84 (m, 1H), 1.76–1.67 (m, 1H). ^13^C NMR (101 MHz, CDCl_3_) δ 132.21 (s), 131.75 (dd, *J* = 10.0, 5.8 Hz), 128.55 (d, *J* = 13.0 Hz), 68.56 (s), 66.55 (d, *J* = 5.9 Hz), 27.84 (s), 25.73 (s). ^31^P NMR (162 MHz, CDCl_3_) δ 32.21 (s). HRMS (ESI): [M + H]^+^ calcd for C_17_H_20_O_3_P^+^ 303.1145; found 303.1144.

(*R)-(2,2-Dimethyl-1,3-dioxolan-4-yl)methyl diphenylphosphinate* (**4p**). Colorless oil. Isolated yield: 45% (19.4 mg); (eluent: petroleum ether/ethyl acetate = 1/1); ^1^H NMR (400 MHz, CDCl_3_) δ 7.83 (dd, *J* = 10.8, 8.7 Hz, 4H), 7.58–7.50 (m, 2H), 7.47 (dd, *J* = 7.3, 3.1 Hz, 4H), 4.44–4.35 (m, 1H), 4.13–4.07 (m, 1H), 4.07–3.98 (m, 2H), 3.89 (dd, *J* = 8.4, 5.6 Hz, 1H), 1.38 (d, *J* = 17.0 Hz, 6H). ^13^C NMR (101 MHz, CDCl_3_) δ 132.37 (d, *J* = 2.8 Hz), 131.75 (d, *J* = 4.0 Hz), 131.65 (d, *J* = 4.0 Hz), 128.62 (d, *J* = 13.2 Hz), 109.83 (s), 74.46 (d, *J* = 7.5 Hz), 66.24 (s), 64.85 (d, *J* = 5.9 Hz), 26.71 (s), 25.27 (s). ^31^P NMR (162 MHz, CDCl_3_) δ 32.76 (s). HRMS (ESI): [M + H]^+^ calcd for C_18_H_22_O_4_P^+^ 333.1250; found 333.1251.

(*9H-Xanthen-9-yl)methyl diphenylphosphinate* (**4q**). White solid. Isolated yield: 68% (28.0 mg); (eluent: petroleum ether/ethyl acetate = 1/1); ^1^H NMR (400 MHz, CDCl_3_) δ 7.60–7.40 (m, 6H), 7.33 (ddd, *J* = 6.9, 5.4, 2.6 Hz, 4H), 7.30–7.20 (m, 4H), 7.15–7.02 (m, 4H), 4.33 (t, *J* = 5.7 Hz, 1H), 4.07 (t, *J* = 5.5 Hz, 2H). ^13^C NMR (101 MHz, CDCl_3_) δ 152.49 (s), 132.05 (d, *J* = 2.8 Hz), 131.53 (d, *J* = 10.2 Hz), 131.04 (d, *J* = 137.4 Hz), 129.43 (s), 128.54 (s), 128.41 (s), 123.31 (s), 121.17 (s), 116.50 (s), 69.59 (d, *J* = 6.2 Hz), 40.26 (d, *J* = 8.1 Hz). ^31^P NMR (162 MHz, CDCl_3_) δ 31.81 (s). HRMS (ESI): [M + H]^+^ calcd for C_26_H_22_O_3_P^+^ 413.1301; found 413.1302.

*2-(4-Isobutylphenyl)propyl diphenylphosphinate* (**4r**). White solid. Isolated yield: 69% (27.0 mg); (eluent: petroleum ether/ethyl acetate = 1/1); ^1^H NMR (400 MHz, CDCl_3_) δ 7.75–7.59 (m, 4H), 7.49 (t, *J* = 7.3 Hz, 2H), 7.44–7.33 (m, 4H), 7.10 (q, *J* = 8.2 Hz, 4H), 4.16–3.88 (m, 2H), 3.25–3.06 (m, 1H), 2.46 (d, *J* = 7.2 Hz, 2H), 1.95–1.78 (m, 1H), 1.35 (d, *J* = 7.0 Hz, 3H), 0.90 (d, *J* = 6.6 Hz, 6H). ^13^C NMR (101 MHz, CDCl_3_) δ 140.08 (d, *J* = 16.2 Hz), 132.05 (d, *J* = 2.6 Hz), 131.65 (t, *J* = 9.8 Hz), 129.17 (s), 128.46 (d, *J* = 13.1 Hz), 127.26 (s), 69.75 (d, *J* = 6.2 Hz), 45.05 (s), 40.15 (d, *J* = 7.3 Hz), 30.29 (s), 22.40 (d, *J* = 2.5 Hz), 17.59 (s). ^31^P NMR (162 MHz, CDCl_3_) δ 31.03 (s). HRMS (ESI): [M + H]^+^ calcd for C_25_H_30_O_2_P^+^ 393.1978; found 393.1984.

(*S)-2-(7-Methoxynaphthalen-2-yl)propyl diphenylphosphinate* (**4s**). White solid. Isolated yield: 77% (32.0 mg); (eluent: petroleum ether/ethyl acetate = 1/1); ^1^H NMR (400 MHz, CDCl_3_) δ 7.71–7.63 (m, 4H), 7.59 (dd, *J* = 11.0, 6.3 Hz, 3H), 7.44 (dd, *J* = 15.1, 7.6 Hz, 2H), 7.32 (ddd, *J* = 18.8, 9.2, 3.4 Hz, 5H), 7.17–7.10 (m, 2H), 4.22–4.09 (m, 2H), 3.91 (s, 3H), 3.40–3.25 (m, 1H), 1.42 (d, *J* = 7.0 Hz, 3H). ^13^C NMR (101 MHz, CDCl_3_) δ 157.47 (s), 138.00 (s), 133.59 (s), 132.05 (s), 131.73 (s), 131.62 (s), 131.52 (s), 130.72 (d, *J* = 26.9 Hz), 129.17 (s), 129.00 (s), 128.45 (dd, *J* = 13.1, 2.2 Hz), 126.93 (s), 126.56 (s), 125.90 (s), 118.90 (s), 105.56 (s), 69.68 (d, *J* = 6.2 Hz), 55.33 (s), 40.47 (d, *J* = 7.2 Hz), 17.66 (s). ^31^P NMR (162 MHz, CDCl_3_) δ 31.26 (s). HRMS (ESI): [M + H]^+^ calcd for C_26_H_26_O_3_P^+^ 417.1614; found 417.1612.

*Adamantan-1-ylmethyl diphenylphosphinate* (**4t**). White solid. Isolated yield: 82% (30.0 mg); (eluent: petroleum ether/ethyl acetate = 1/1); ^1^H NMR (400 MHz, CDCl_3_) δ 7.82 (dd, *J* = 12.0, 7.2 Hz, 4H), 7.56–7.48 (m, 2H), 7.45 (dt, *J* = 10.1, 5.1 Hz, 4H), 3.57 (d, *J* = 4.9 Hz, 2H), 2.00 (s, 3H), 1.73 (d, *J* = 12.1 Hz, 3H), 1.65 (d, *J* = 12.0 Hz, 3H), 1.59 (s, 6H). ^13^C NMR (101 MHz, CDCl_3_) δ 132.05 (d, *J* = 2.8 Hz), 131.71 (d, *J* = 10.0 Hz), 131.71 (d, *J* = 138.4 Hz), 128.52 (d, *J* = 13.1 Hz), 74.16 (d, *J* = 6.5 Hz), 39.09 (s), 36.96 (s), 34.01 (d, *J* = 7.4 Hz), 28.03 (s). ^31^P NMR (162 MHz, CDCl_3_) δ 30.79 (s). HRMS (ESI): [M + H]^+^ calcd for C_23_H_28_O_2_P^+^ 367.1821; found 367.1816.

*2-(1H-indol-2-yl)ethyl diphenylphosphinate* (**4u**). Colorless oil. Isolated yield: 64% (23.1 mg); (eluent: petroleum ether/ethyl acetate = 1/1); ^1^H NMR (400 MHz, CDCl_3_) δ 8.42 (s, 1H), 7.81–7.69 (m, 4H), 7.53–7.44 (m, 3H), 7.44–7.35 (m, 4H), 7.30 (d, *J* = 34.1 Hz, 1H), 7.17 (t, *J* = 7.5 Hz, 1H), 7.06 (t, *J* = 7.4 Hz, 1H), 7.01 (s, 1H), 4.28 (q, *J* = 7.1 Hz, 2H), 3.20 (t, *J* = 7.1 Hz, 2H). ^13^C NMR (101 MHz, CDCl_3_) δ 136.20 (s), 132.13 (d, *J* = 2.6 Hz), 131.67 (d, *J* = 10.1 Hz), 130.75 (s), 128.52 (d, *J* = 13.1 Hz), 127.43 (s), 122.23 (d, *J* = 46.2 Hz), 119.02 (d, *J* = 63.8 Hz), 111.23 (s), 65.02 (d, *J* = 6.2 Hz), 26.82 (d, *J* = 6.9 Hz). ^31^P NMR (162 MHz, CDCl_3_) δ 31.70 (s). HRMS (ESI): [M + H]^+^ calcd for C_22_H_21_NO_2_P^+^ 362.1304; found 362.1294.

*3-(1,3-Dioxoisoindolin-2-yl)propyl diphenylphosphinate* (**4v**). White solid. Isolated yield: 83% (33.6 mg); (eluent: petroleum ether/ethyl acetate = 1/1); ^1^H NMR (400 MHz, CDCl_3_) δ 7.89–7.73 (m, 6H), 7.69 (dd, *J* = 5.3, 3.1 Hz, 2H), 7.54–7.46 (m, 2H), 7.43 (td, *J* = 7.3, 3.5 Hz, 4H), 4.10 (q, *J* = 6.2 Hz, 2H), 3.88 (t, *J* = 6.9 Hz, 2H), 2.13 (p, *J* = 6.4 Hz, 2H). **^1^**^3^C NMR (101 MHz, CDCl_3_) δ 168.22 (s), 133.99 (s), 132.18 (d, *J* = 2.8 Hz), 132.01 (s), 131.64 (d, *J* = 10.2 Hz), 131.22 (d, *J* = 137.4 Hz), 128.55 (d, *J* = 13.2 Hz), 123.22 (s), 62.21 (d, *J* = 5.7 Hz), 34.93 (s), 29.47 (d, *J* = 6.7 Hz). ^31^P NMR (162 MHz, CDCl_3_) δ 31.87 (s). HRMS (ESI): [M + H]^+^ calcd for C_23_H_21_NO_4_P^+^ 406.1203; found 406.1204.

## Figures and Tables

**Table 1:** Reaction condition optimization ^a^.

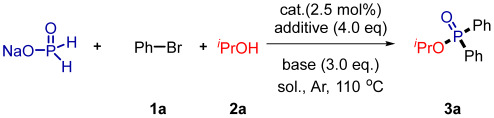					
Entry	Additive	Base	Cat.	Sol.	Result/% ^b^
1	none	DABCO	Pd(dppf)Cl_2_	PhMe	N.R. ^g^
2	BzCl	DABCO	Pd(dppf)Cl_2_	PhMe	N.R. ^g^
3	TsCl	DABCO	Pd(dppf)Cl_2_	PhMe	N.R. ^g^
4	PivCl	DABCO	Pd(dppf)Cl_2_	PhMe	83
5	PivCl	NaHCO_3_	Pd(dppf)Cl_2_	PhMe	36
6	PivCl	NaOAc	Pd(dppf)Cl_2_	PhMe	trace
7	PivCl	DBU ^f^	Pd(dppf)Cl_2_	PhMe	67
8	PivCl	Et_3_N ^f^	Pd(dppf)Cl_2_	PhMe	53
9	PivCl	DIPEA ^f^	Pd(dppf)Cl_2_	PhMe	60
10 ^c^	PivCl	DABCO	Pd(dppf)Cl_2_	PhMe	74
11 ^d^	PivCl	DABCO	Pd(dppf)Cl_2_	PhMe	80
12	PivCl	DABCO	Pd(dppe)Cl_2_ ^e^	PhMe	64
13	PivCl	DABCO	Pd(PPh_3_)_2_Cl_2_ ^e^	PhMe	16
14	PivCl	DABCO	Ni(dppf)Cl_2_	PhMe	N.R. ^g^
15	PivCl	DABCO	Co(dppf)Cl_2_	PhMe	N.R. ^g^
16	PivCl	DABCO	Pd(dppf)Cl_2_	THF	51
17	PivCl	DABCO	Pd(dppf)Cl_2_	EtOAc	63
18	PivCl	DABCO	Pd(dppf)Cl_2_	DCE	trace

^a^ Bromobenzene (0.2 mmol), NaH_2_PO_2_ (0.4 mmol), isopropyl alcohol (1.6 mmol), additive (0.8 mmol), catalyst (2.5 mol%), DABCO (0.6 mmol), solvent (2.0 mL) under Ar at 100 °C for 28 h; ^b^ Isolated yield; ^c^ 1.0 mL solvent; ^d^ 3.0 mL solvent; ^e^ dppe = 1,2-Bis (diphenylphosphino) ethane; dppf = 1,1’-Bis (diphenylphosphino) ferrocene; PPh_3_ = triphenylphosphine; ^f^ DBU = 1,8-Diazabicyclo[5.4.0] undec-7-ene; Et_3_N = triethylamine; DIPEA = N,N-Diisopropylethylamine; DABCO = 1,4-Diazabicyclo[2.2.2] octane; ^g^ N.R.= No Reaction.

## Data Availability

Data are contained within the article and Supplementary Materials.

## Supplementary Materials

The following supporting information can be downloaded at https://www.mdpi.com/article/10.3390/molecules30071564/s1: Table S1: Reaction condition optimization; Derivatization reaction of **3a**; Characterization data of all products; and Scanned ^1^H NMR, ^13^C NMR, ^31^P NMR, and ^19^F NMR spectra of all products.
