# Supplementary material for: Pd-Catalyzed Direct Diarylation of Sodium Hypophosphite Enables the Synthesis of Diarylphosphonates
Source: Molecules. 2025 Mar 31;30(7):1564. doi: 10.3390/molecules30071564 (PMC11990813; doi:10.3390/molecules30071564)

Pd-catalyzed Direct Diarylation of Sodium Hypophosphite Enables the Synthesis of Diarylphosphinates

JinYang,[a] Dang-Wei Qian,[a] Gang-Wei Wang,[a] Shang-Dong Yang*[a,b]

[a]*State Key Laboratory of Applied Organic Chemistry, Lanzhou University Lanzhou 730000, P. R. China.*
[b]*State Key Laboratory for Oxo Synthesis and Selective Oxidation, Lanzhou Institute of Chemical Physics, Chinese Academy of Sciences, Lanzhou 730000 (P.R. China).*

Corresponding author email: yangshd@lzu.edu.cn.

Table of Contents

1. Screening of Optimal Reaction Conditions 2

2. Scanned ^{1}H NMR, ^{13}C NMR, ^{31}P NMR and ^{19}F NMR Spectra of All products..4

1. Screening of Optimal Reaction Conditions

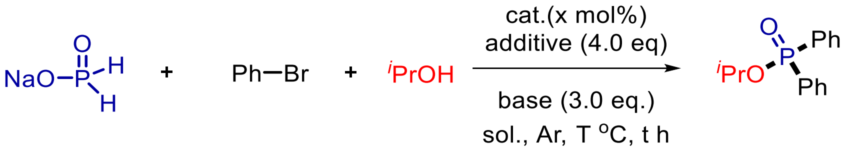

1a 2a 3a

entry	additive	base	T/ °C	t/h	cat.	sol.	yield/%[b]
1	BzCl	DABCO	100	20	$Pd(dppf)Cl_2$/5 mol%	PhMe	n.r
2	CyC(O)Cl	DABCO	100	20	$Pd(dppf)Cl_2$/5 mol%	PhMe	19
3	TsCl	DABCO	100	20	$Pd(dppf)Cl_2$/5 mol%	PhMe	n.r
4	AcCl	DABCO	100	20	$Pd(dppf)Cl_2$/5 mol%	PhMe	38%
5	PivCl	DABCO	100	20	$Pd(dppf)Cl_2$/5 mol%	PhMe	48%
6	PivCl	DABCO	60	20	$Pd(dppf)Cl_2$/5 mol%	PhMe	n.r
7	PivCl	DABCO	80	20	$Pd(dppf)Cl_2$/5 mol%	PhMe	23
8	PivCl	DABCO	120	20	$Pd(dppf)Cl_2$/5 mol%	PhMe	48
9	PivCl	DABCO	100	12	$Pd(dppf)Cl_2$/5 mol%	PhMe	23
10	PivCl	DABCO	100	24	$Pd(dppf)Cl_2$/5 mol%	PhMe	58
11	PivCl	DABCO	100	28	$Pd(dppf)Cl_2$/5 mol%	PhMe	68
12	PivCl	DABCO	100	36	$Pd(dppf)Cl_2$/5 mol%	PhMe	61
13	PivCl	DABCO	100	28	$Pd(dppf)Cl_2$/1 mol%	PhMe	trace
14	PivCl	DABCO	100	28	$Pd(dppf)Cl_2$/2.5 mol%	PhMe	84
15	PivCl	DABCO	100	28	$Pd(dppf)Cl_2$/10 mol%	PhMe	45
16	PivCl	$NaHCO_3$	100	28	$Pd(dppf)Cl_2$/2.5 mol%	PhMe	36
17	PivCl	$KHCO_3$	100	28	$Pd(dppf)Cl_2$/2.5 mol%	PhMe	47
18	PivCl	NaOAc	100	28	$Pd(dppf)Cl_2$/2.5 mol%	PhMe	trace
19	PivCl	KOAc	100	28	$Pd(dppf)Cl_2$/2.5 mol%	PhMe	44
20	PivCl	DBU	100	28	$Pd(dppf)Cl_2$/2.5 mol%	PhMe	67
21	PivCl	Et_3N	100	28	$Pd(dppf)Cl_2$/2.5 mol%	PhMe	53
22	PivCl	DIPEA	100	28	$Pd(dppf)Cl_2$/2.5 mol%	PhMe	60
23[c]	PivCl	DABCO	100	28	$Pd(dppf)Cl_2$/2.5 mol%	PhMe	74

24[d]	PivCl	DABCO	100	28	Pd(dppf)Cl_2/2.5 mol%	PhMe	80
25[e]	PivCl	DABCO	100	28	Pd(dppf)Cl_2/2.5 mol%	PhMe	71
26	PivCl	DABCO	100	28	Pd(dppe)Cl_2/2.5 mol%	PhMe	64
27	PivCl	DABCO	100	28	Pd(PPh_3)$_2$$Cl_2$/2.5 mol%	PhMe	16
28	PivCl	DABCO	100	28	Ni(dppf)Cl_2/2.5 mol%	PhMe	n.r
29	PivCl	DABCO	100	28	Co(dppf)Cl_2/2.5 mol%	PhMe	n.r
30	PivCl	DABCO	100	28	Pd(dppf)Cl_2/2.5 mol%	xylene	74
31	PivCl	DABCO	100	28	Pd(dppf)Cl_2/2.5 mol%	dioxane	trace
32	PivCl	DABCO	100	28	Pd(dppf)Cl_2/2.5 mol%	THF	58
33	PivCl	DABCO	100	28	Pd(dppf)Cl_2/2.5 mol%	EtOAc	68
34	PivCl	DABCO	100	28	Pd(dppf)Cl_2/2.5 mol%	DCE	trace

Table S1(a) Reaction conditions: bromobenzene (0.2 mmol), NaH_2PO_2 (0.4 mmol), ^{i}PrOH (1.6 mmol), PivCl (0.8 mmol), anhydrous solvent (2.0 mL); (b) Isolated yield; (c) 1.0 mL solvent; (d) 3.0 mL solvent; (e) 4.0 mL solvent.

2. Scanned ^{1}H NMR, ^{13}C NMR, ^{31}P NMR and ^{19}F NMR Spectra of All products

^{1}H NMR (400 MHz, $CDCl_3$) spectrum for 3a

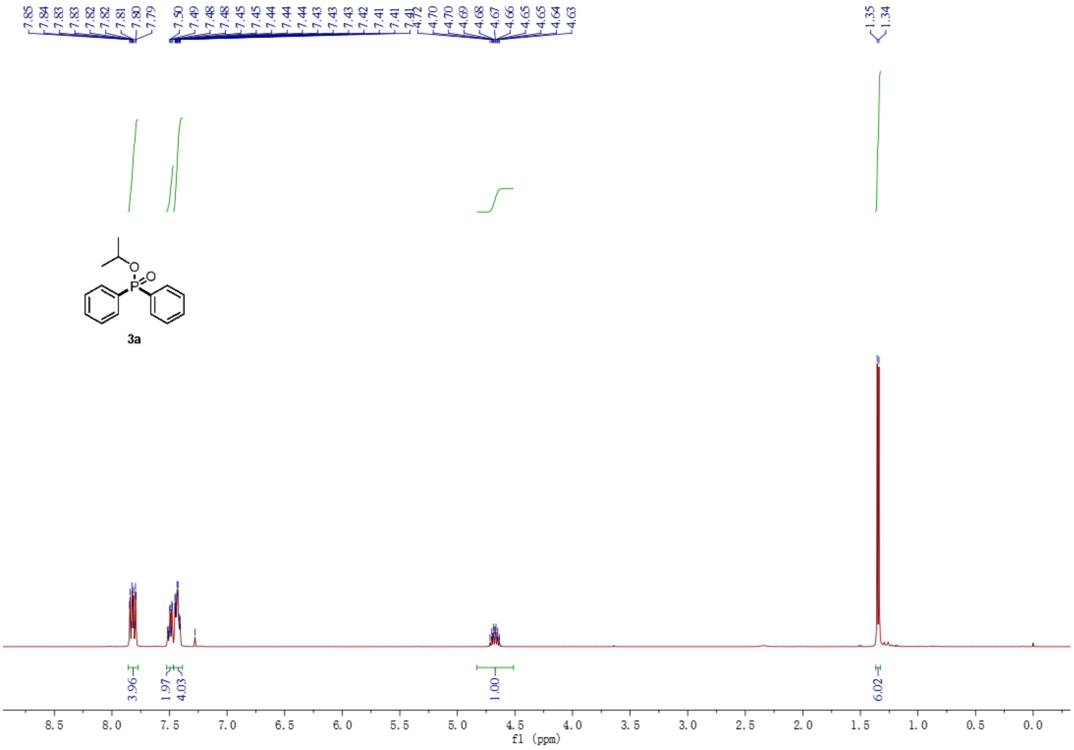

^{13}C NMR (101 MHz, $CDCl_3$) spectrum for 3a

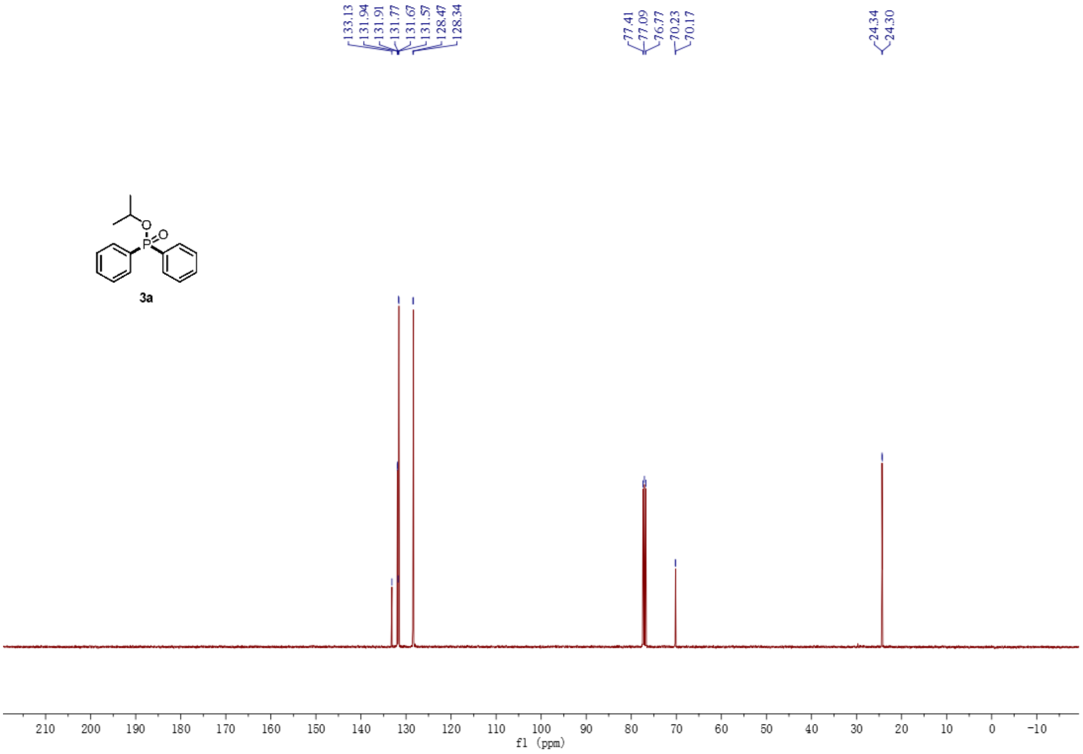

^{31}P NMR (121 MHz, CDCl$_3$) spectrum for 3a

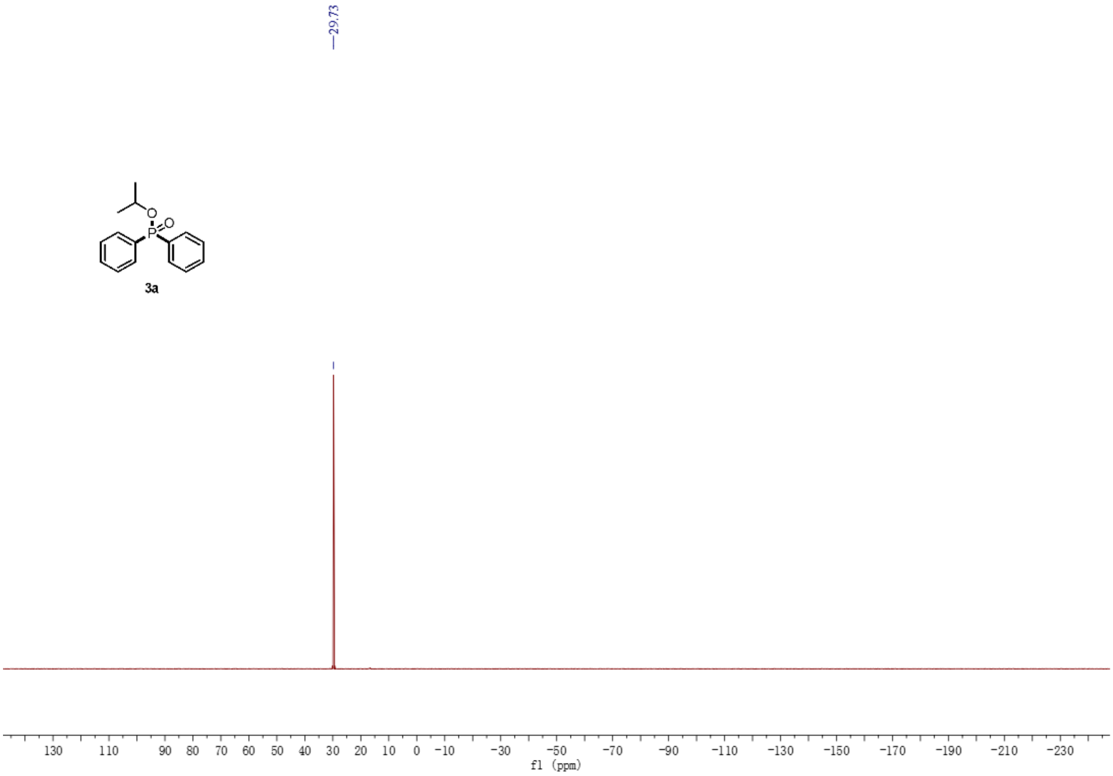

^{1}H NMR (400 MHz, CDCl$_3$) spectrum for 3b

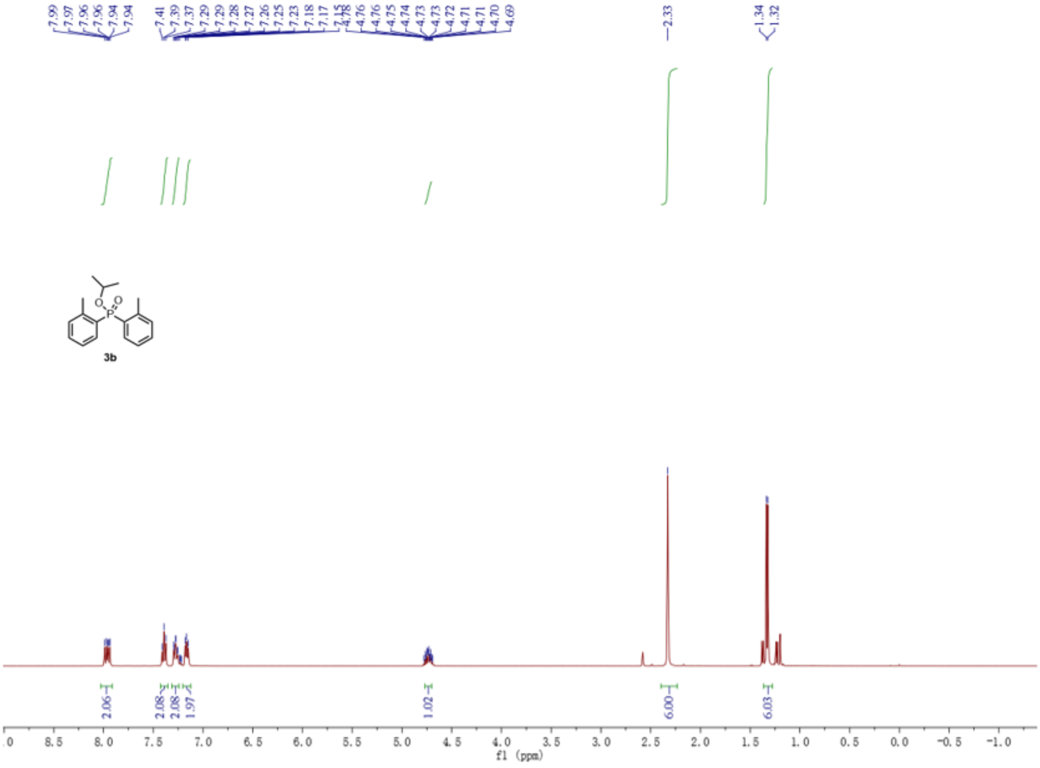

^{13}C NMR (101 MHz, CDCl3) spectrum for 3b

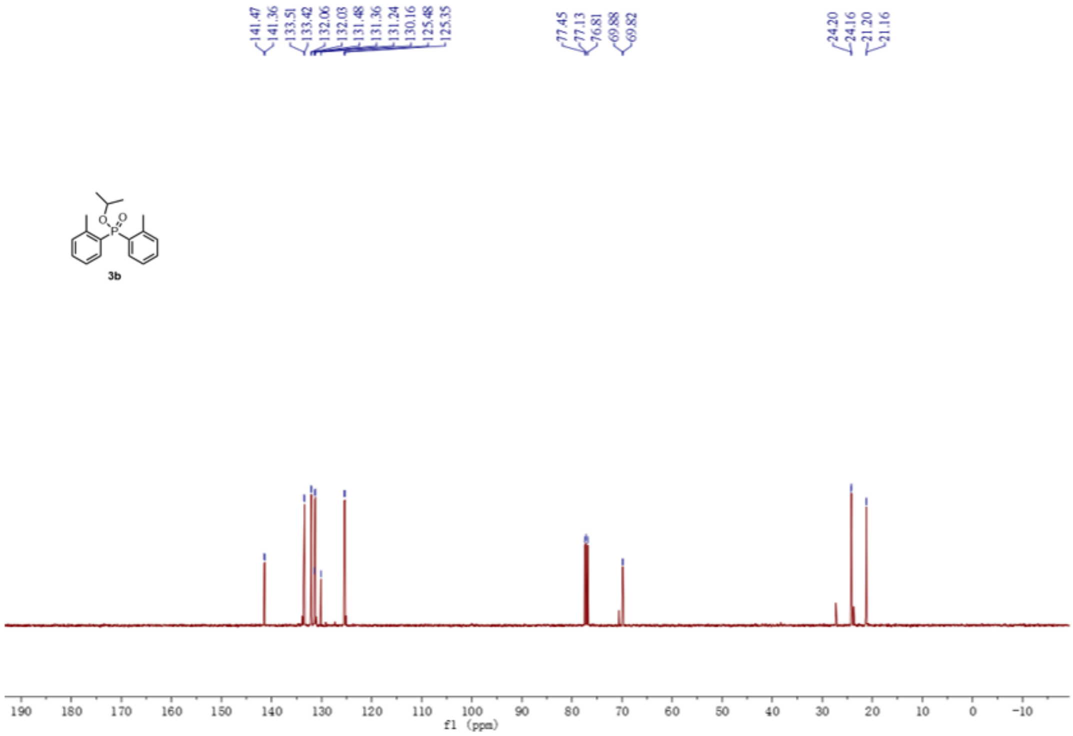

^{31}P NMR (121 MHz, CDCl3) spectrum for 3b

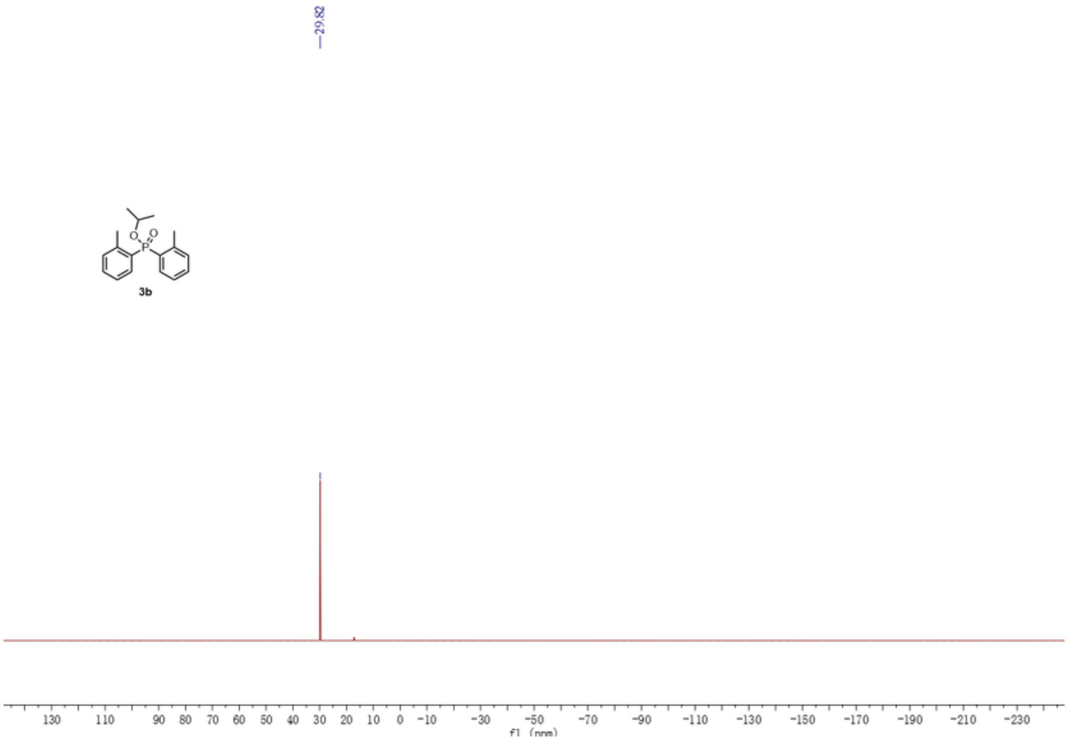

^{1}H NMR (400 MHz, $CDCl_3$) spectrum for 3c

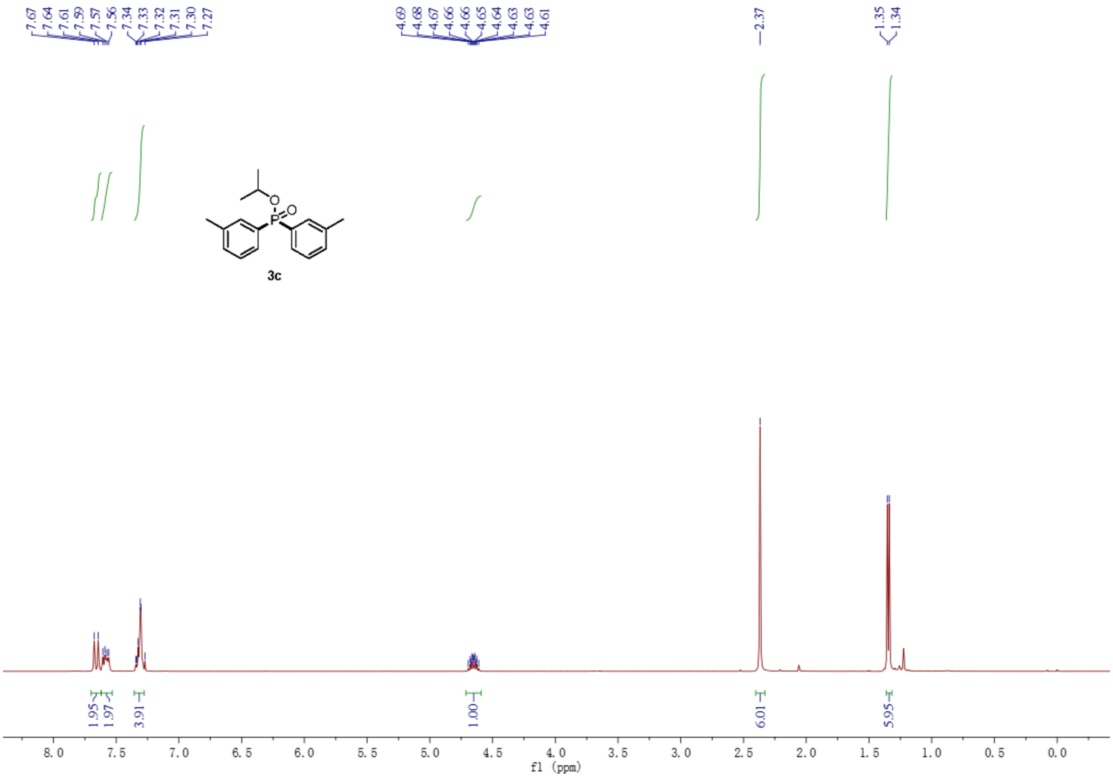

^{13}C NMR (101 MHz, $CDCl_3$) spectrum for 3c

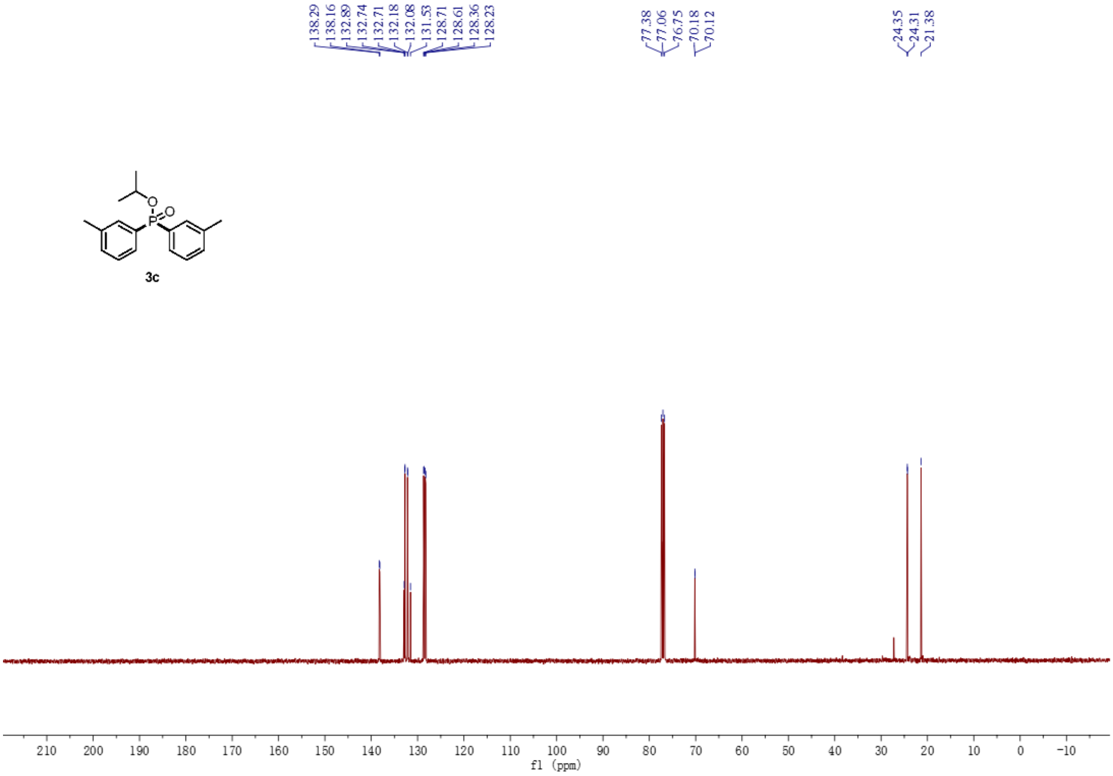

^{31}P NMR (121 MHz, $CDCl_3$) spectrum for 3c

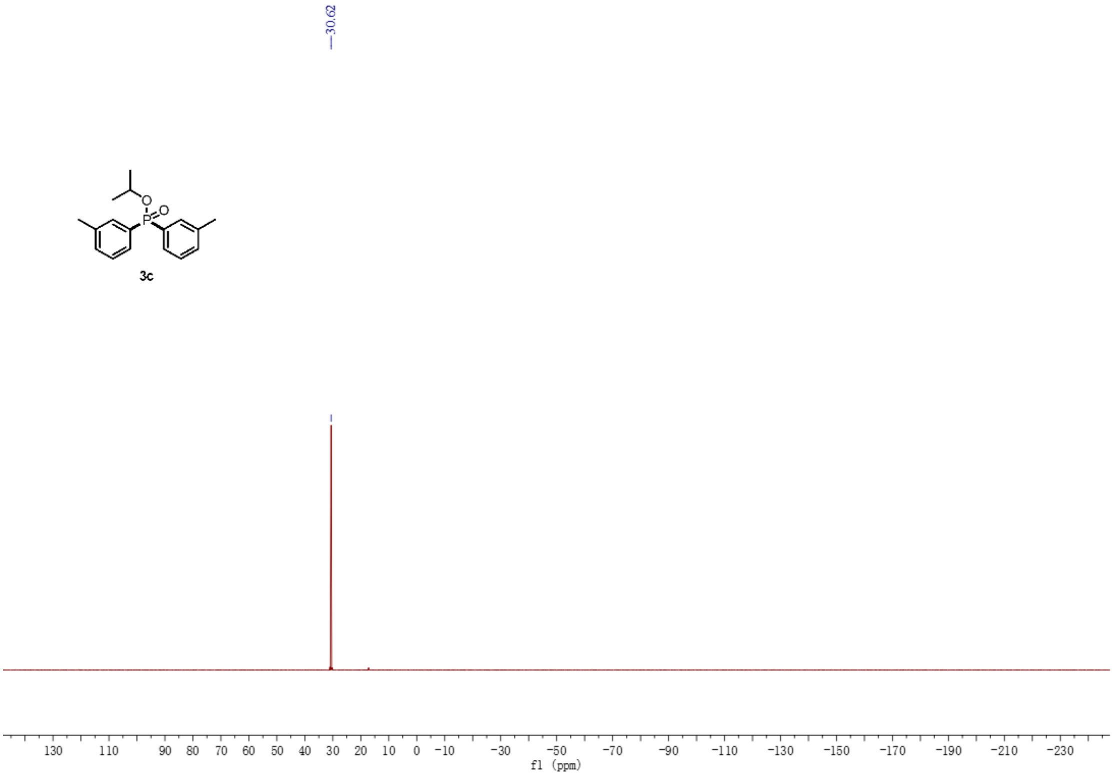

^{1}H NMR (400 MHz, $CDCl_3$) spectrum for 3d

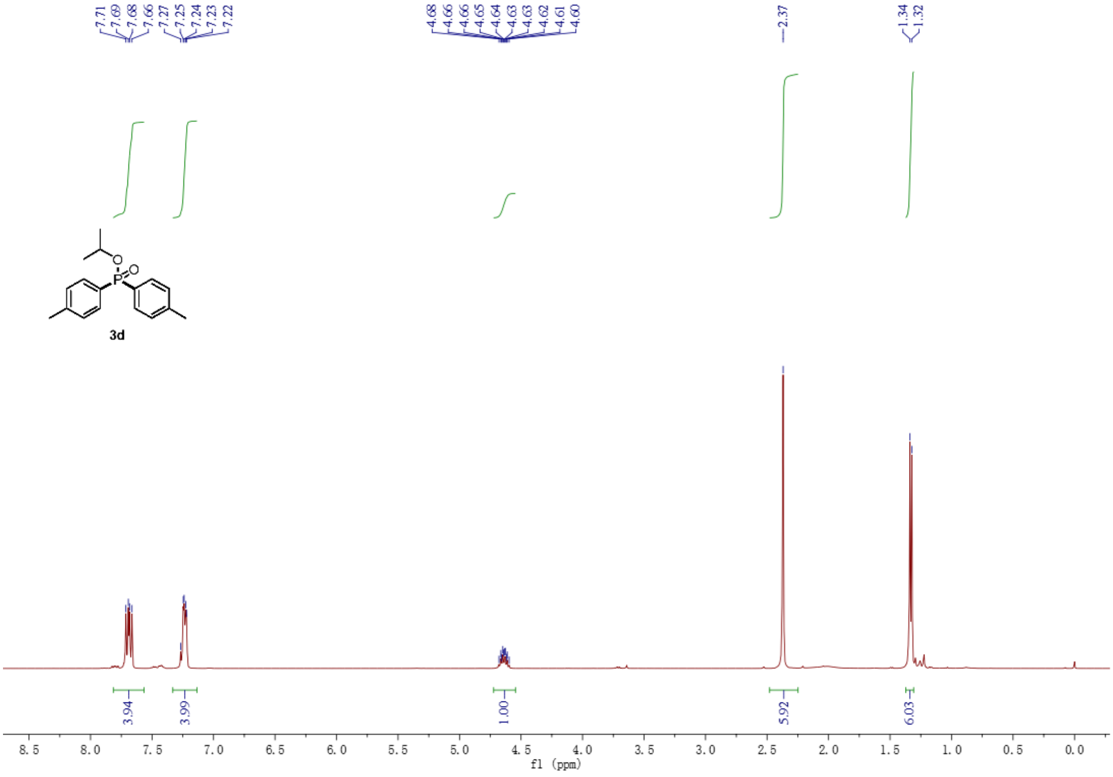

^{13}C NMR (101 MHz, $CDCl_3$) spectrum for 3d

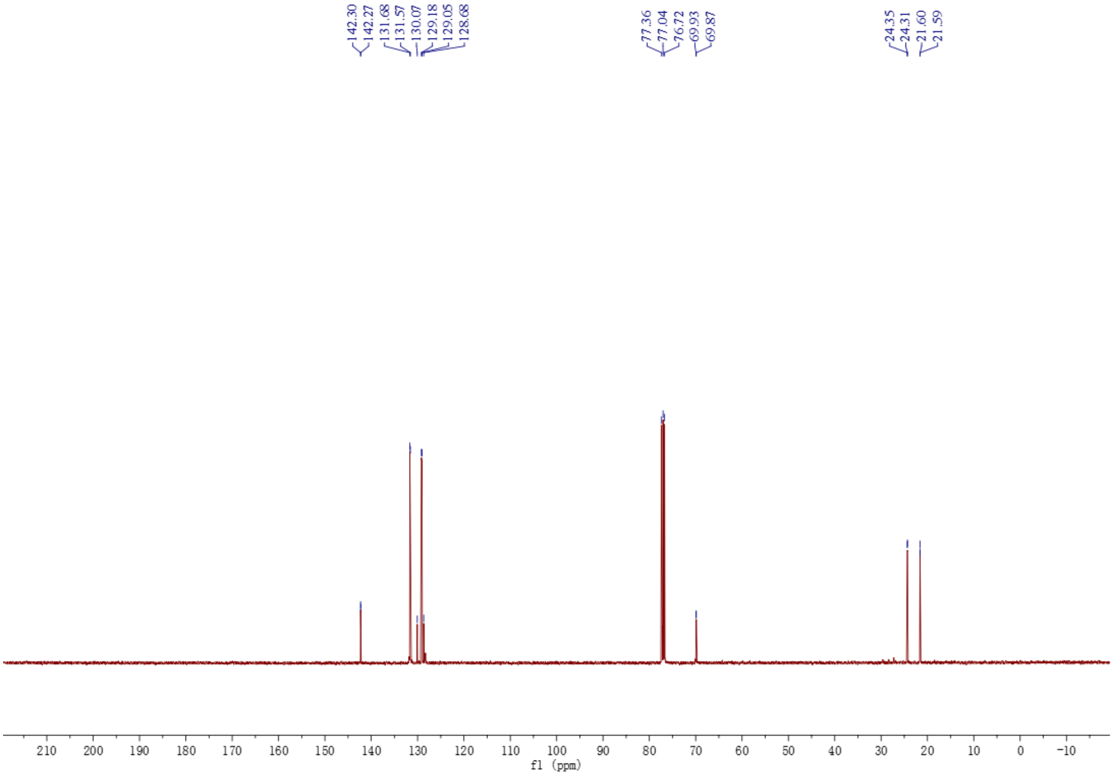

^{31}P NMR (121 MHz, $CDCl_3$) spectrum for 3d

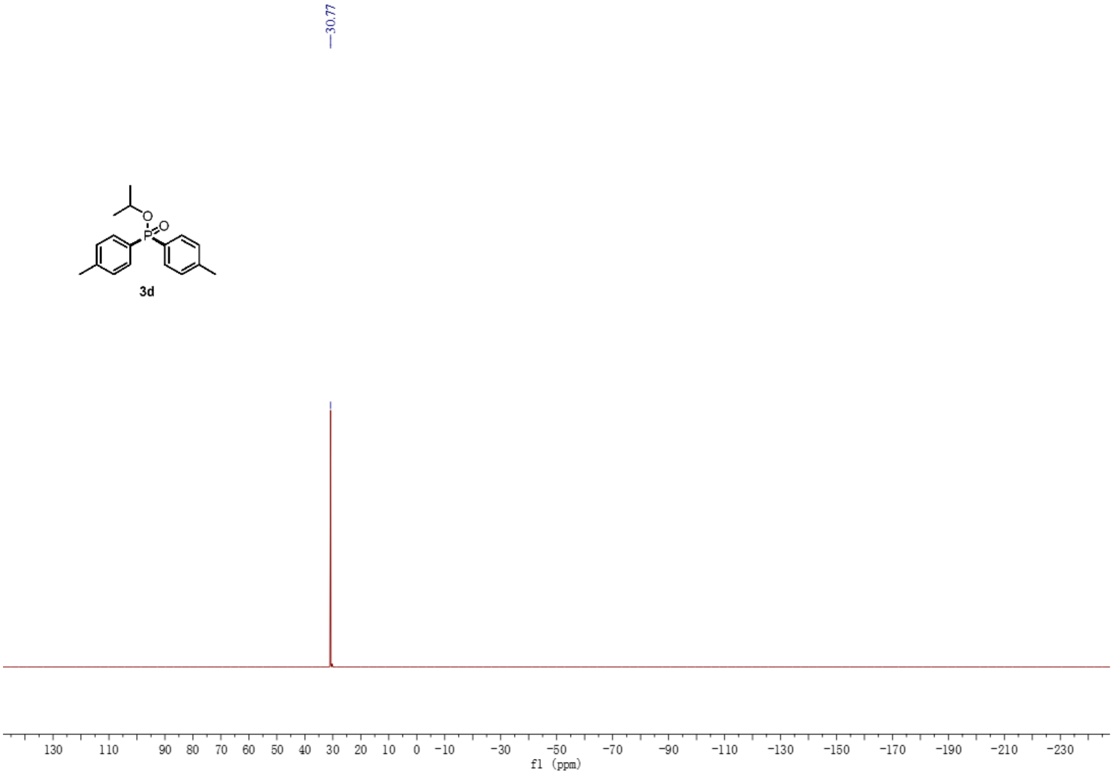

^{1}H NMR (400 MHz, $CDCl_3$) spectrum for 3e

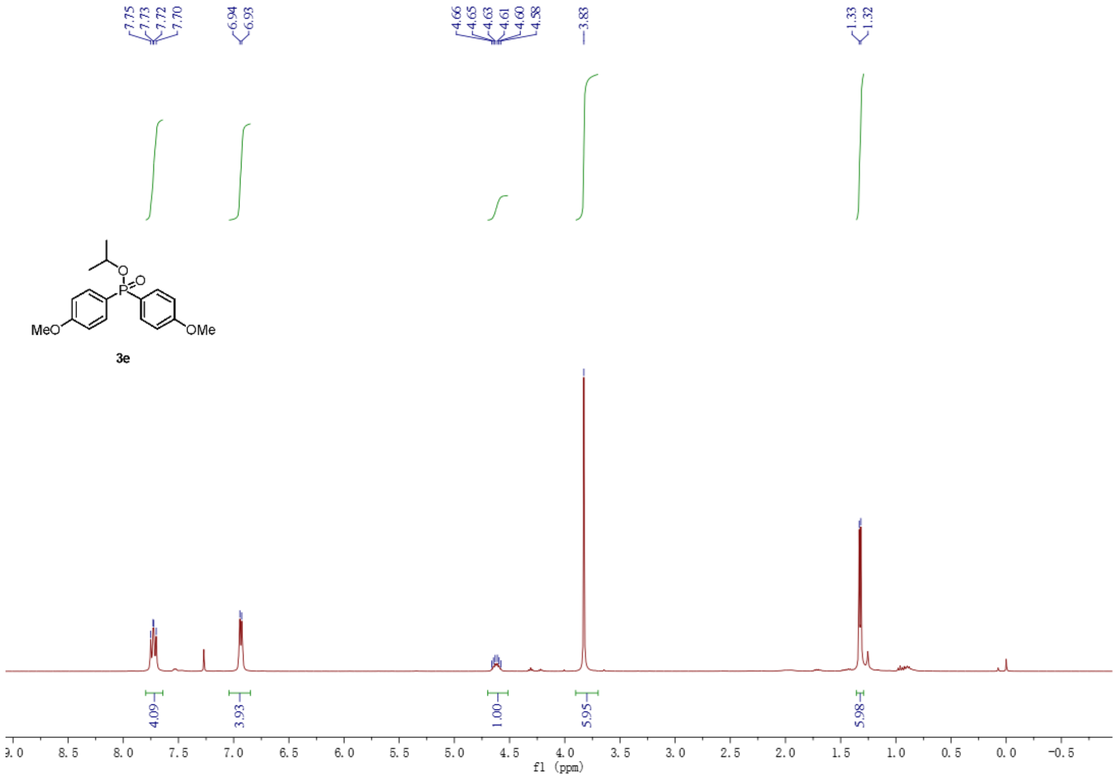

^{13}C NMR (101 MHz, $CDCl_3$) spectrum for 3e

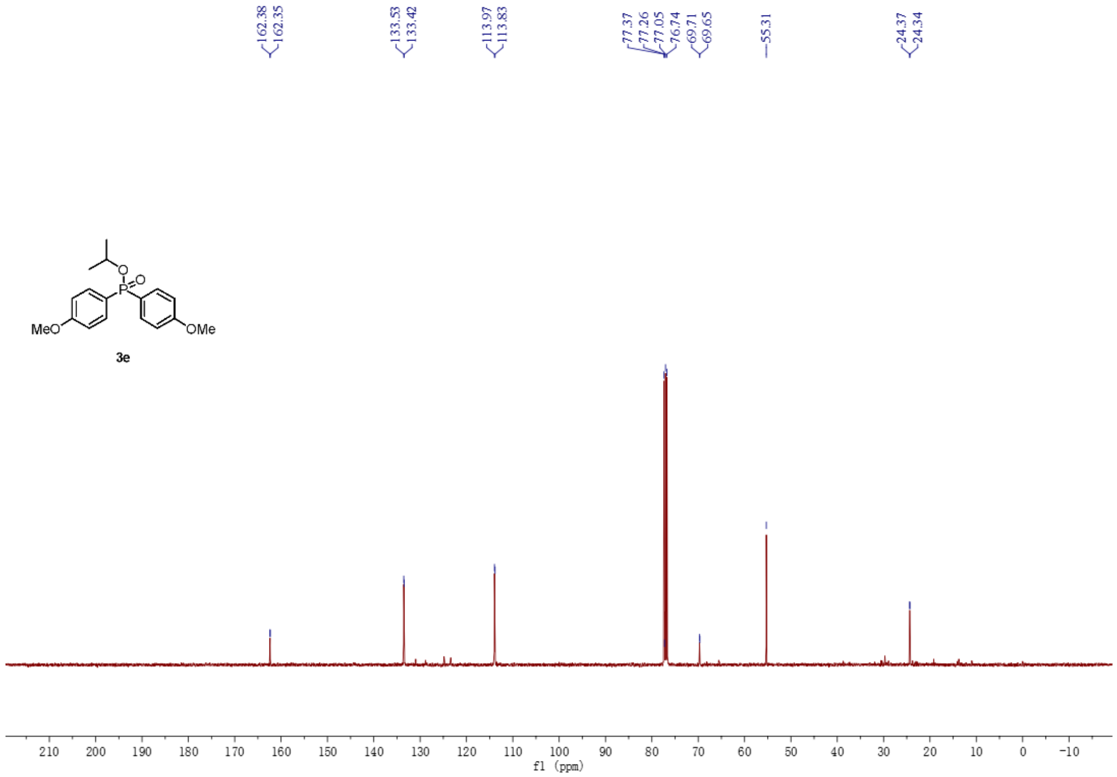

^{31}P NMR (121 MHz, CDCl$_3$) spectrum for 3e

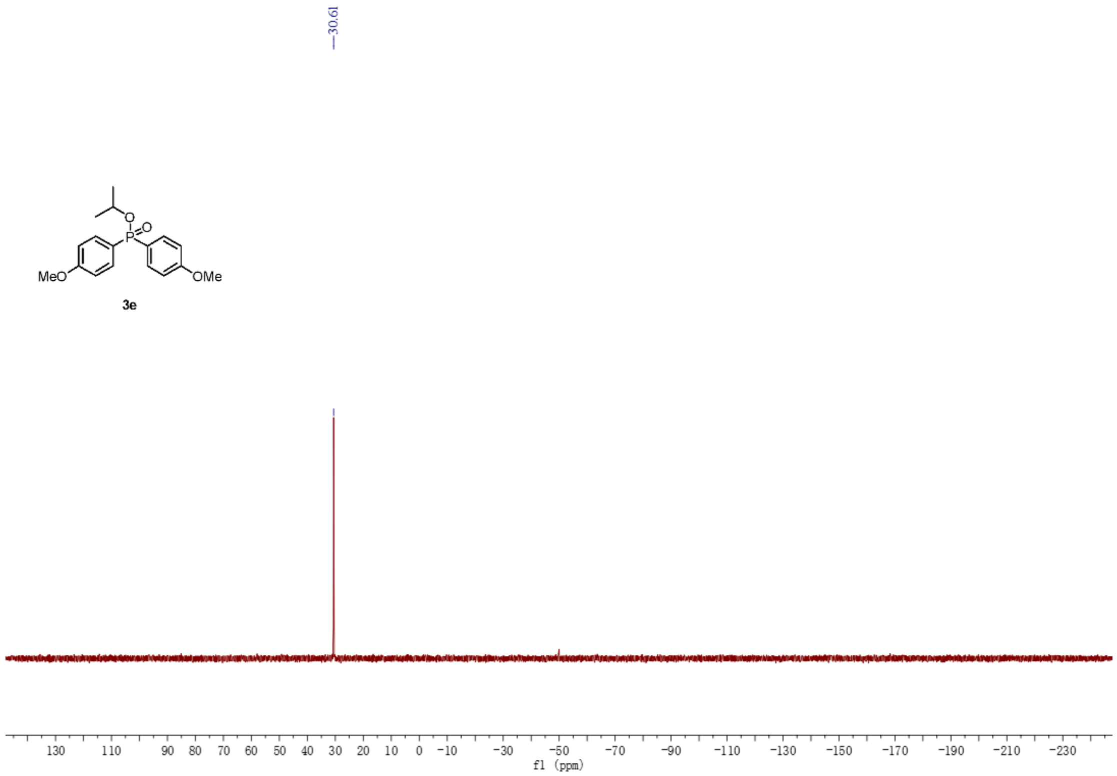

^{1}H NMR (400 MHz, CDCl$_3$) spectrum for 3f

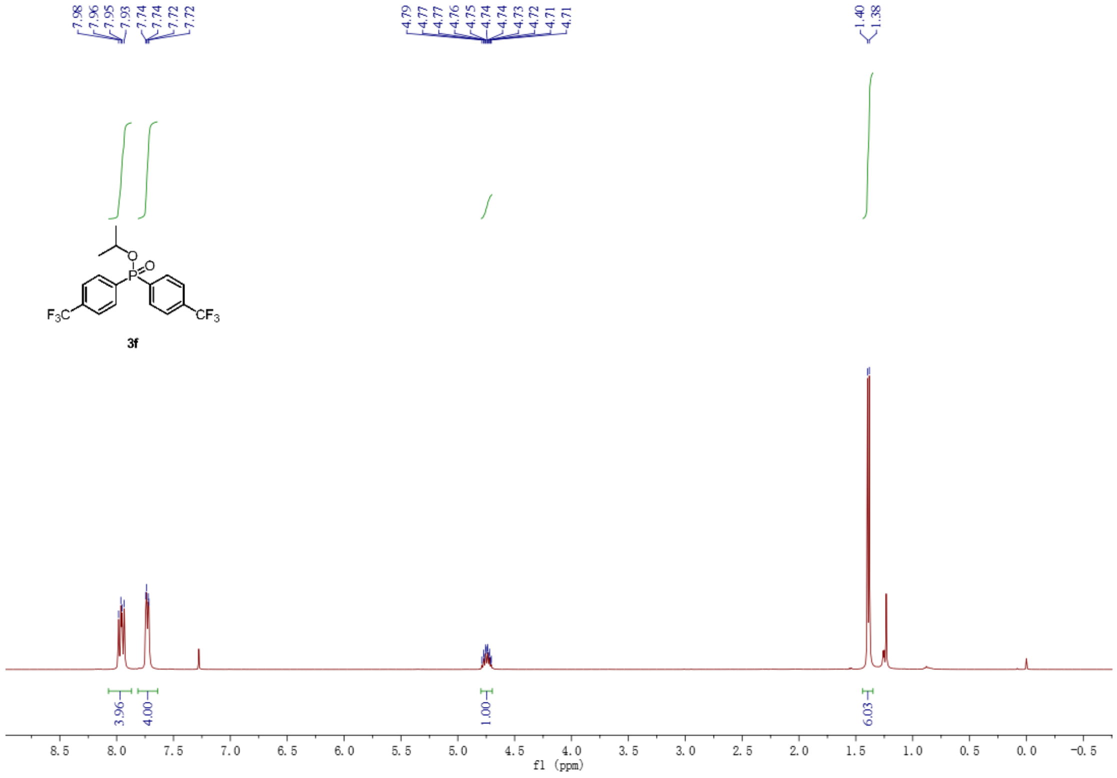

^{13}C NMR (101 MHz, $CDCl_3$) spectrum for 3f

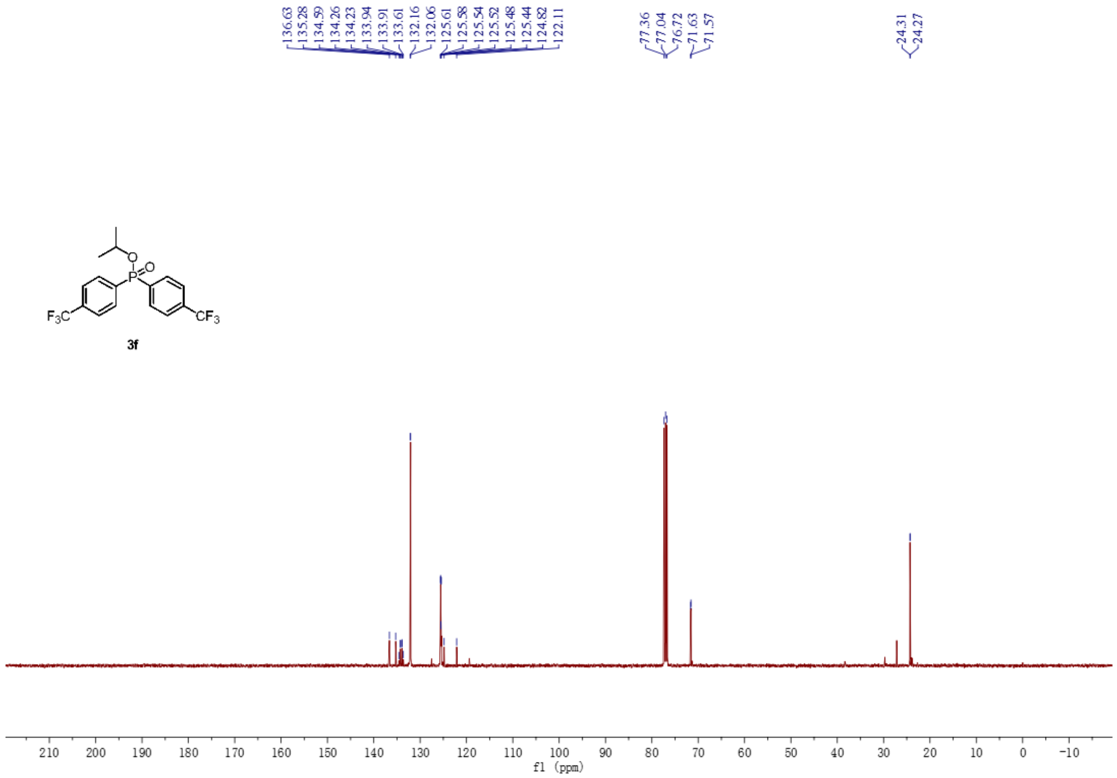

^{31}P NMR (121 MHz, $CDCl_3$) spectrum for 3f

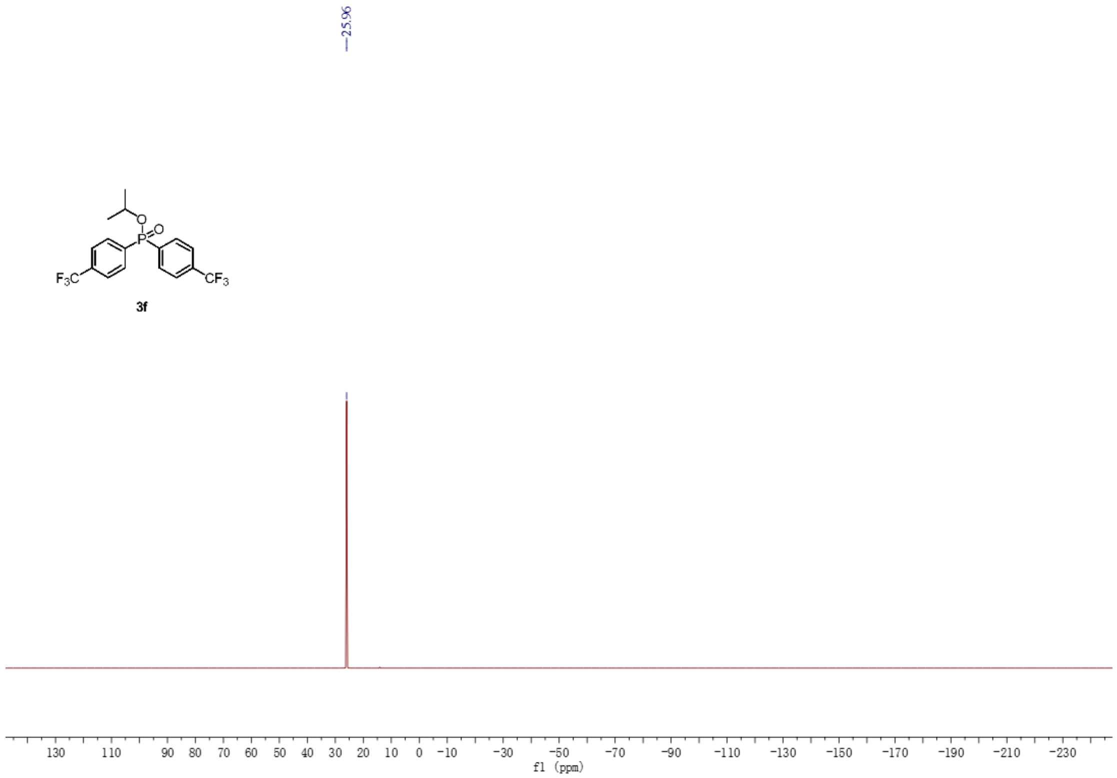

^{19}F NMR (282 MHz, $CDCl_3$) spectrum for 3f

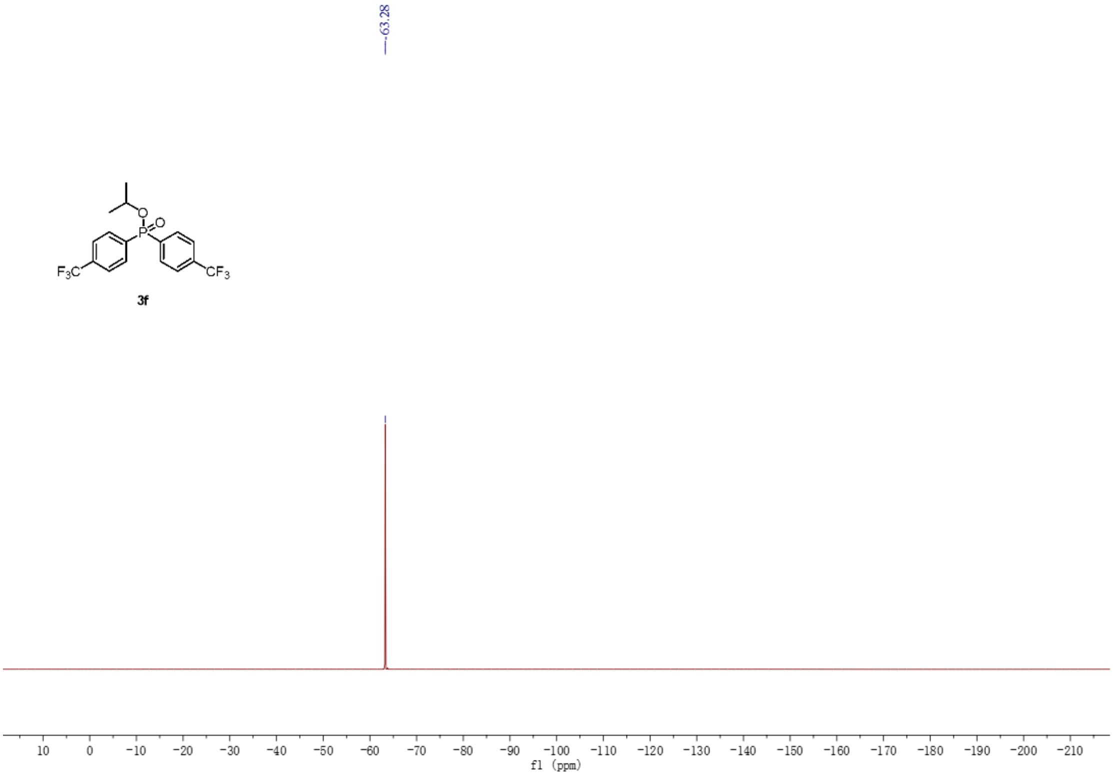

^{1}H NMR (400 MHz, $CDCl_3$) spectrum for 3g

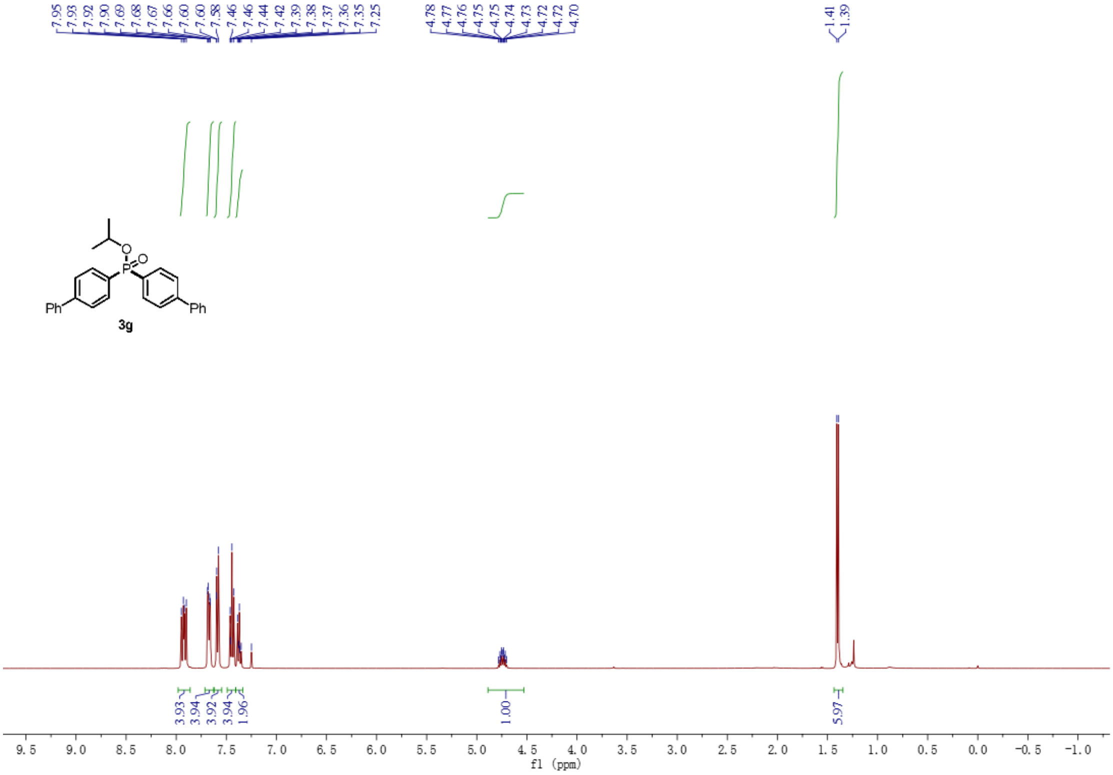

^{13}C NMR (101 MHz, $CDCl_3$) spectrum for 3g

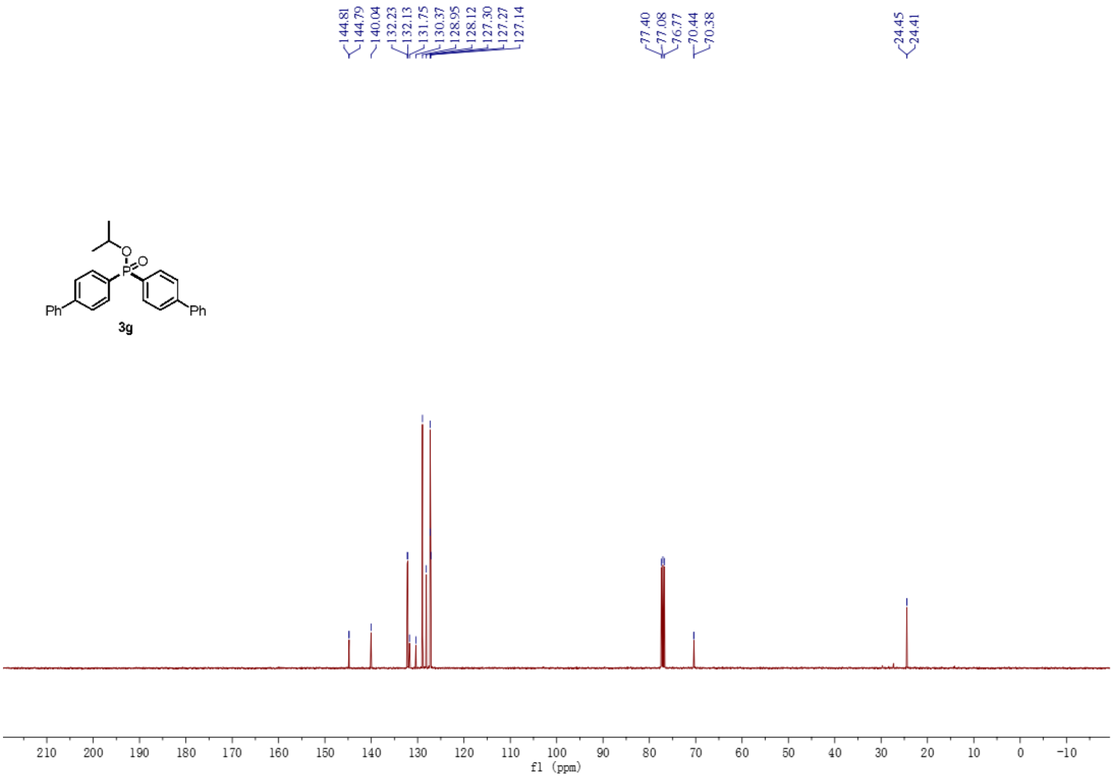

^{31}P NMR (121 MHz, $CDCl_3$) spectrum for 3g

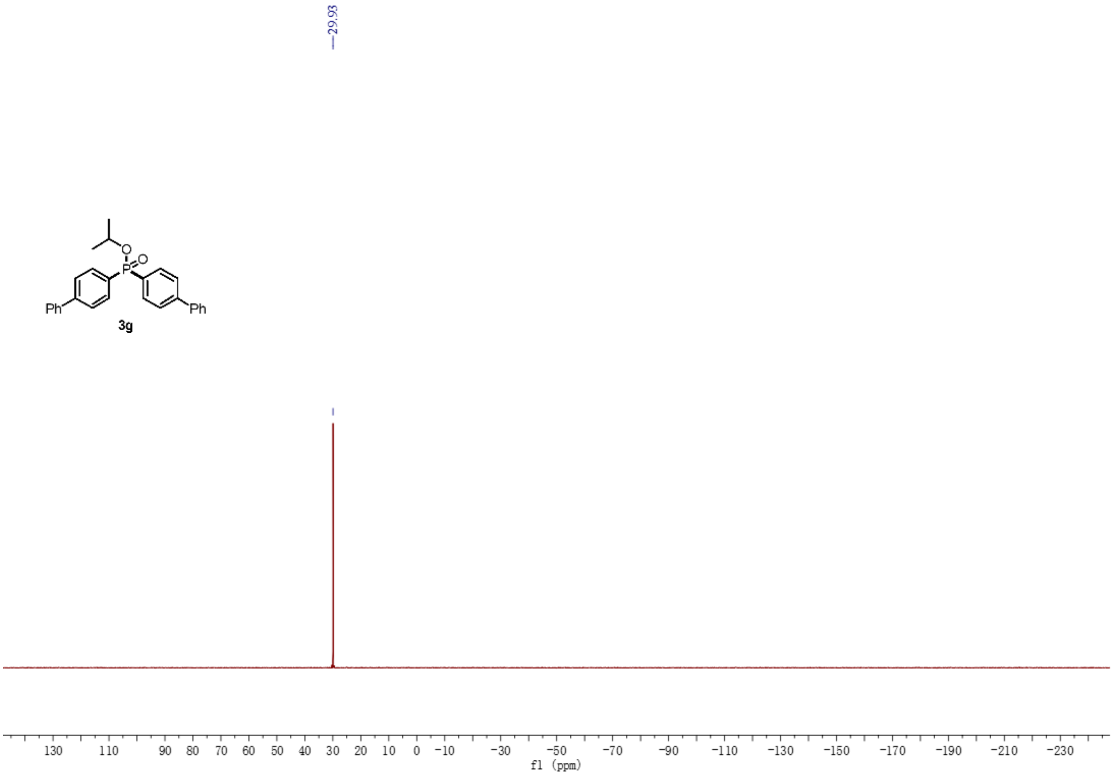

^{1}H NMR (400 MHz, CDCl3) spectrum for 3h

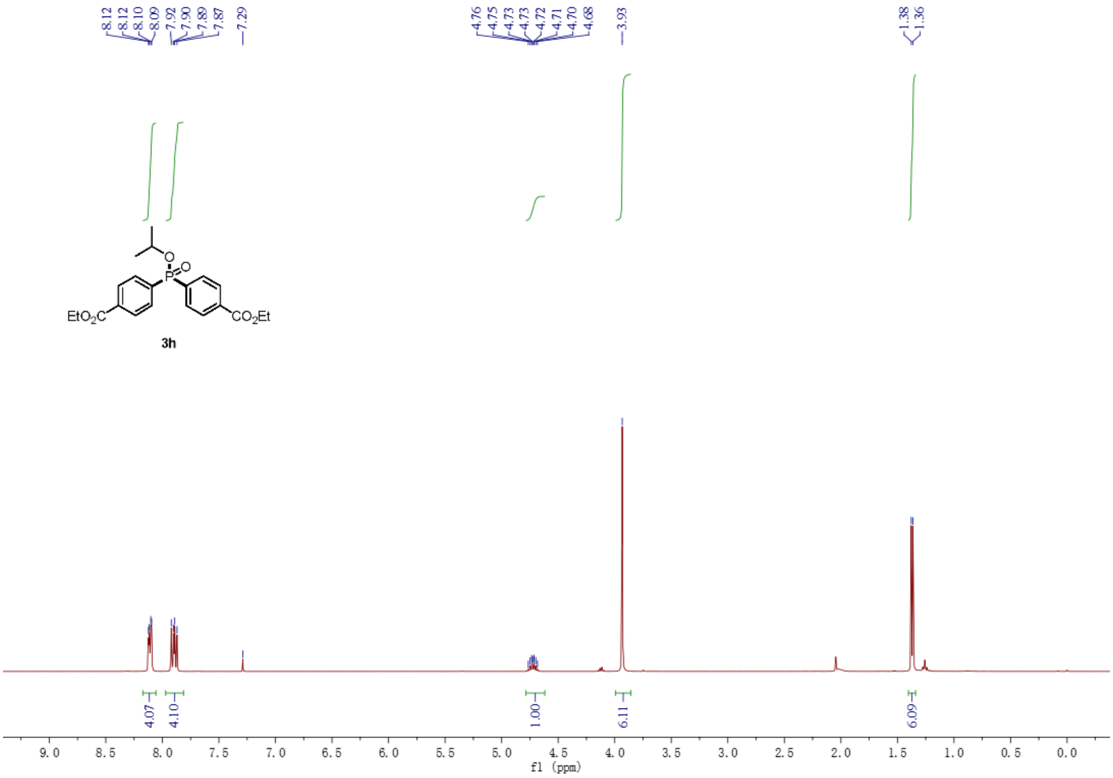

^{13}C NMR (101 MHz, CDCl3) spectrum for 3h

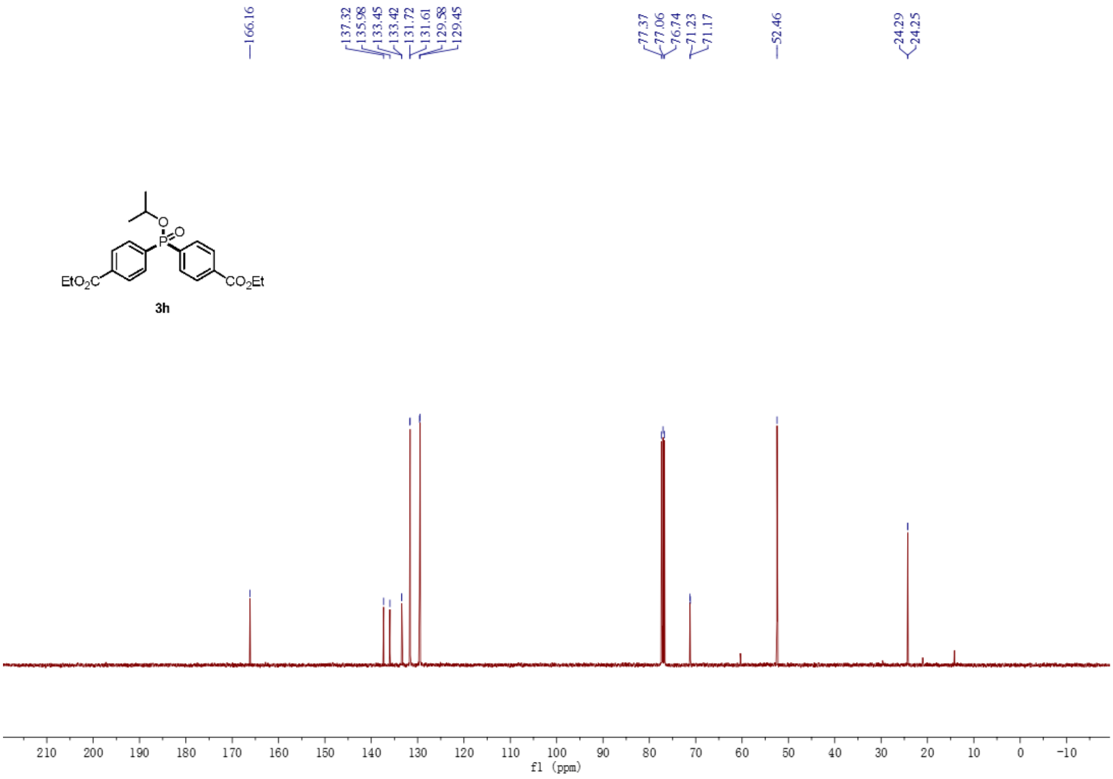

^{31}P NMR (121 MHz, $CDCl_3$) spectrum for 3h

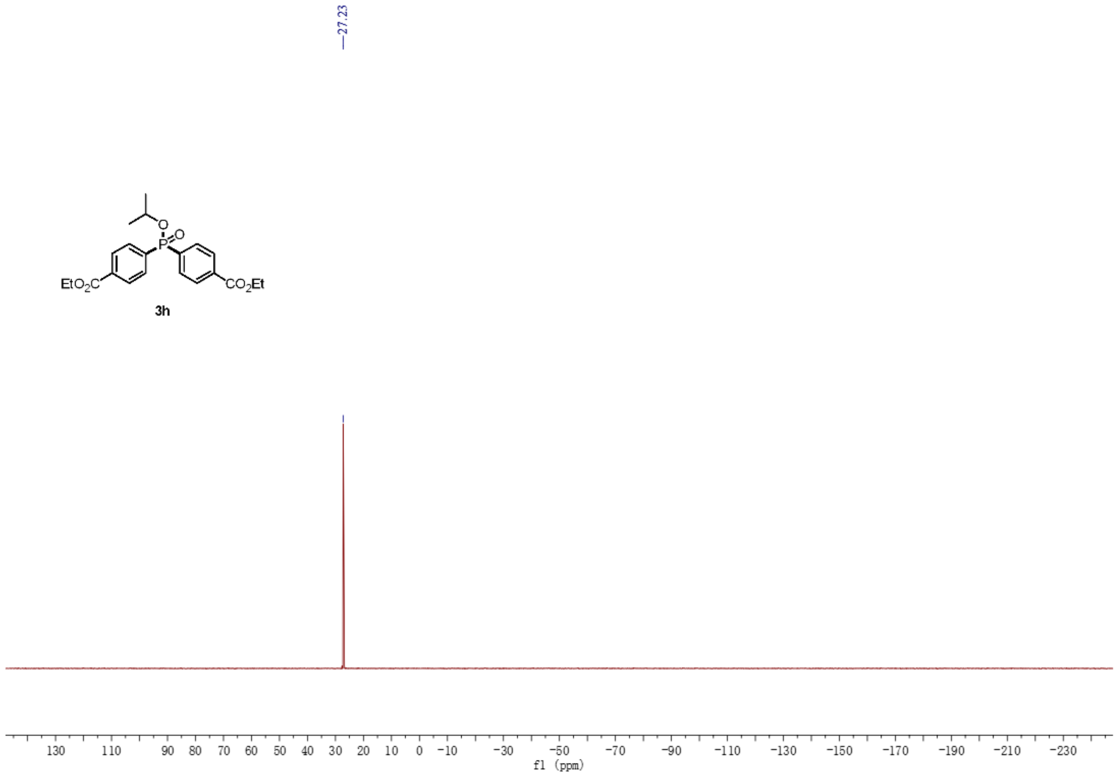

^{1}H NMR (400 MHz, $CDCl_3$) spectrum for 3i

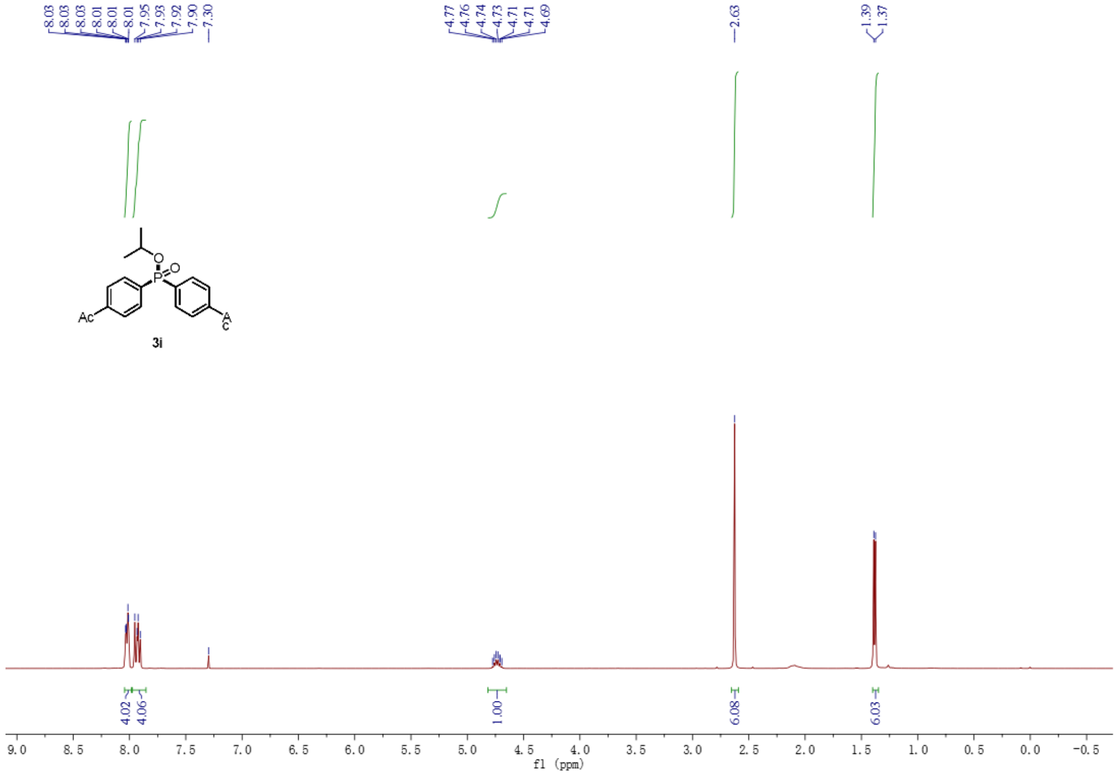

^{13}C NMR (101 MHz, CDCl$_3$) spectrum for 3i

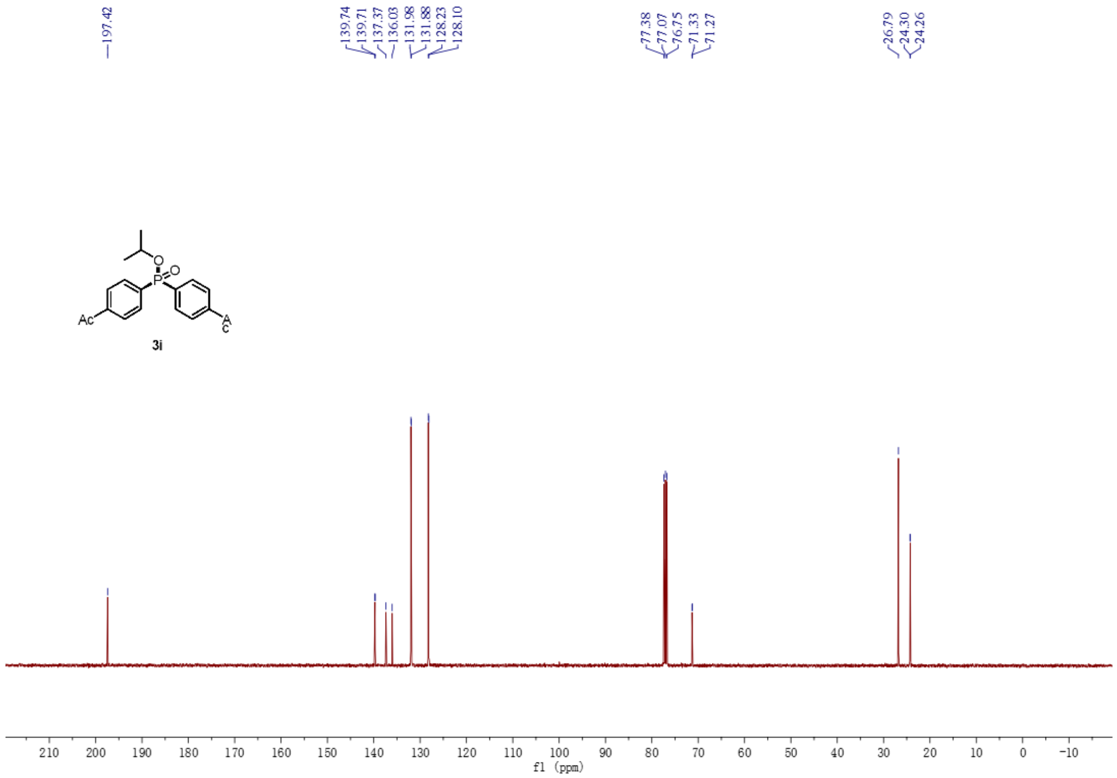

^{31}P NMR (121 MHz, CDCl$_3$) spectrum for 3i

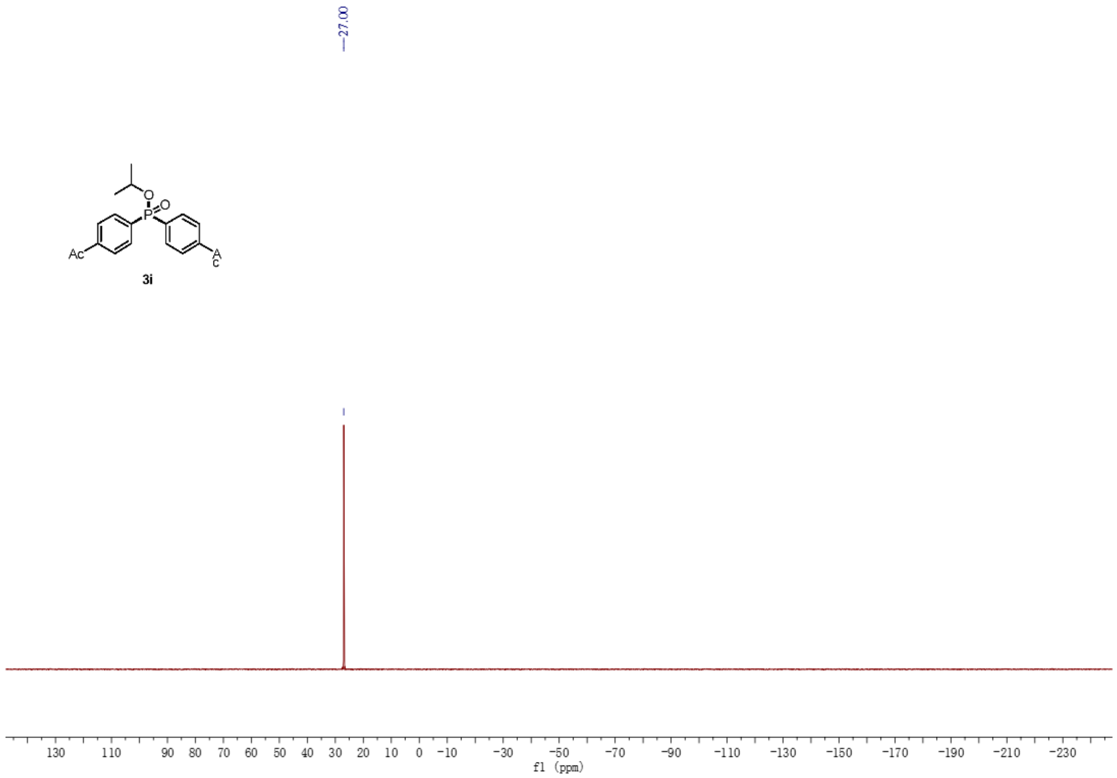

^{1}H NMR (400 MHz, $CDCl_3$) spectrum for 3j

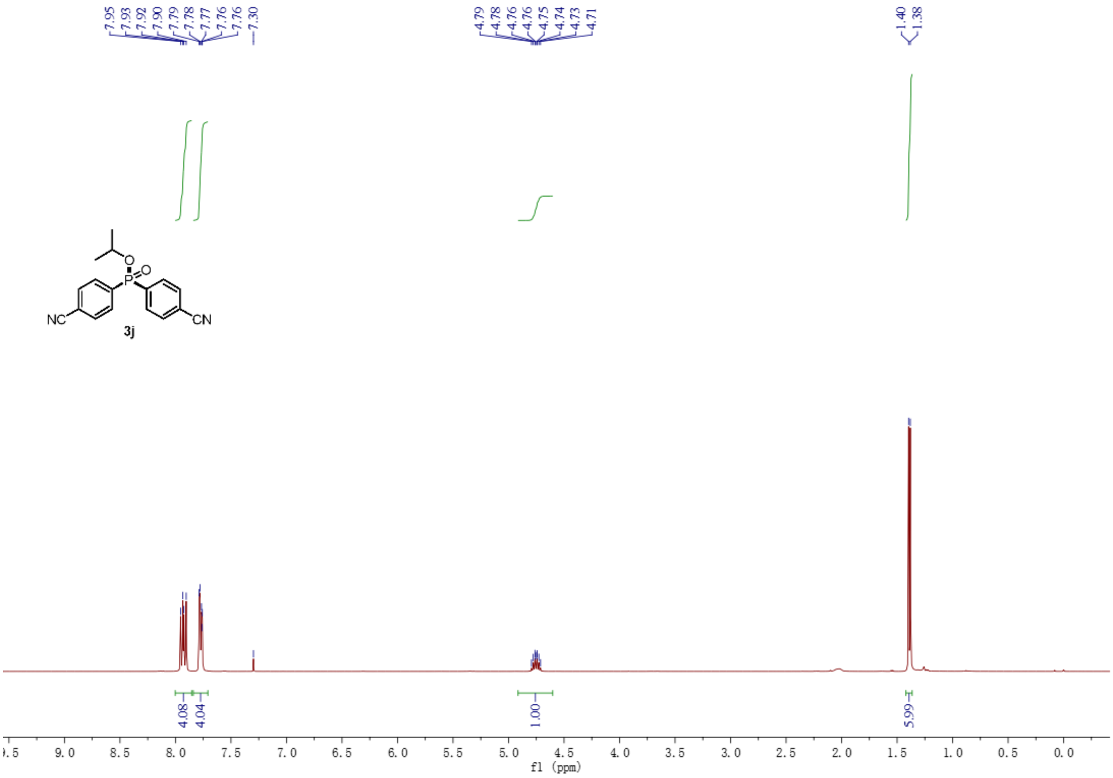

^{13}C NMR (101 MHz, $CDCl_3$) spectrum for 3j

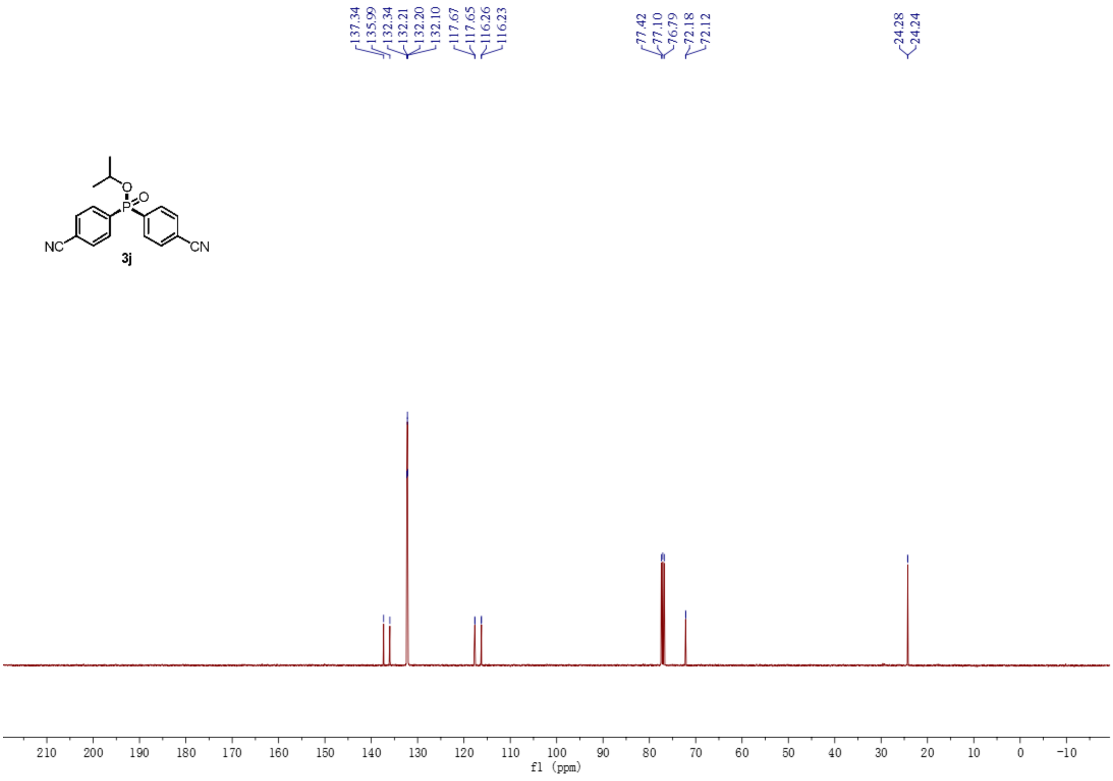

^{31}P NMR (121 MHz, $CDCl_3$) spectrum for 3j

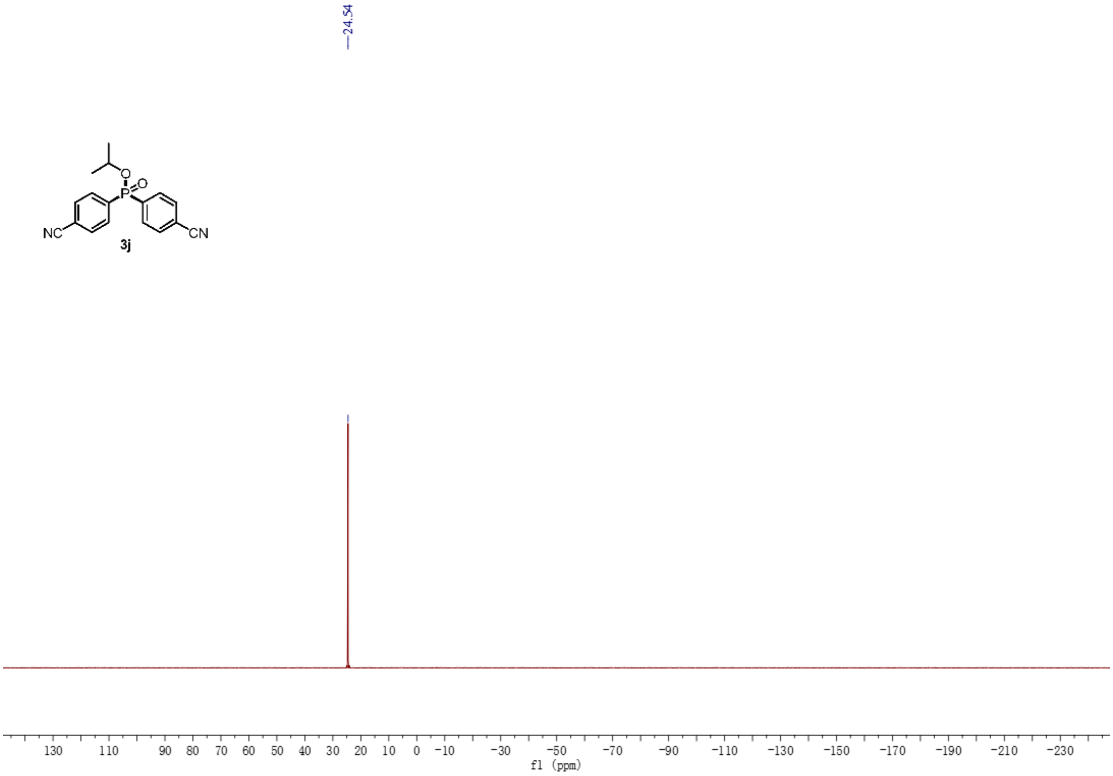

^{1}H NMR (400 MHz, $CDCl_3$) spectrum for 3k

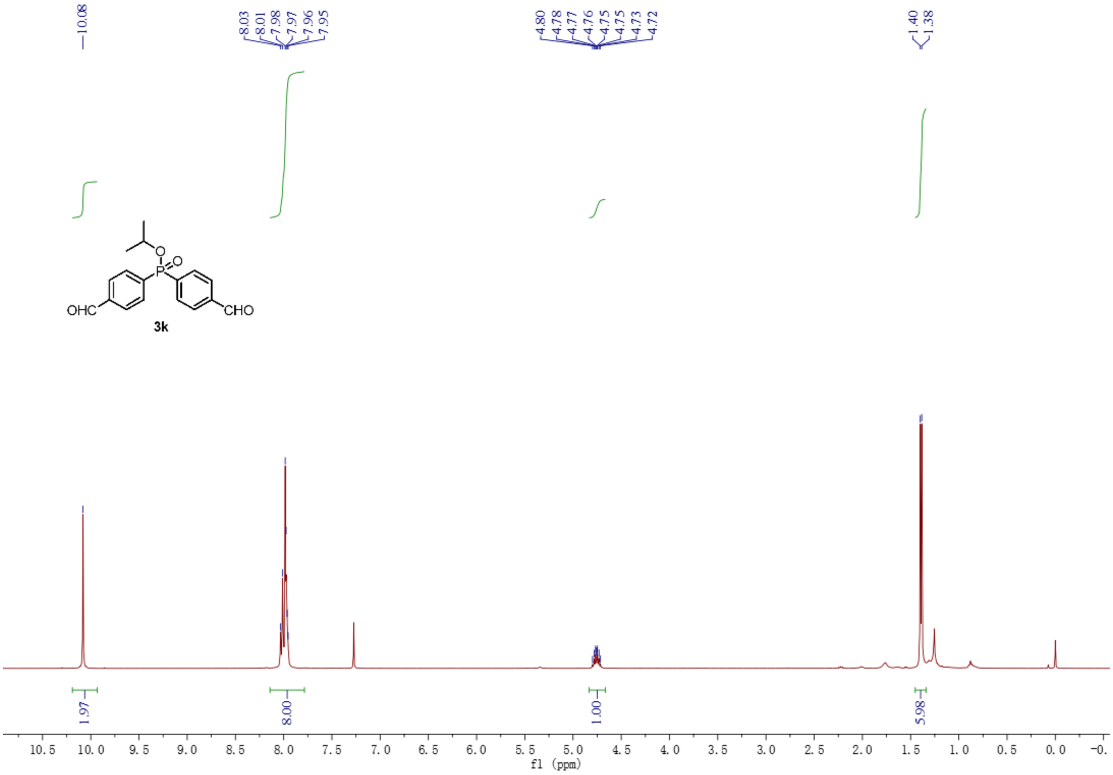

^{13}C NMR (101 MHz, $CDCl_3$) spectrum for 3k

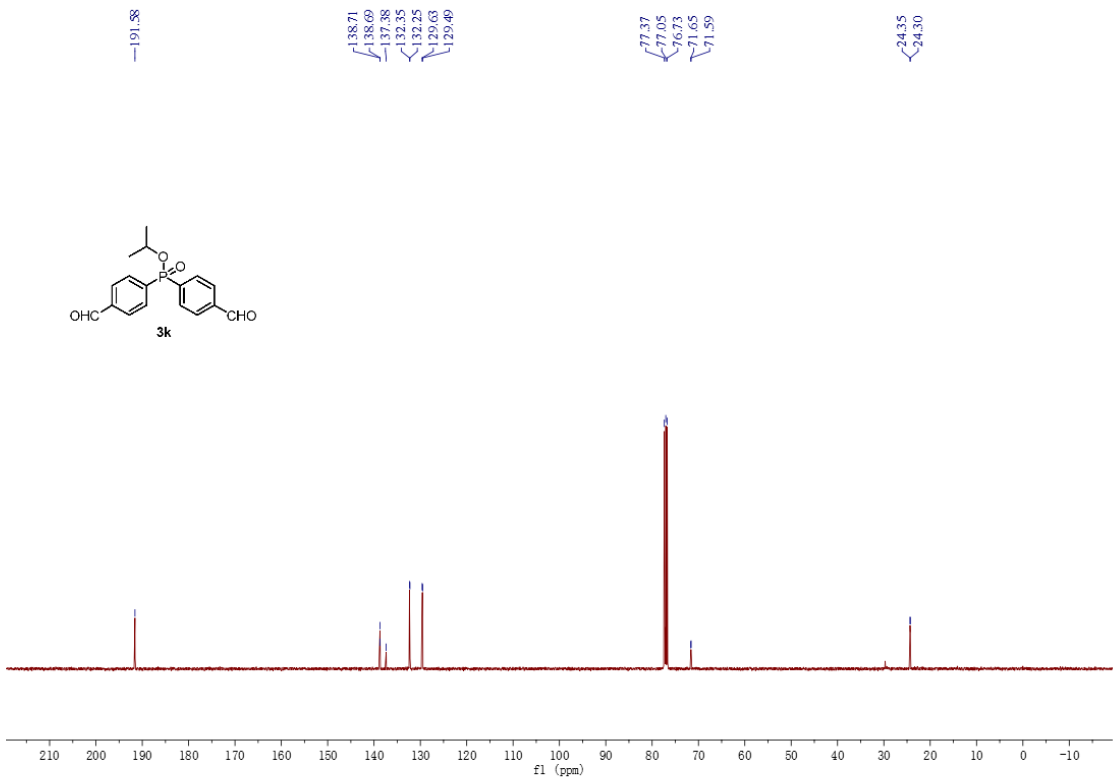

^{31}P NMR (121 MHz, $CDCl_3$) spectrum for 3k

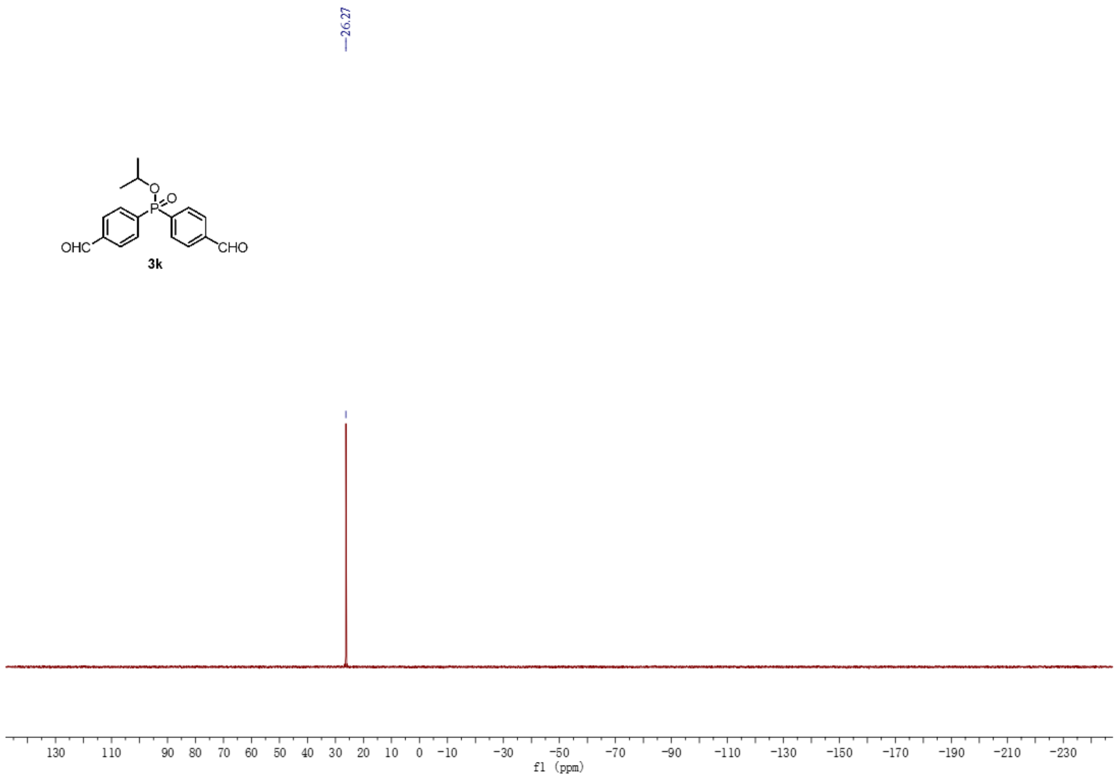

^{1}H NMR (400 MHz, $CDCl_3$) spectrum for 3l

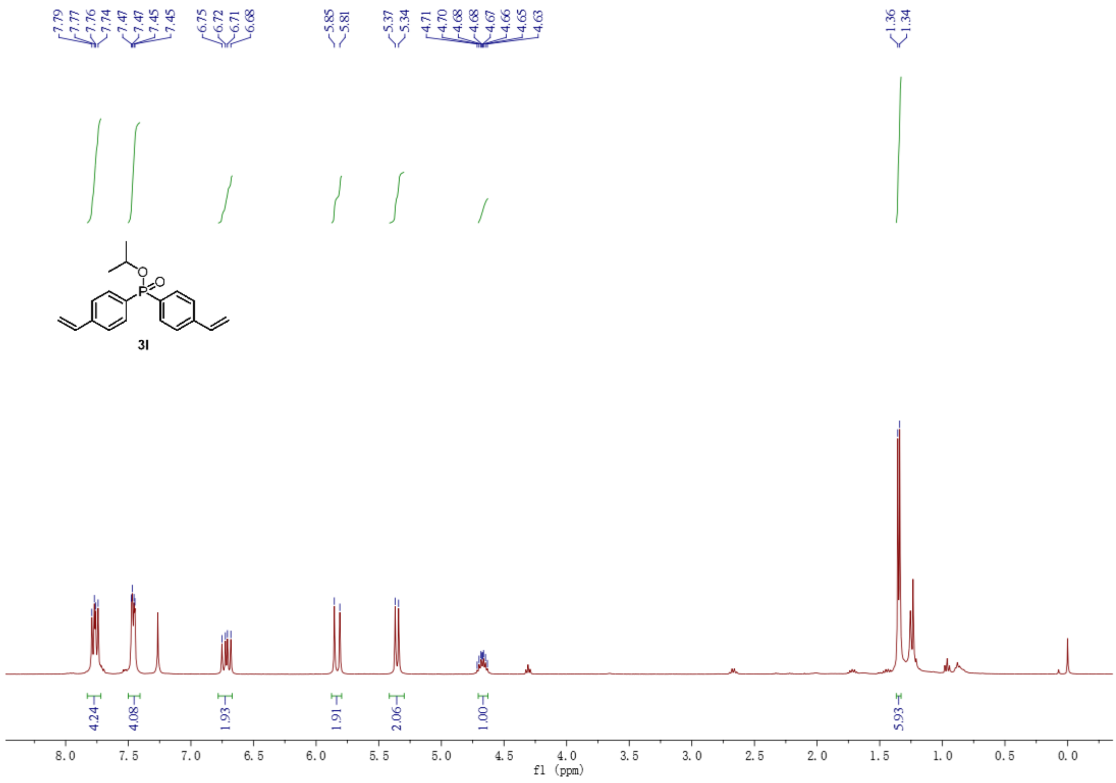

^{13}C NMR (101 MHz, $CDCl_3$) spectrum for 3l

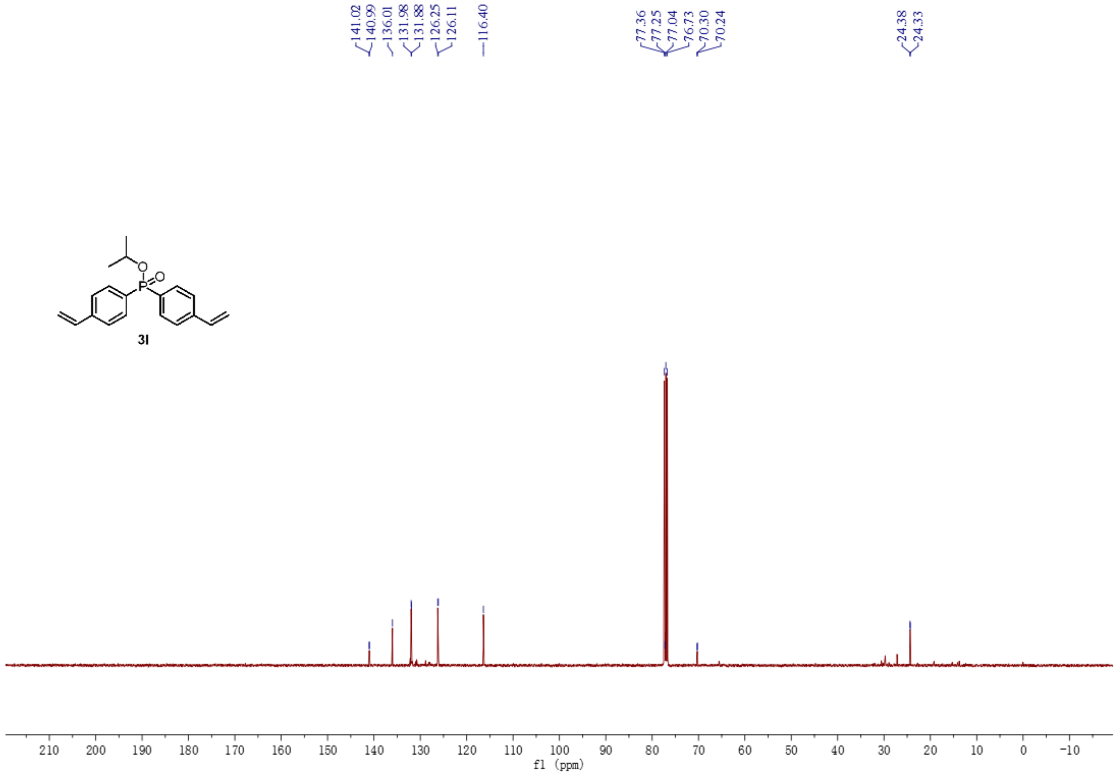

^{31}P NMR (121 MHz, $CDCl_3$) spectrum for 3l

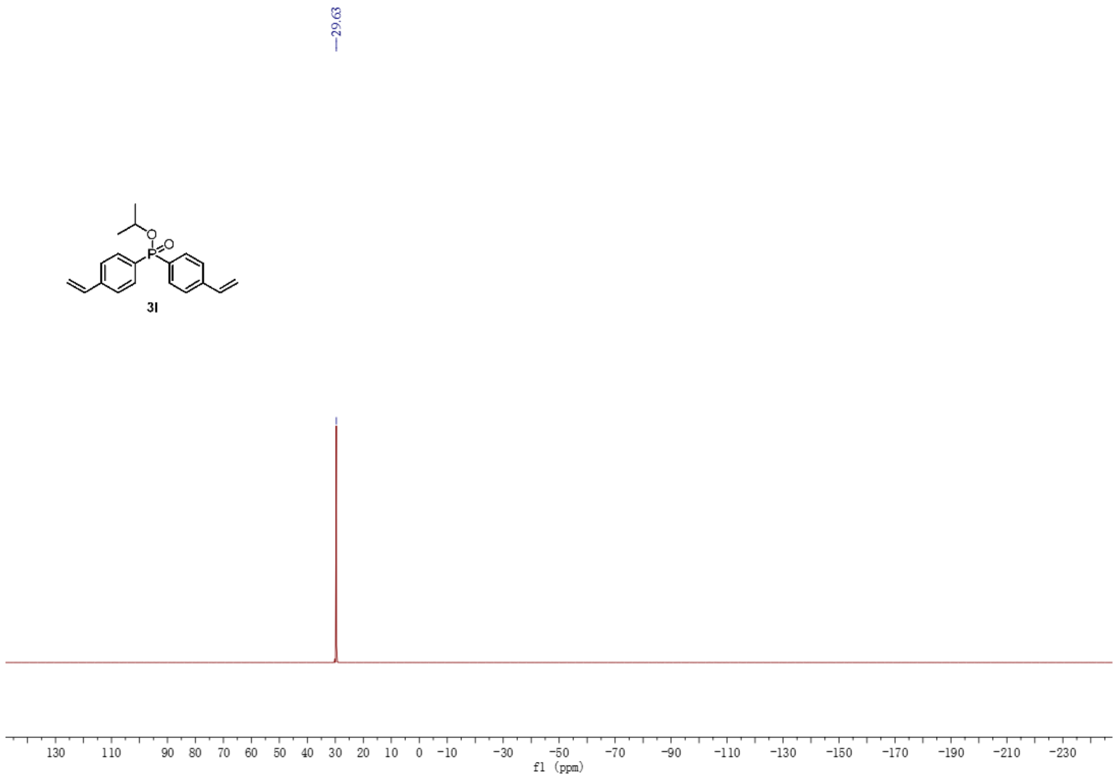

^{1}H NMR (400 MHz, $CDCl_3$) spectrum for 3m

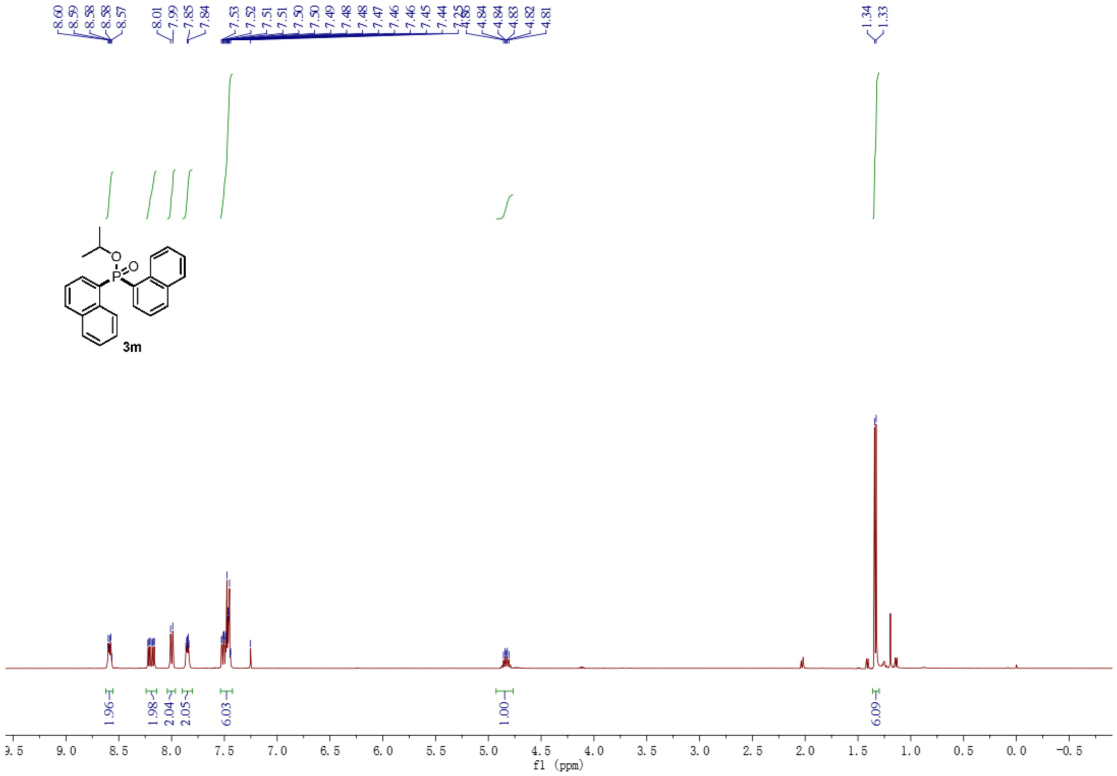

^{13}C NMR (101 MHz, $CDCl_3$) spectrum for 3m

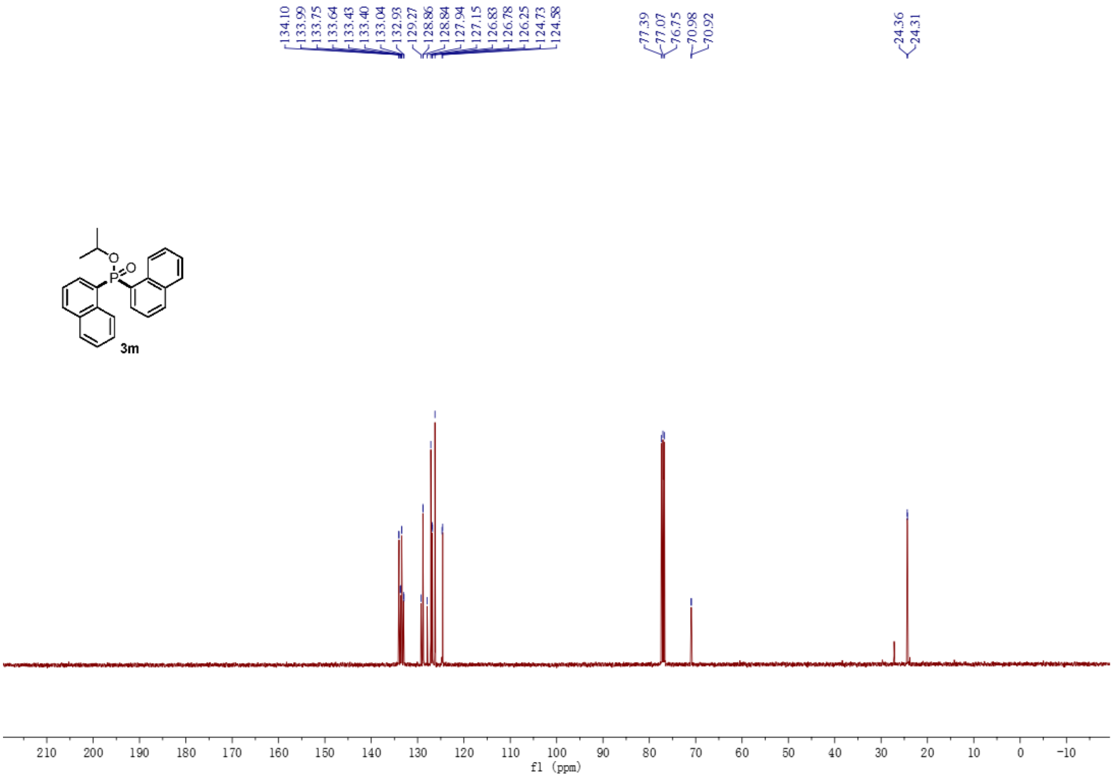

^{31}P NMR (121 MHz, $CDCl_3$) spectrum for 3m

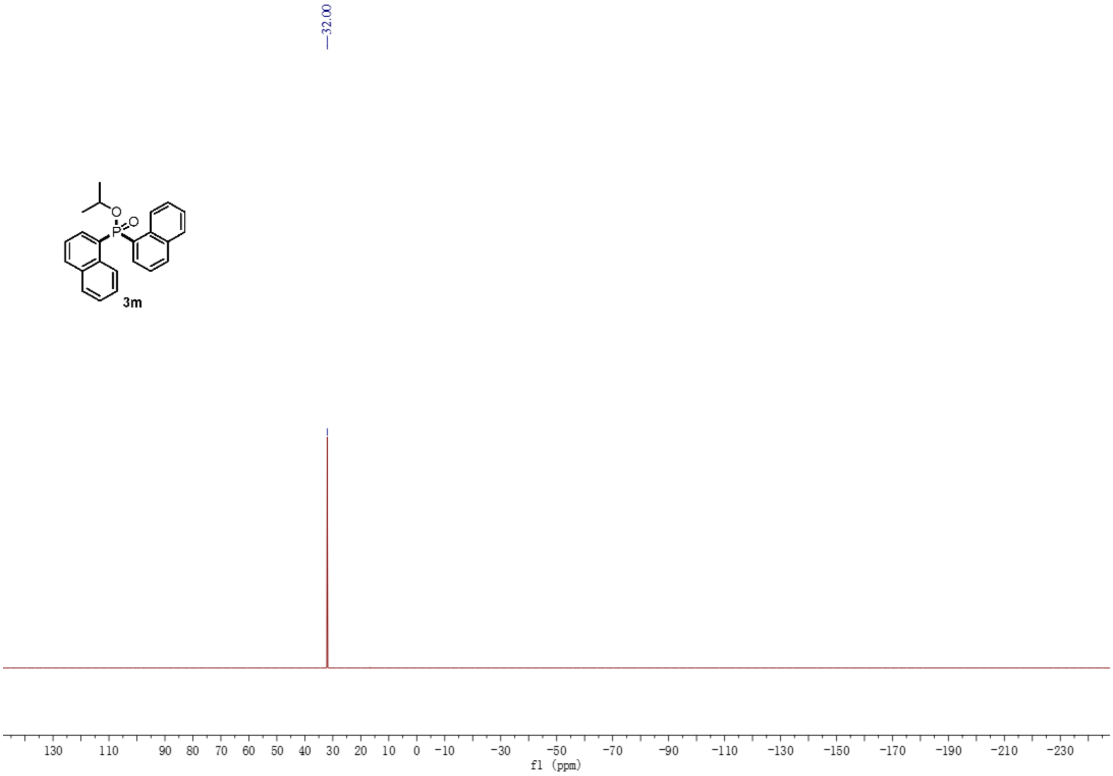

^{1}H NMR (400 MHz, $CDCl_3$) spectrum for 3n

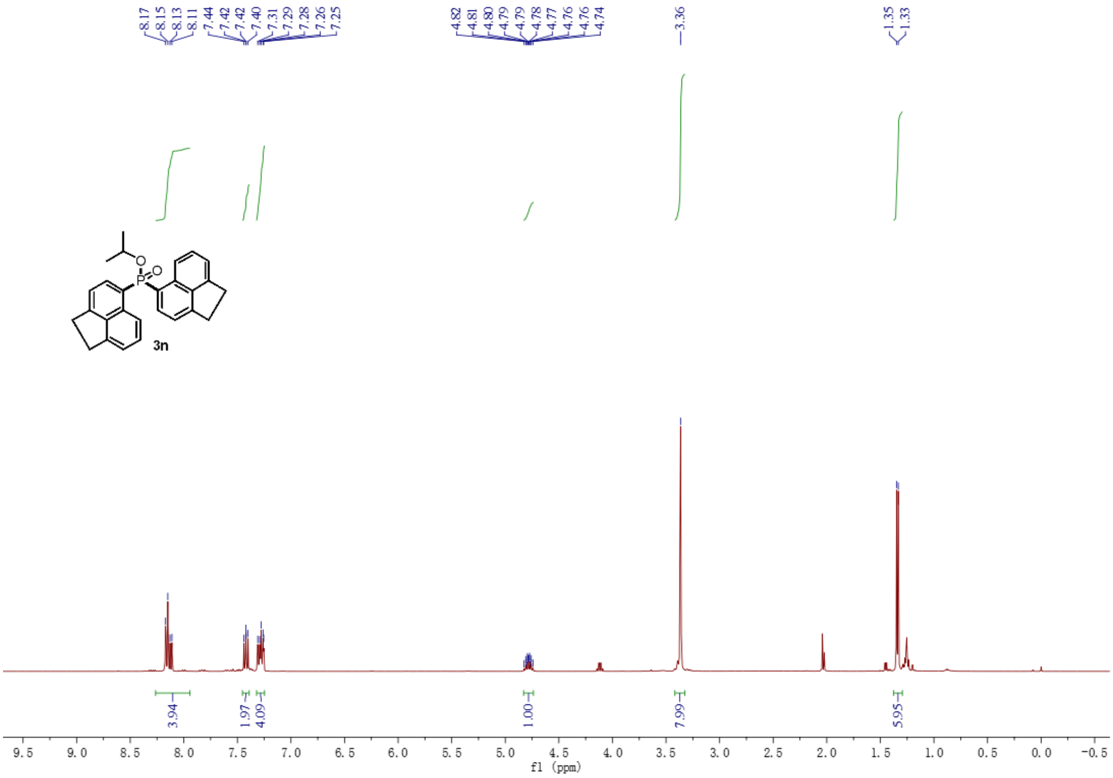

^{13}C NMR (101 MHz, $CDCl_3$) spectrum for 3n

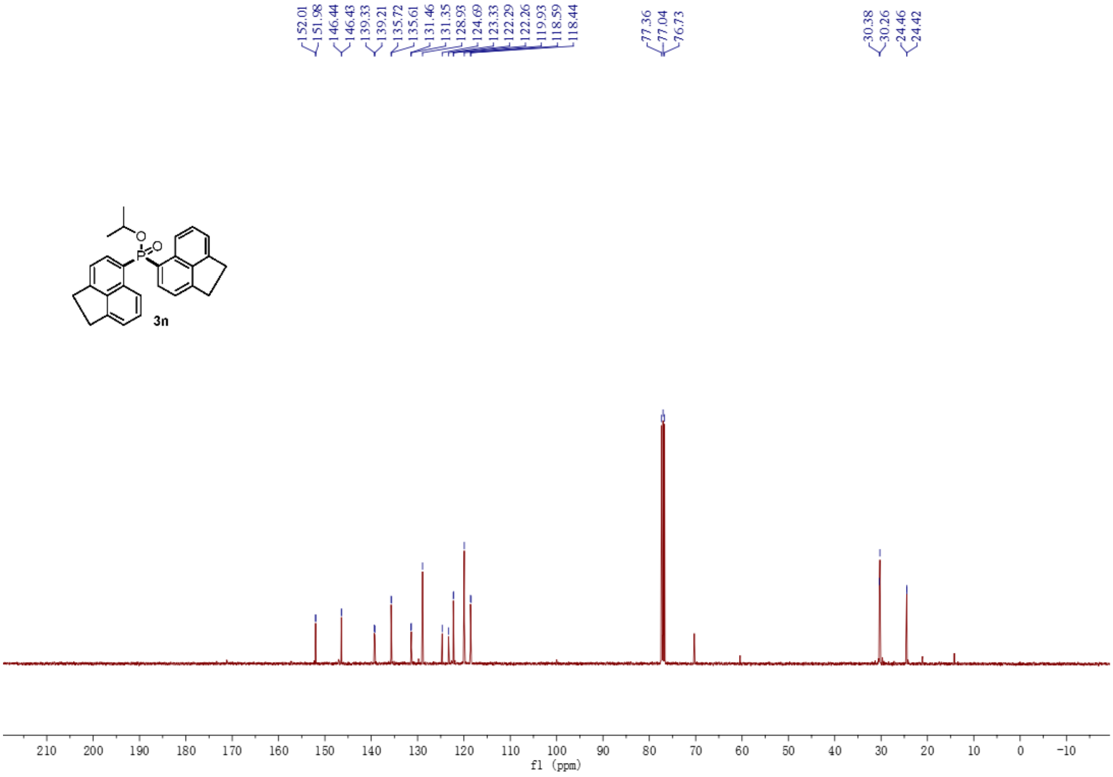

^{31}P NMR (121 MHz, $CDCl_3$) spectrum for 3n

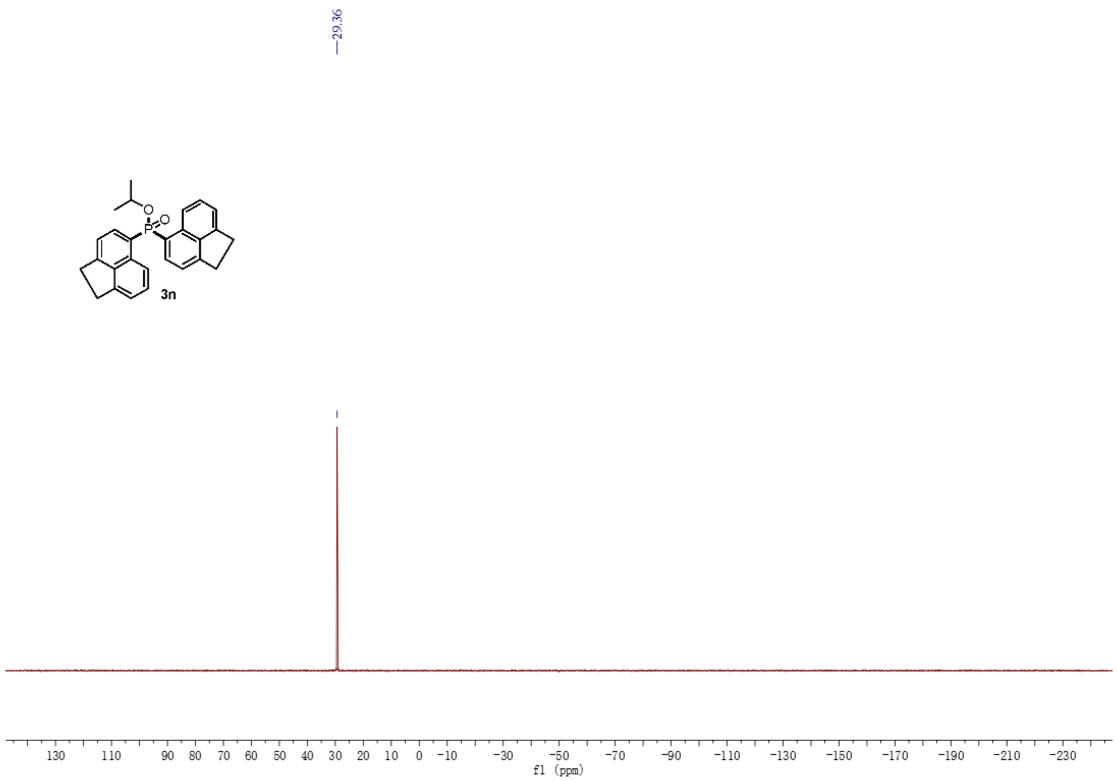

^{1}H NMR (400 MHz, $CDCl_3$) spectrum for 3o

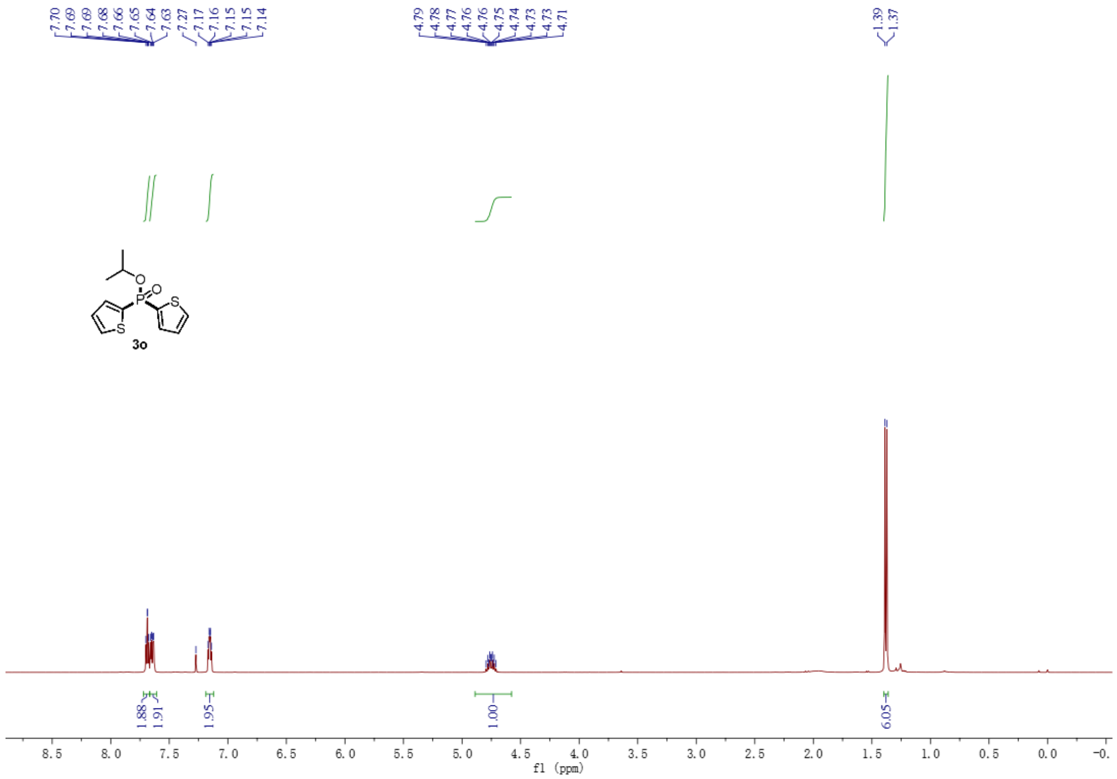

^{13}C NMR (101 MHz, $CDCl_3$) spectrum for 3o

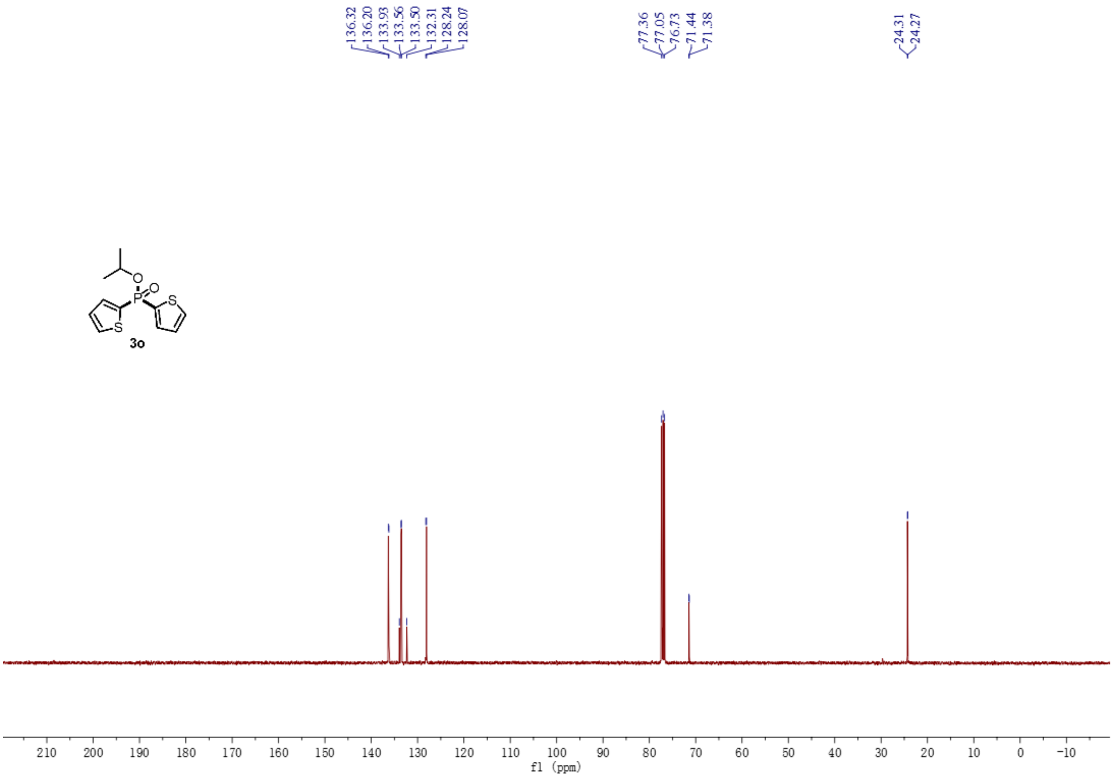

^{31}P NMR (121 MHz, $CDCl_3$) spectrum for 3o

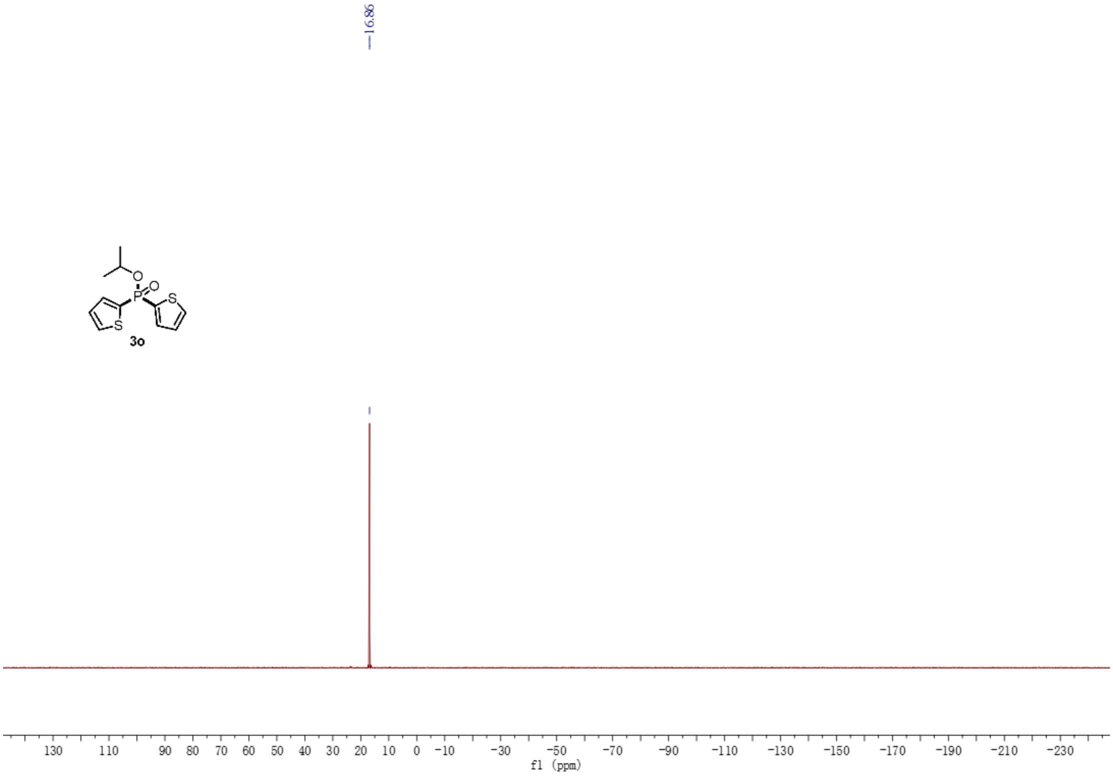

^{1}H NMR (400 MHz, $CDCl_3$) spectrum for 3p

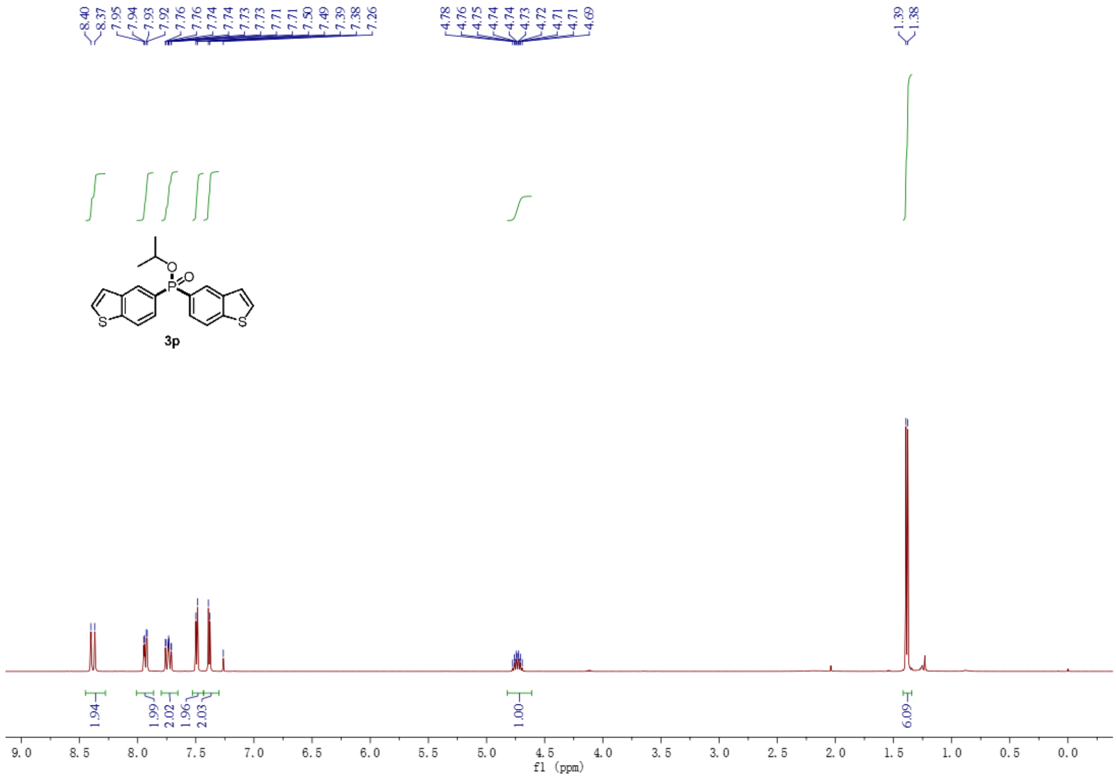

^{13}C NMR (101 MHz, $CDCl_3$) spectrum for 3p

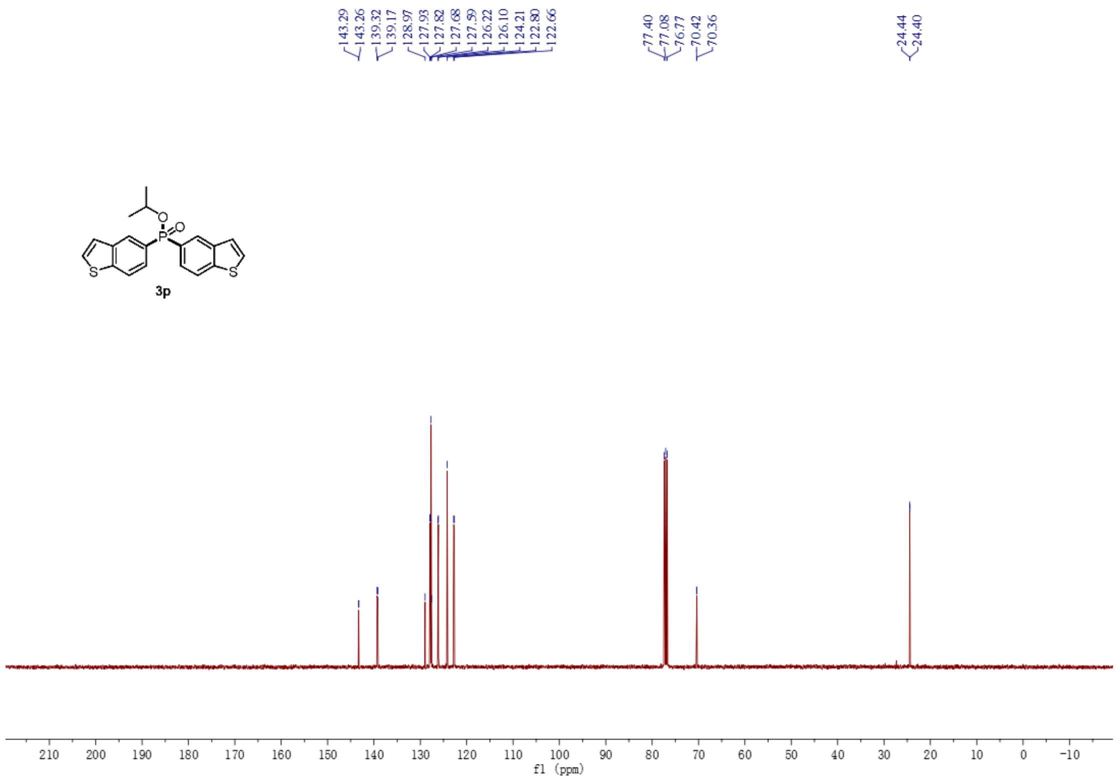

^{31}P NMR (121 MHz, CDCl$_3$) spectrum for 3p

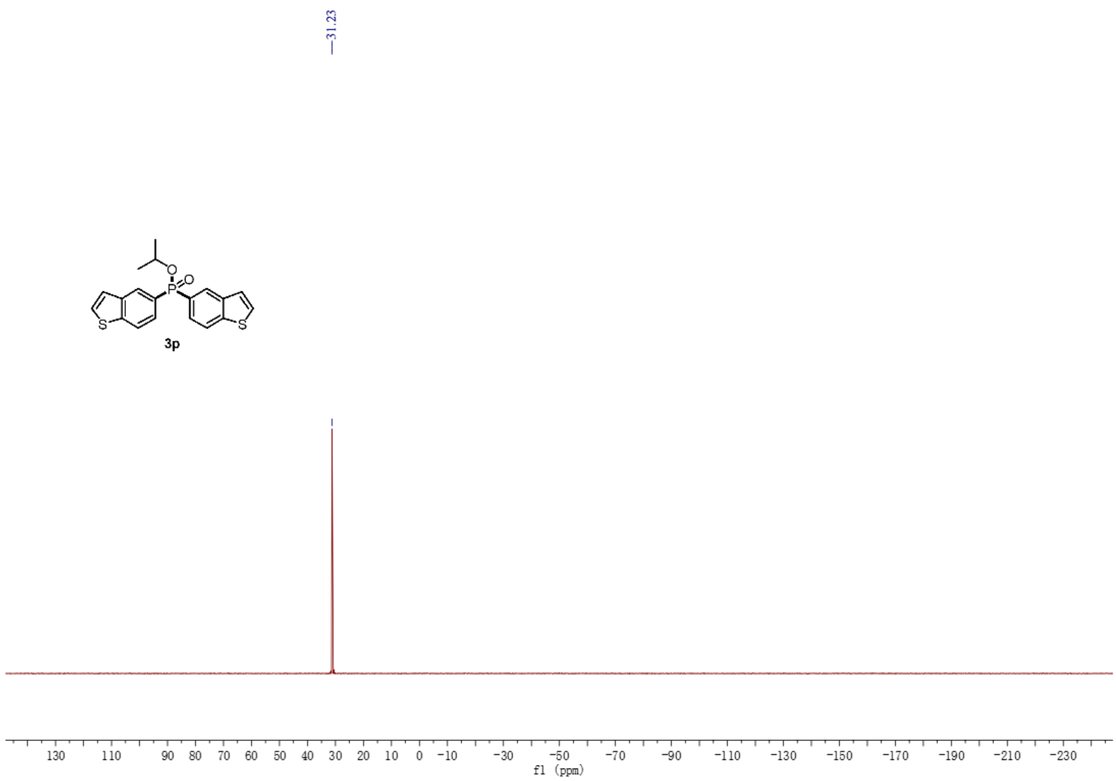

^{1}H NMR (400 MHz, CDCl$_3$) spectrum for 3q

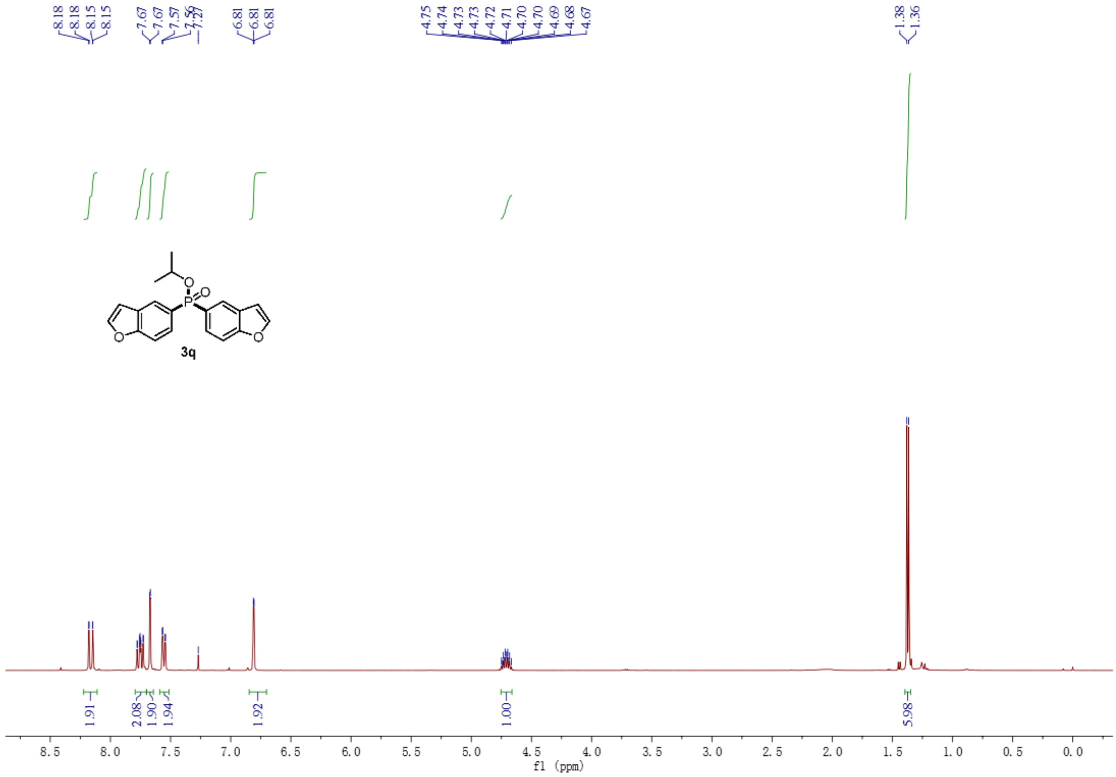

^{13}C NMR (101 MHz, $CDCl_3$) spectrum for 3q

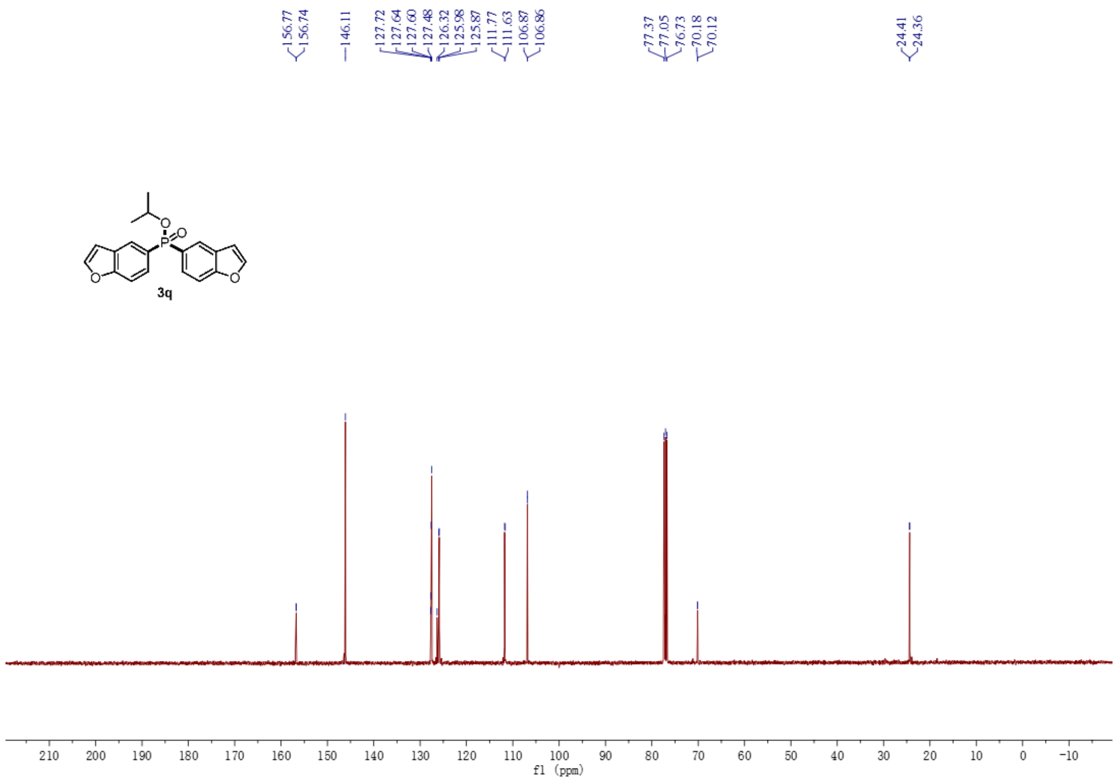

^{31}P NMR (121 MHz, $CDCl_3$) spectrum for 3q

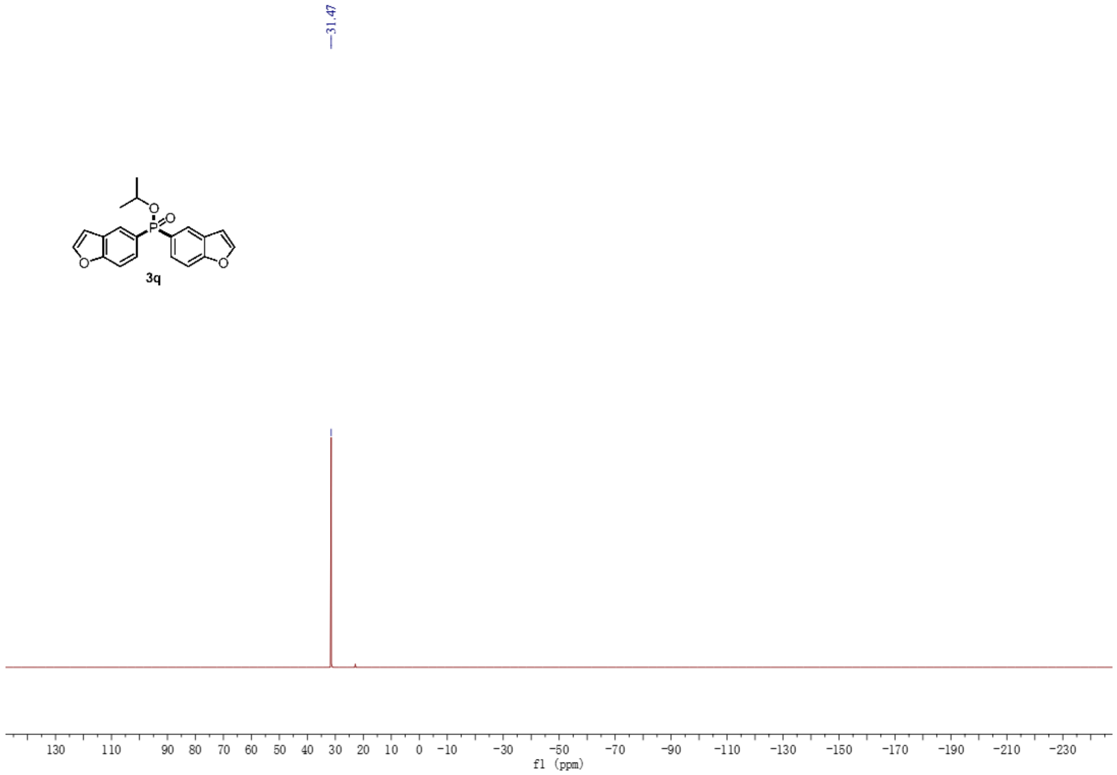

^{1}H NMR (400 MHz, $CDCl_3$) spectrum for 3r

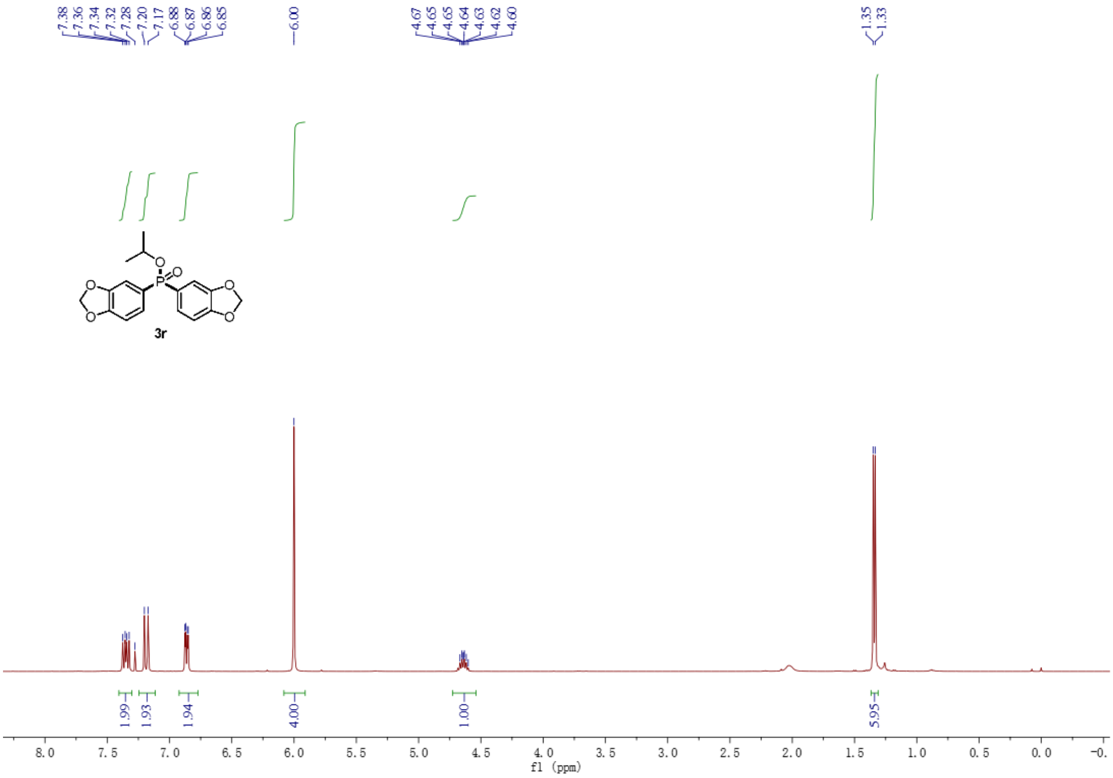

^{13}C NMR (101 MHz, $CDCl_3$) spectrum for 3r

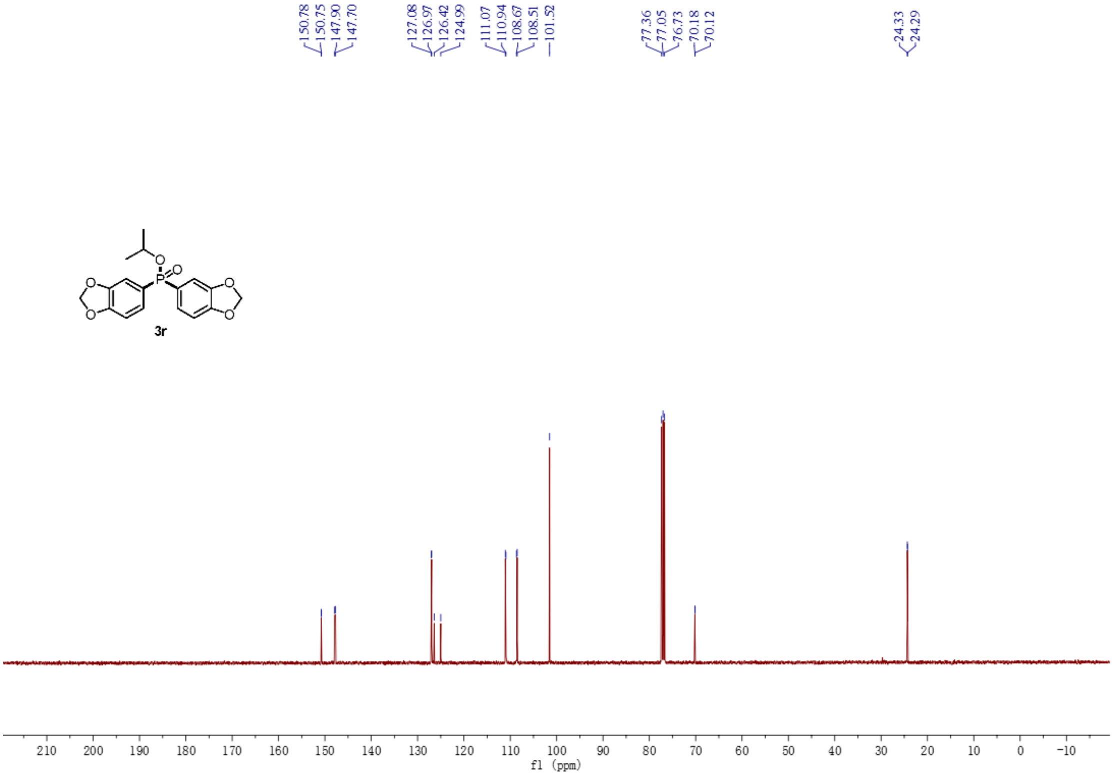

^{31}P NMR (121 MHz, $CDCl_3$) spectrum for 3r

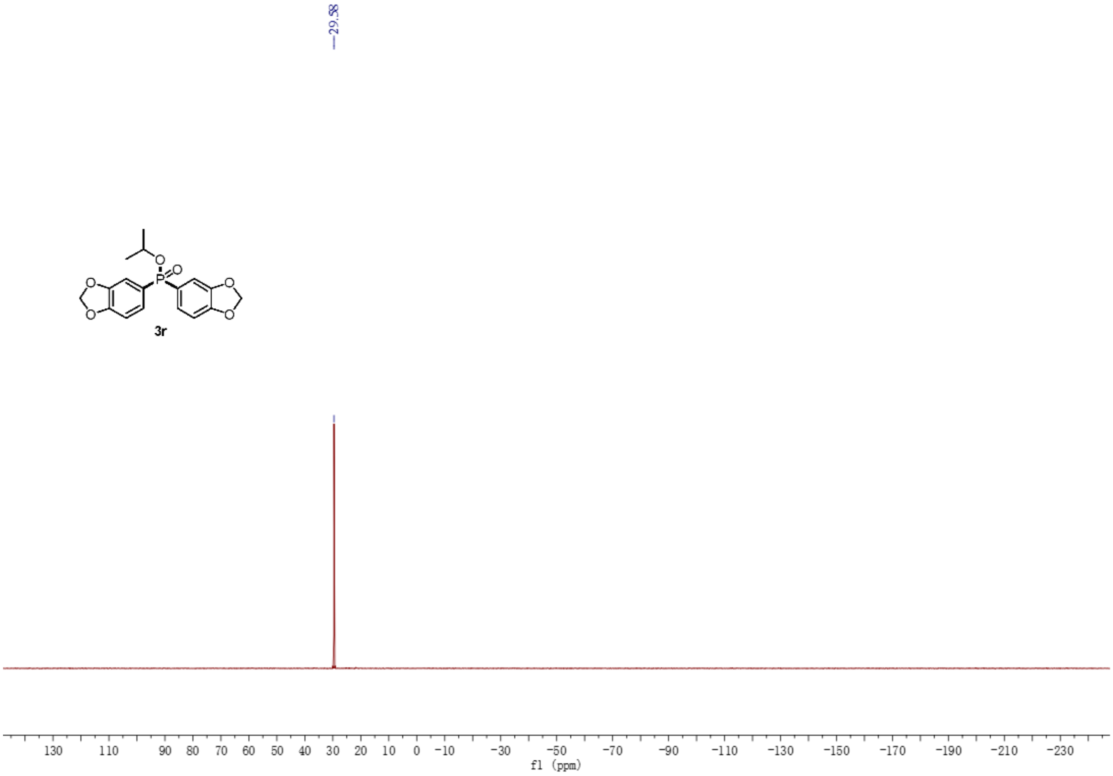

^{1}H NMR (400 MHz, $CDCl_3$) spectrum for 3s

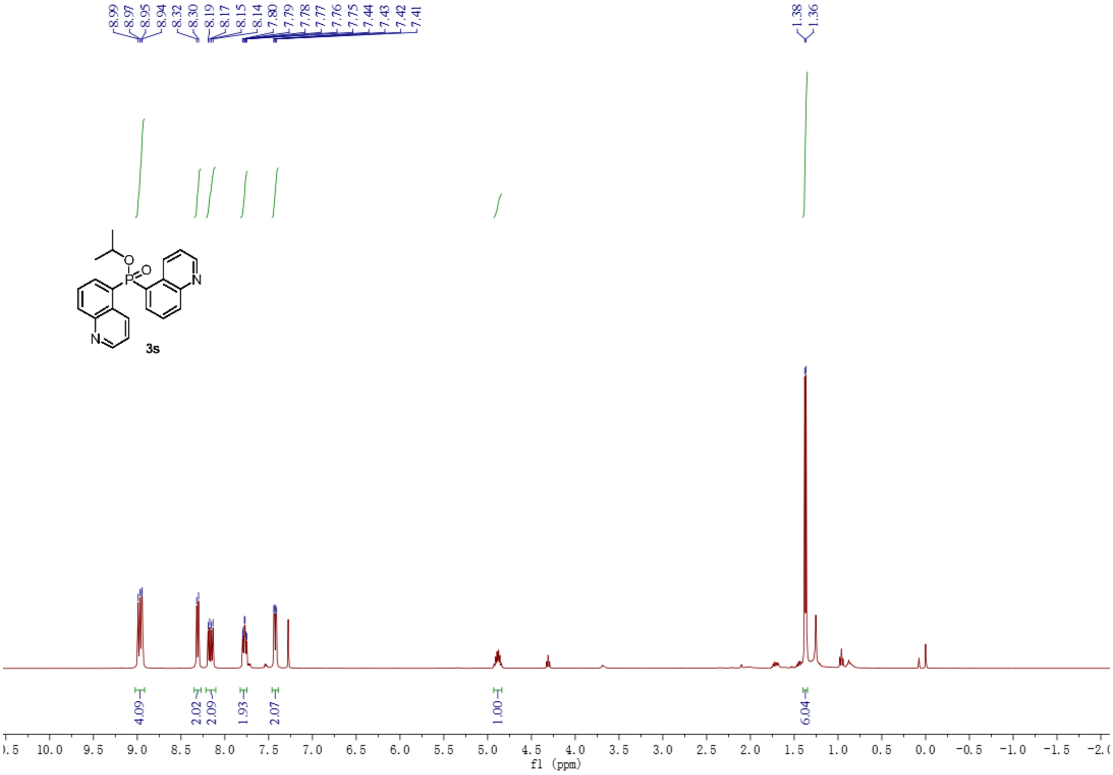

^{13}C NMR (101 MHz, $CDCl_3$) spectrum for 3s

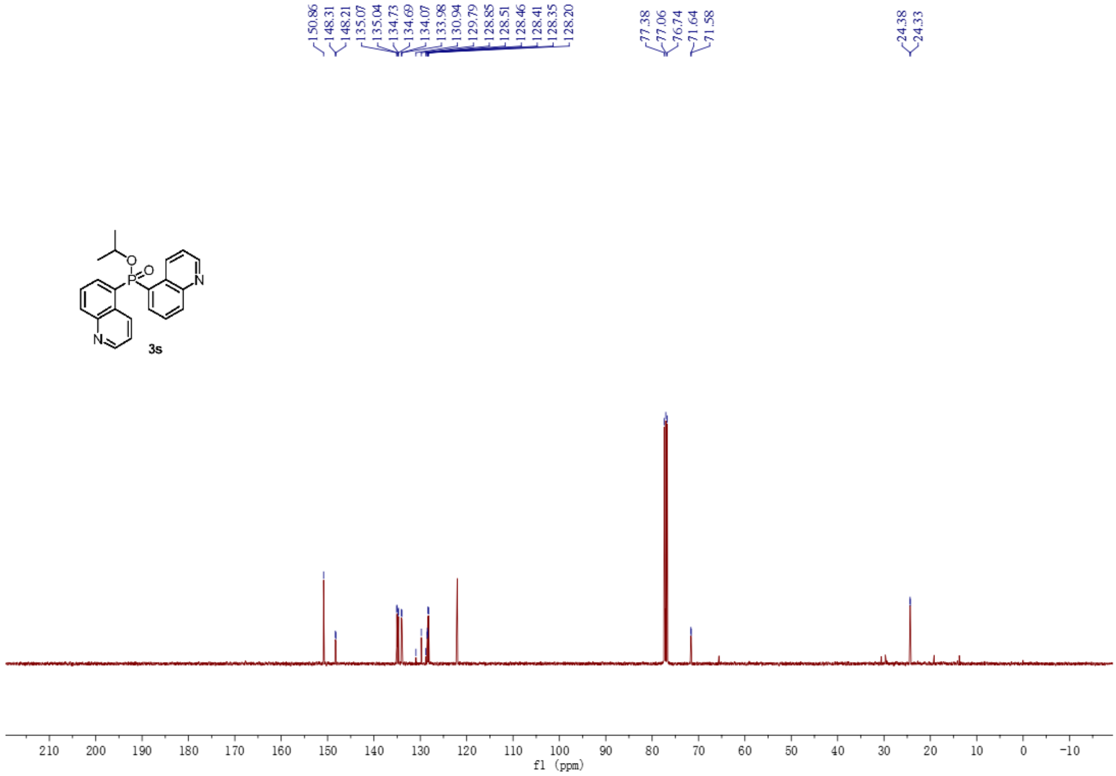

^{31}P NMR (121 MHz, $CDCl_3$) spectrum for 3s

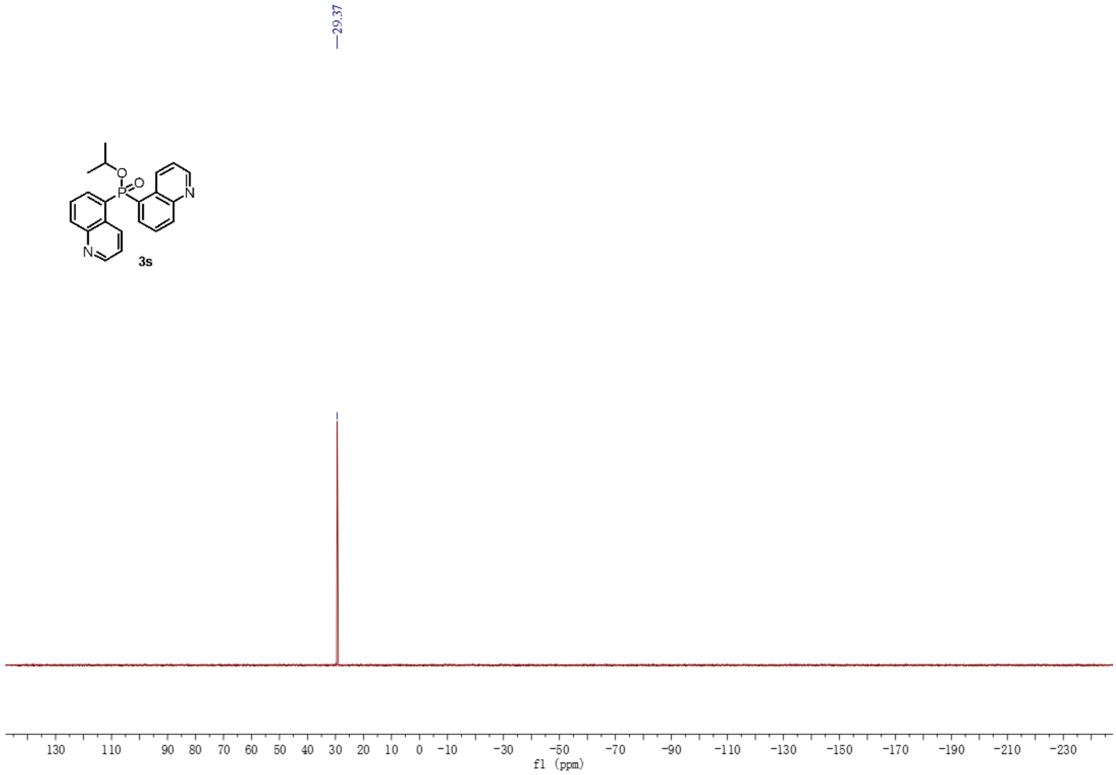

^{1}H NMR (400 MHz, $CDCl_3$) spectrum for 3t

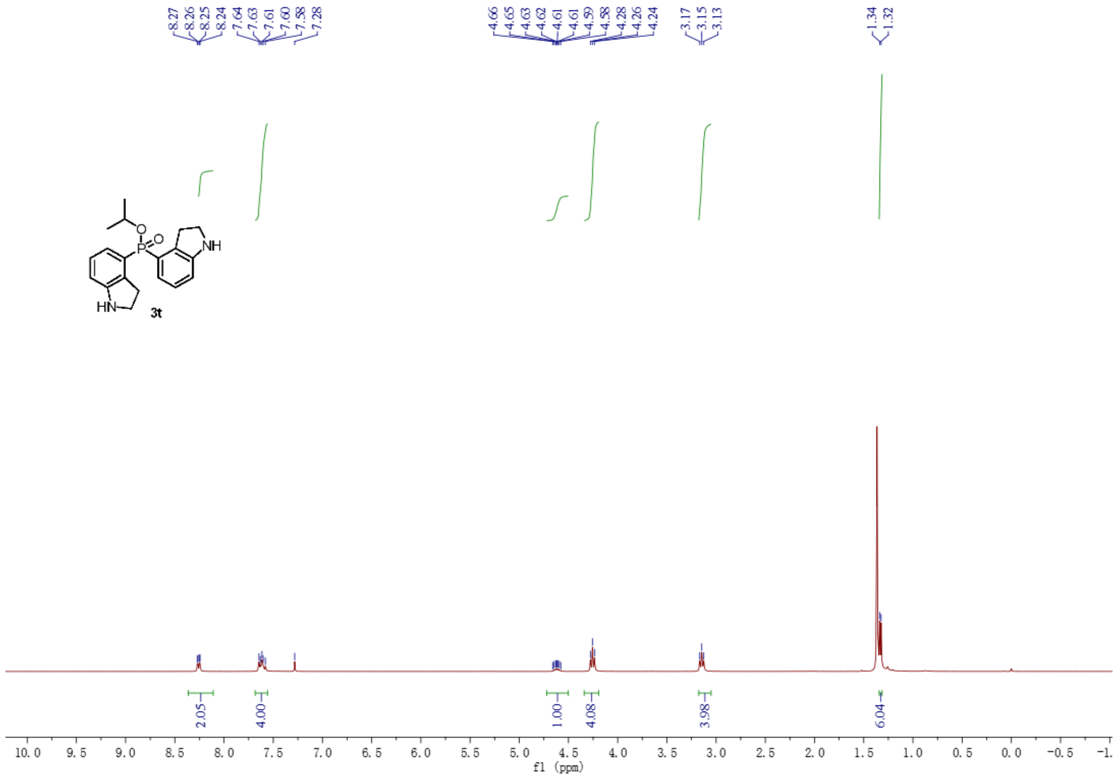

^{13}C NMR (101 MHz, $CDCl_3$) spectrum for 3t

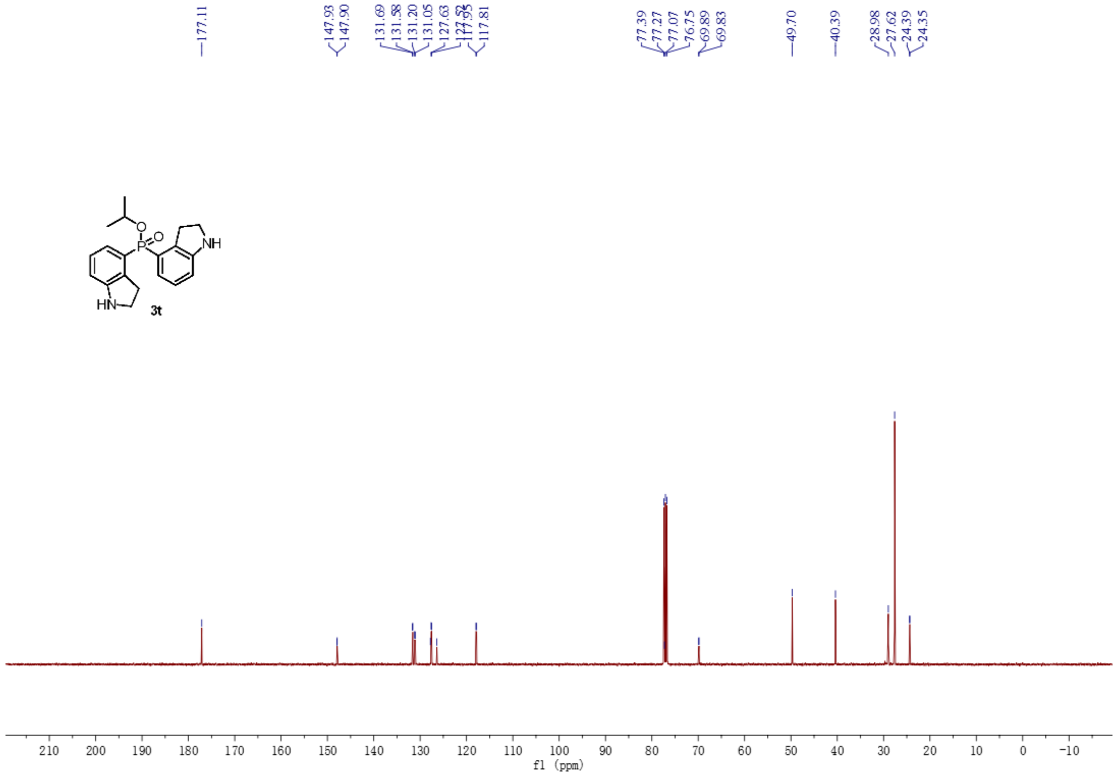

^{31}P NMR (121 MHz, CDCl$_3$) spectrum for 3t

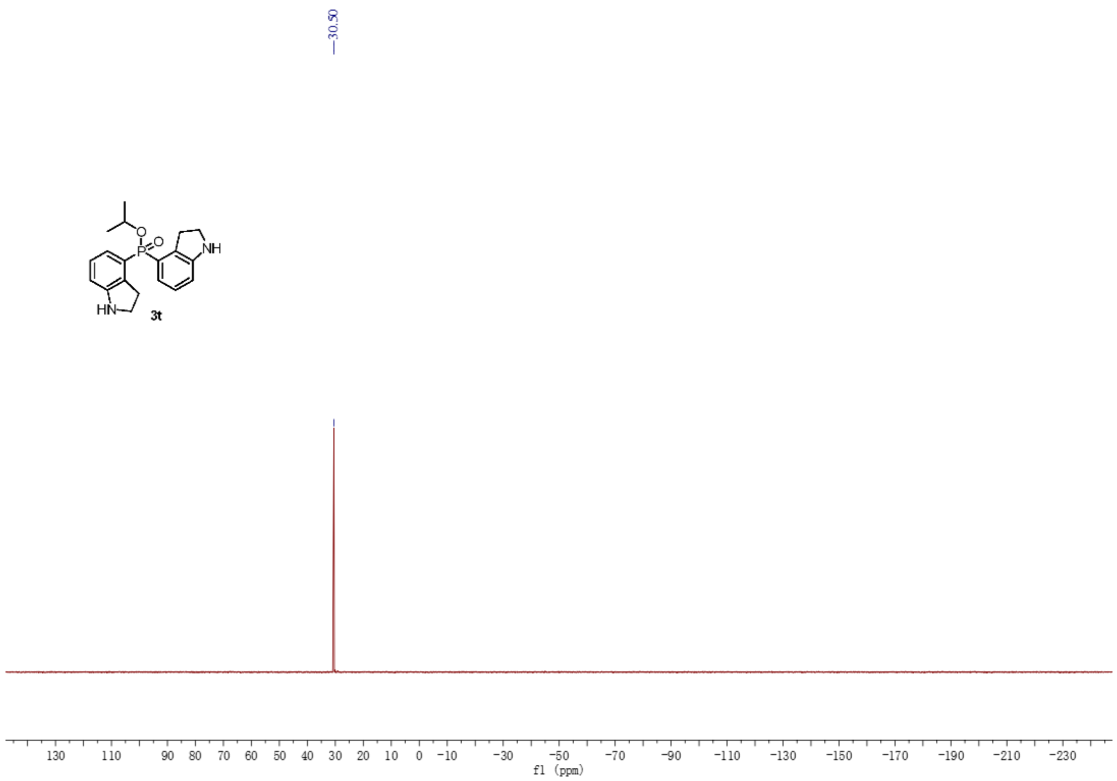

^{1}H NMR (400 MHz, CDCl$_3$) spectrum for 3u

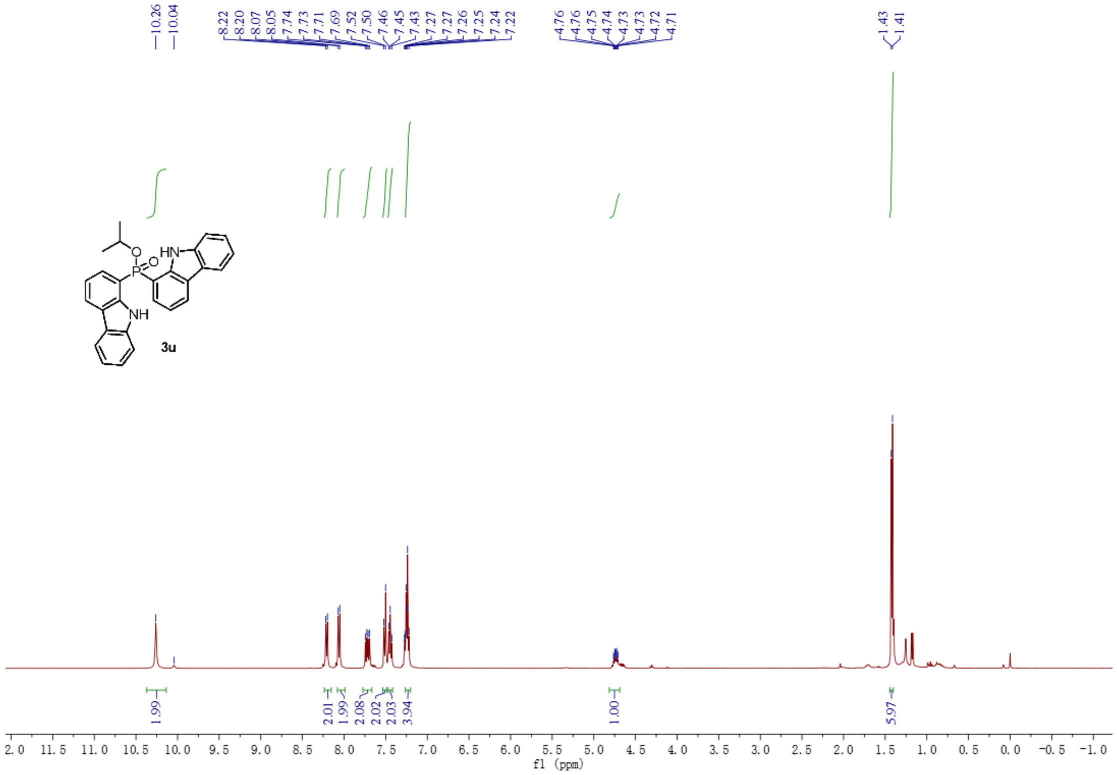

^{13}C NMR (101 MHz, $CDCl_3$) spectrum for 3u

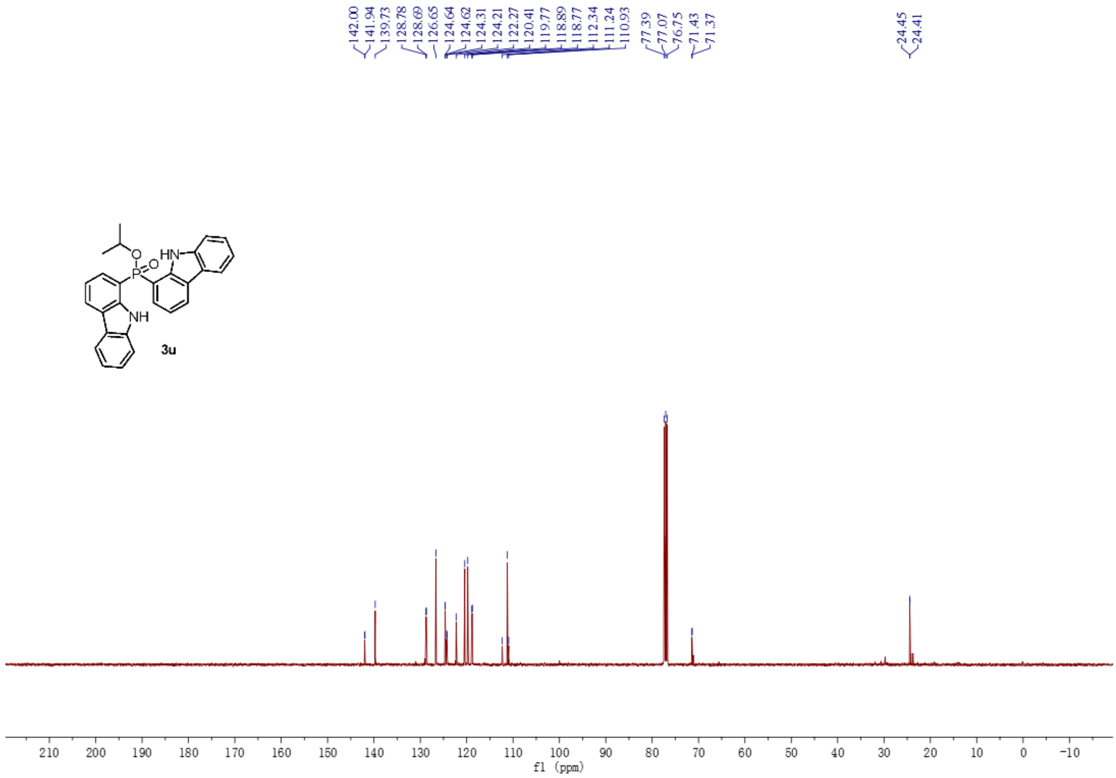

^{31}P NMR (121 MHz, $CDCl_3$) spectrum for 3u

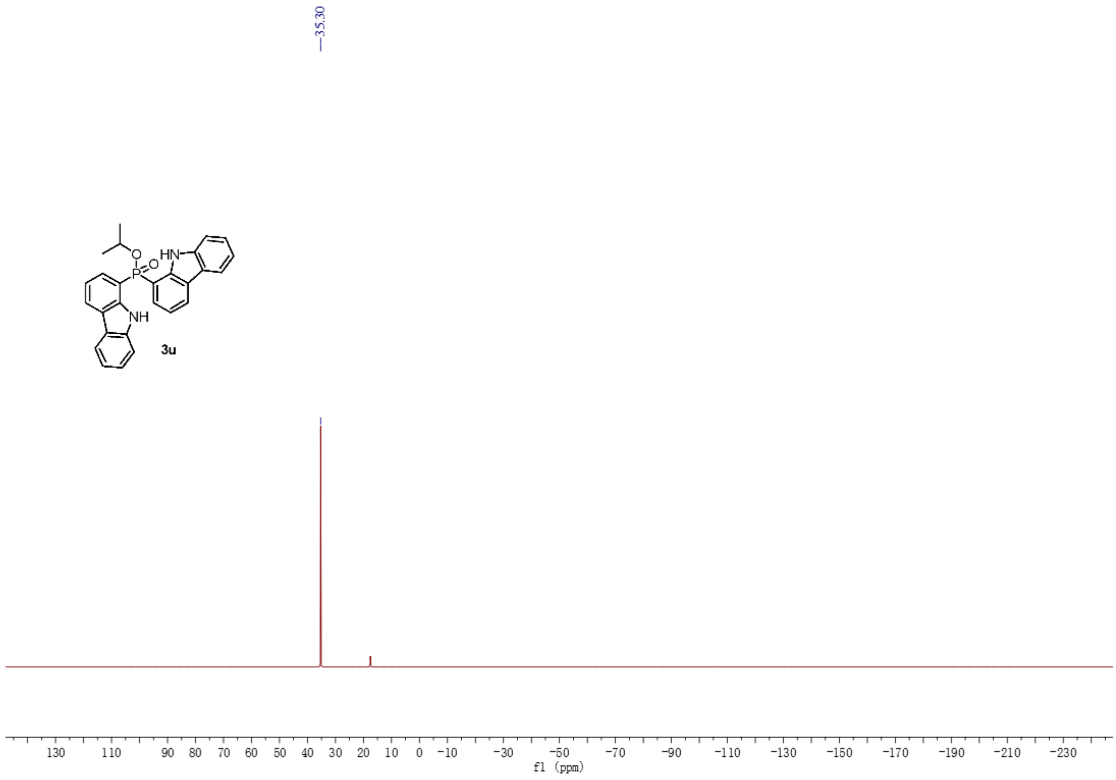

^{1}H NMR (400 MHz, CDCl3) spectrum for 3v

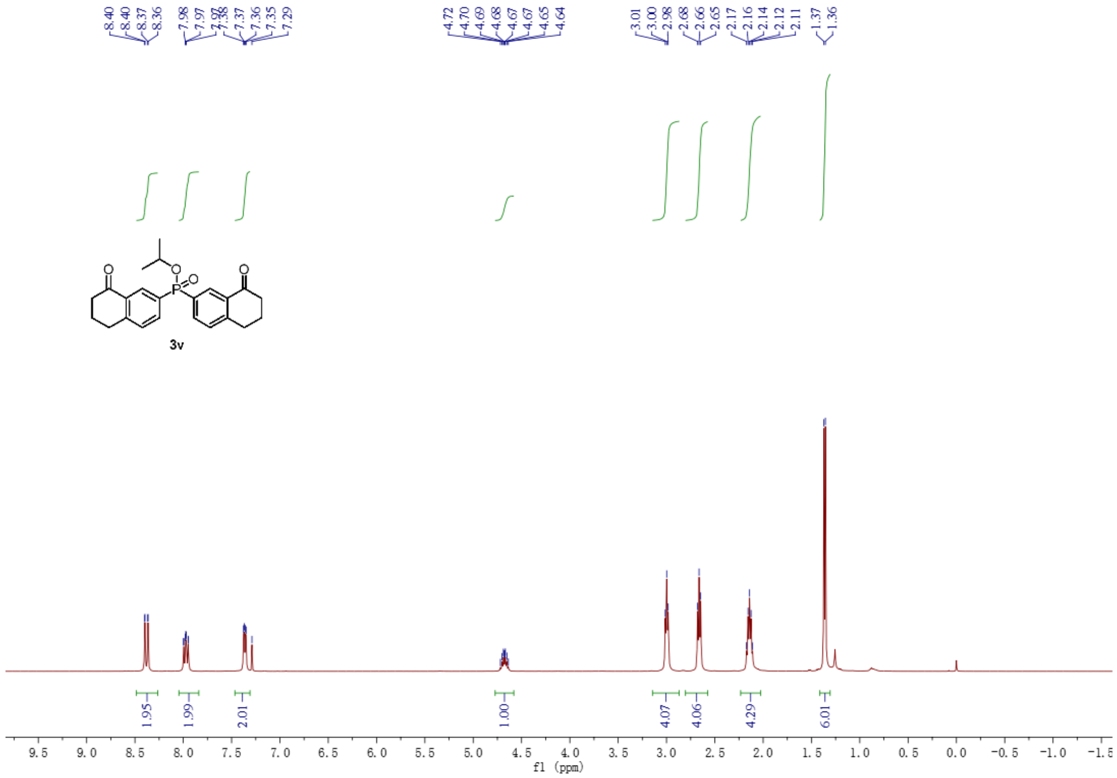

^{13}C NMR (101 MHz, CDCl3) spectrum for 3v

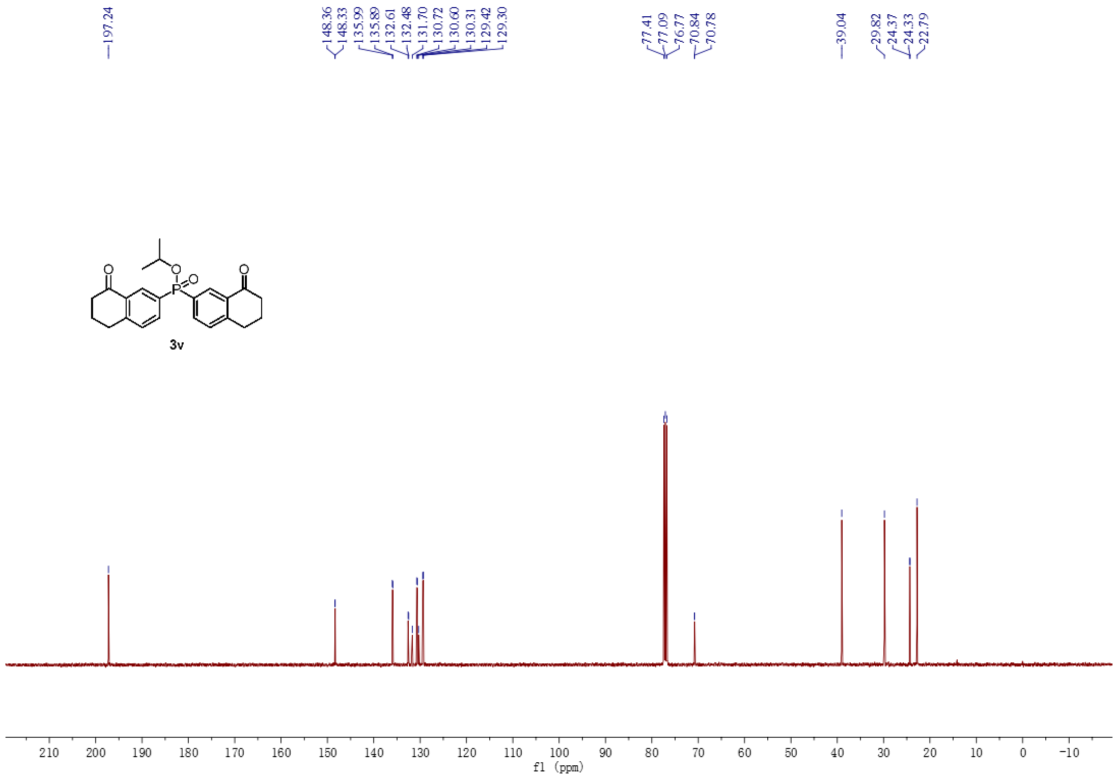

^{31}P NMR (121 MHz, $CDCl_3$) spectrum for 3v

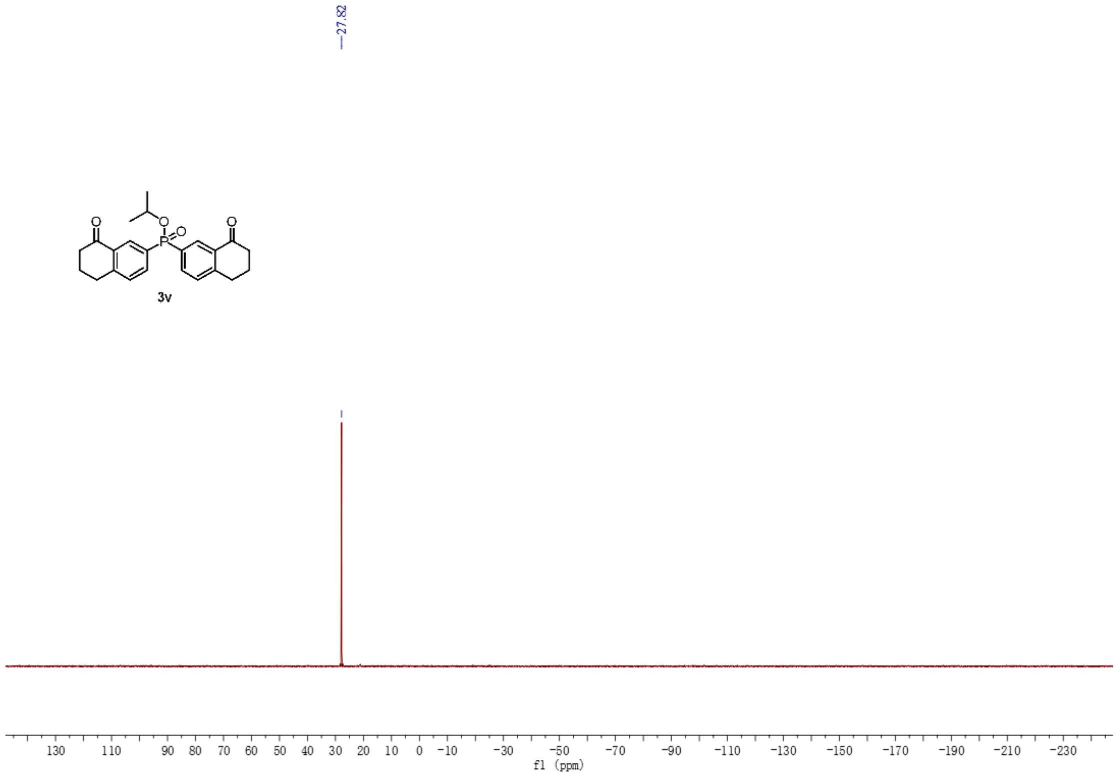

^{1}H NMR (400 MHz, $CDCl_3$) spectrum for 3w

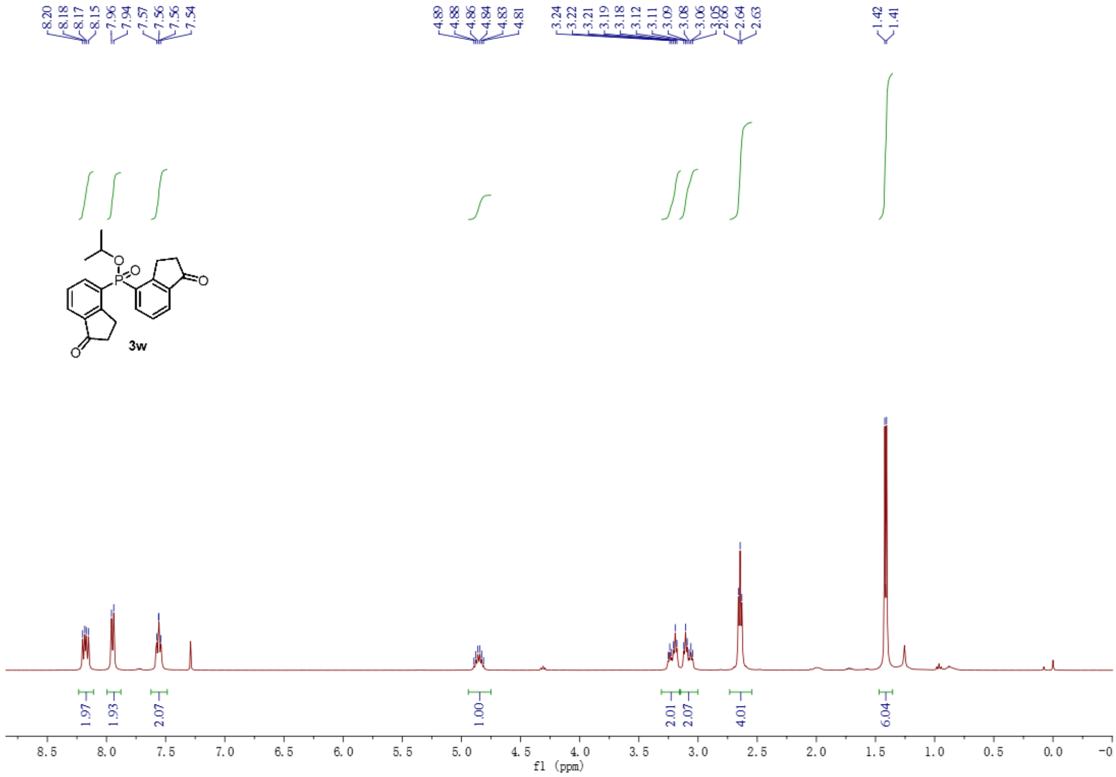

^{13}C NMR (101 MHz, $CDCl_3$) spectrum for 3w

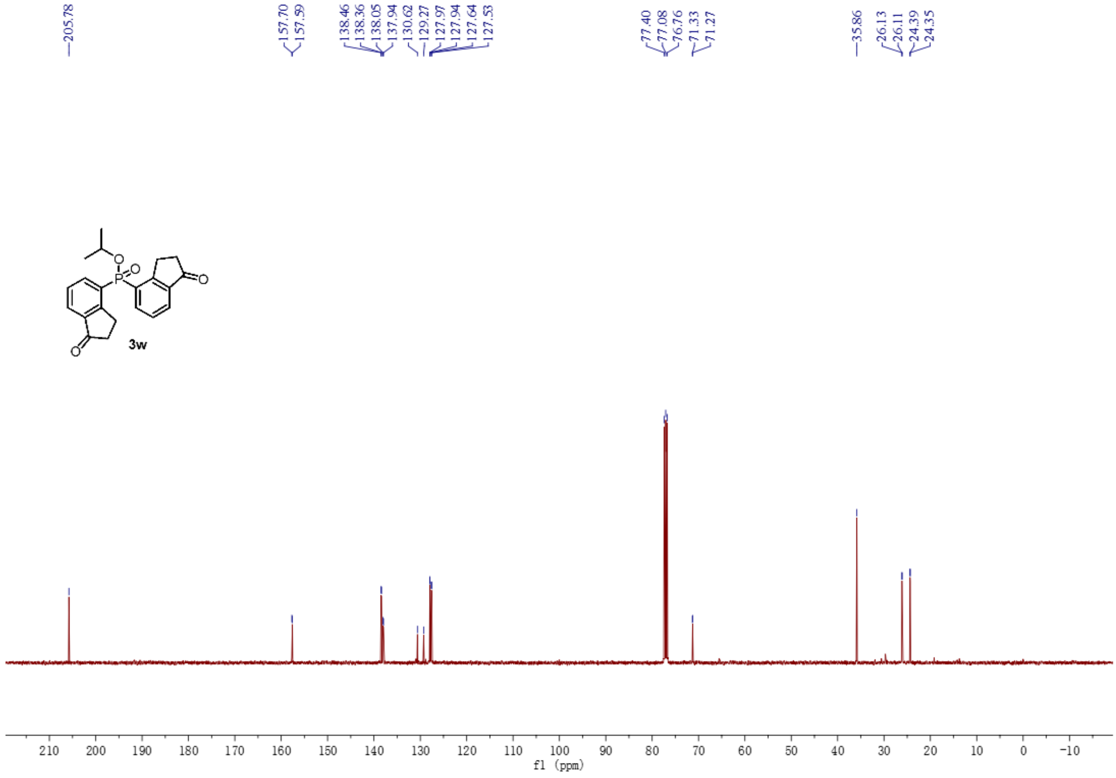

^{31}P NMR (121 MHz, $CDCl_3$) spectrum for 3w

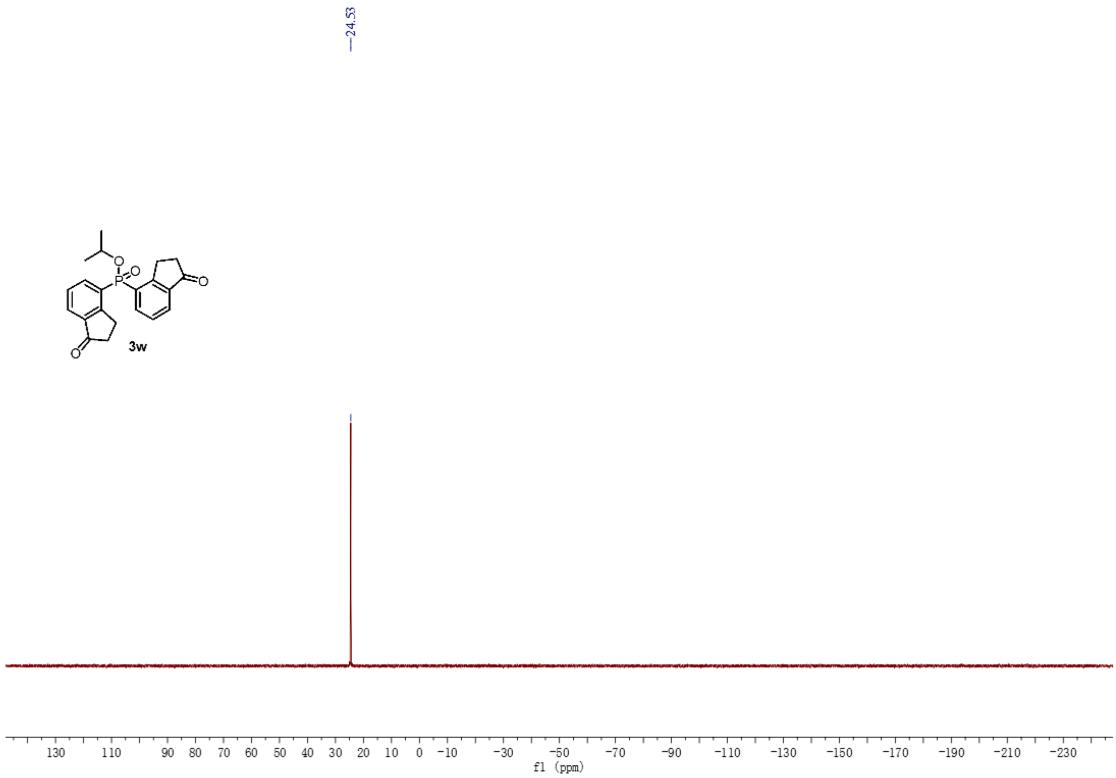

^{1}H NMR (400 MHz, $CDCl_3$) spectrum for 4a

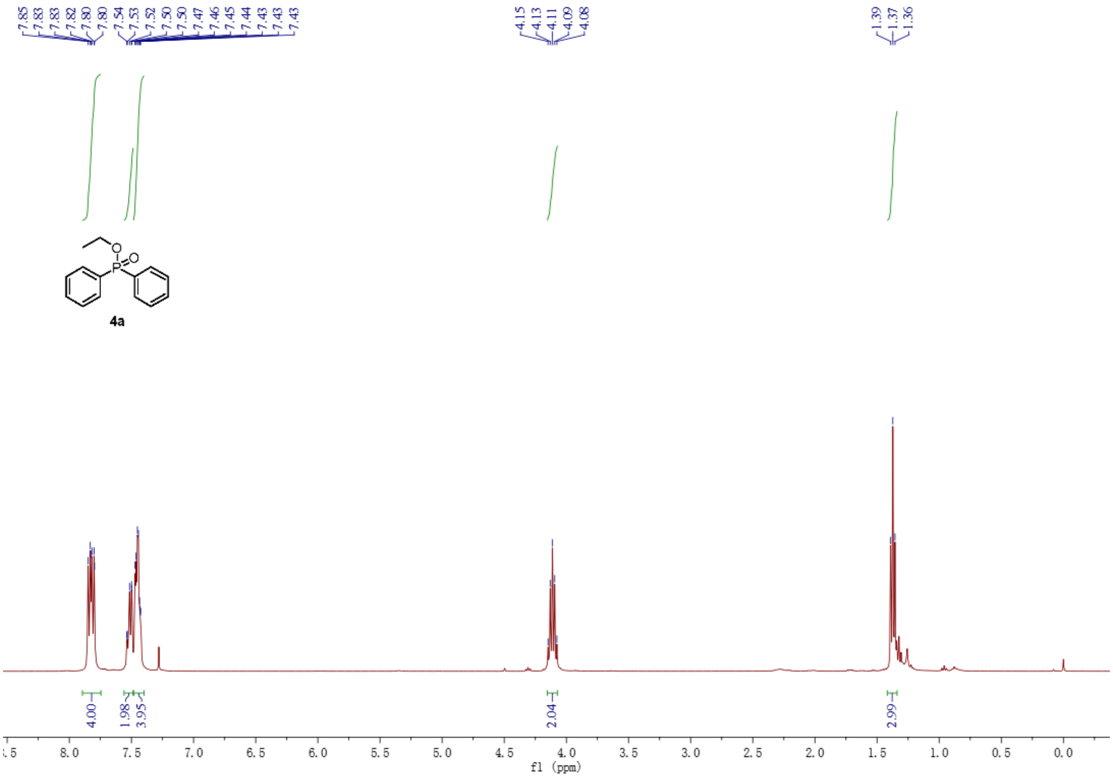

^{13}C NMR (101 MHz, $CDCl_3$) spectrum for 4a

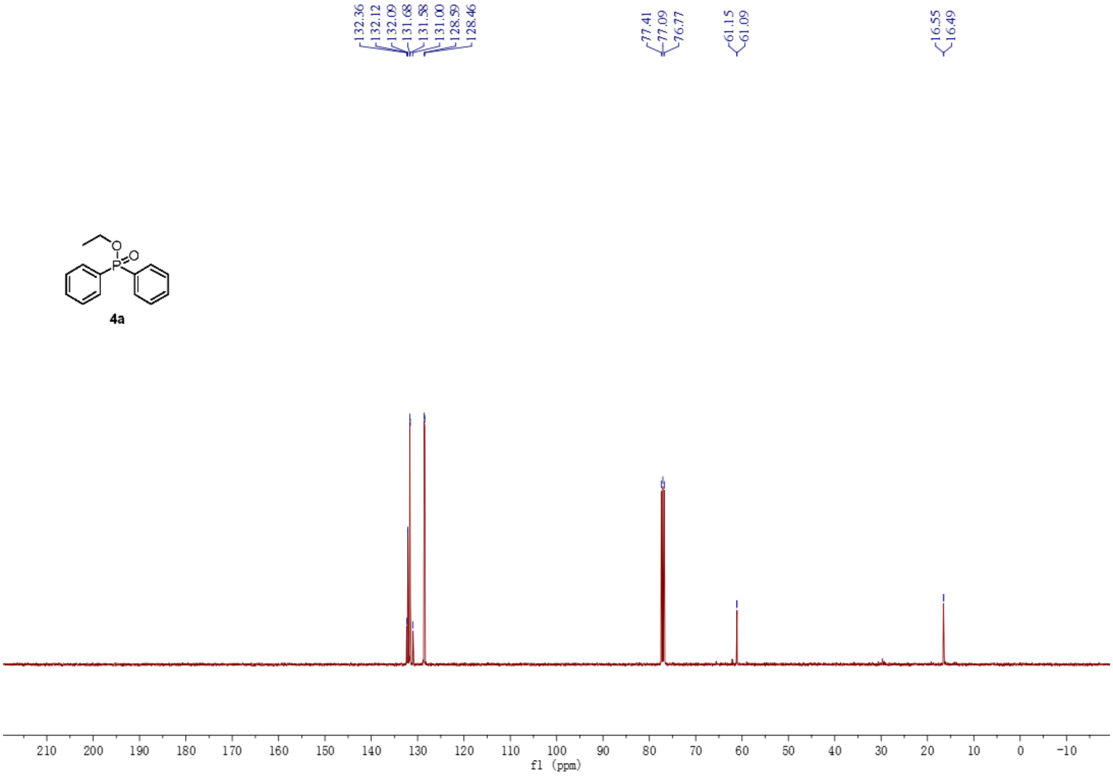

^{31}P NMR (121 MHz, $CDCl_3$) spectrum for 4a

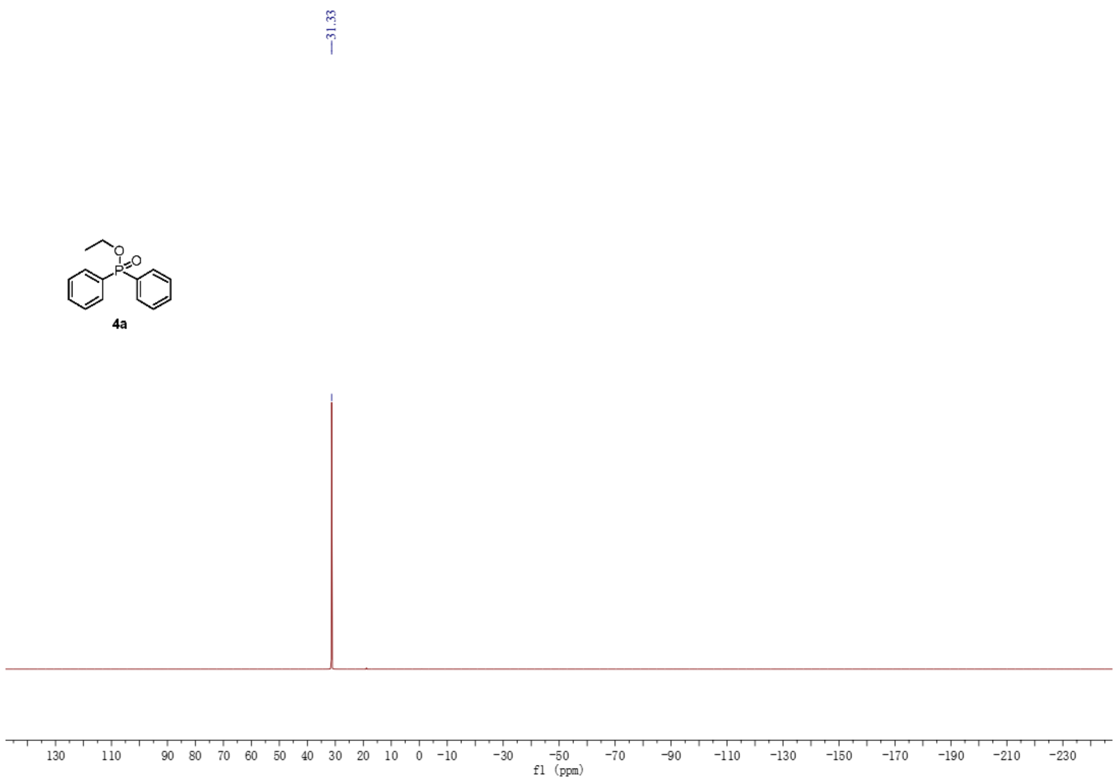

^{1}H NMR (400 MHz, $CDCl_3$) spectrum for 4b

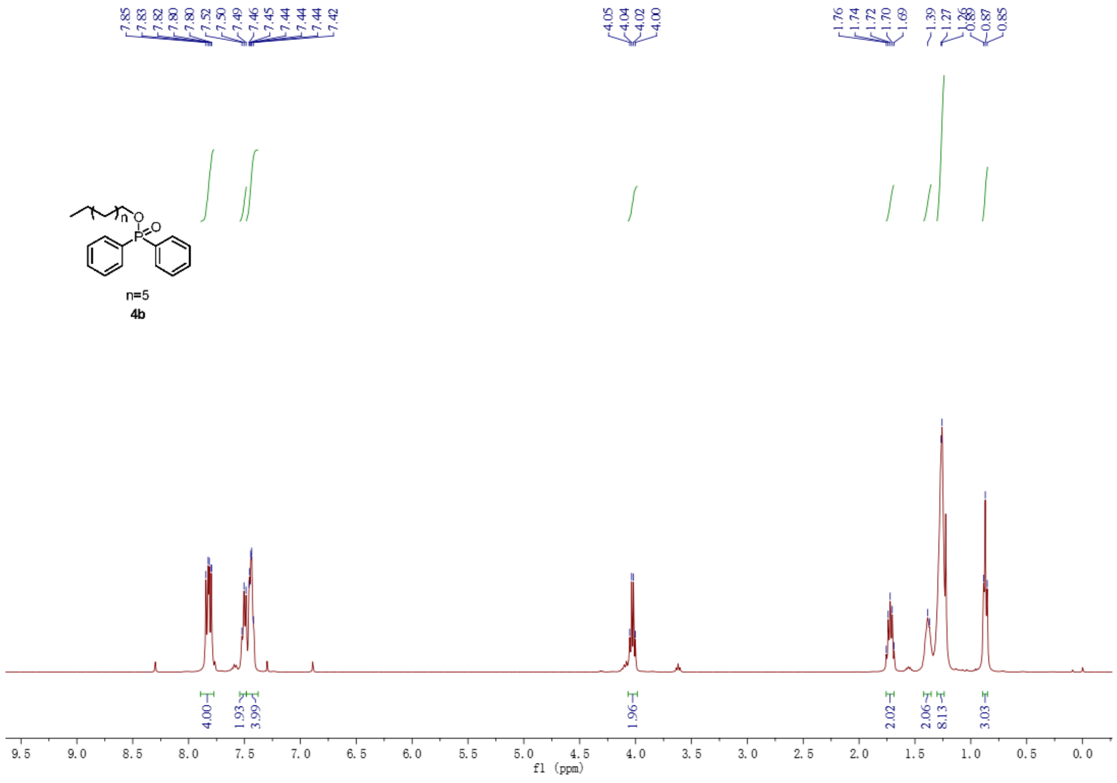

^{13}C NMR (101 MHz, $CDCl_3$) spectrum for 4b

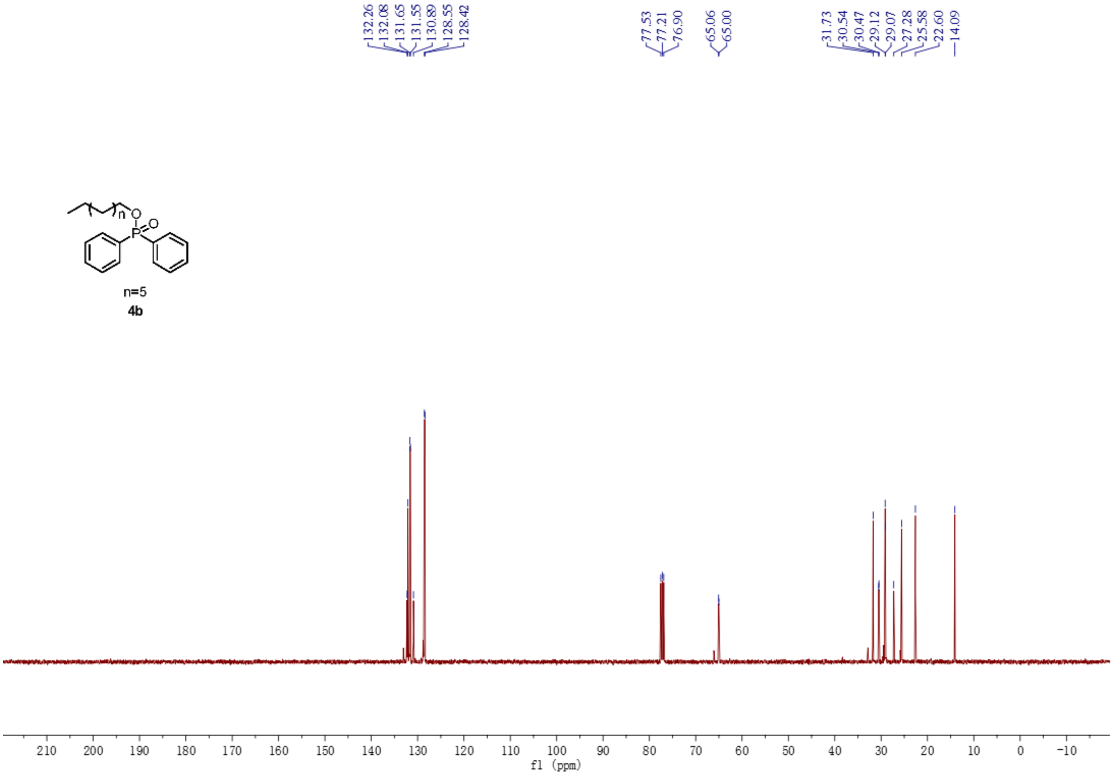

^{31}P NMR (121 MHz, $CDCl_3$) spectrum for 4b

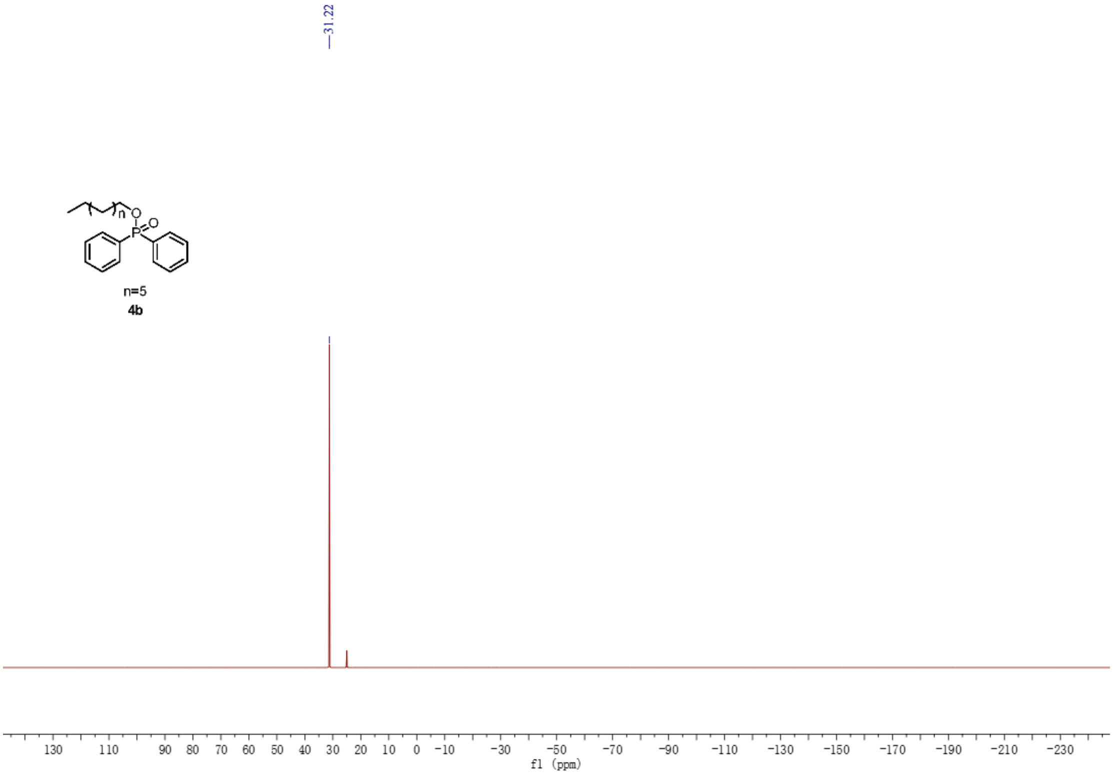

^{1}H NMR (400 MHz, $CDCl_3$) spectrum for 4c

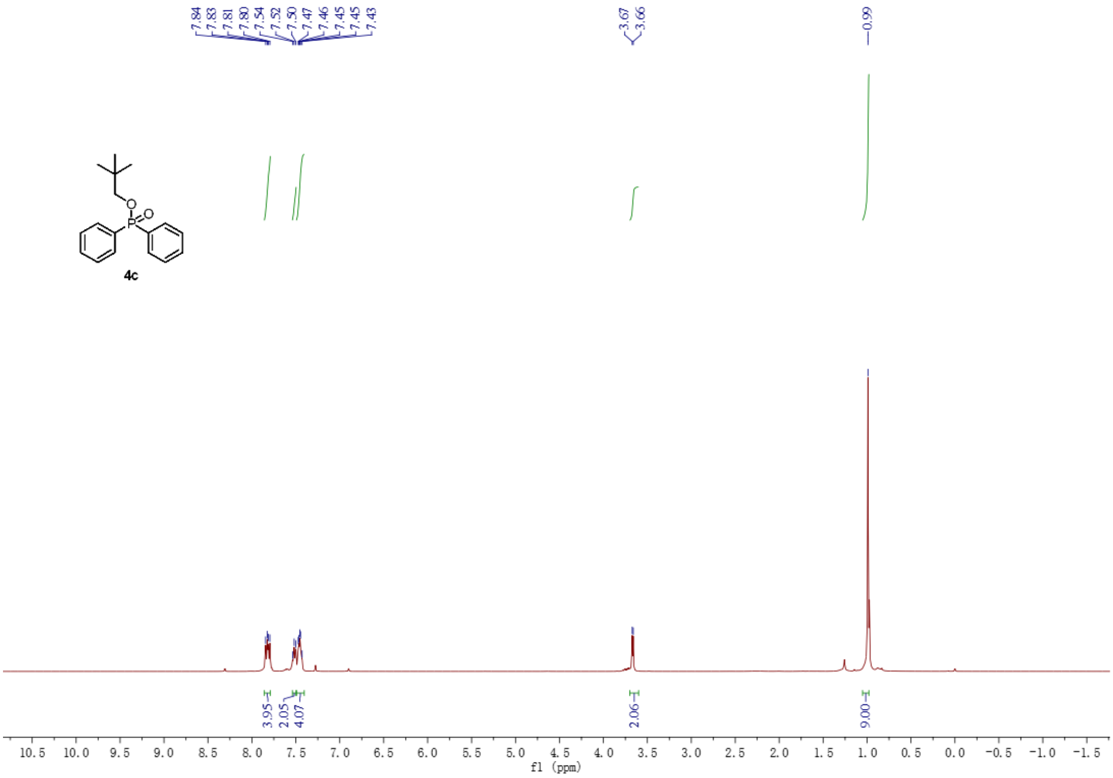

^{13}C NMR (101 MHz, $CDCl_3$) spectrum for 4c

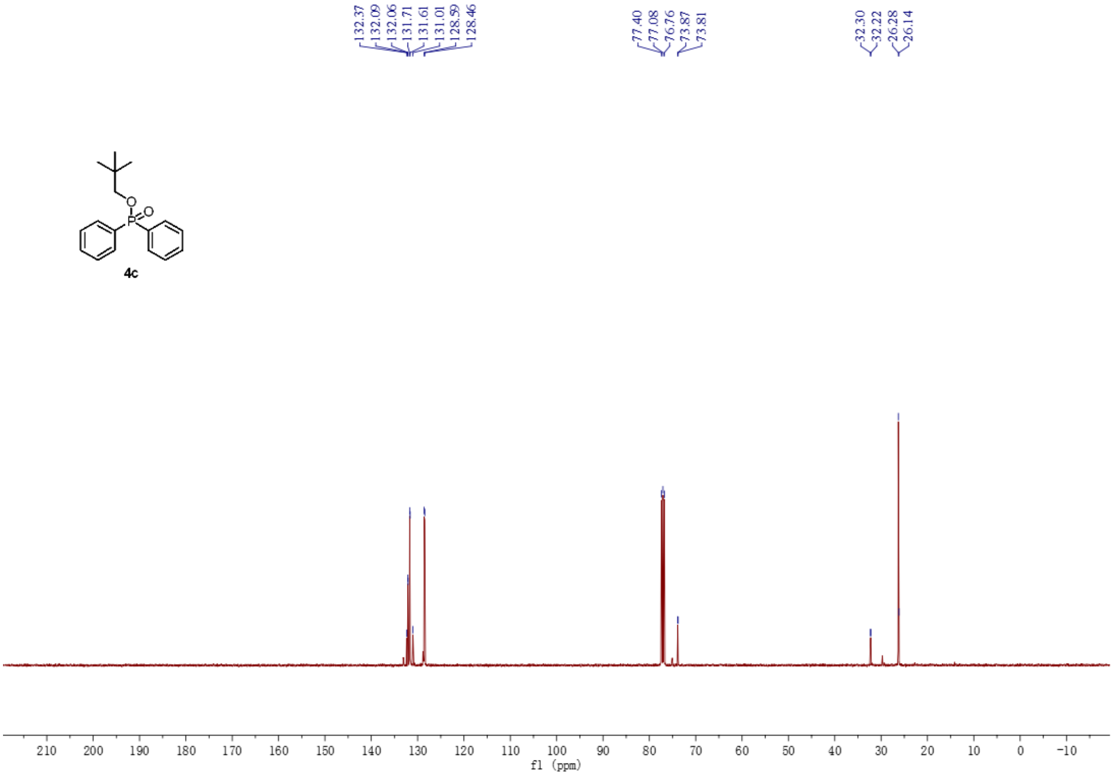

^{31}P NMR (121 MHz, $CDCl_3$) spectrum for 4c

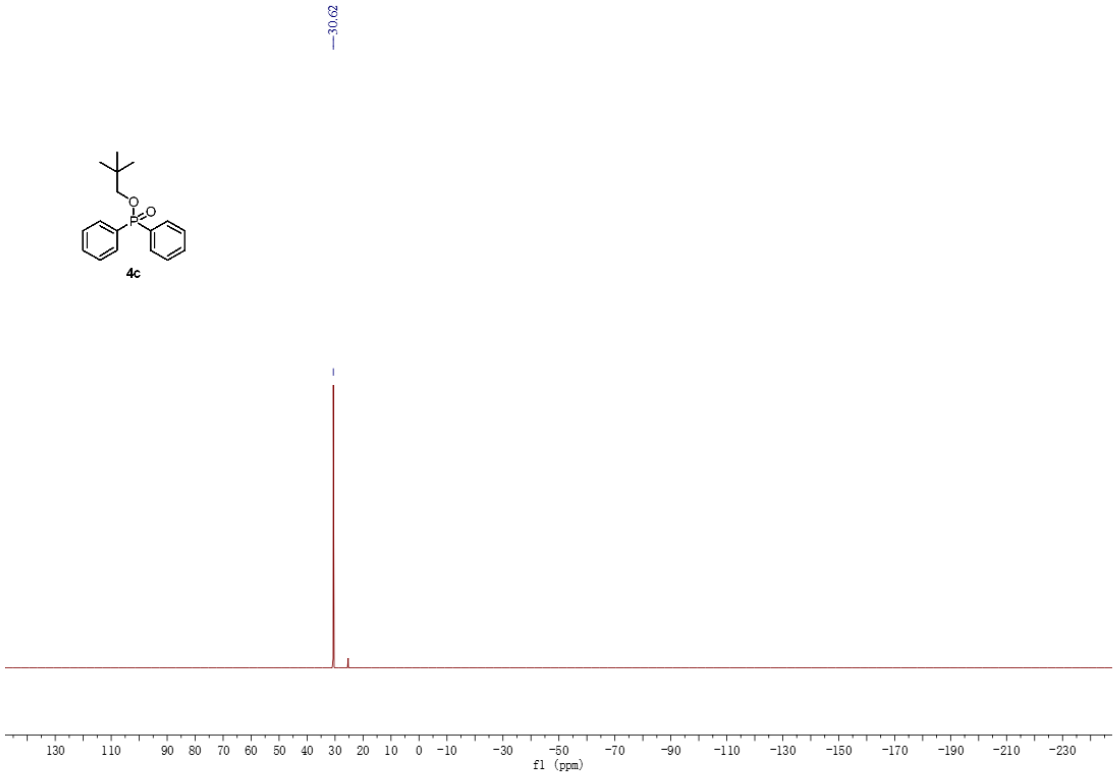

^{1}H NMR (400 MHz, $CDCl_3$) spectrum for 4d

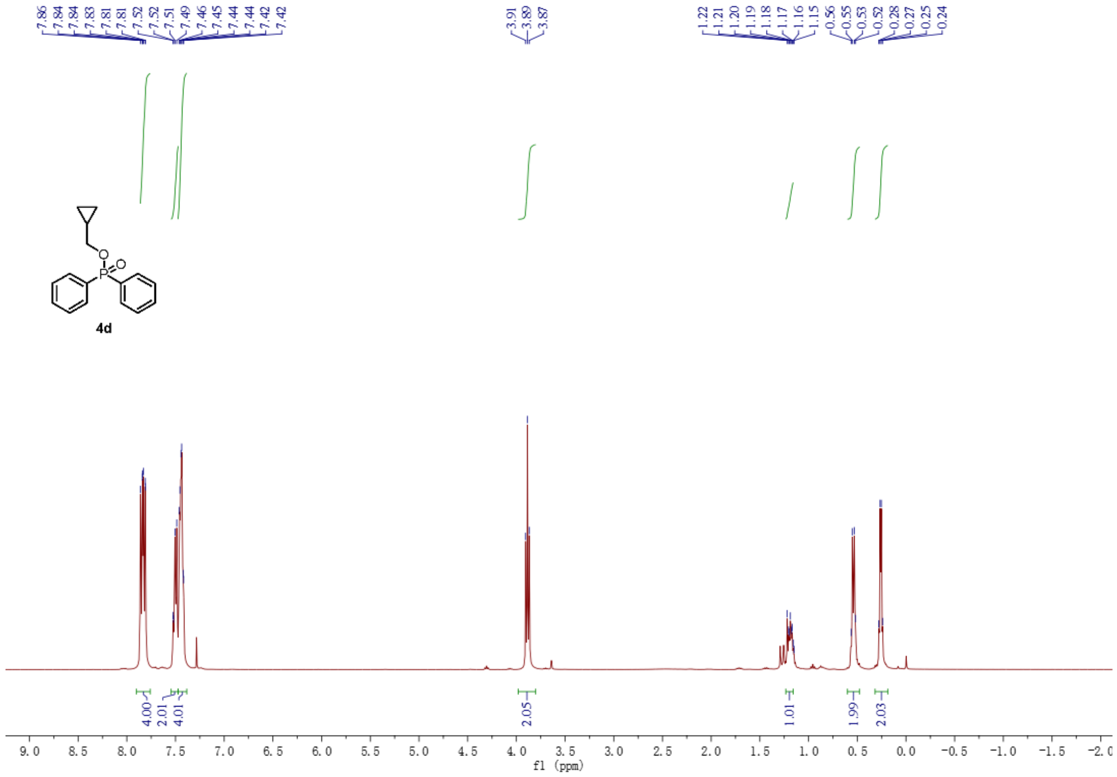

^{13}C NMR (101 MHz, $CDCl_3$) spectrum for 4d

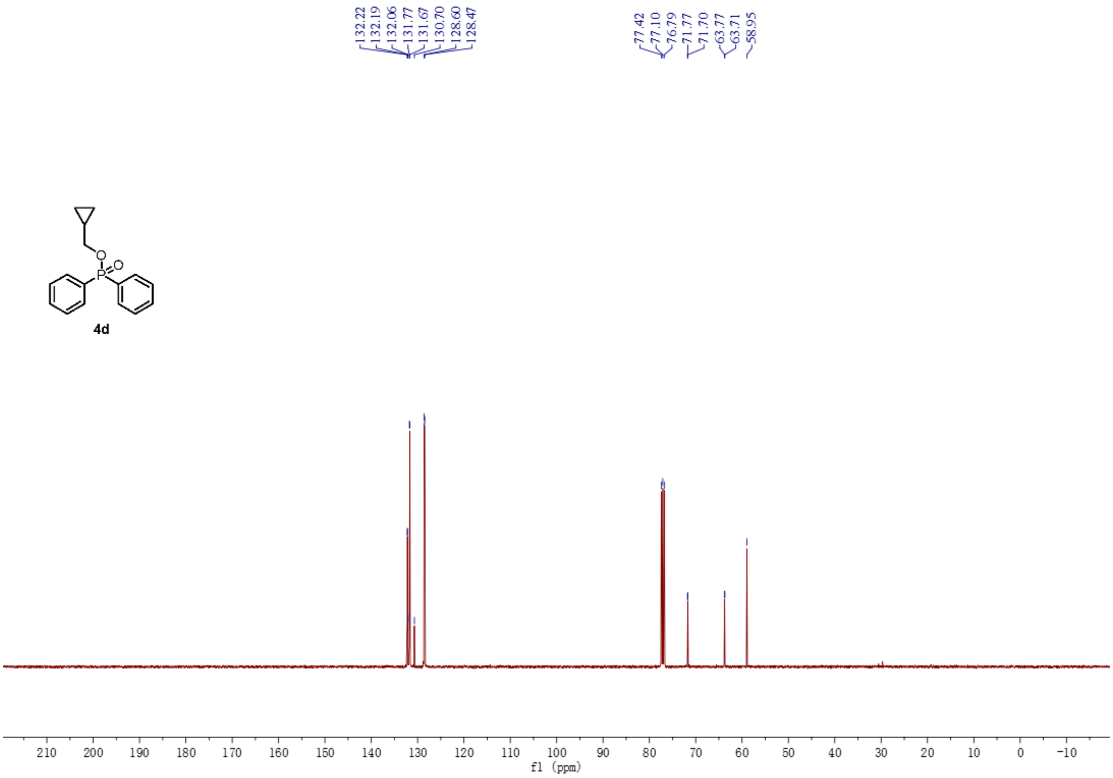

^{31}P NMR (121 MHz, $CDCl_3$) spectrum for 4d

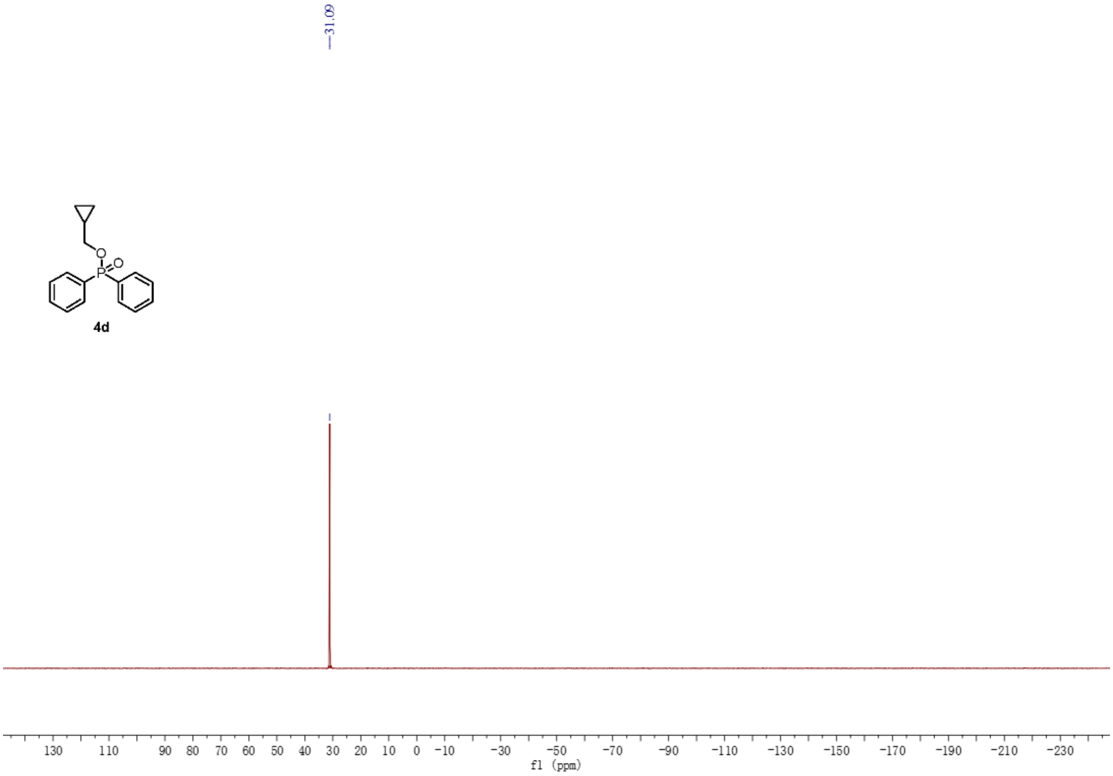

^1H NMR (400 MHz, $CDCl_3$) spectrum for 4e

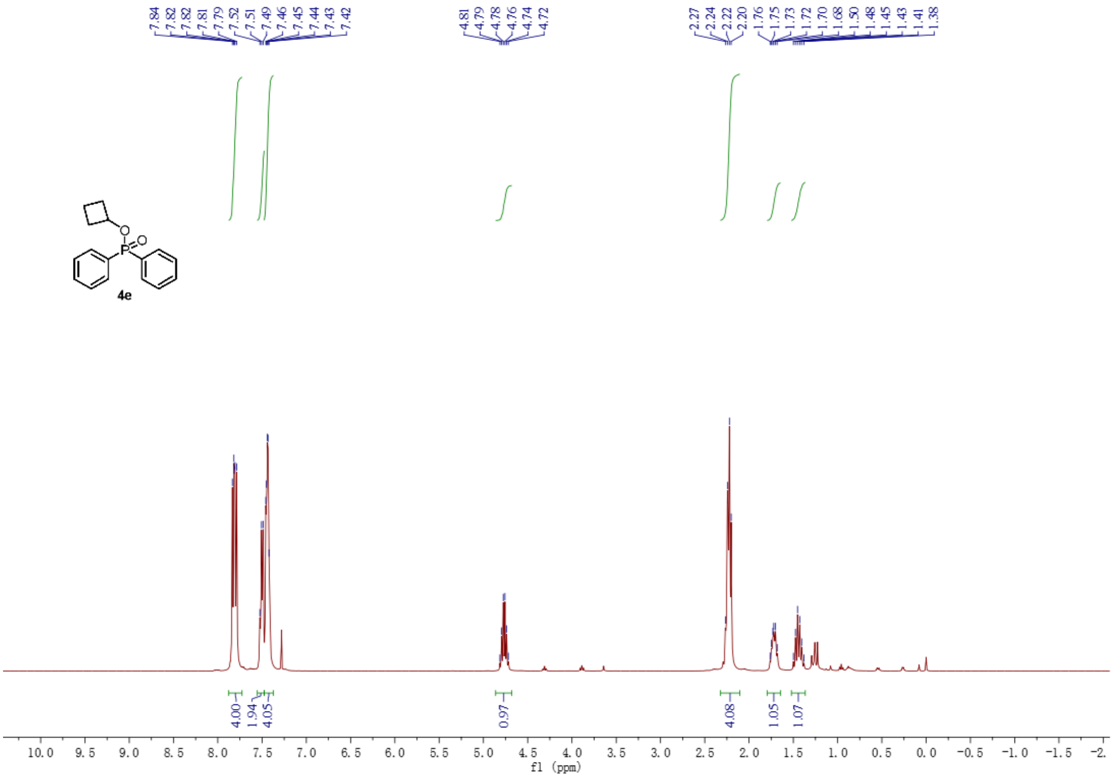

^{13}C NMR (101 MHz, $CDCl_3$) spectrum for 4e

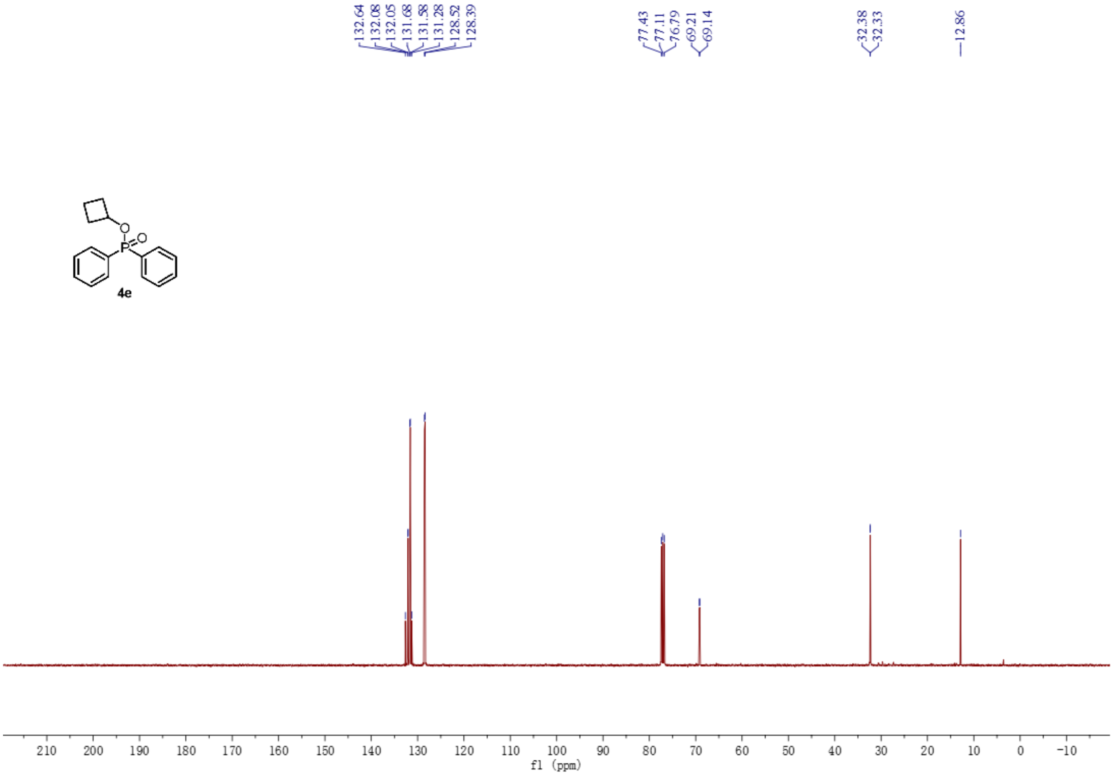

^{31}P NMR (121 MHz, CDCl$_3$) spectrum for 4e

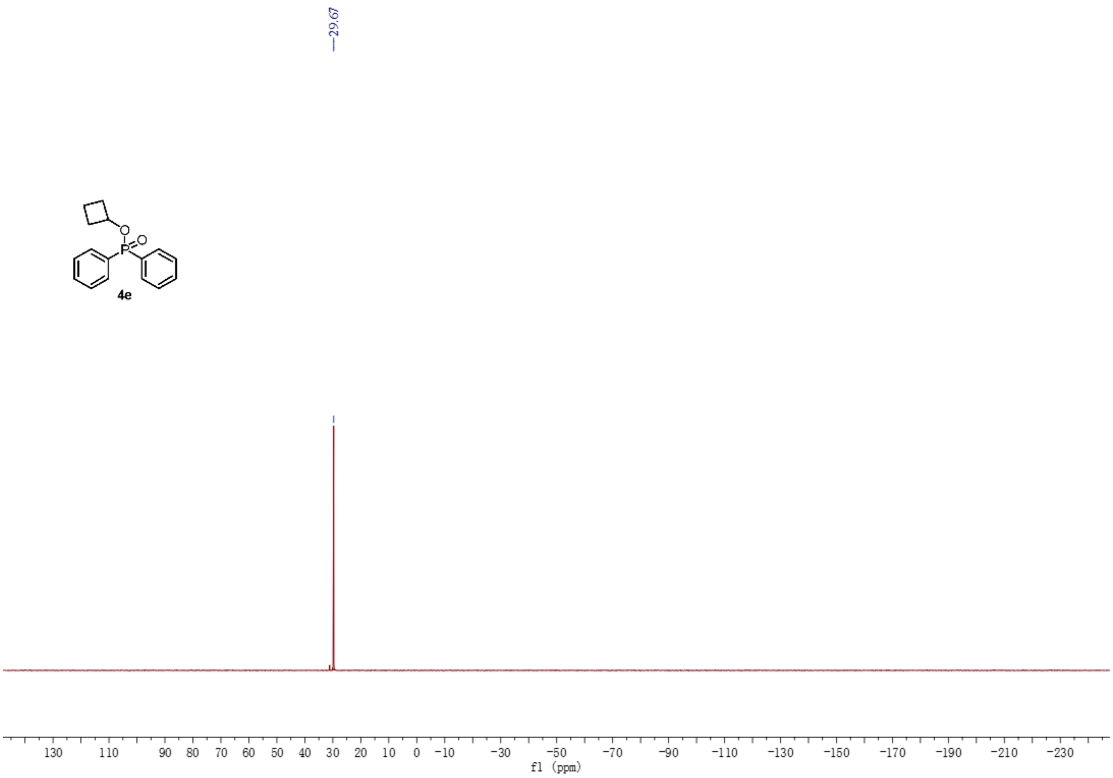

^{1}H NMR (400 MHz, CDCl$_3$) spectrum for 4f

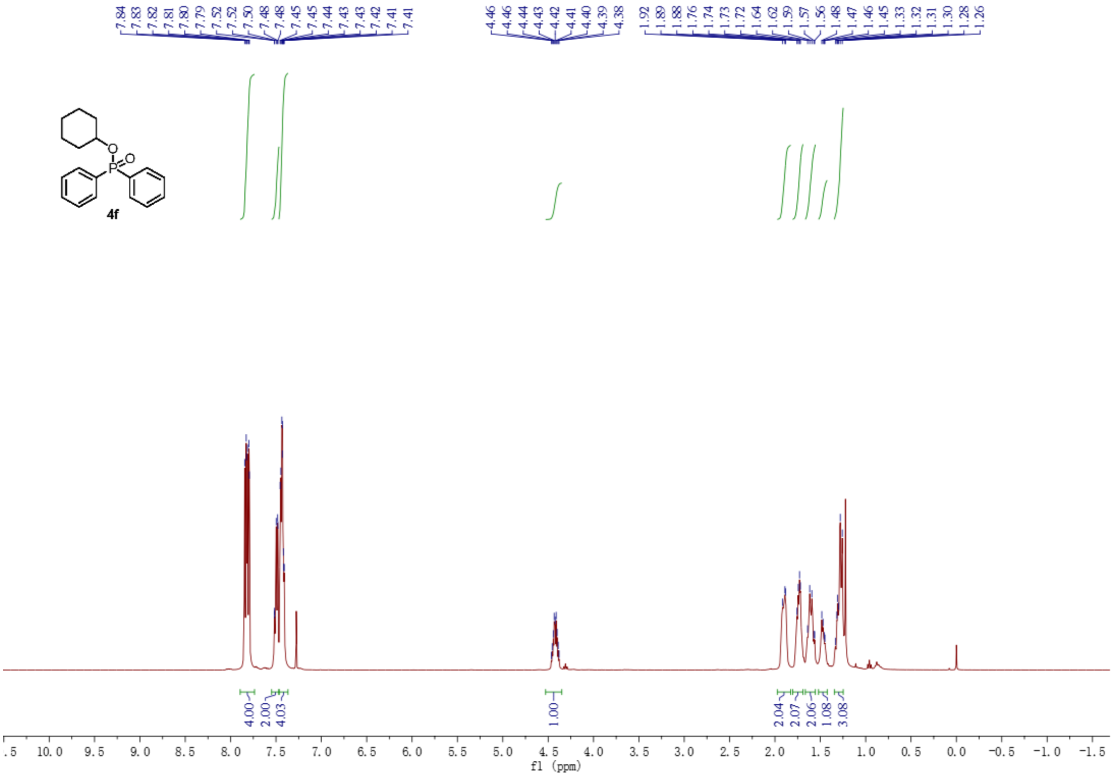

^{13}C NMR (101 MHz, $CDCl_3$) spectrum for 4f

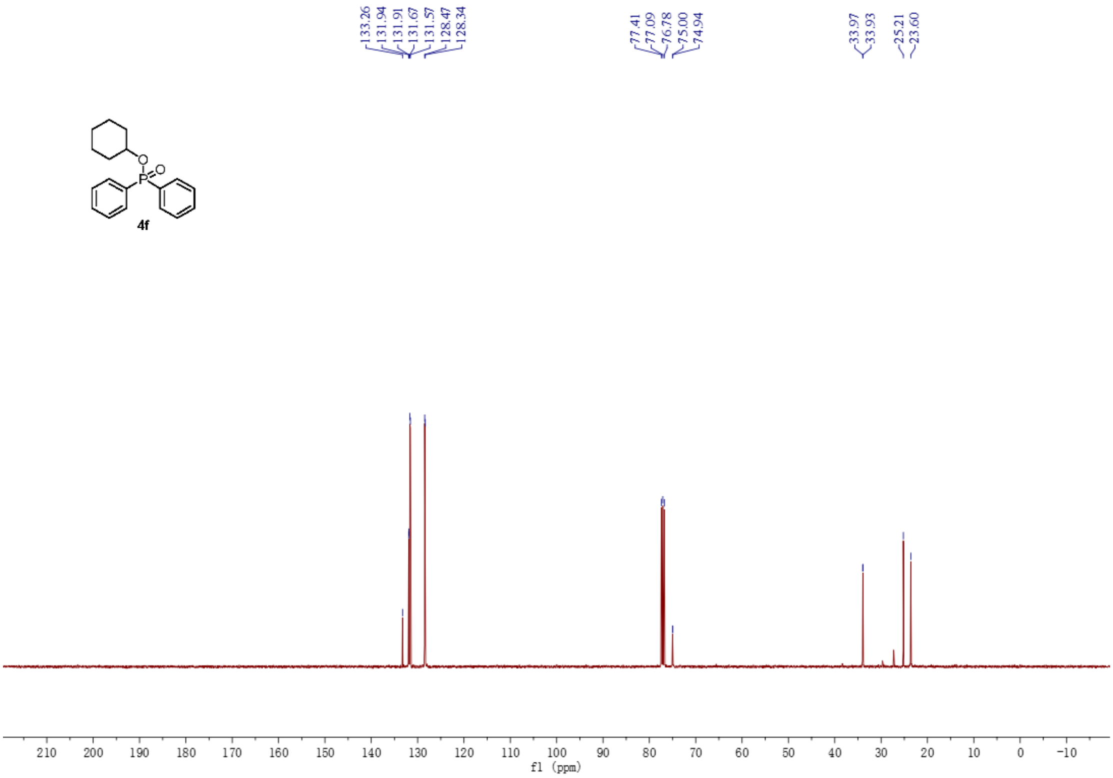

^{31}P NMR (121 MHz, $CDCl_3$) spectrum for 4f

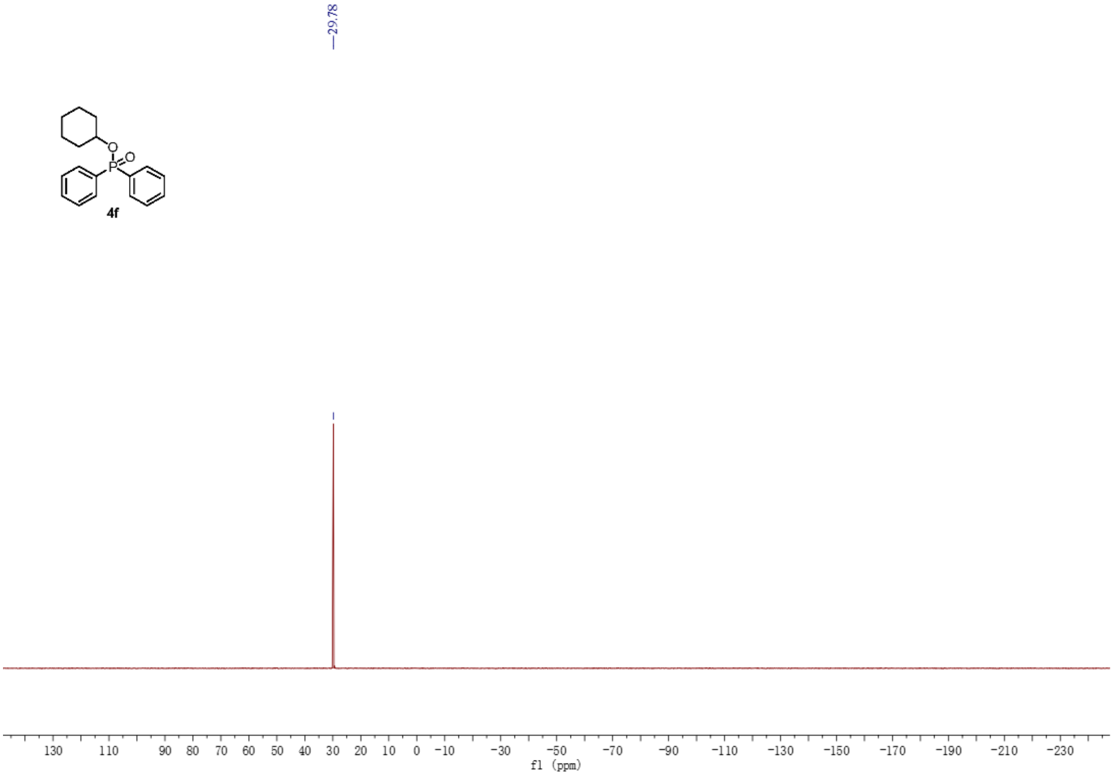

^{1}H NMR (400 MHz, $CDCl_3$) spectrum for 4g

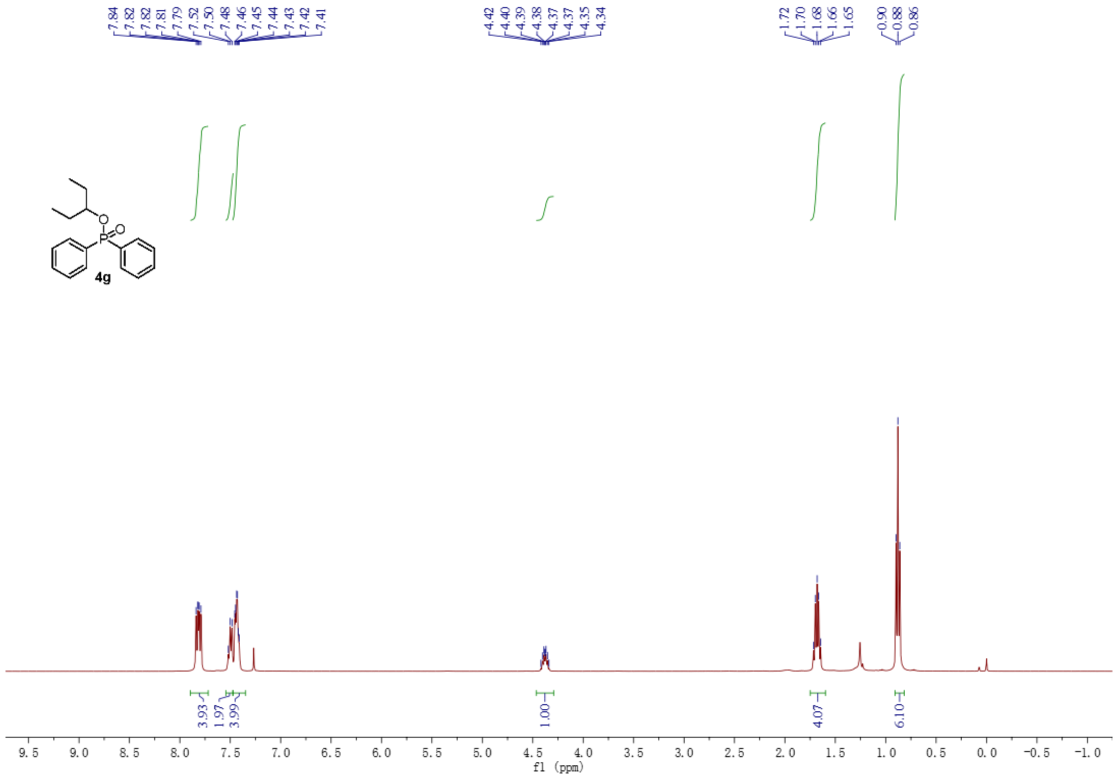

^{13}C NMR (101 MHz, $CDCl_3$) spectrum for 4g

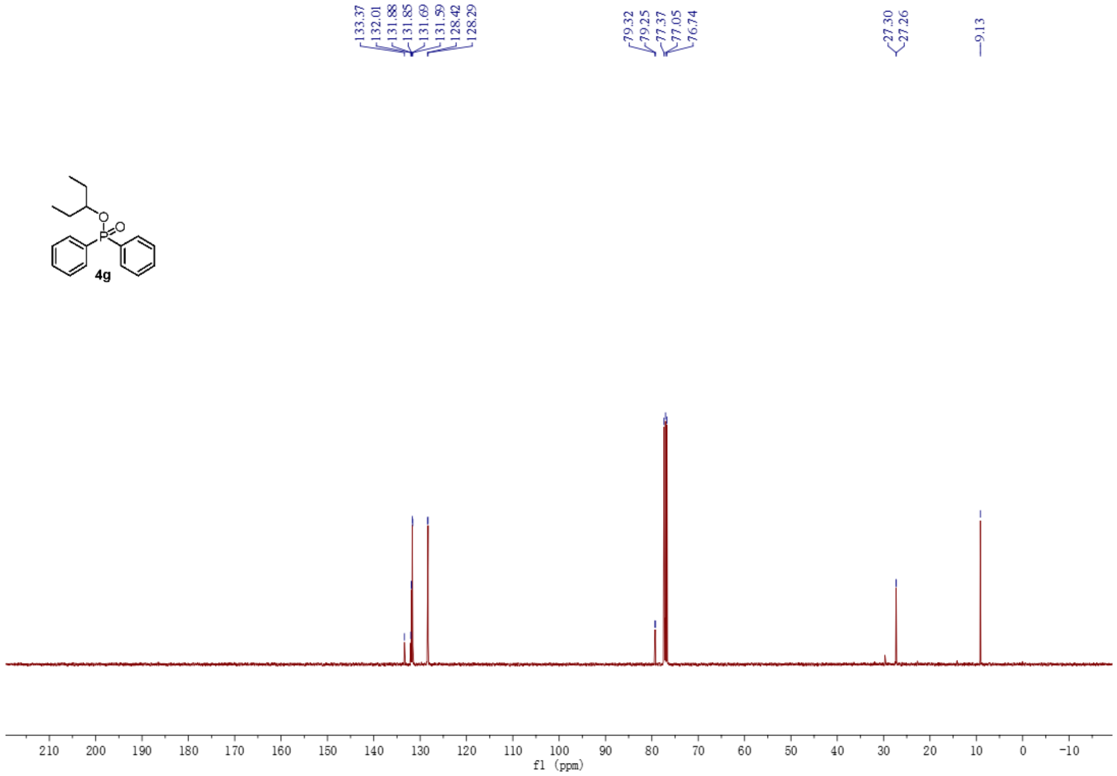

^{31}P NMR (121 MHz, $CDCl_3$) spectrum for 4g

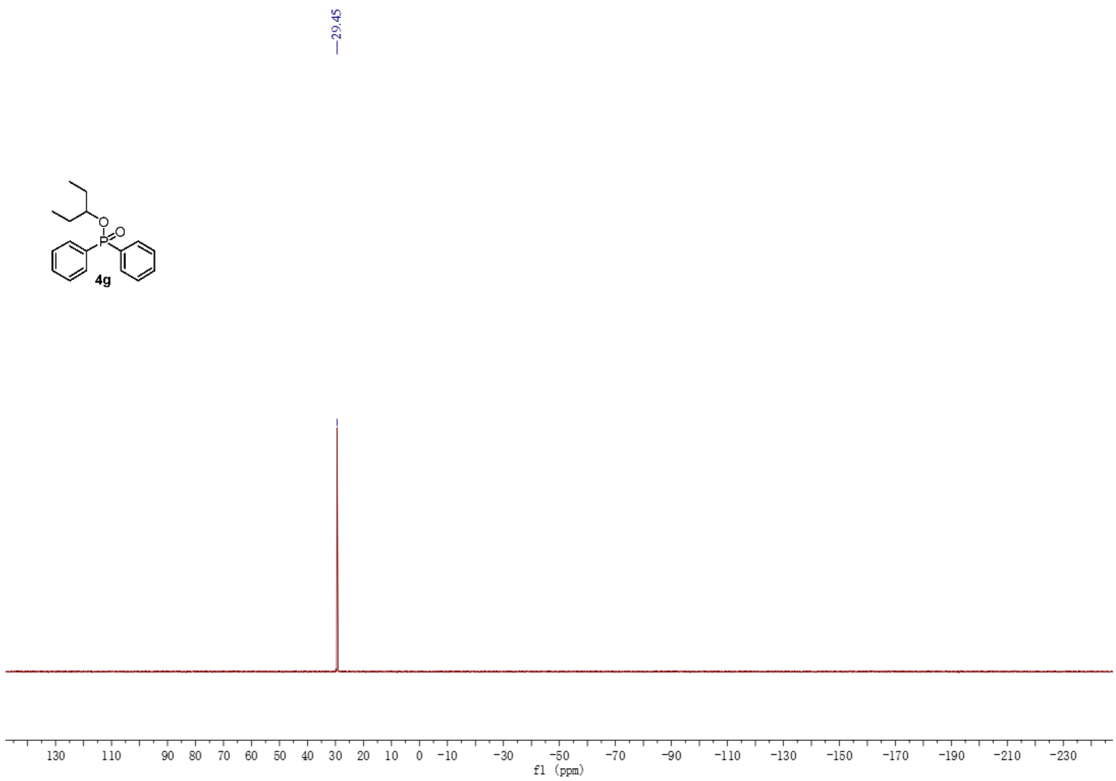

^{1}H NMR (400 MHz, $CDCl_3$) spectrum for 4i

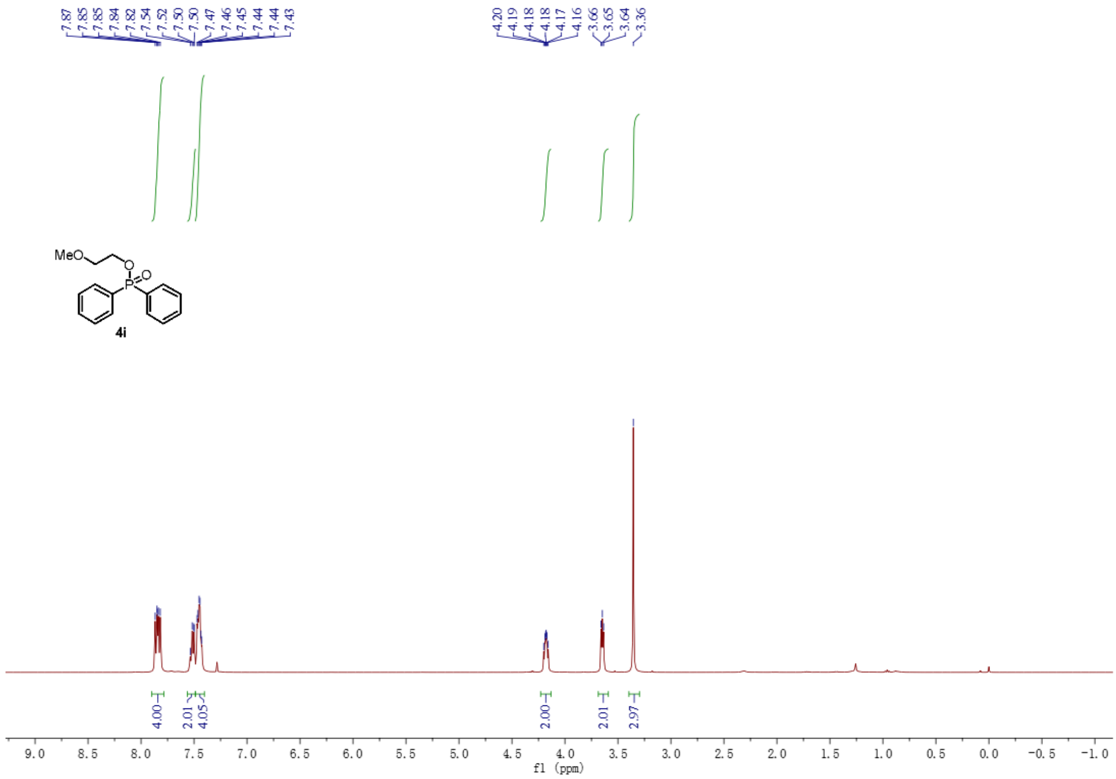

^{13}C NMR (101 MHz, CDCl$_3$) spectrum for 4i

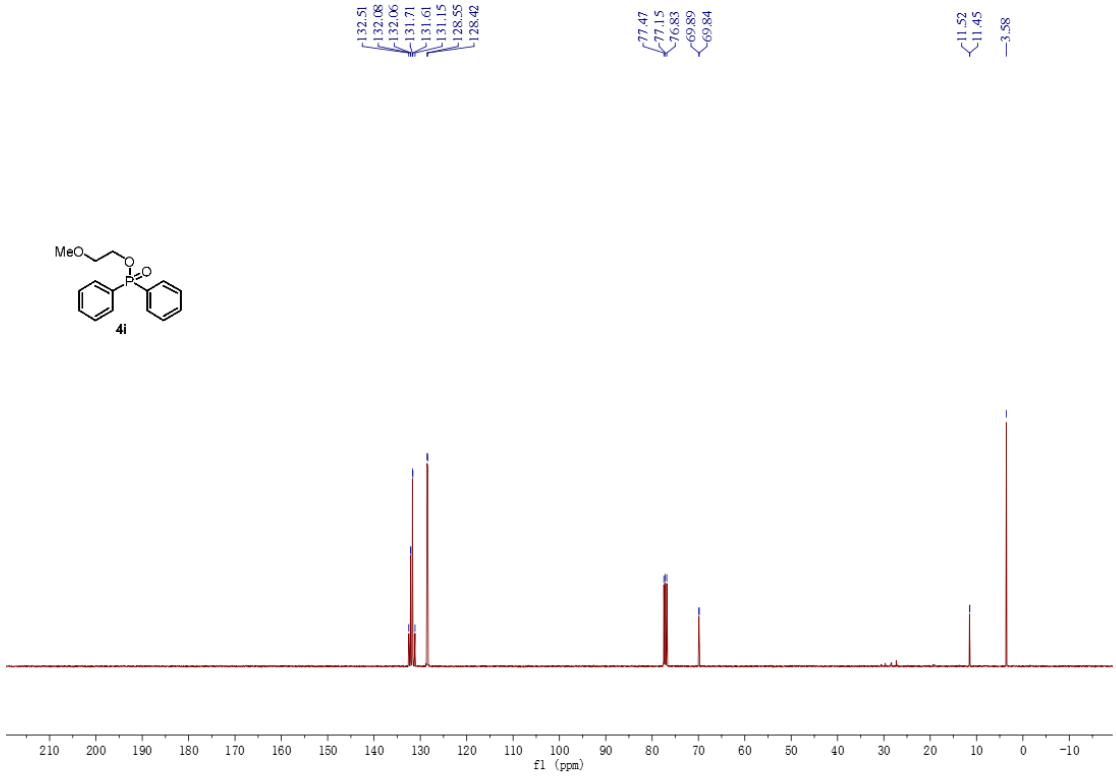

^{31}P NMR (121 MHz, CDCl$_3$) spectrum for 4i

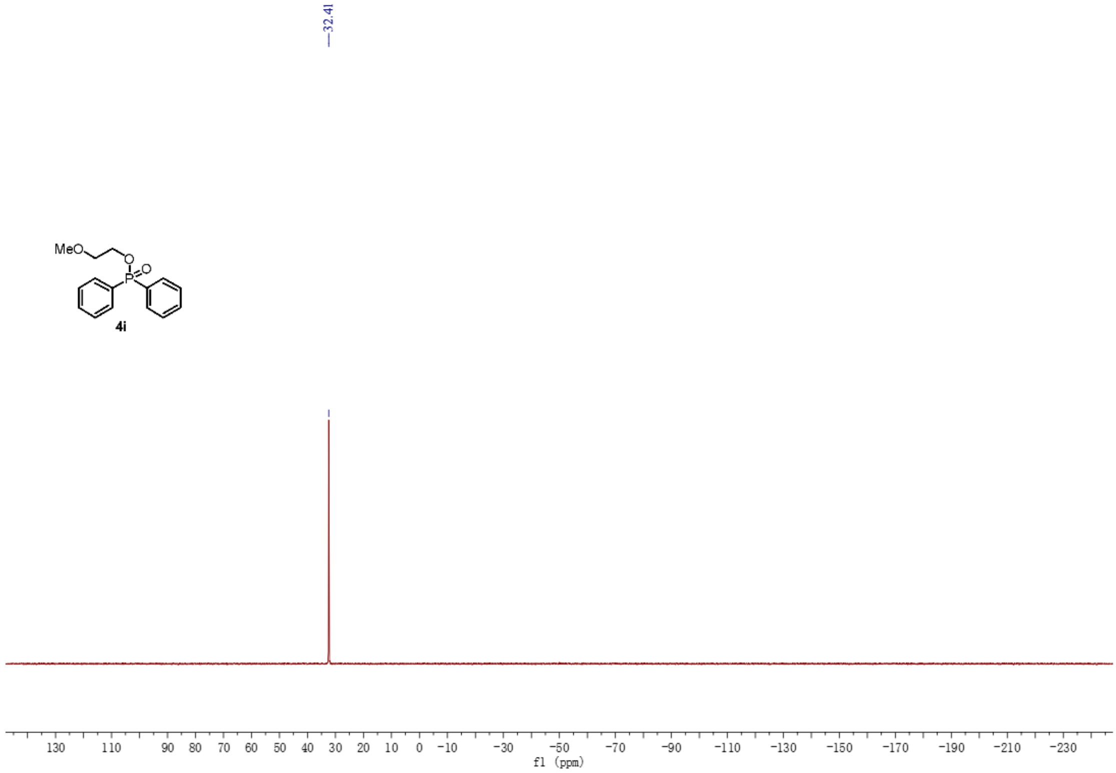

^{1}H NMR (400 MHz, CDCl3) spectrum for 4j

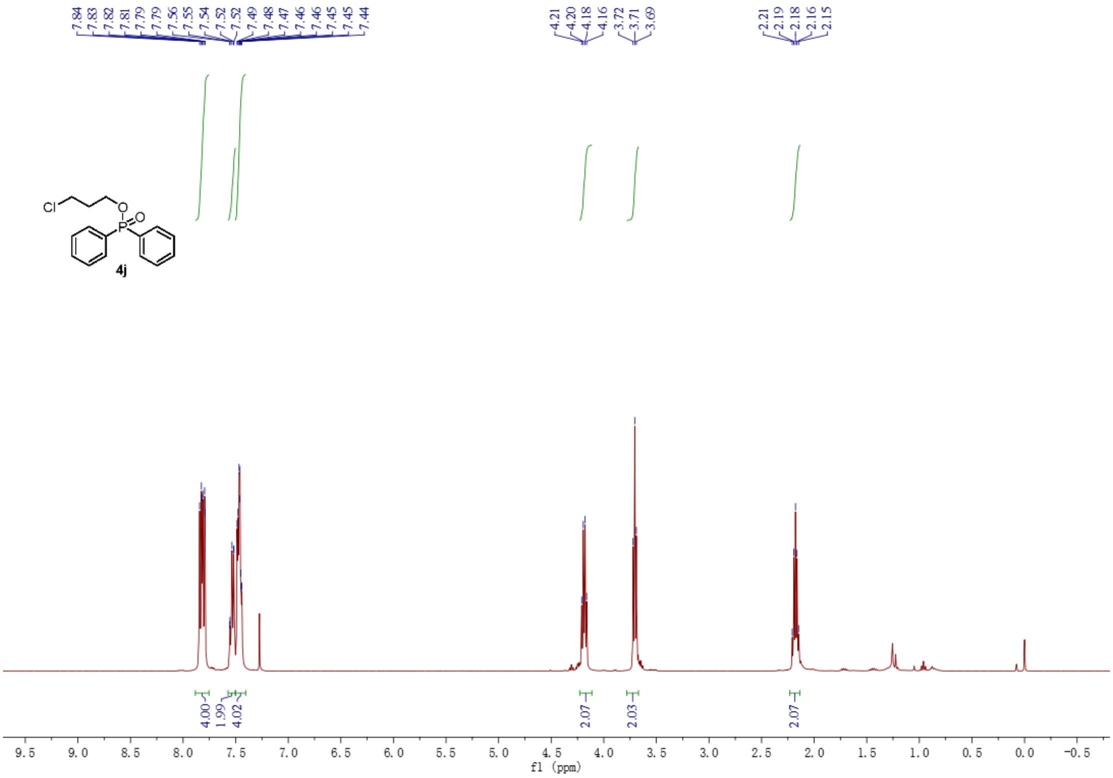

^{13}C NMR (101 MHz, CDCl3) spectrum for 4j

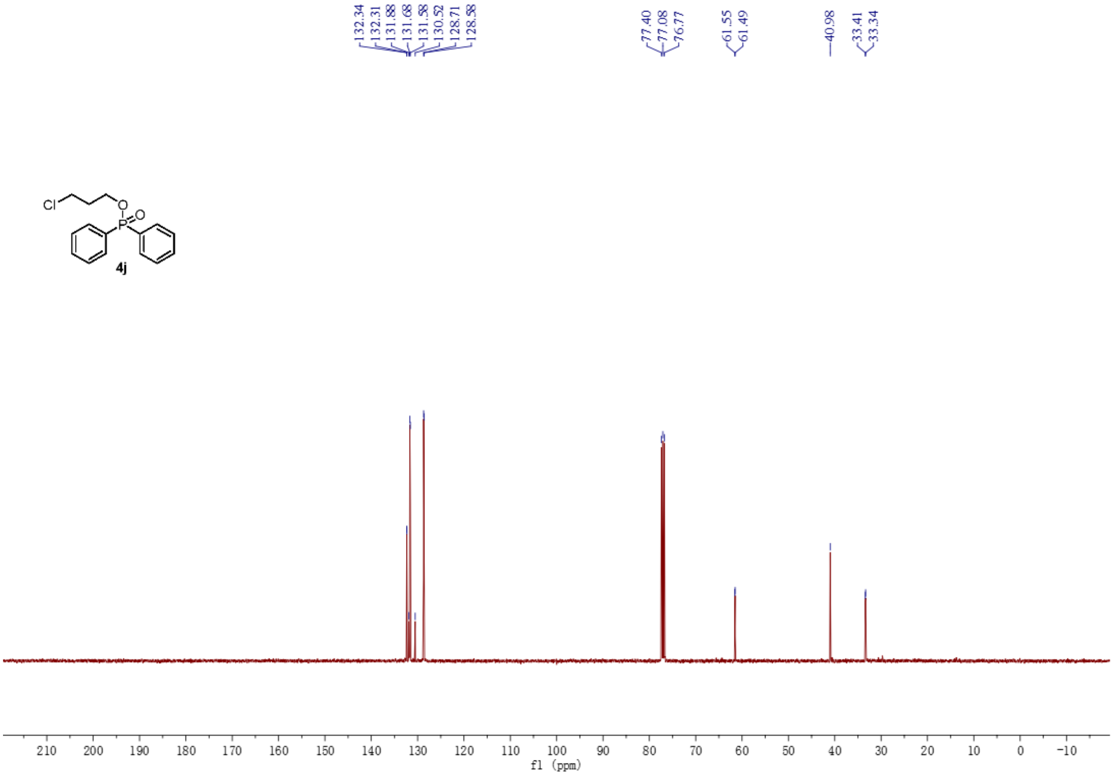

^{31}P NMR (121 MHz, $CDCl_3$) spectrum for 4j

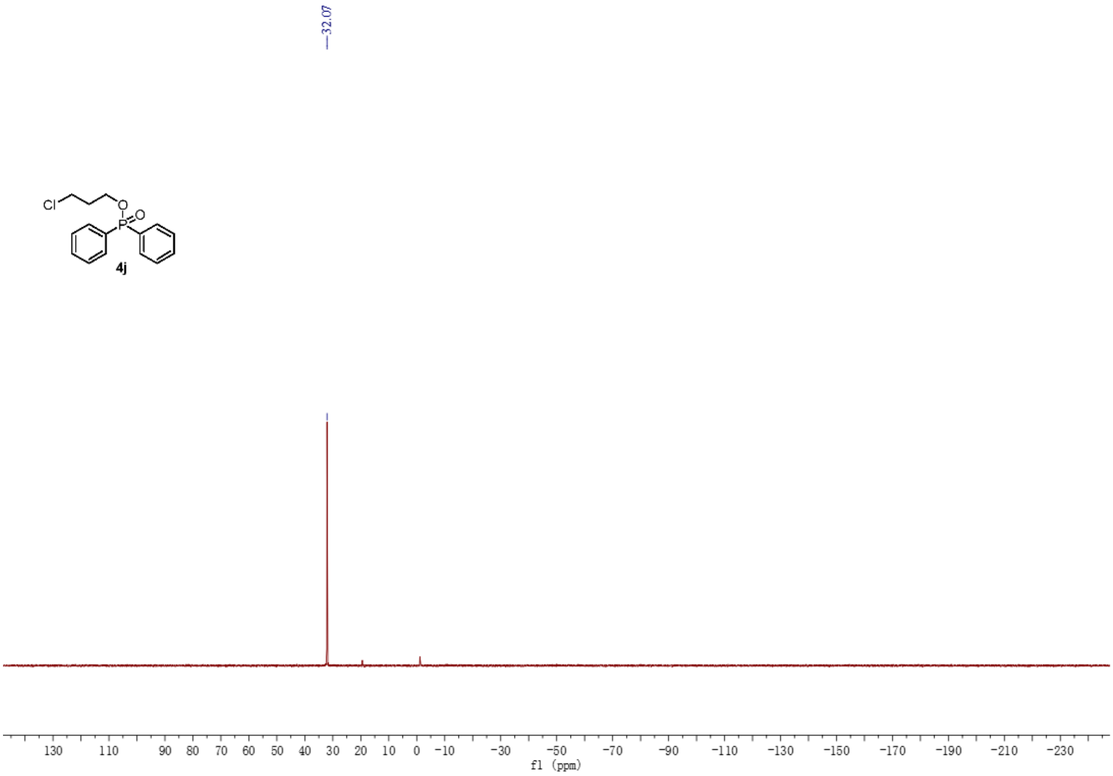

^{1}H NMR (400 MHz, $CDCl_3$) spectrum for 4k

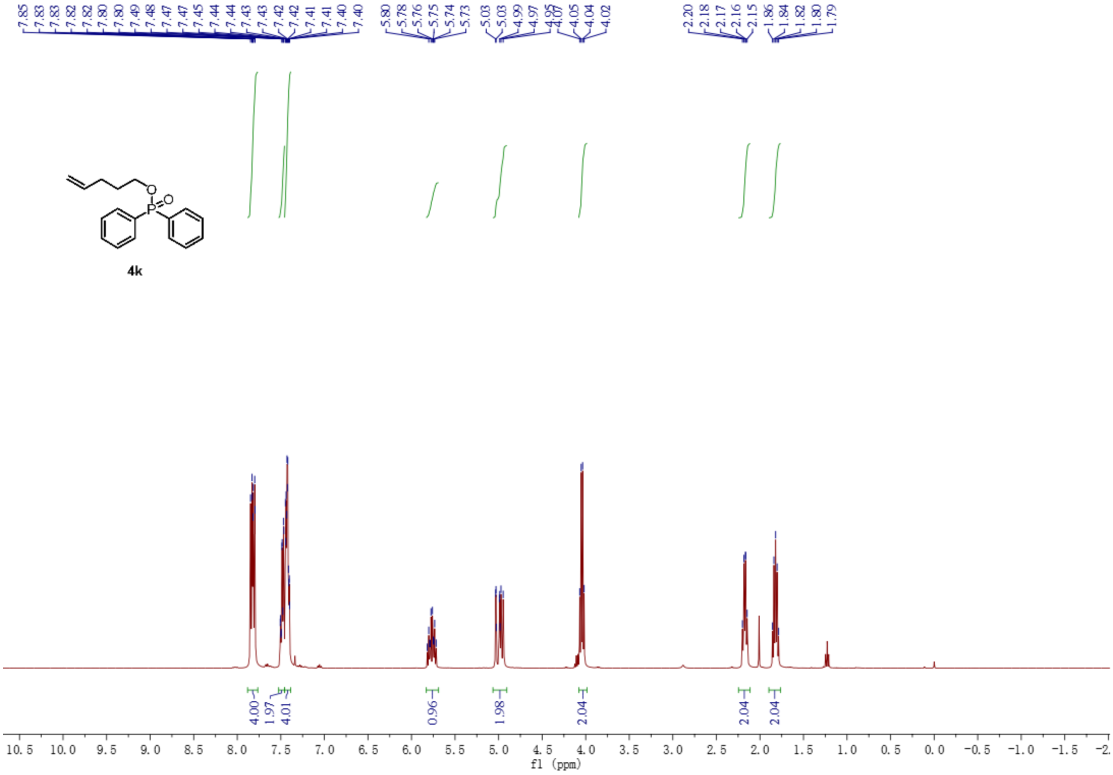

^{13}C NMR (101 MHz, CDCl$_3$) spectrum for 4k

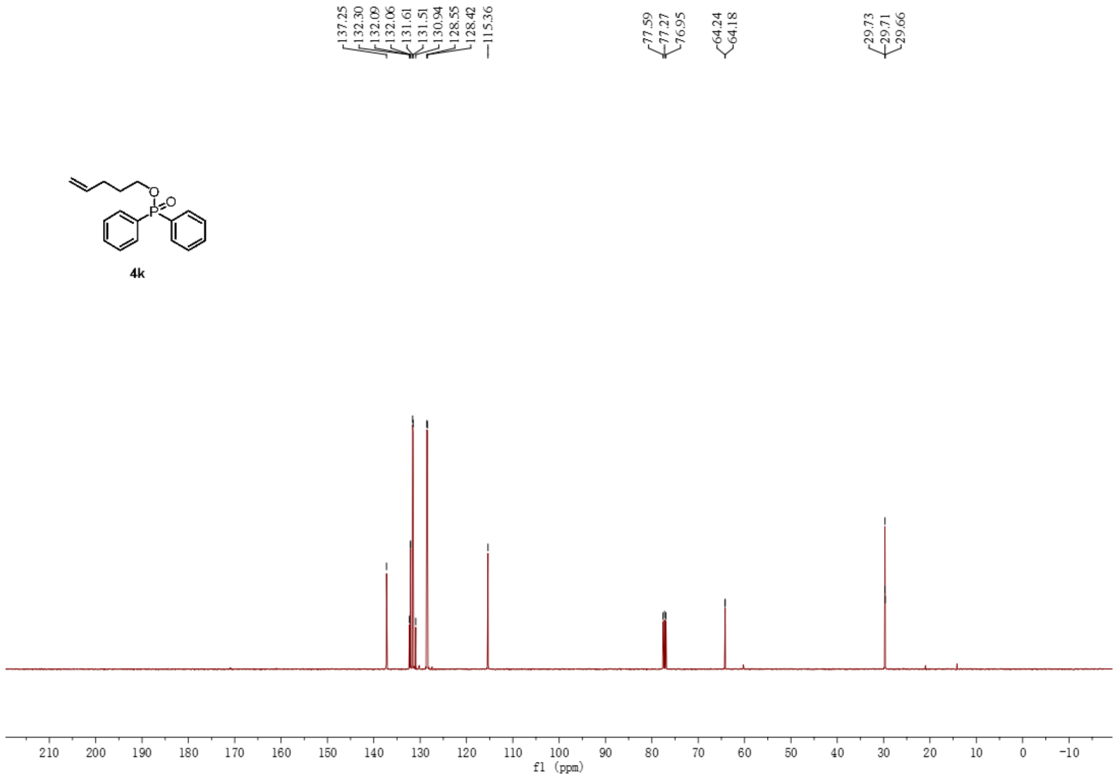

^{31}P NMR (121 MHz, CDCl$_3$) spectrum for 4k

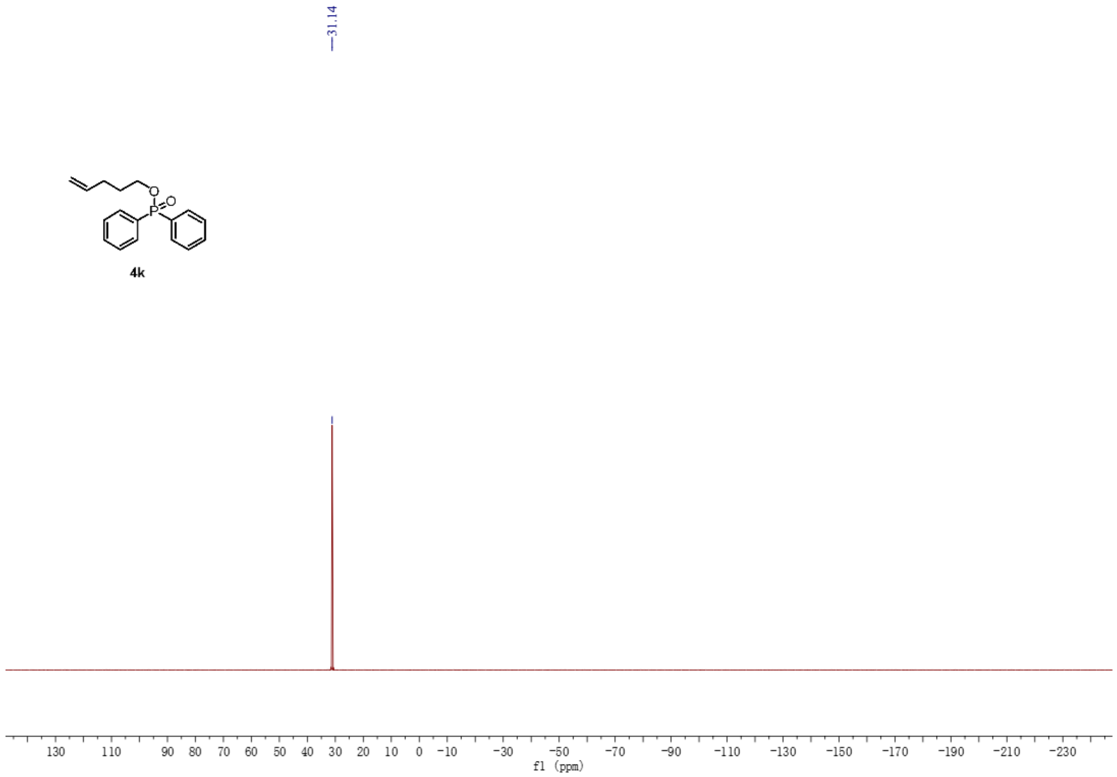

^{1}H NMR (400 MHz, $CDCl_3$) spectrum for 4l

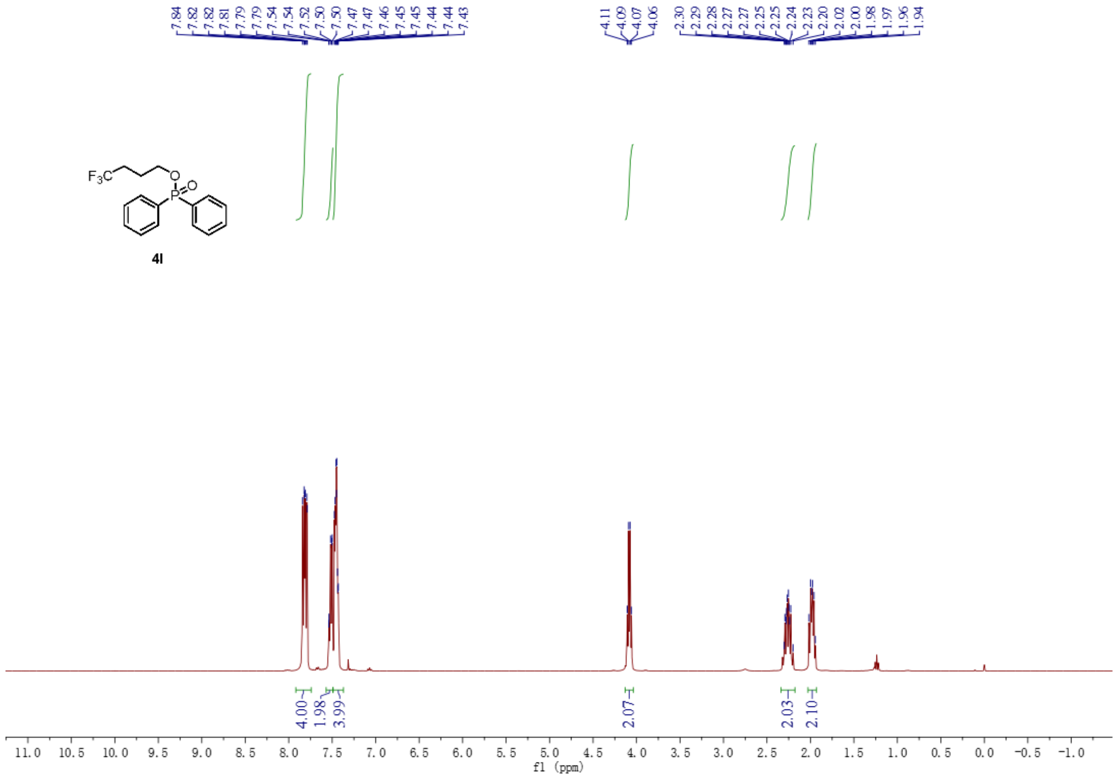

^{13}C NMR (101 MHz, $CDCl_3$) spectrum for 4l

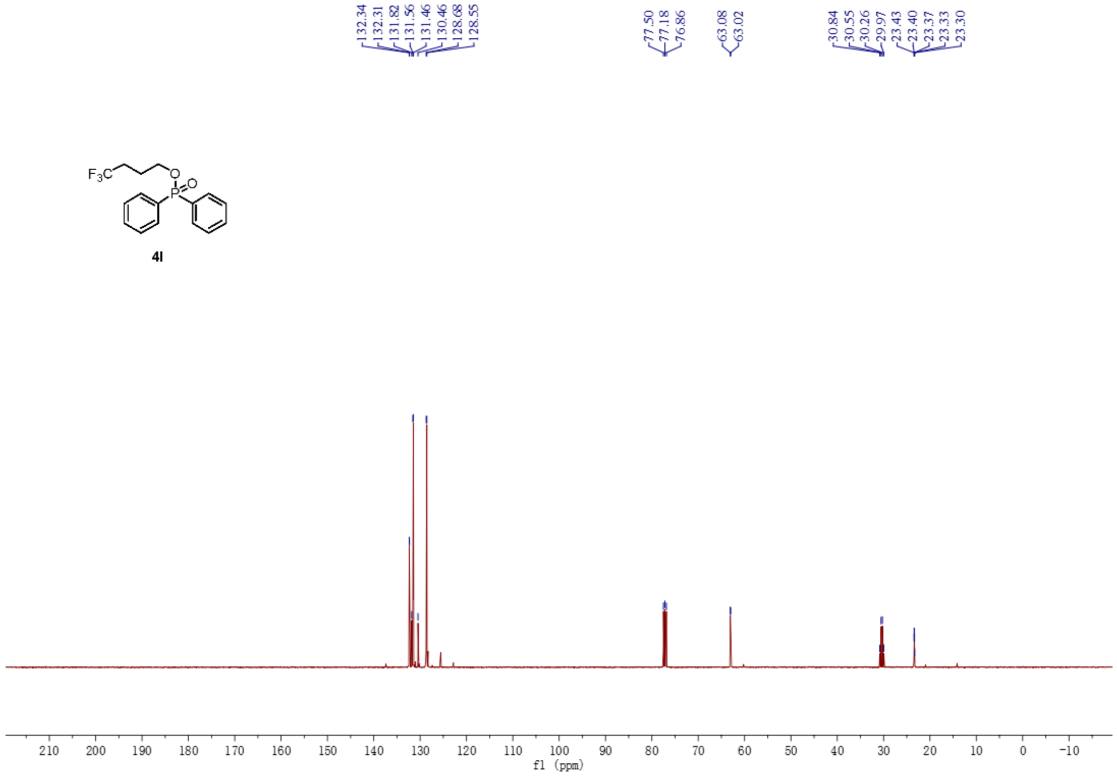

^{31}P NMR (121 MHz, $CDCl_3$) spectrum for 4l

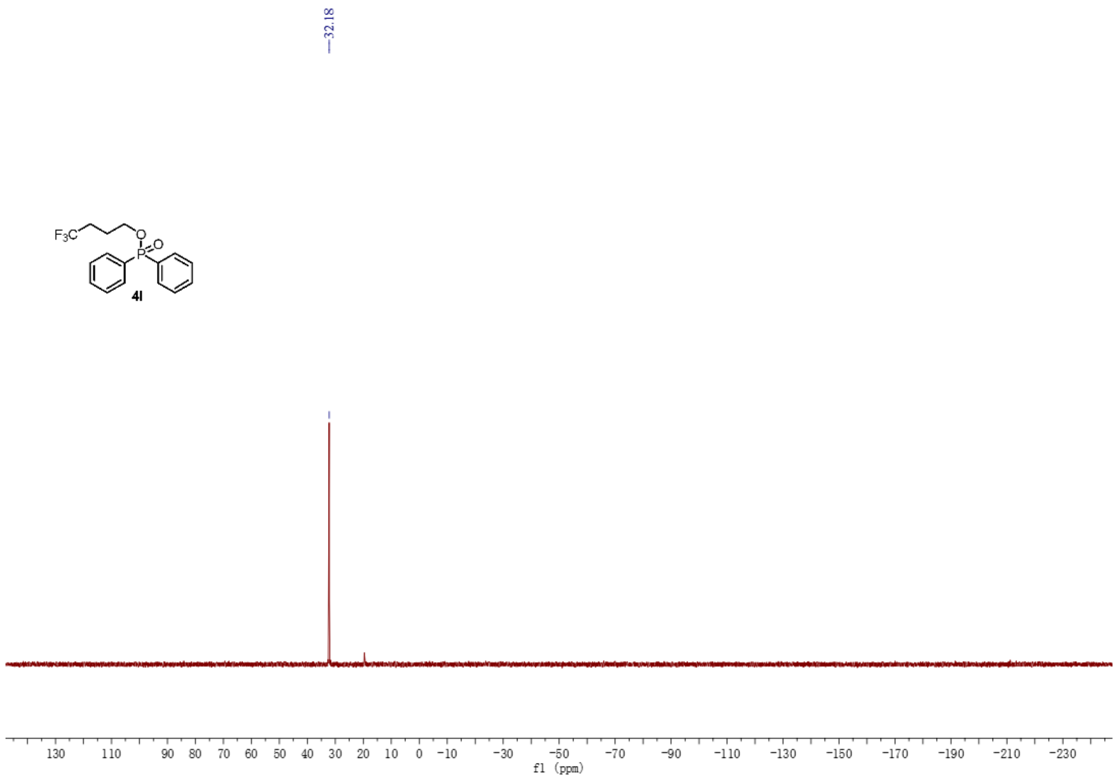

^{19}F NMR (282 MHz, $CDCl_3$) spectrum for 4l

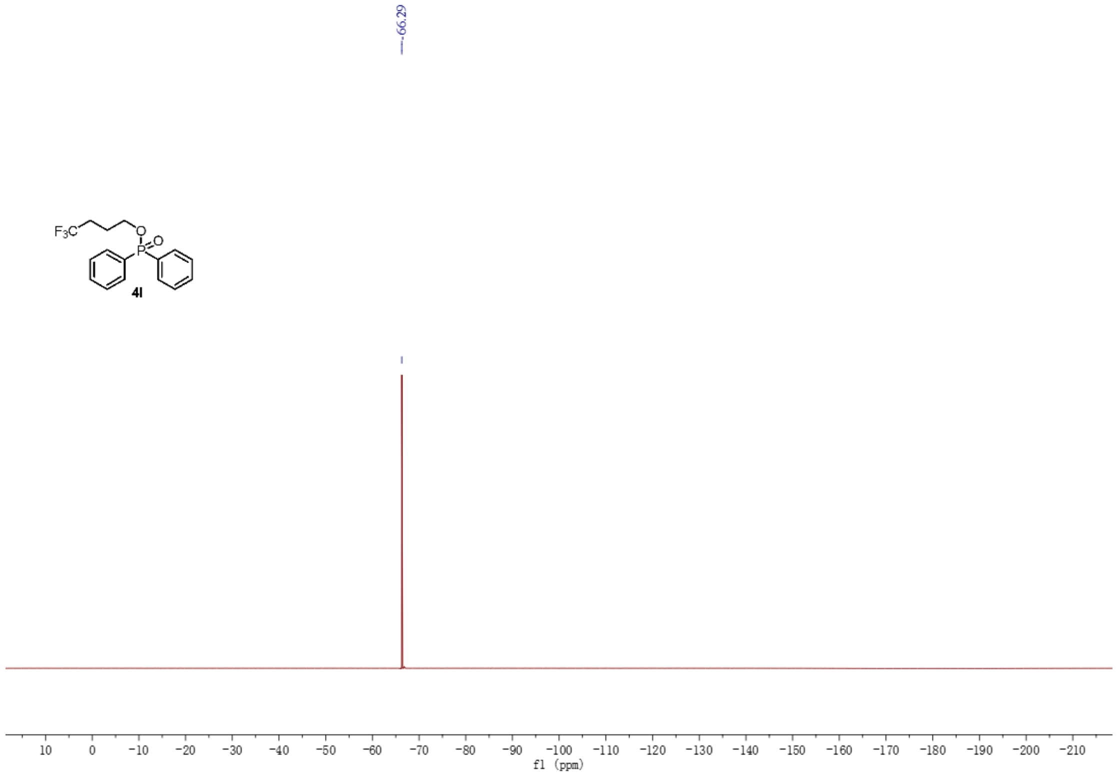

^{1}H NMR (400 MHz, $CDCl_3$) spectrum for 4m

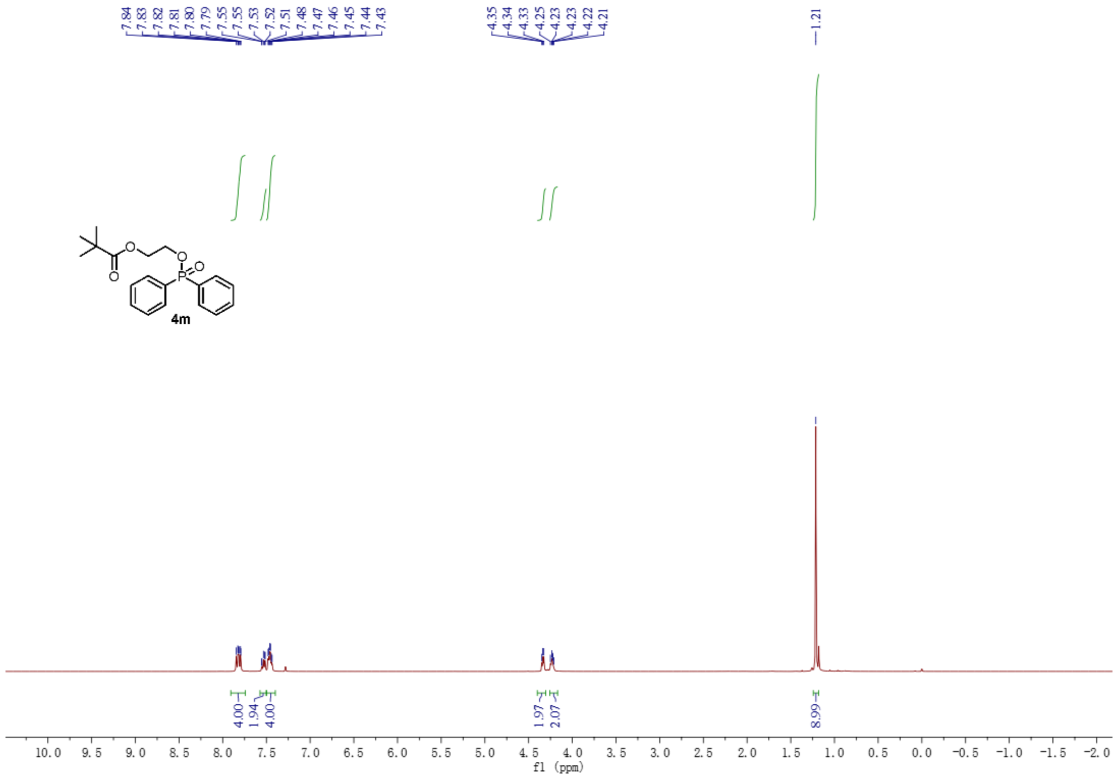

^{13}C NMR (101 MHz, $CDCl_3$) spectrum for 4m

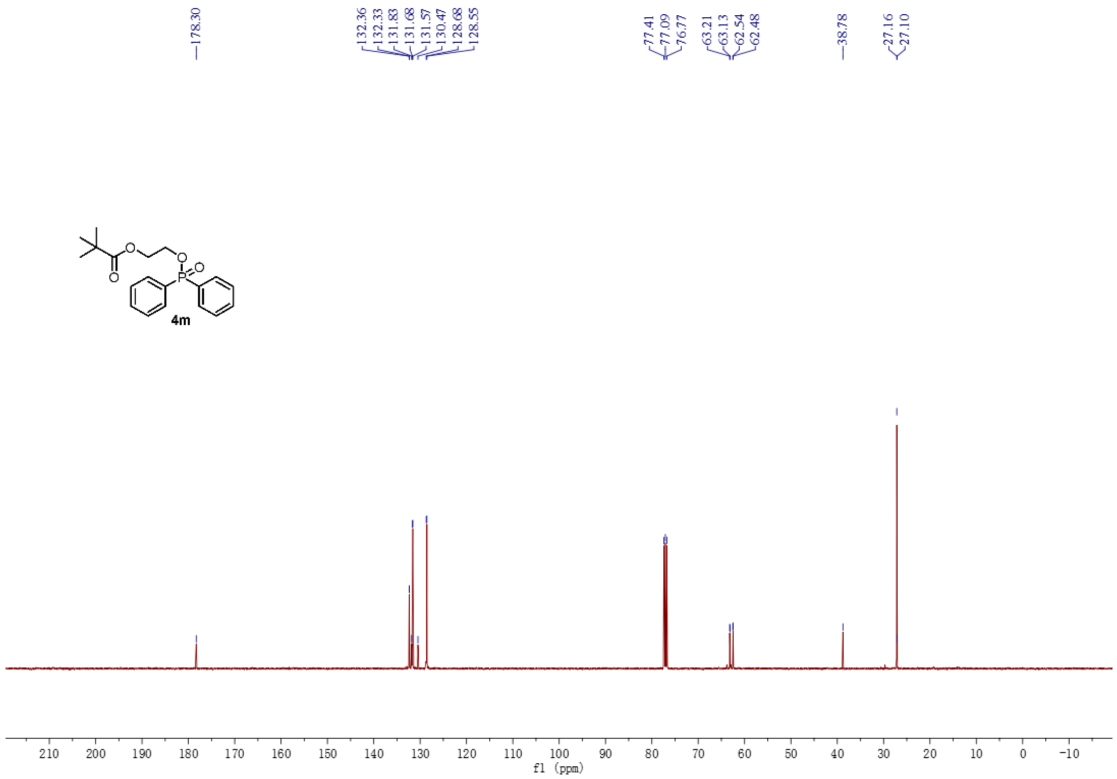

^{31}P NMR (121 MHz, $CDCl_3$) spectrum for 4m

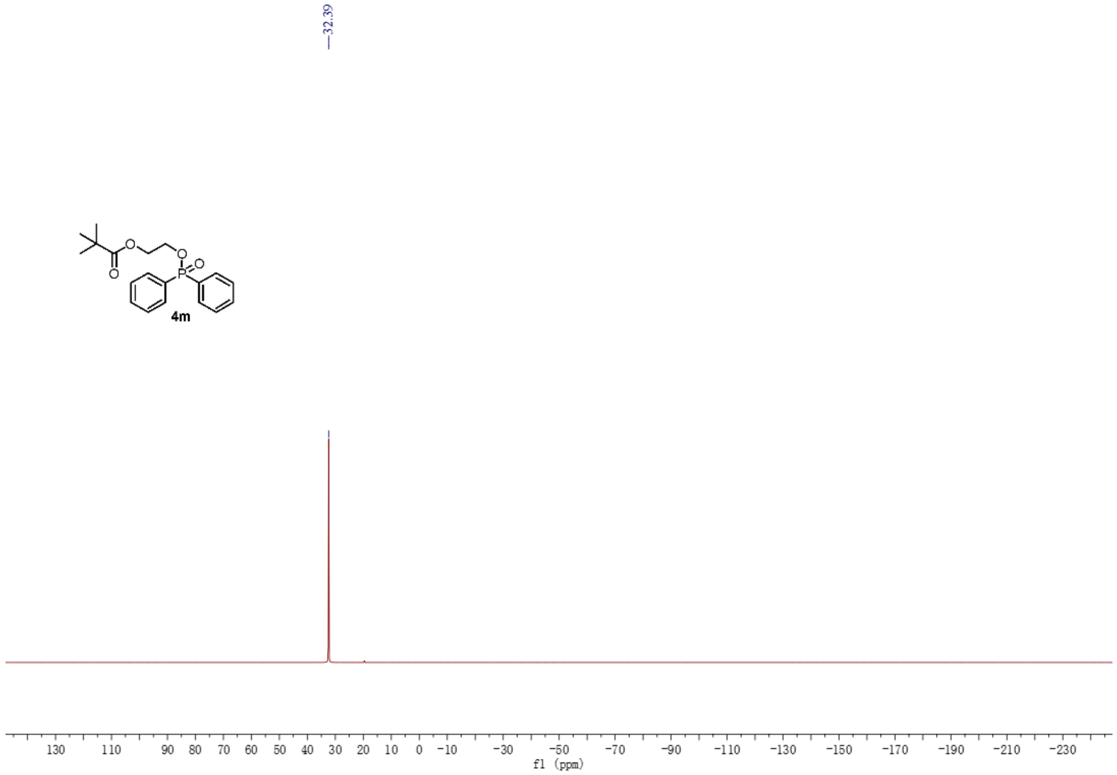

^{1}H NMR (400 MHz, $CDCl_3$) spectrum for 4n

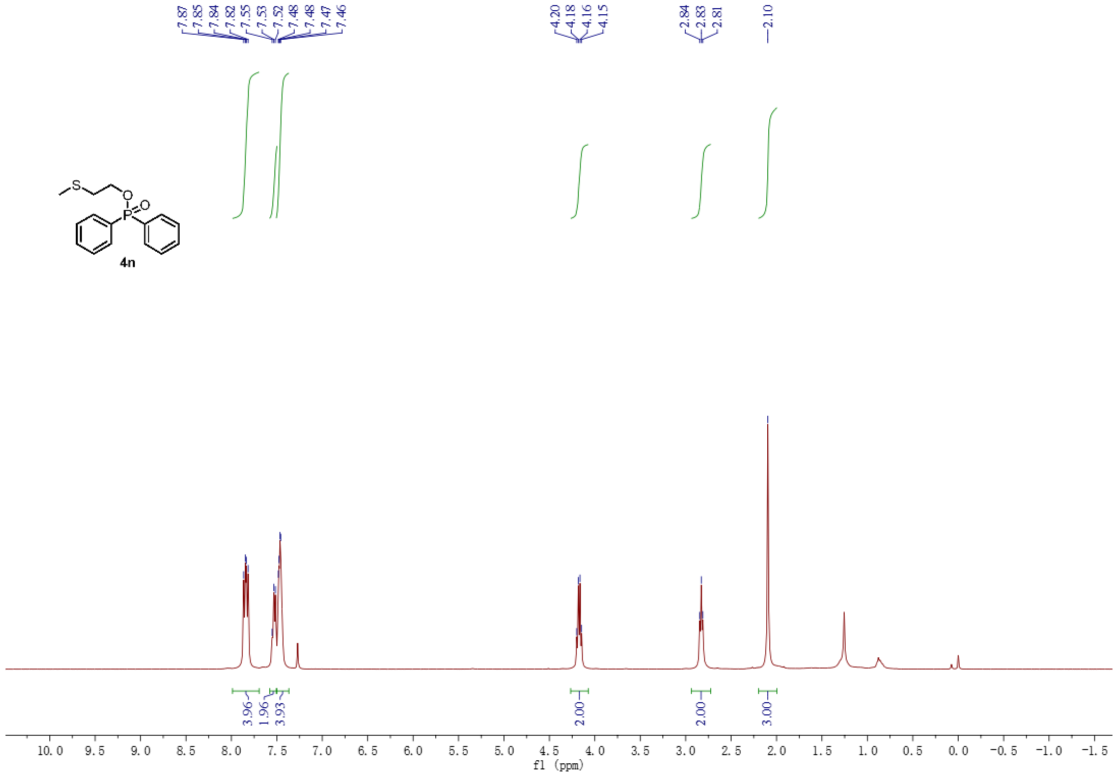

^{13}C NMR (101 MHz, $CDCl_3$) spectrum for 4n

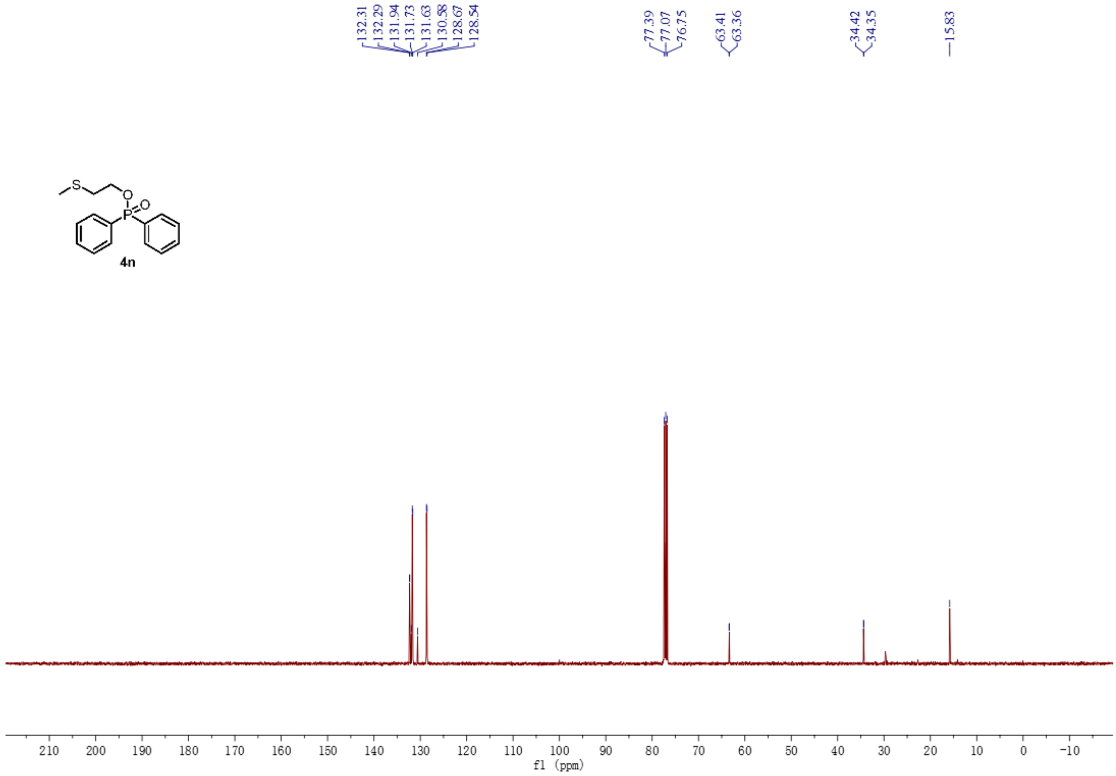

^{31}P NMR (121 MHz, $CDCl_3$) spectrum for 4n

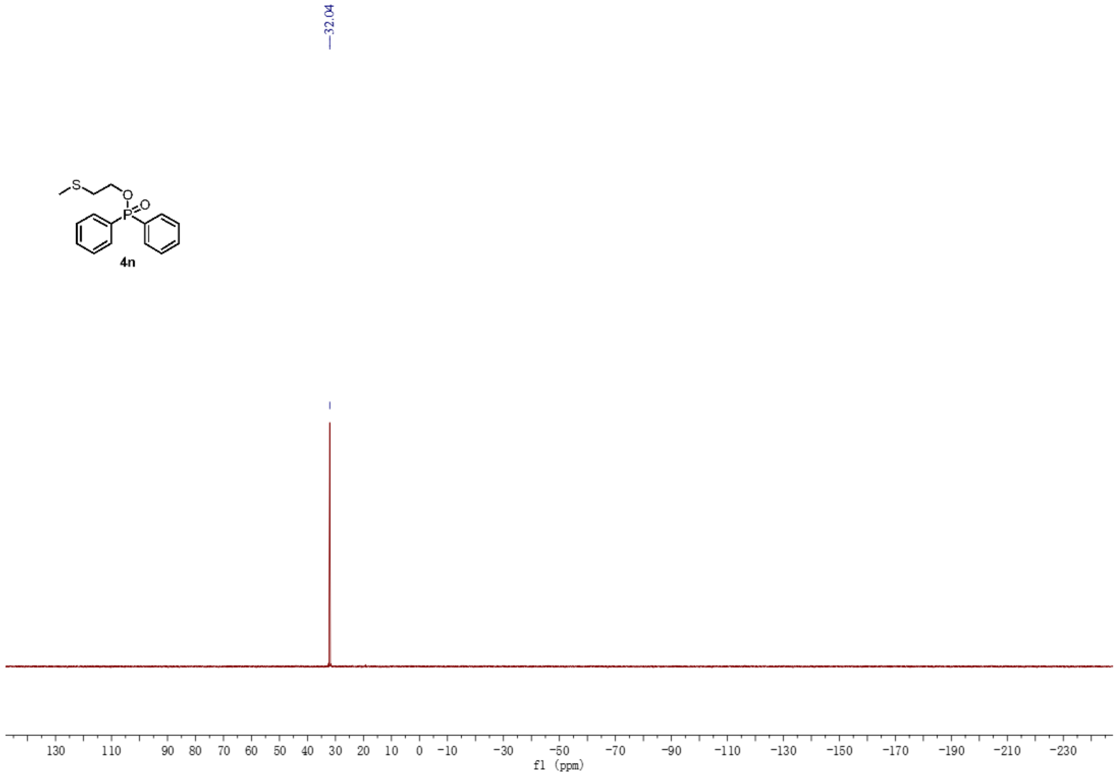

^{1}H NMR (400 MHz, $CDCl_3$) spectrum for 4o

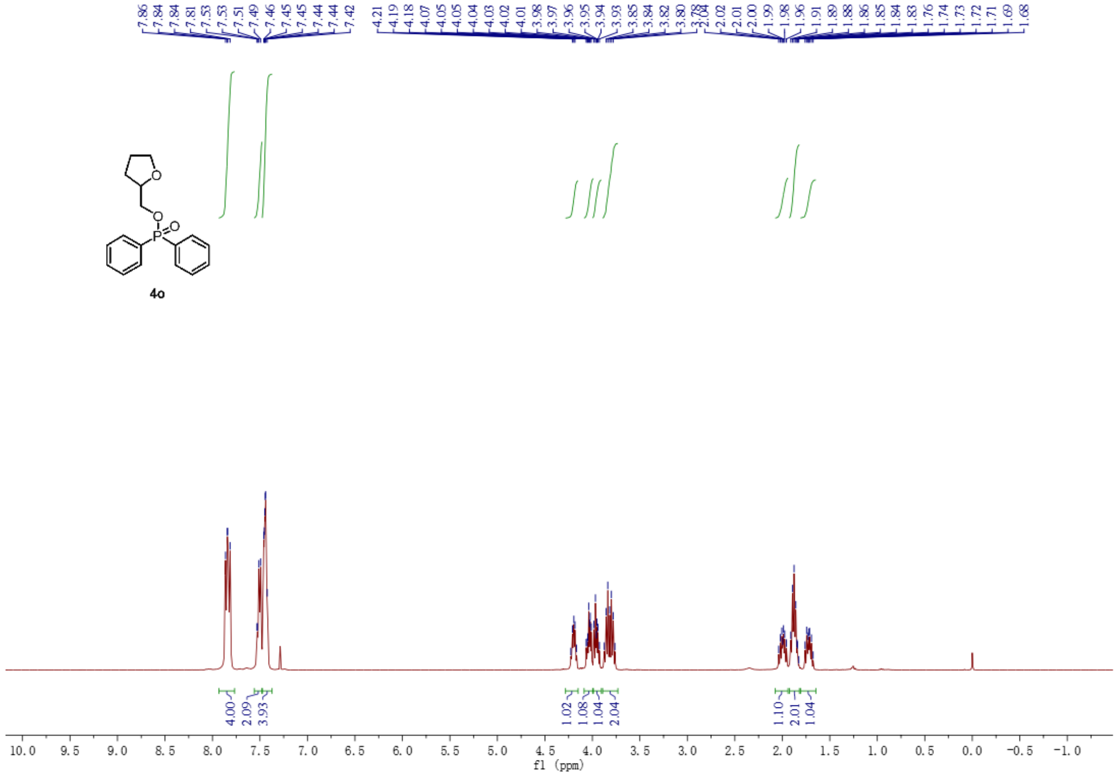

^{13}C NMR (101 MHz, $CDCl_3$) spectrum for 4o

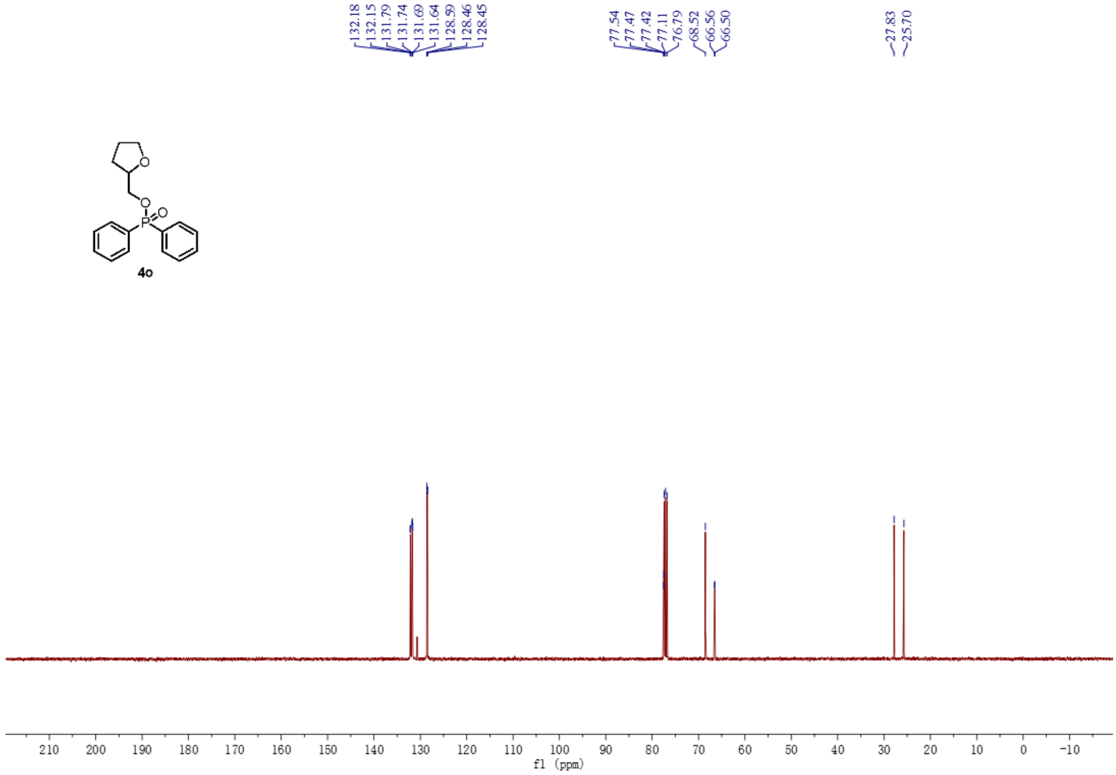

^{31}P NMR (121 MHz, $CDCl_3$) spectrum for 4o

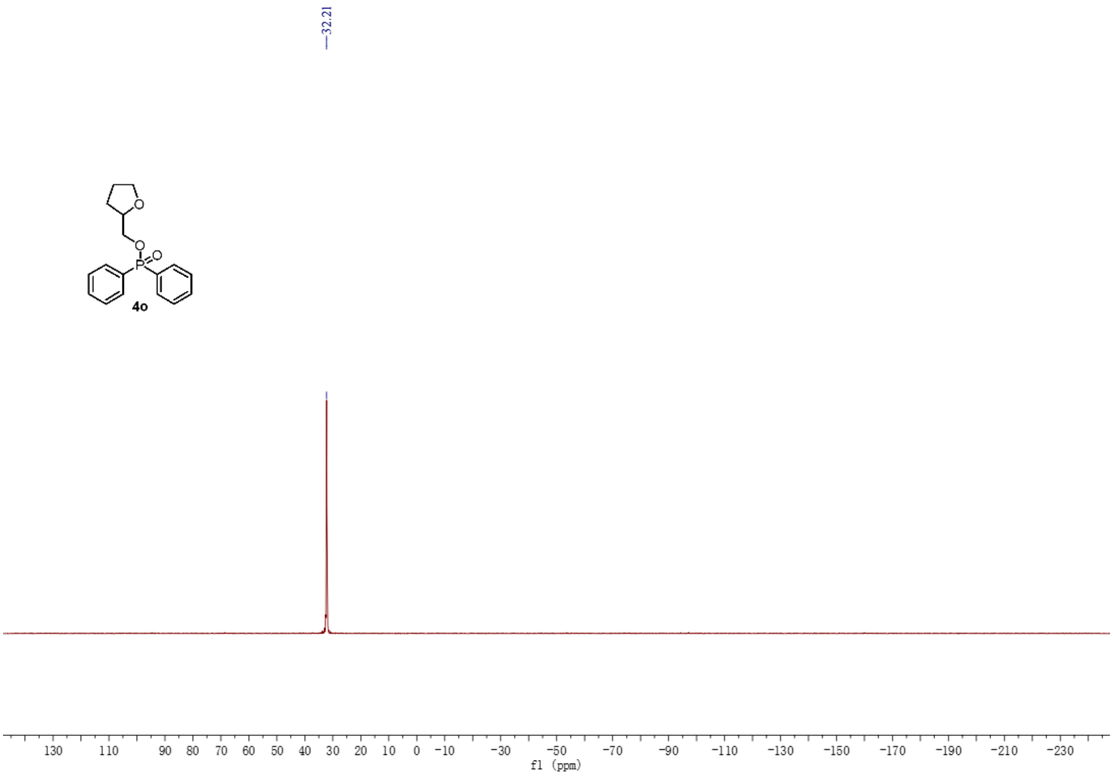

^{1}H NMR (400 MHz, $CDCl_3$) spectrum for 4p

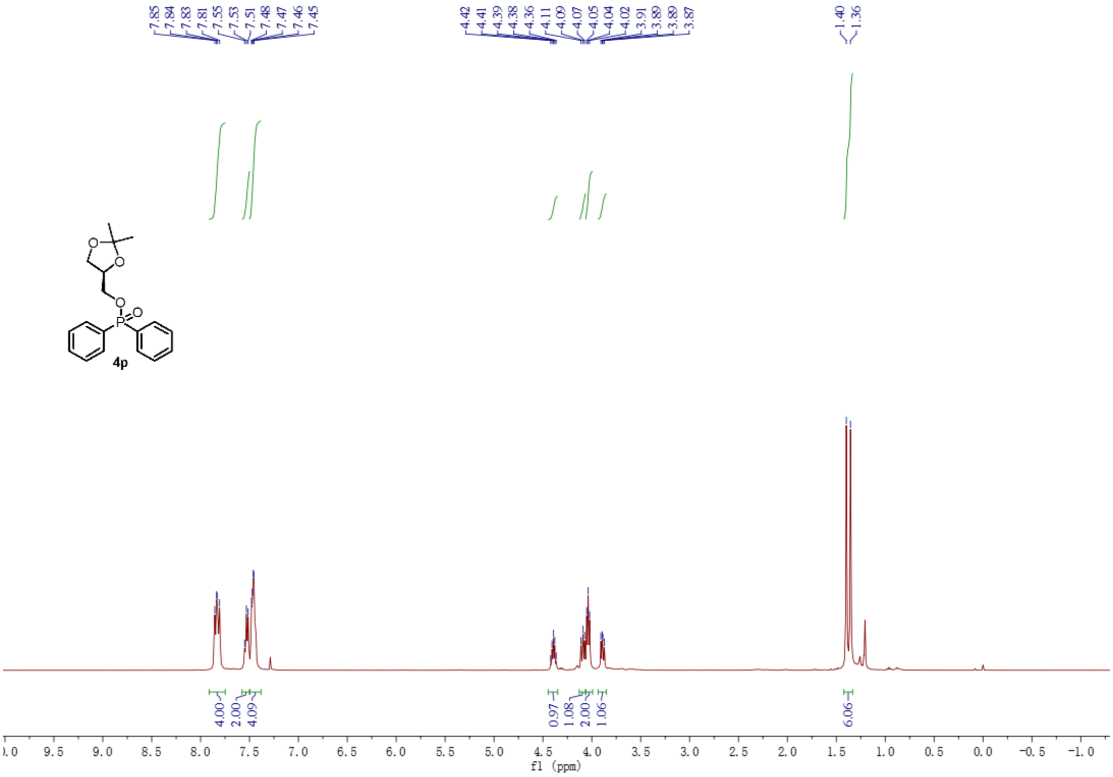

^{13}C NMR (101 MHz, $CDCl_3$) spectrum for 4p

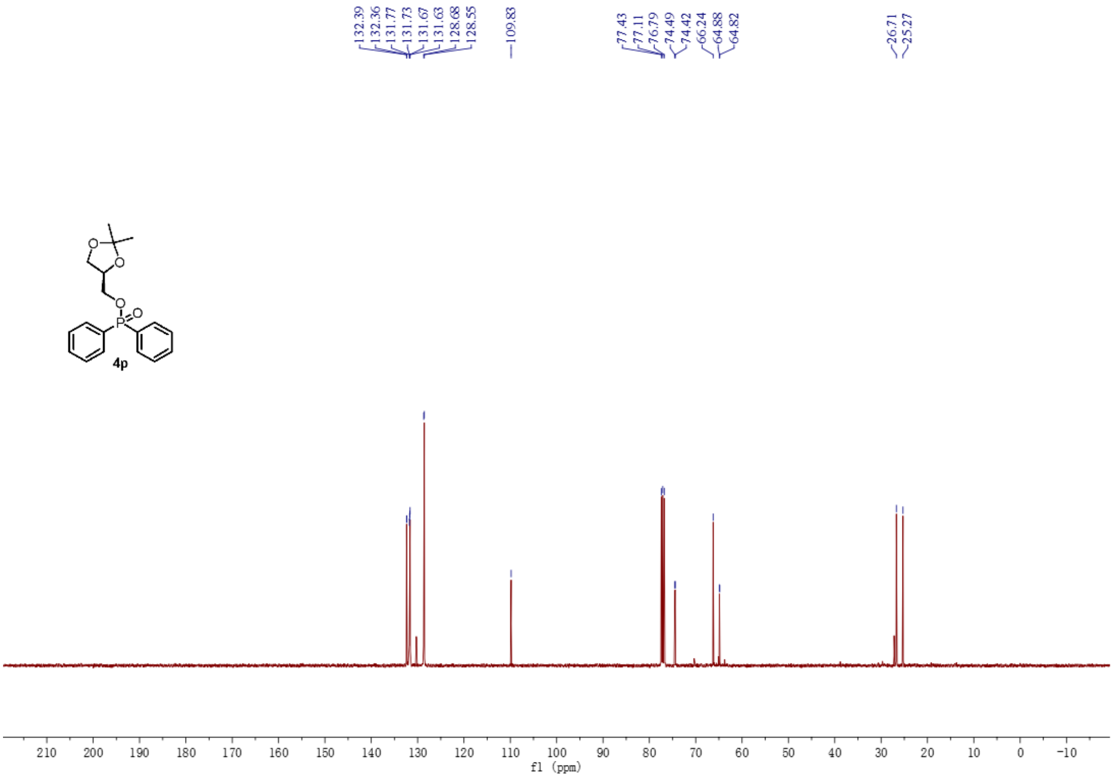

^{31}P NMR (121 MHz, $CDCl_3$) spectrum for 4p

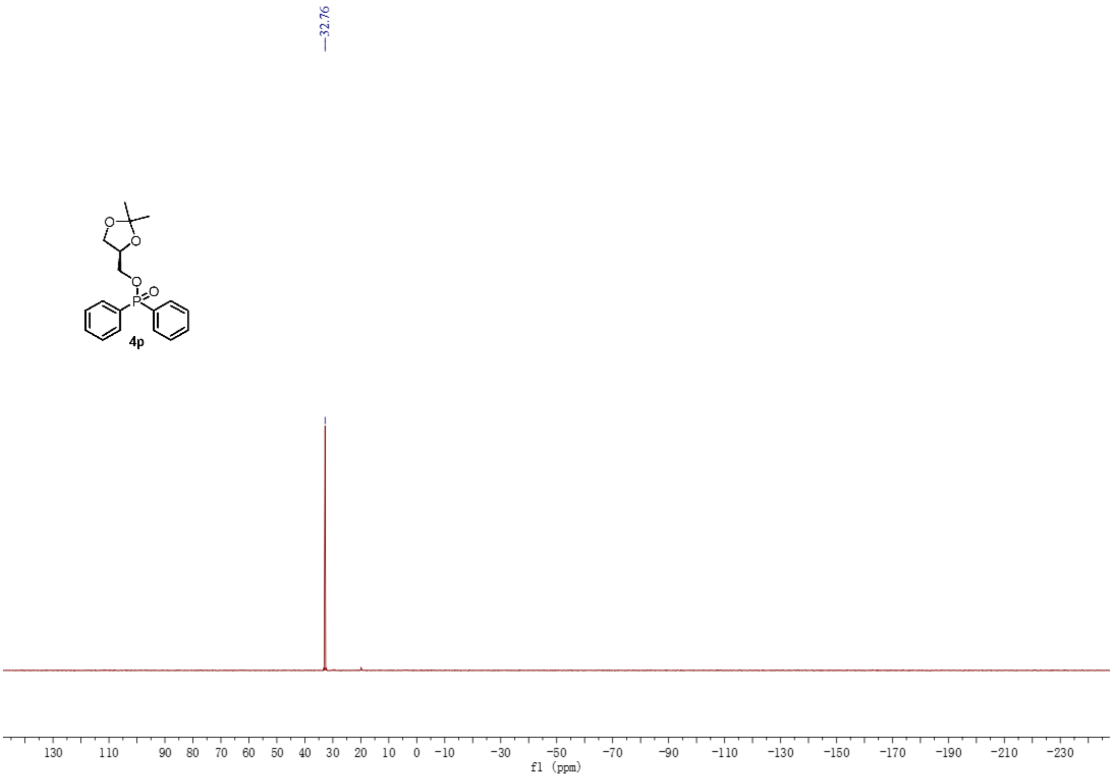

^{1}H NMR (400 MHz, $CDCl_3$) spectrum for 4q

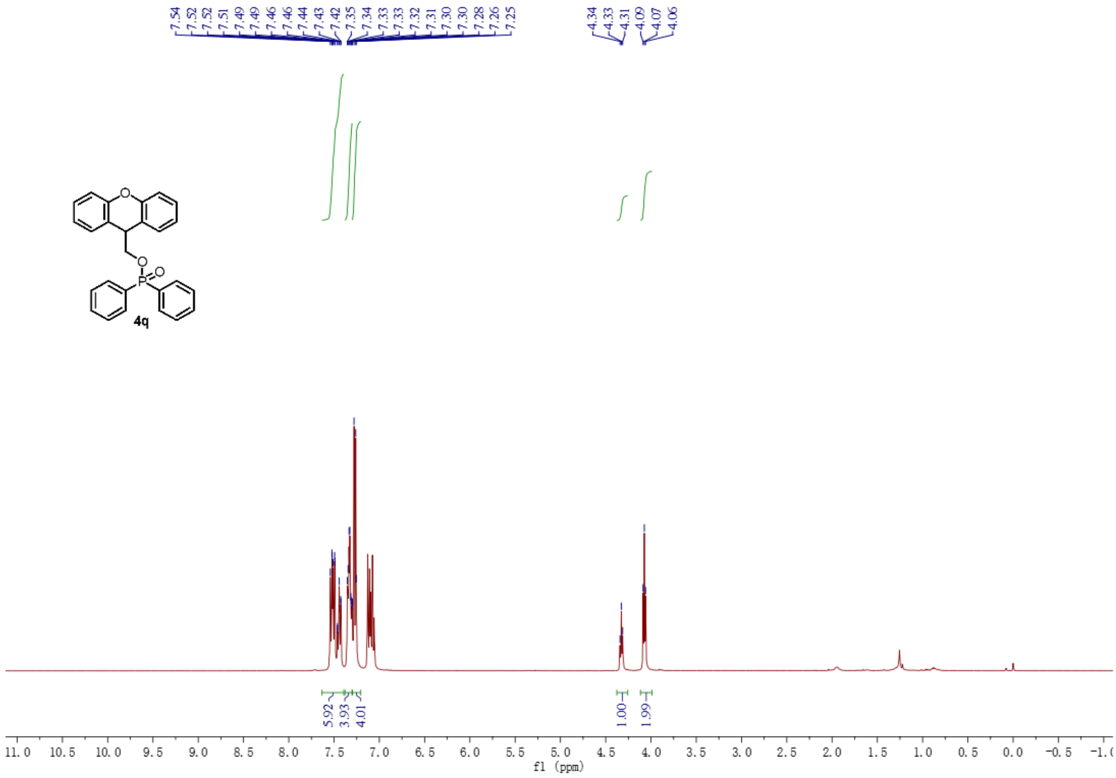

^{13}C NMR (101 MHz, $CDCl_3$) spectrum for 4q

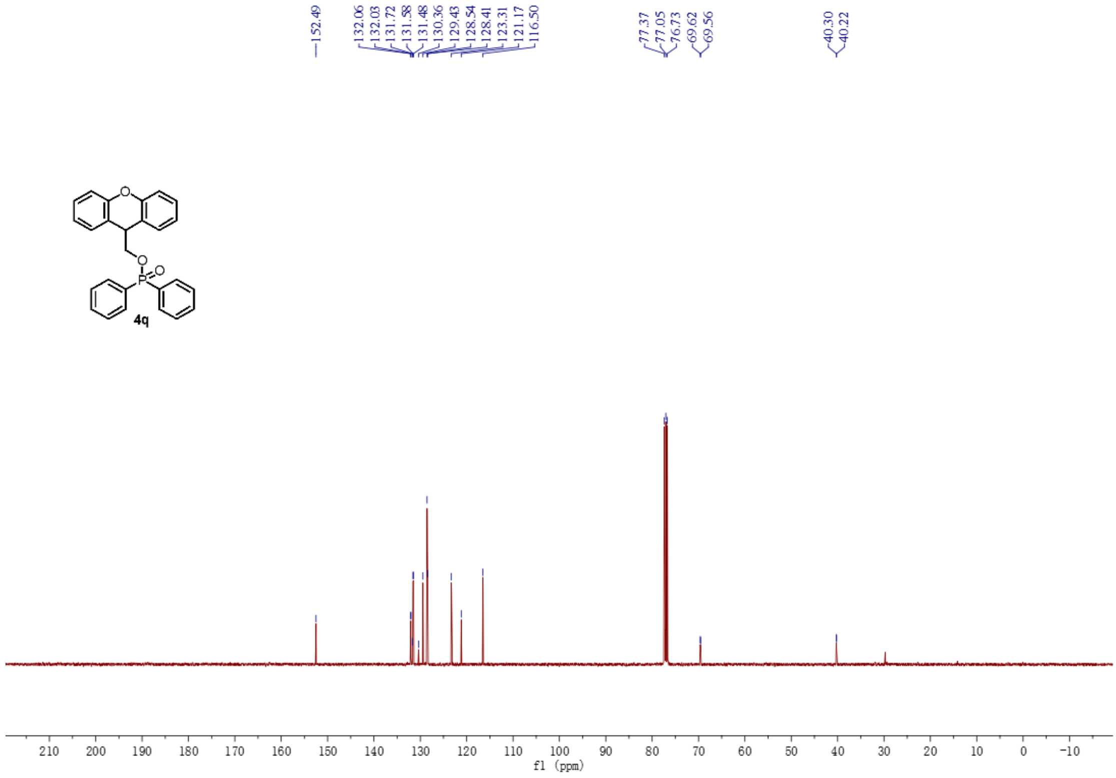

^{31}P NMR (121 MHz, $CDCl_3$) spectrum for 4q

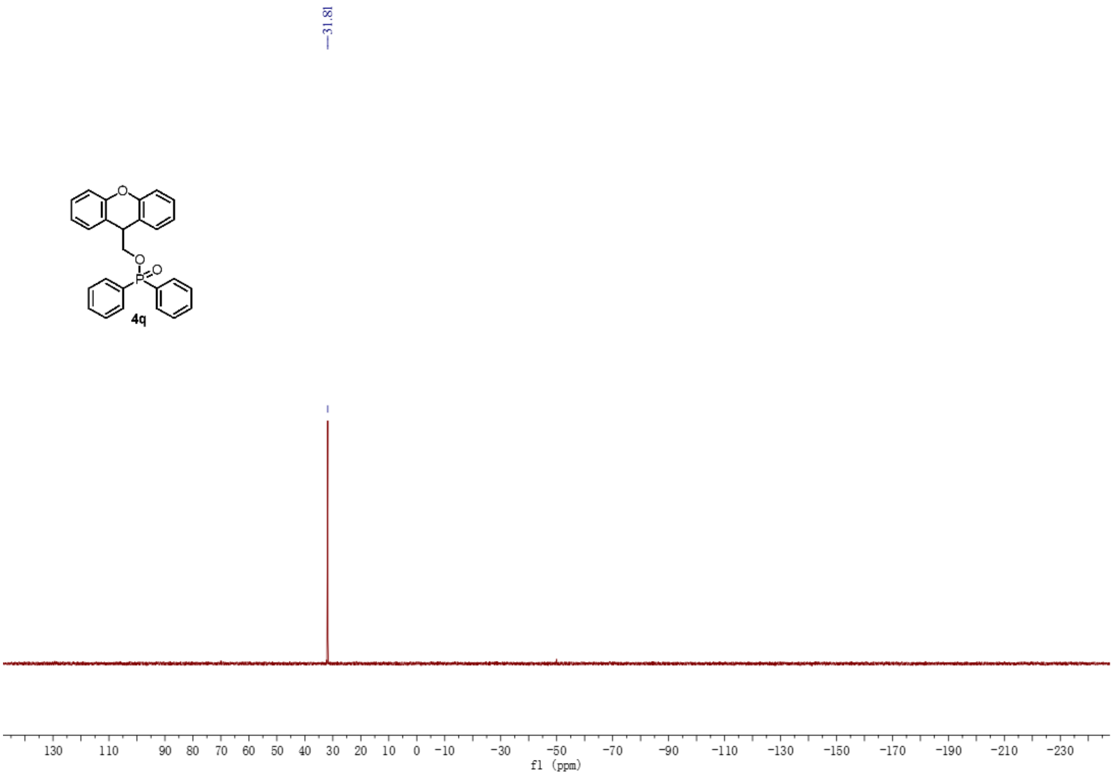

^{1}H NMR (400 MHz, $CDCl_3$) spectrum for 4r

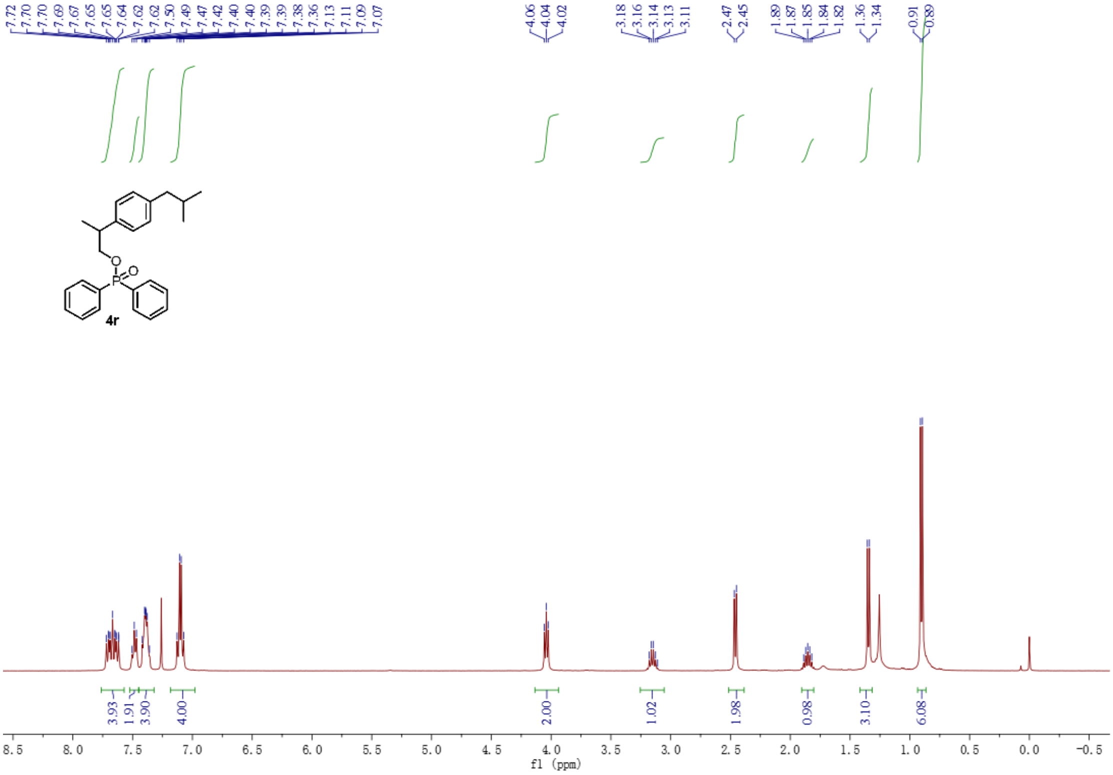

^{13}C NMR (101 MHz, $CDCl_3$) spectrum for 4r

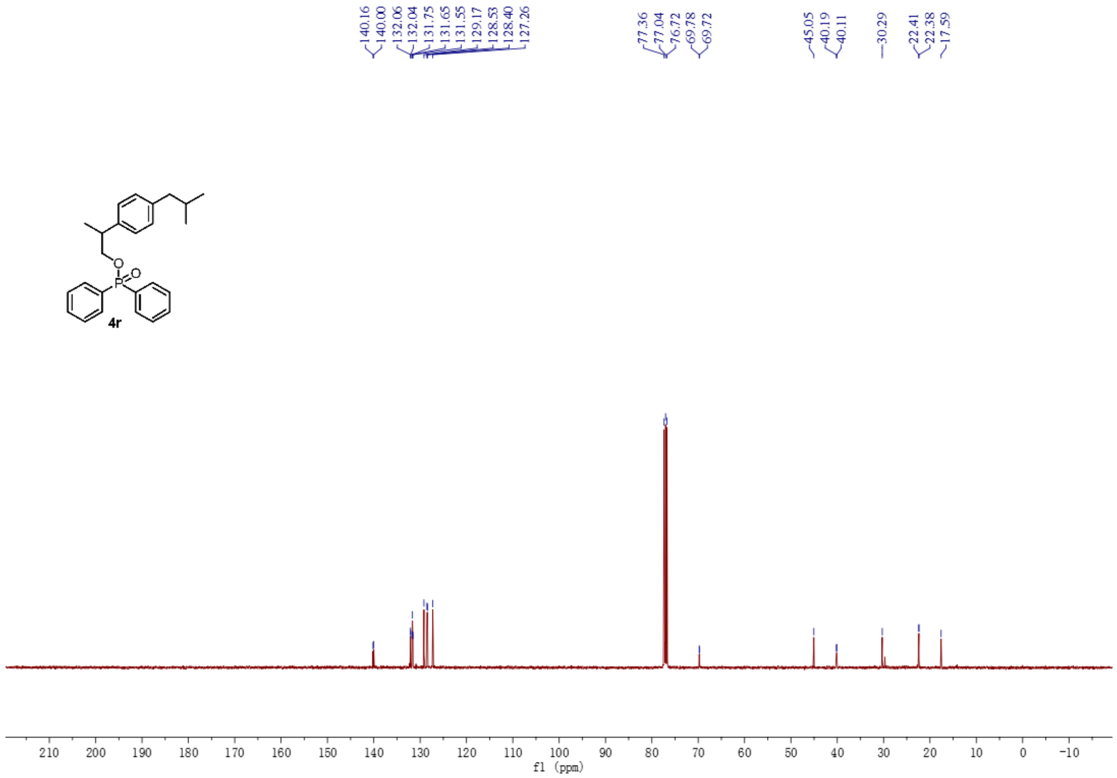

^{31}P NMR (121 MHz, $CDCl_3$) spectrum for 4r

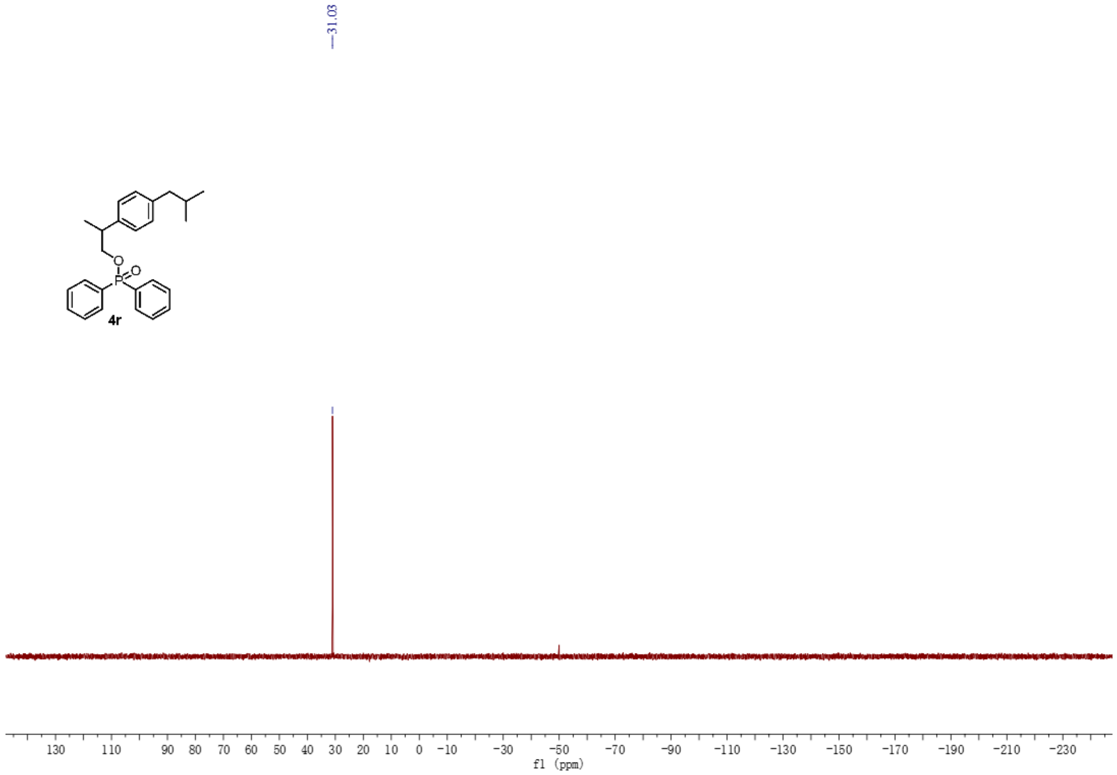

^{1}H NMR (400 MHz, CDCl3) spectrum for 4s

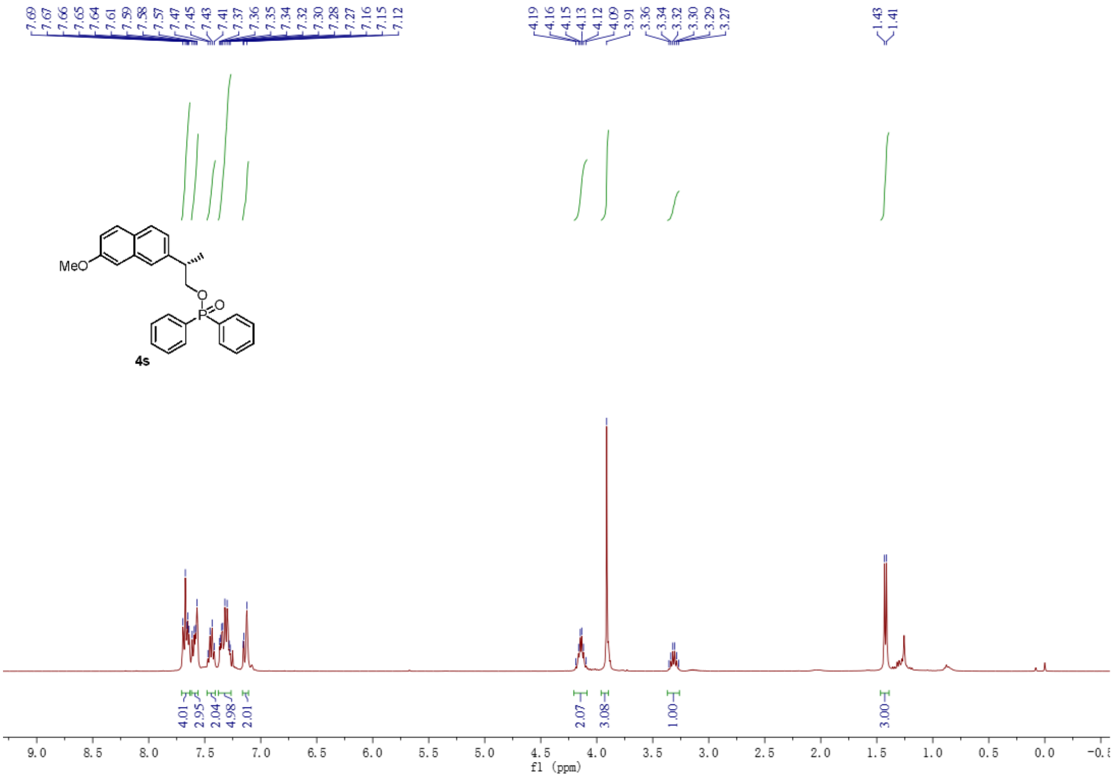

^{13}C NMR (101 MHz, CDCl3) spectrum for 4s

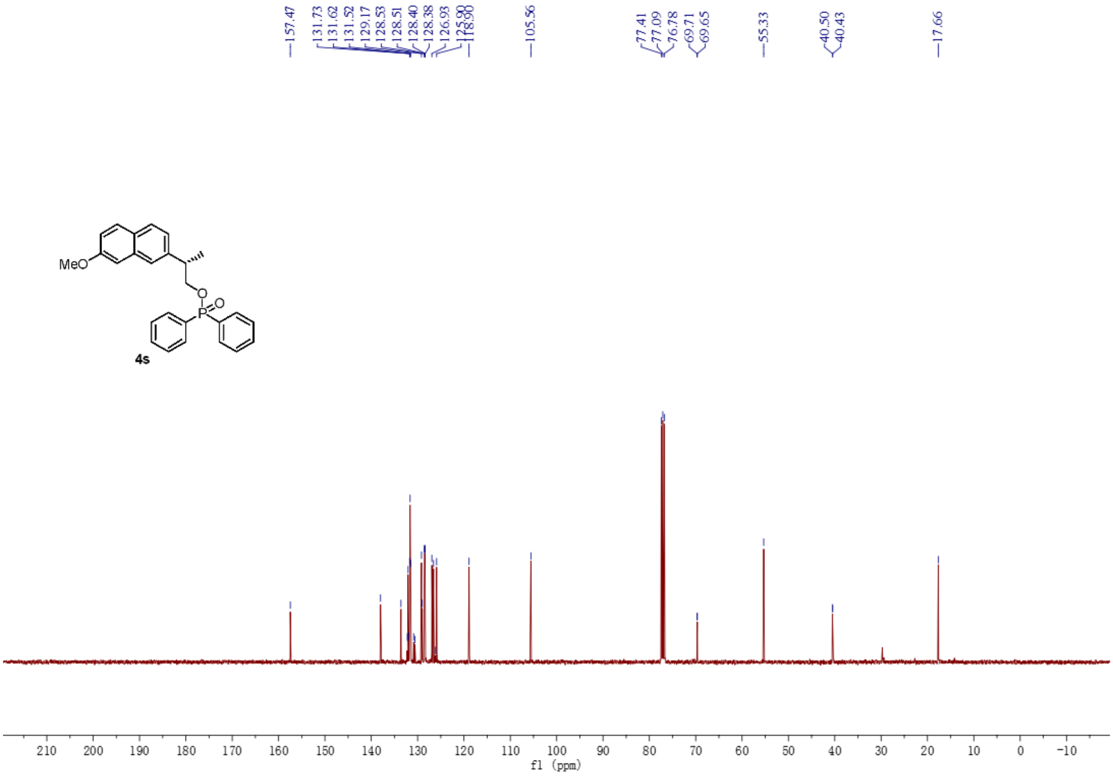

^{31}P NMR (121 MHz, $CDCl_3$) spectrum for 4s

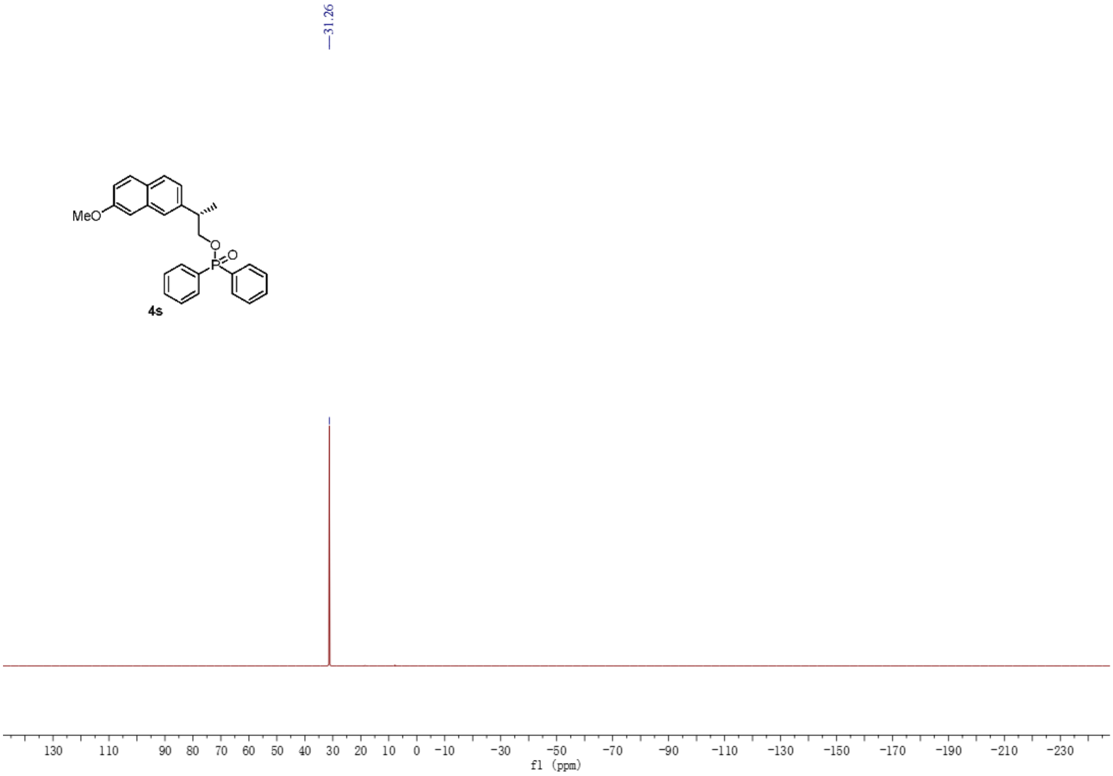

^{1}H NMR (400 MHz, $CDCl_3$) spectrum for 4t

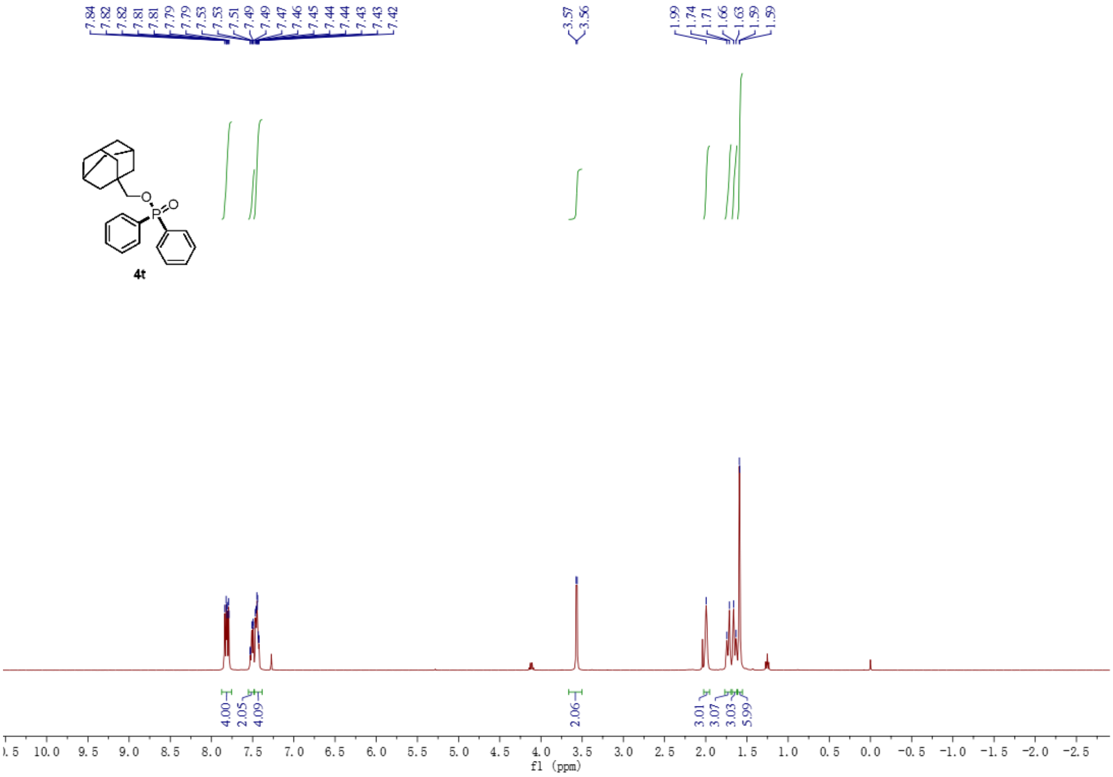

^{13}C NMR (101 MHz, $CDCl_3$) spectrum for 4t

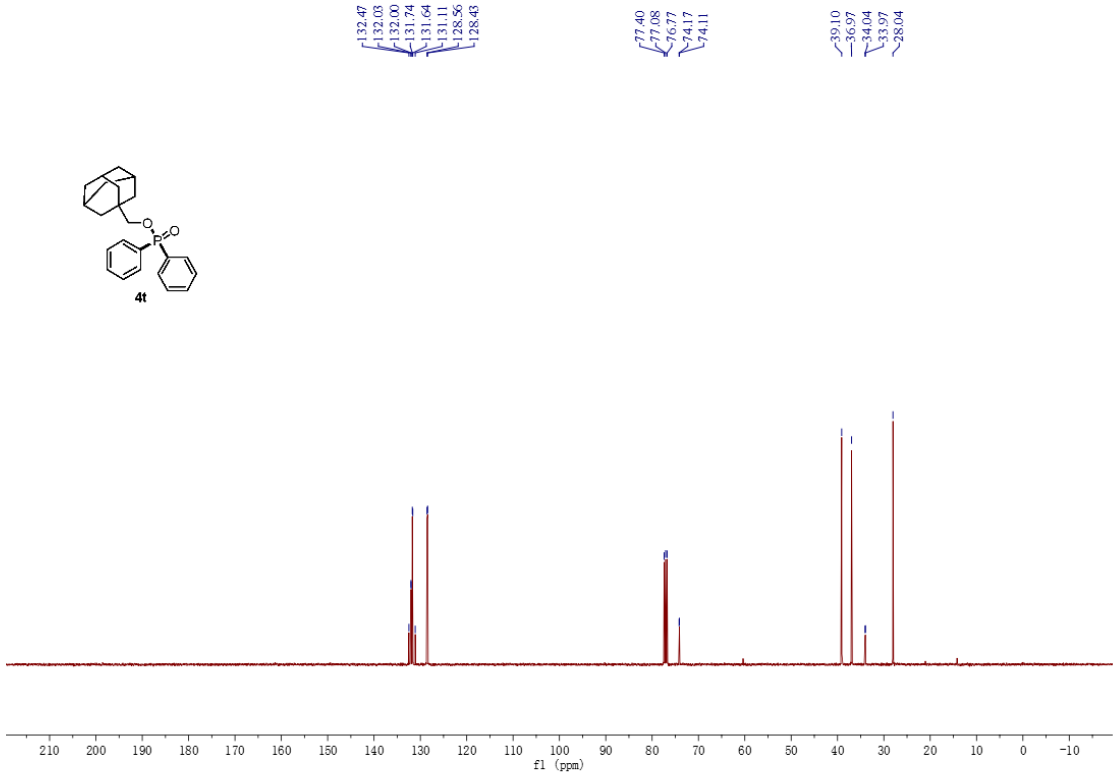

^{31}P NMR (121 MHz, $CDCl_3$) spectrum for 4t

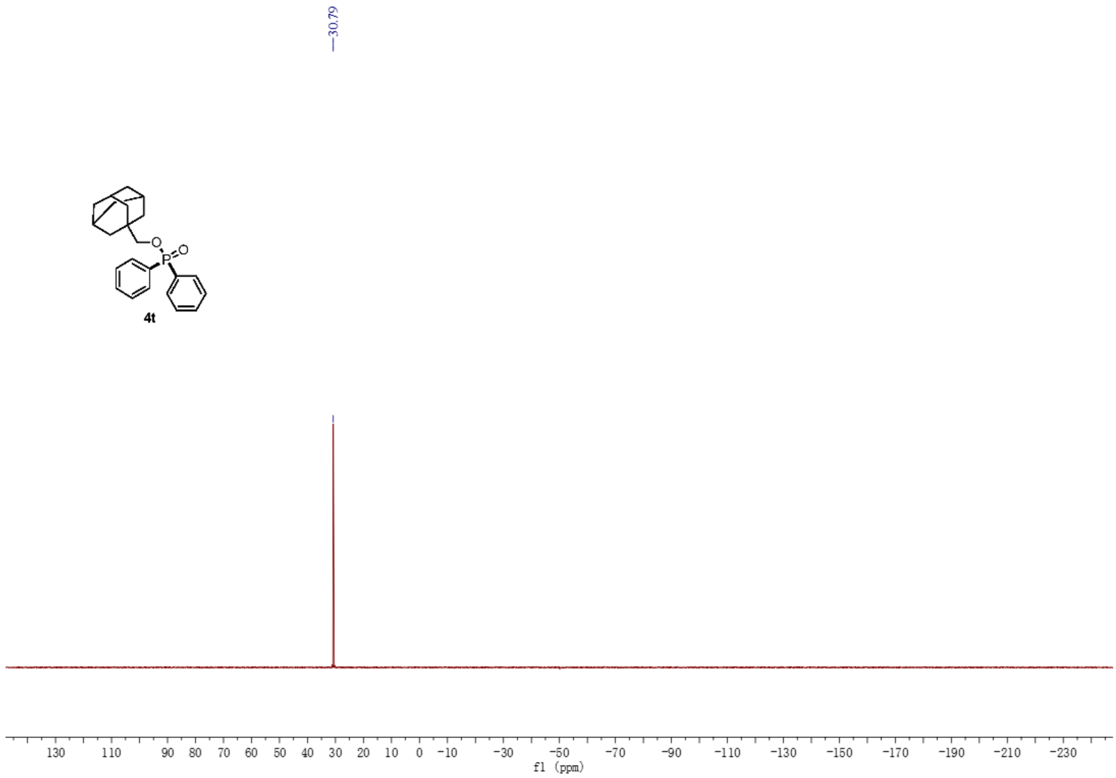

^{1}H NMR (400 MHz, CDCl$_3$) spectrum for 4u

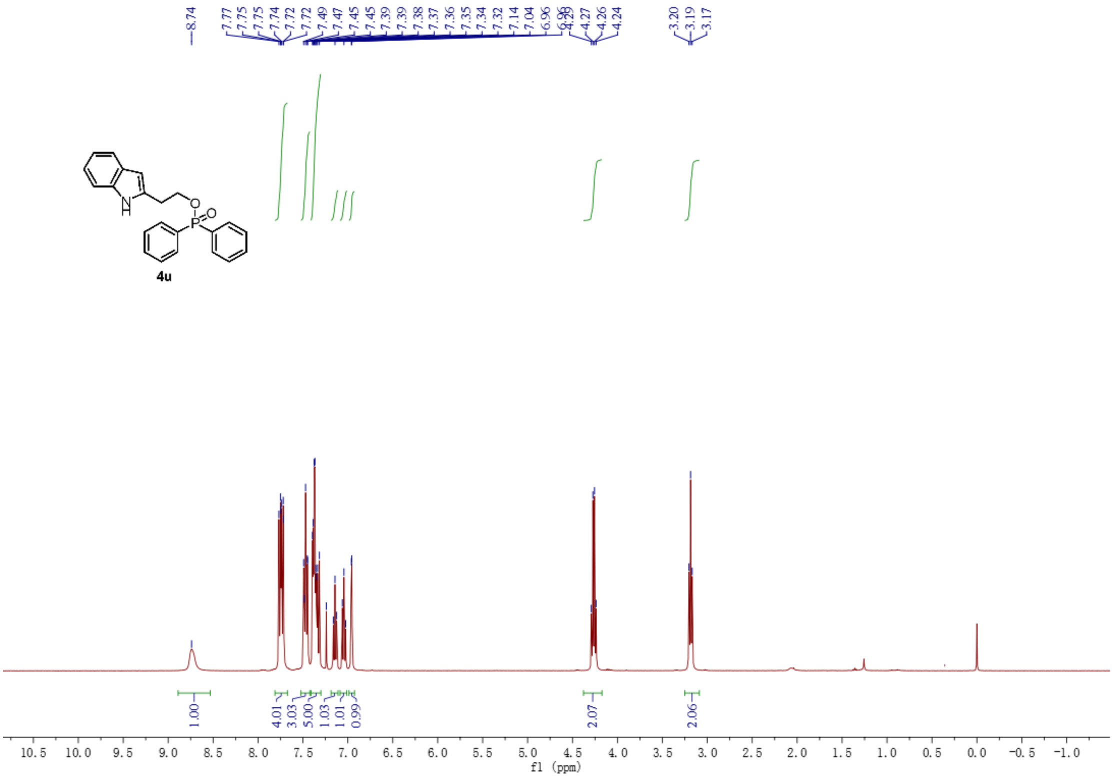

^{13}C NMR (101 MHz, CDCl$_3$) spectrum for 4u

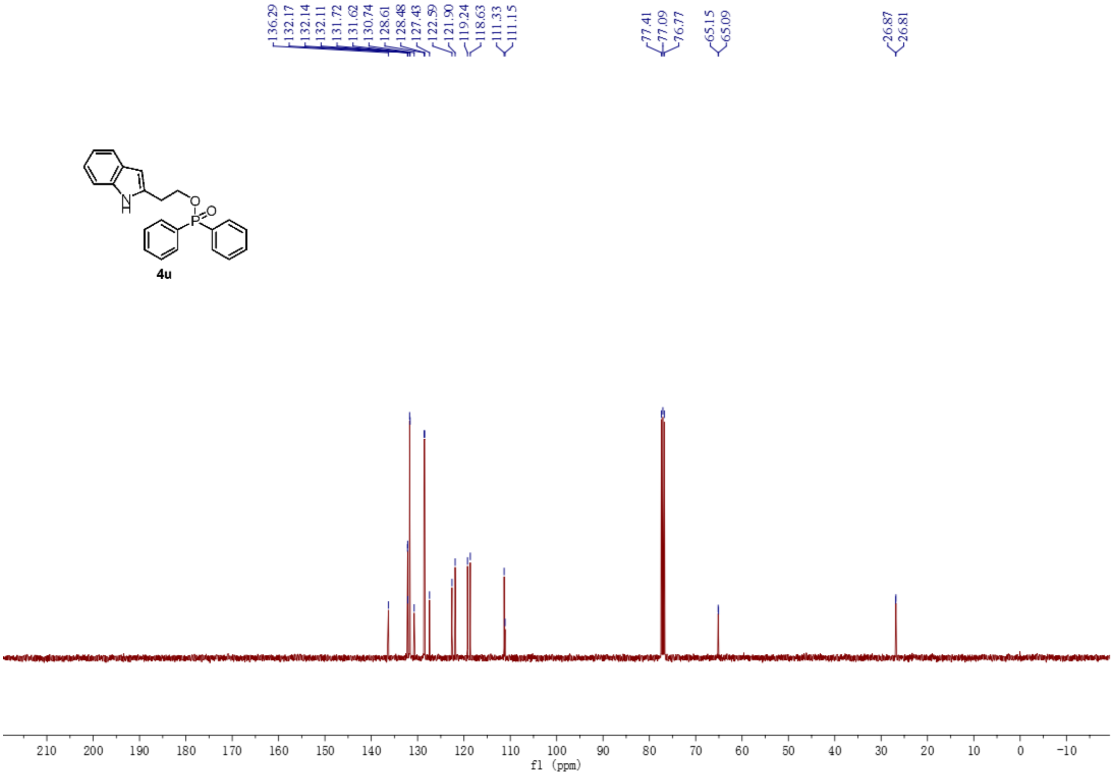

^{31}P NMR (121 MHz, CDCl$_3$) spectrum for 4u

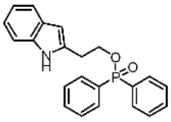

4u

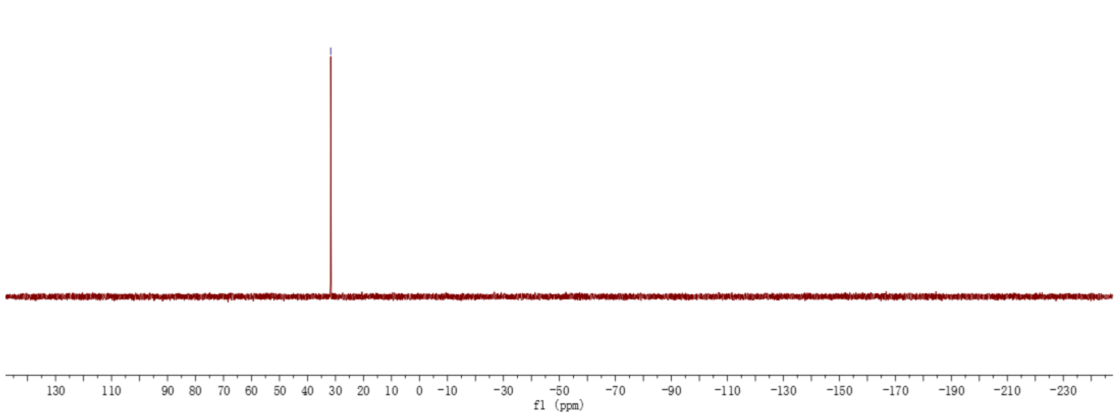

^{1}H NMR (400 MHz, CDCl$_3$) spectrum for 4v

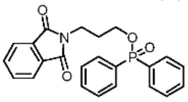

4v

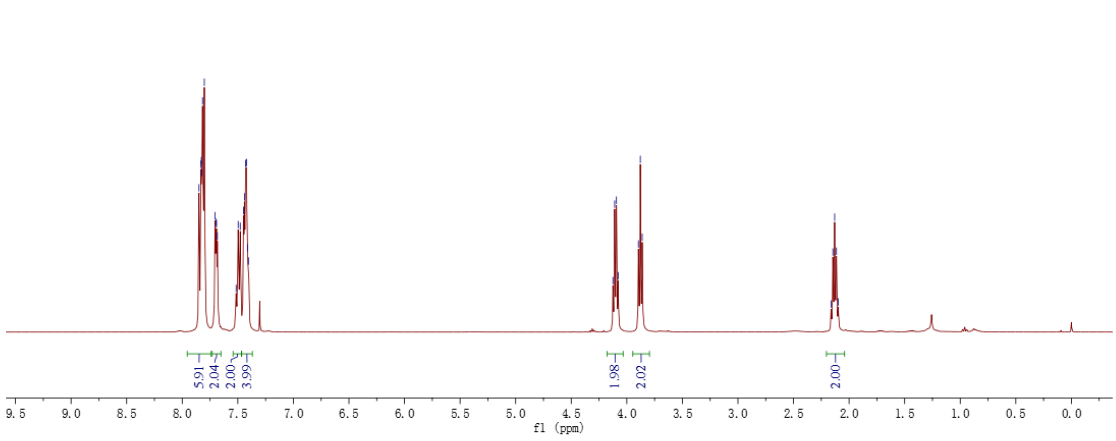

^{13}C NMR (101 MHz, $CDCl_3$) spectrum for 4v

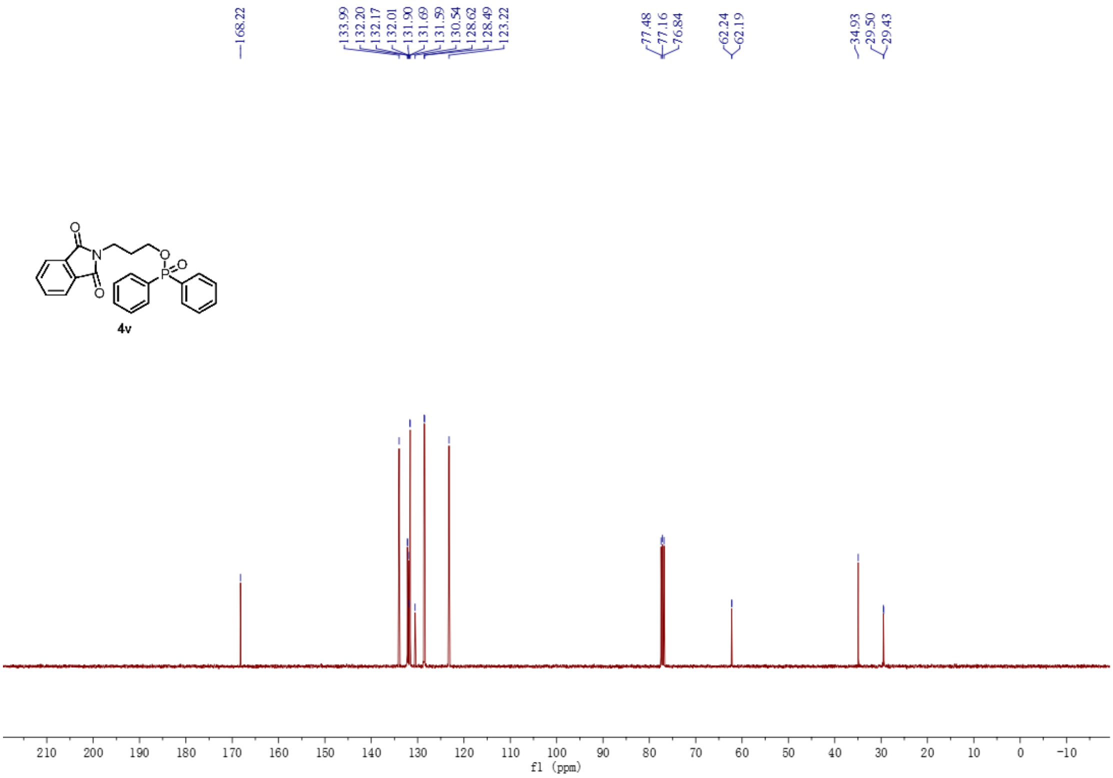

^{31}P NMR (121 MHz, $CDCl_3$) spectrum for 4v

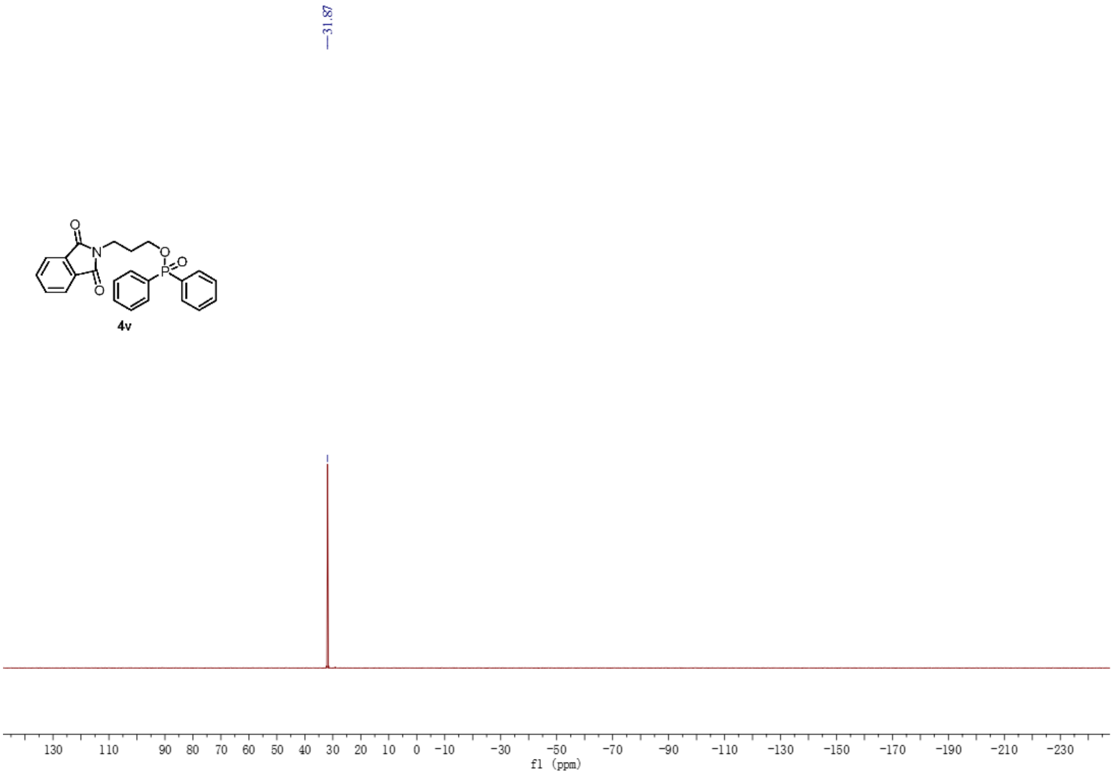

Supplement: Supplementary file 1 [file molecules-30-01564-s001.zip › molecules-3527998-supplementary.pdf]
